# A new lineage nomenclature to aid genomic surveillance of dengue virus

**DOI:** 10.1371/journal.pbio.3002834

**Published:** 2024-09-16

**Authors:** Verity Hill, Sara Cleemput, James Siqueira Pereira, Robert J. Gifford, Vagner Fonseca, Houriiyah Tegally, Anderson F. Brito, Gabriela Ribeiro, Vinicius Carius de Souza, Isabela Carvalho Brcko, Igor Santana Ribeiro, Iago Trezena Tavares De Lima, Svetoslav Nanev Slavov, Sandra Coccuzzo Sampaio, Maria Carolina Elias, Vi Thuy Tran, Duong Thi Hue Kien, Tuyen Huynh, Sophie Yacoub, Idrissa Dieng, Richard Salvato, Gabriel Luz Wallau, Tatiana S. Gregianini, Fernanda M. S. Godinho, Chantal B. F. Vogels, Mallery I. Breban, Mariana Leguia, Suraj Jagtap, Rahul Roy, Chanditha Hapuarachchi, Gaspary Mwanyika, Marta Giovanetti, Luiz C. J. Alcantara, Nuno R. Faria, Christine V. F. Carrington, Kathryn A. Hanley, Edward C. Holmes, Wim Dumon, Alex Ranieri Jerônimo Lima, Tulio de Oliveira, Nathan D. Grubaugh

**Affiliations:** 1 Department of Epidemiology of Microbial Diseases, Yale School of Public Health, New Haven, Connecticut, United States of America; 2 Emweb bv, Herent, Belgium; 3 Centro para Vigilância Viral e Avaliação Sorológica (CeVIVAS), Instituto Butantan, São Paulo, Brazil; 4 MRC-University of Glasgow Centre for Virus Research, Bearsden, Glasgow, United Kingdom; 5 Centre for Epidemic Response and Innovation (CERI), School of Data Science and Computational Thinking, Stellenbosch University, Stellenbosch, South Africa; 6 Department of Exact and Earth Sciences, University of the State of Bahia, Salvador, Brazil; 7 KwaZulu-Natal Research Innovation and Sequencing Platform (KRISP), Nelson R. Mandela School of Medicine, University of KwaZulu-Natal, Durban, South Africa; 8 Instituto Todos pela Saúde, São Paulo, Brazil; 9 Oxford University Clinical Research Unit, Ho Chi Minh City, Vietnam; 10 Arboviruses and Haemorrhagic Fever Viruses Unit, Virology Department, Institut Pasteur de Dakar, Dakar, Senegal; 11 Centro Estadual de Vigilância em Saúde da Secretaria de Saúde do Estado do Rio Grande do Sul (CDCT/CEVS/SES-RS), Rio Grande do Sul, Brazil; 12 Departamento de Entomologia, Instituto Aggeu Magalhães (IAM)-Fundação Oswaldo Cruz-FIOCRUZ, Recife, Brazil; 13 Department of Arbovirology, Bernhard Nocht Institute for Tropical Medicine, WHO Collaborating Center for Arbovirus and Hemorrhagic Fever Reference, Hamburg, Germany; 14 National Reference Center for Tropical Infectious Diseases. Bernhard, Hamburg, Germany; 15 Genomics Laboratory, Pontificia Universidad Católica del Peru, Lima, Peru; 16 Department of Chemical Engineering, Indian Institute of Science, Bengaluru, Karnataka, India; 17 Center for BioSystems Science and Engineering, Indian Institute of Science, Bengaluru, Karnataka, India; 18 Environmental Health Institute, National Environment Agency, Singapore; 19 Department of Applied Sciences, Mbeya University of Science and Technology (MUST), Mbeya, Tanzania; 20 Department of Sciences and Technologies for Sustainable Development and One Health, Universita Campus Bio-Medico di Roma, Roma, Italy; 21 Instituto René Rachou, Fundação Oswaldo Cruz, Minas Gerais, Brazil; 22 Climate Amplified Diseases and Epidemics (CLIMADE), Minas Gerais, Brazil; 23 MRC Centre for Global Infectious Disease Analysis, Jameel Institute, Imperial College London, London, United Kingdom; 24 Instituto de Medicina Tropical, Faculdade de Medicina da Universidade de São Paulo, São Paulo, Brazil; 25 Department of Preclinical Sciences, The University of the West Indies, St. Augustine Campus, St. Augustine, Trinidad and Tobago; 26 Department of Biology, New Mexico State University, Las Cruces, New Mexico, United States of America; 27 Sydney Institute for Infectious Diseases, School of Medical Sciences, The University of Sydney, Sydney, Australia; 28 Department of Global Health, University of Washington, Seattle, Washington, United States of America; 29 Yale Institute for Global Health, Yale University, New Haven, Connecticut, United States of America; 30 Department of Ecology and Evolutionary Biology, Yale University, New Haven, Connecticut, United States of America

## Abstract

Dengue virus (DENV) is currently causing epidemics of unprecedented scope in endemic settings and expanding to new geographical areas. It is therefore critical to track this virus using genomic surveillance. However, the complex patterns of viral genomic diversity make it challenging to use the existing genotype classification system. Here, we propose adding 2 sub-genotypic levels of virus classification, named major and minor lineages. These lineages have high thresholds for phylogenetic distance and clade size, rendering them stable between phylogenetic studies. We present assignment tools to show that the proposed lineages are useful for regional, national, and subnational discussions of relevant DENV diversity. Moreover, the proposed lineages are robust to classification using partial genome sequences. We provide a standardized neutral descriptor of DENV diversity with which we can identify and track lineages of potential epidemiological and/or clinical importance. Information about our lineage system, including methods to assign lineages to sequence data and propose new lineages, can be found at: dengue-lineages.org.

## Abstract 2

Dengue virus (DENV) is causing increasingly larger and more widespread epidemics, which makes it critical to track the virus using genomic surveillance, but there is currently no suitable system to classify circulating variants. This Consensus View proposes a new DENV nomenclature system and free online tools to identify and track lineages of potential epidemiological and/or clinical importance.

## Introduction

Dengue virus (DENV: *Flaviviridae*; *Orthoflavivirus*) inflicts the heaviest global burden on public health of any mosquito-borne virus, causing more than 100 million infections per year [1]. Dengue incidence is increasing worldwide, with major outbreaks across endemic regions in the tropics in 2023 [2], and sustained local transmission in non-endemic regions such as the state of Florida in the United States [3] and Italy [4,5]. As DENV continues to spread, tracking the evolution at a high resolution is key to understanding its transmission patterns on local, regional, and global scales.

Dengue virus in its human-endemic cycle consists of 4 serotypes (DENV-1-4) that likely correspond to at least 4 successful spillover events from its ancestral sylvatic cycle (Box 1. Glossary) that took place several centuries ago [6]. Each serotype includes several genotypes that were designated in the late 1990s and early 2000s based on partial genetic sequences [7,8]. In addition, DENV-2 and DENV-4 include genotypes encompassing viruses from the sylvatic cycle. These serotypes and genotypes have provided the basis for decades of work characterizing the natural history, phenotypic diversity, and transmission dynamics of DENV. However, with recent large increases in global sequencing capacity and its integration into public health systems, additional granularity of DENV diversity is required. Several previous studies have already classified sub-genotypic diversity on a country or regional level (e.g., [9–11], but there is a need to standardize this discussion between research groups and countries to aid communication and facilitate identification of common patterns. The continued evolution and spread of DENV has led to the emergence of distinct evolutionary lineages within recognized genotypes. Further, with the implementation of interventions (e.g., vaccines, *Wolbachia*-infected mosquitoes to suppress DENV transmission) that may eventually select for specific viral lineages, it is imperative to have a precise and common language to monitor continued DENV transmission in different spatiotemporal scales, and that this is communicable to clinicians and public health officials who may not have a background in genomics.

**Box 1. Glossary**

**Amplicon drop-outs**

The situation (during a gene amplification technique) when an amplicon, i.e., a segment of genetic material that undergoes amplification in the process of “amplicon sequencing,” is not amplified. The region of the genome covered by that amplicon therefore is not sequenced and is displayed as a series of “N”s in the consensus genome.

**Antigenic distance**

Difference between 2 antigens, i.e., the part of a pathogen or otherwise that the immune system responds to, therefore, a proxy of the difference in immune response to 2 pathogens.

**Autochthonous transmission**

Local transmission of a pathogen, i.e., not a travel-derived case.

**Genotype**

The genetic make-up of an organism. Here, it is the second level of dengue virus classification based on their nucleotide sequence. There are multiple per each serotype, denoted by Roman numerals.

**Indel**

An insertion or deletion of a nucleotide base.

**Monophyly**

A clade in a phylogenetic tree that contains all of the descendants of a common ancestor, and none that are not descendants.

**Recombination**

Exchange of genetic material between 2 organisms or virus particles, leading to a genome which has mixed ancestry.

**Serotype**

A way of grouping microorganisms, such as bacteria or viruses, based on molecules found on their surfaces. In this article, it is the highest level of dengue virus classification, based on their surface antigens. There are 4 dengue virus serotypes: DENV-1, DENV-2, DENV-3, and DENV-4.

**Subclade**

Subdivision of a clade, which is an arbitrary grouping of branches on a phylogeny.

**Substitution**

A mutation where one nucleotide base is replaced by a different one.

**Sylvatic cycle**

Viral transmission between nonhuman primates and mosquitoes living in trees.

Here, we propose a system for the classification and nomenclature of DENV lineages that builds on existing serotype and genotype (see Box 1) classifications to (1) provide additional temporal and spatial granularity; and (2) standardize the discussion of important diversity globally. We take inspiration from the design of the pango nomenclature system, a hierarchical lineage system set up to track SARS-CoV-2 evolution [12], as well as lessons learned from its design and implementation. It also borrows from older systems, such as the H5 influenza nomenclature [13]. We discuss the design, validation, and application of our proposed DENV lineage system, show how it enhances resolution when monitoring circulating lineages, and introduce tools enabling end-users to assign lineages to their own sequences. By making the system compatible with existing classifications and showcasing its utility, we aim to achieve widespread uptake and introduce a truly standardized global language with which to discuss DENV genetic diversity.

### Previously defined genotypes provide useful but not sufficient resolution

**Fig 1:**
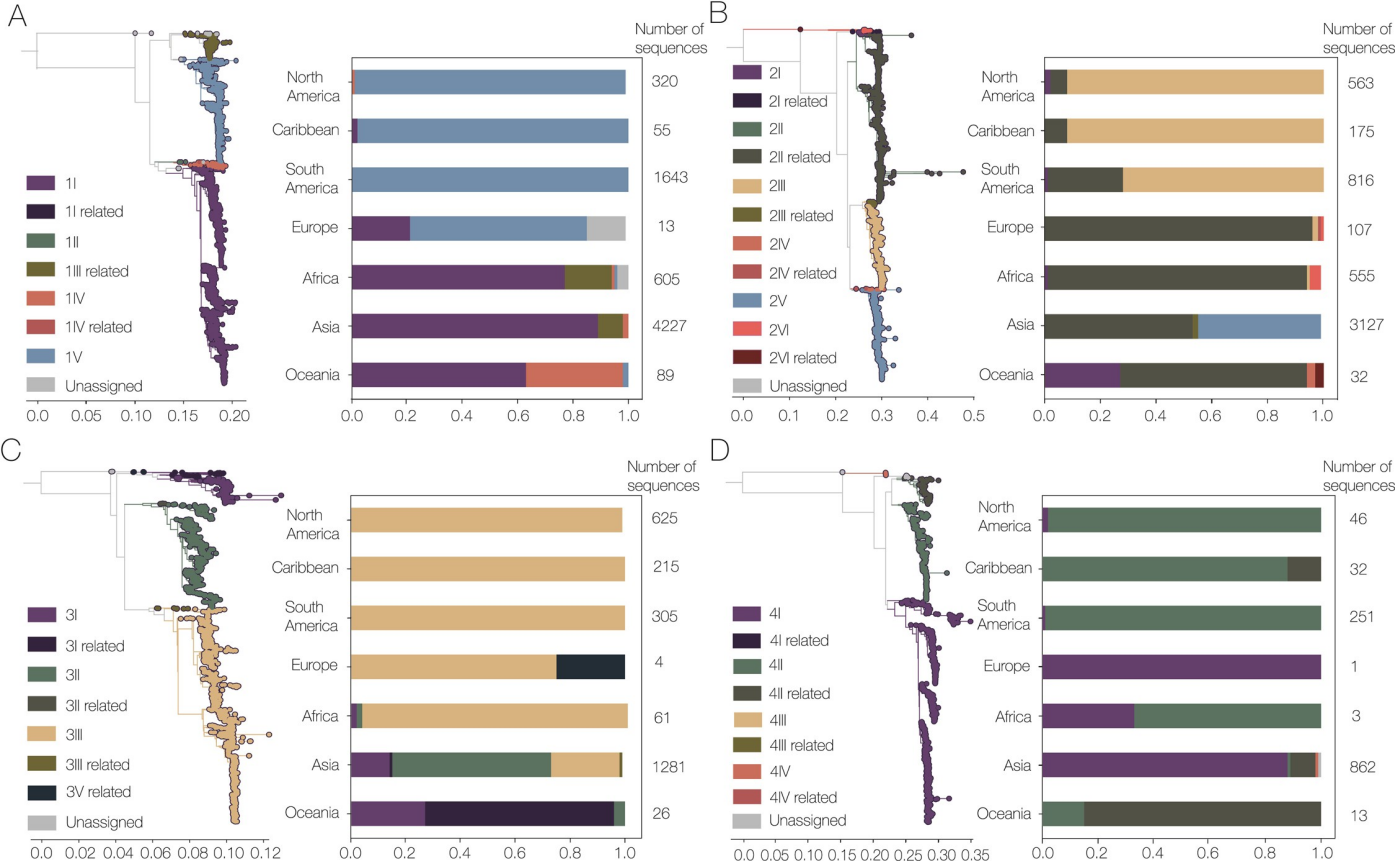
Current system of classifying dengue virus serotypes into distinct genotypes provides insufficient geographical resolution. Maximum likelihood phylogenetic trees scaled by genetic distance for each DENV serotype: (A) DENV-1, (B) DENV-2, (C) DENV-3, and (D) DENV-4. Trees are colored by the current genotype classification obtained using the Genome Detective Dengue Virus Typing Tool [22,23]. Bar charts indicate the frequency of whole genomes sampled in each continent assigned to each existing genotype, and numbers at the end of each bar indicate the number of sequences in each data set. Note that every serotype has a dominant genotype across the Americas (i.e., North America, Caribbean, and South America). “Related” refers to sequences that are not reliably placed into the clade as there is considerable bootstrap support for the clade without the query as well as with the query. We note that there are no whole genomes in this data set (see Methods) which are assigned DENV-1III, only to its related genotype.

DENV is currently classified into 4 serotypes, which in turn include varying numbers of genotypes: 6 for DENV-1, 6 for DENV-2, 5 for DENV-3, and 4 for DENV-4 (Fig 1). Genotype classification systems were originally based on greater than 6% pairwise genetic distance within the genotype, using a 240 nucleotide sequence (i.e., a single amplicon) of the envelope (E)/nonstructural protein 1 (NS1) protein coding region, as this arbitrary threshold split then-known DENV-1 diversity into manageable groups [7]. As more sequence data was generated, these were replaced by systems based on entire protein-coding regions, especially E, with which many of the current genotypes were designated 20 to 30 years ago [14–16]. Maintenance of this system is through continued usage, and there is no official body which regulates genotype designation.

This serotype/genotype nomenclature system still holds well with newer whole genome sequences, with some geographic distinction between continents. For example, within DENV-1 we found that the Americas are dominated by genotype V, whereas Asia and Oceania have more sequences of genotype I (Fig 1A). Further, much of the existing research uses genotypes to characterize circulating variants [17], ensure adequate genomic diversity for sequencing panels [18,19], identify new introductions leading to outbreaks [20], explore differences in viral fitness, and disease association [21]. However, there has been a huge increase in publicly available sequence data, both in terms of the number and the region from which samples are being sequenced, since these genotype classification systems were established. This has led to a similarly large increase in the known genetic diversity within each serotype. In particular, there are now groups of genomes that do not reliably fall into genotypes: 6.41% of DENV-1 sequences, 12.8% of DENV-2, 2.14% of DENV-3, and 9.75% of DENV-4 sequences are related to defined genotypes, but cluster basally to them (designated as “related” by Genome Detective Dengue Virus Typing tool [22,23]; Fig 1). A smaller number of genomes in each serotype do not fall into any currently defined genotype and are designated as “Unassigned” (0.60% DENV-1, 0.25% DENV-2, 0.14% DENV-3, and 0.10% DENV-4). This increase in the known genetic diversity of circulating lineages also leads to complexities on the sub-genotypic level, as some genotypes are now very large and highly diverse—5 out of 17 genotypes contain more than 1,000 whole genome sequences. Of particular note, DENV-1 genotype I contains 4,281 published sequences, which is more than 3 times the size of the entire DENV-4 whole genome data set that we used here (*n* = 1,215). The large number of genomes in many of these genotypes, combined with increased air travel and the expanded range of the mosquito vectors [24,25], lead us to conclude that genotypes alone do not provide sufficient resolution for many epidemiological questions (Fig 1). For example, the current genotype assignment does not provide additional epidemiological information compared to the serotype for DENV-1, DENV-2, and DENV-3 in the Americas, as they are dominated overwhelmingly by a single genotype for each serotype. Indeed, many previous studies in this region have already named sublineages within genotypes (e.g., lineage classification systems in Brazil and Nicaragua [9,26–29]). Therefore, while genotypes provide an important base for research, there is clearly a need for an updated classification system to include newly recognized global diversity and provide additional sub-genotype resolution in a systematic and standardized way.

### New lineage classification system design

**Fig 2:**
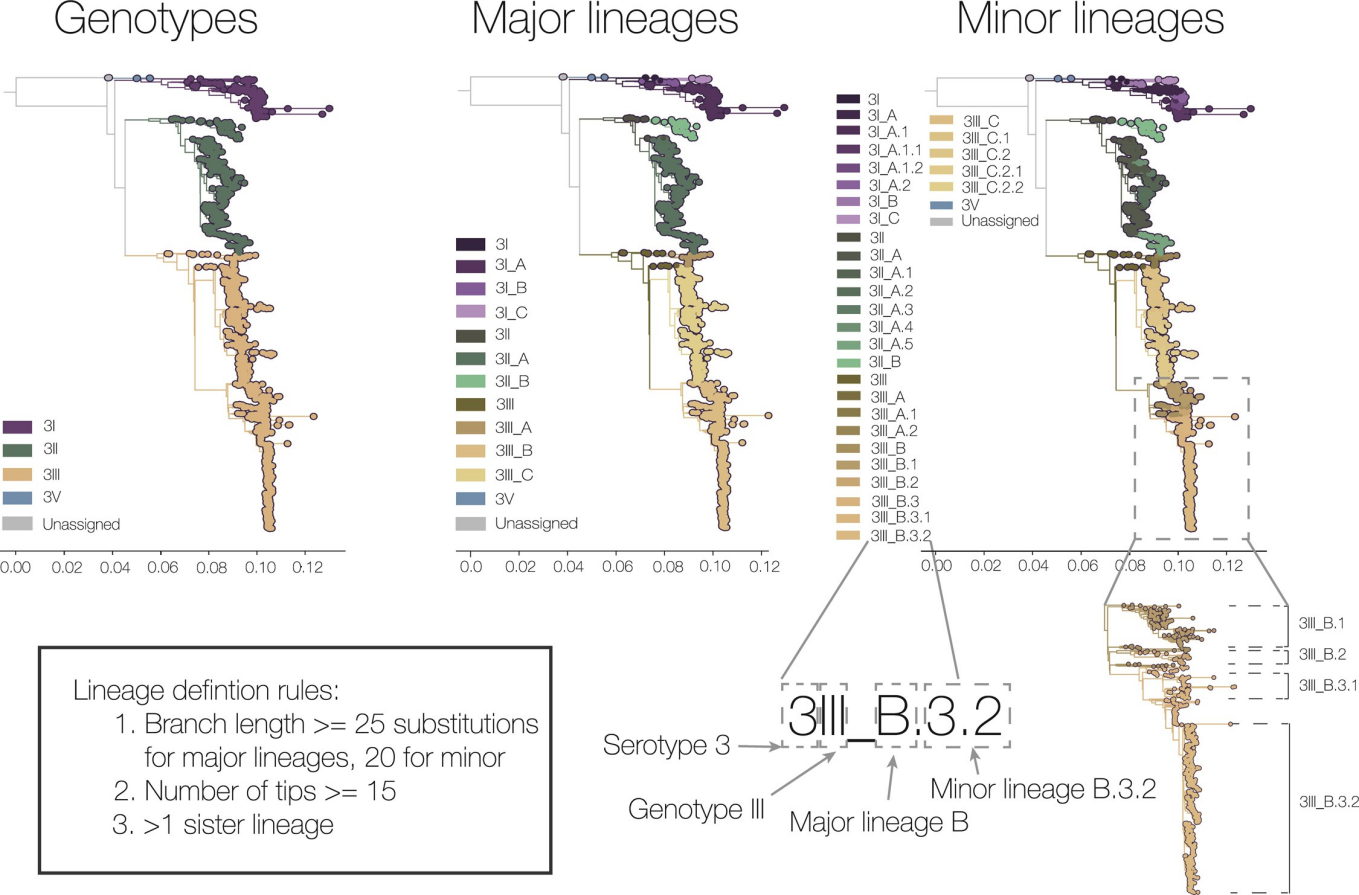
Description of proposed dengue virus lineage classification system using serotype 3 as an example. Genotype-level classification has been expanded to include most previously unassigned genomes. Two additional layers of classification have been proposed, major and minor lineages, the rules of which are shown here. The nomenclature is described here in its shorthand form (e.g., DENV-3III_C.2), with each element highlighted by a dotted box. The new lineage classifications for serotypes 1, 2, and 4 are shown in S2 Fig.

To better describe the circulating diversity of DENV, we propose a new system that builds on the existing serotype/genotype system discussed above (Fig 2). First, we updated the genotype definitions so that fewer genomes are unassigned or ambiguously classified as “related.” Then, we added 2 additional layers of resolution within the genotypes, major and minor lineages, with associated nomenclature.

We found that many DENV sequences fall basal to the genotype-defining nodes, meaning that they do not fit cleanly within the current classification system and are thus classified as “related” to a genotype (Fig 1). We identified 16 categories of “related” genotypes within our global DENV genomic data set. We therefore moved the genotype-defining node closer to the root of the tree so that they were included in the main definition of the genotype. We did not remove any of the already defined genotypes, including a newly proposed DENV-1 genotype VI [30]. We also propose a new genotype, named DENV-1 VII, for a clade containing 27 sequences primarily from the Democratic Republic of the Congo. Even after our adjustments, some unassigned sequences remain (2 sequences for each serotype, DENV-1 = 0.037%, DENV-2 = 0.051%, DENV-3 = 0.095%, DENV-4 = 0.20%), but these are mostly basal to other defined genotypes or singleton outliers. For DENV-1, DENV-2, and DENV-4, the sequences are sylvatic sequences from Malaysia, Borneo, and Australia. For DENV-3, these sequences are older (1953 and 1963) from Puerto Rico. These updated genotype definitions also still fit the original arbitrary definition of less than 6% pairwise genetic distance within a genotype, even across the whole genome. It is of note, however, that the pairwise distance within genotypes is highly variable (S1A Fig), and this variation is not related to the number of sequences within a genotype (S1B Fig).

We propose that Roman numerals are consistently used to refer to genotypes, thereby removing the current geography-based names for DENV-2 genotypes. We are motivated by 2 key reasons: (1) some of these genotypes are now very widespread and are not limited to the region that the name implies; and (2) geographical names can lead to discrimination, especially when they cause large outbreaks, and as such are against best practices for naming pathogens [31]. We use the standard Roman numerals for these genotypes instead, and the comparison between the systems can be found in S1 Table.

The new genotype definitions that we propose succeed in reducing the number of unassigned DENV sequences. Large geographical spaces, however, are still dominated by single genotypes within a serotype. Therefore, we propose 2 additional levels of classification: major and minor lineages (Figs 2 and S2). Major lineages, designated using letters of the Roman alphabet, are designed to help answer regional scale questions. Minor lineages, designated using numbers and full-stops, provide more fine-scale resolution, and therefore have a nomenclature more similar to SARS-CoV-2 pango lineages [12]. Importantly, like the existing genotypes, this lineage nomenclature system is evolutionarily neutral—i.e., they are designated based entirely on phylogenetic metrics and not on any possible phenotypic differences. This provides an *a priori* system for naming clades, thereby providing a framework to identify possible phenotypic changes when they arise. For example, the sudden growth of a single clade, which may indicate a change in transmissibility or immune evasion, may be more easily identified when many sequences are rapidly assigned to a lineage that is named consistently throughout the world. We also note that lineage definitions are based on the nodes of the phylogeny, rather than the tips.

Major and minor lineages are strictly hierarchical. We use similar defining rules for both levels of at least (1) 15 sequences (arbitrary cutoff); (2) 25 inferred nucleotide substitutions for major lineages and 20 for minor lineages (across the whole genome) along the ancestral branch (arbitrary); and (3) one sister lineage at the same level—in other words, there cannot be an A lineage without a B lineage (S3 Fig). The first 2 rules aim to capture epidemiologically important lineages, which are stable between iterations of phylogenetic inference. The high phylogenetic distance is possible due to the high genomic diversity of DENV, and builds on experience in the pre-variant era of SARS-CoV-2. Due to its low global genetic diversity, SARS-CoV-2 lineages were defined on a single evolutionary event (i.e., a substitution, an indel, or a recombination event, see Box 1 [12]) and so would sometimes break monophyly (see Box 1) when new trees were inferred, causing issues with communication. Our thresholds are also high to avoid high levels of nesting in the names of the lineages at this stage, also a lesson learned from SARS-CoV-2 lineage system designs. The final rule on compulsory sister lineages is to ensure that each designation level provides new information that is usable for public health—i.e., not simply the same lineage circulating in the same area year after year, but a distinguishable clade that differentiates it from other geographical areas. Some manual curation was also performed on this initial designation step (see Methods); specifically, that moved some nodes closer to the present to break up some very large major lineages. To ensure that most sequences since 2000 were in a major lineage in case of continued circulation, we also artificially added some additional lineages with more generous thresholds (10 substitutions and 5 sequences) to avoid the system being out of date at the start of its implementation. We do not anticipate needing to do this in future lineage releases as we will be designating major lineages prospectively, obviating the same very large unbroken diversity observed with retrospective designation.

Combining our updates to the genotype placements and the addition of lineages, sequences can be discussed using a formal longhand and a simpler shorthand nomenclature. For example, “Dengue virus serotype 3, genotype III, lineage C.2” can be abbreviated as “DENV-3III_C.2” (read as: “dengue three-three-C-dot-two”; Fig 2). New sublineages of DENV-3III_C.2 would be given names DENV-3III_C.2.1 and DENV-3III_C.2.2 and so on.

### Applications of the new lineage system

After designing a new lineage classification system and applying it to the global DENV genomic data set, we evaluated its utility for addressing real-world public health questions. We specifically tested the lineage system based on its 2 key design principles: (1) to increase resolution with which to discuss genetic diversity; and (2) to standardize the discussion.

**Fig 3:**
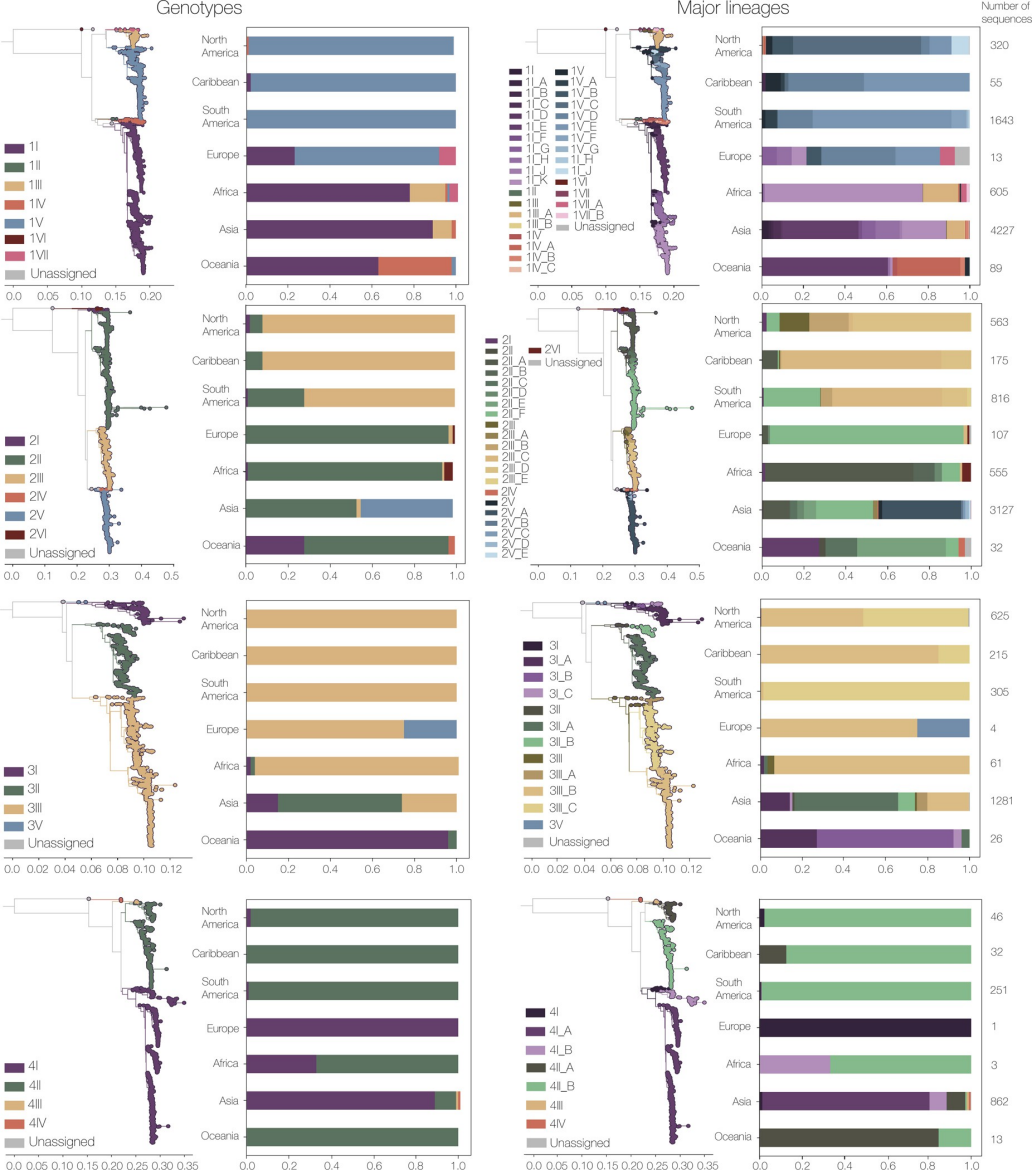
Global distributions of the new classifications of dengue virus genotypes and major lineages. Each serotype’s new genotype (first column) and major lineage (second column). Genetic distance trees are colored by genotype or major lineage, and bar charts show the percentage of whole genome sequences in each continent by classification level. Note that major lineages break down genotypic diversity further and provide additional resolution and a continent level. Numbers by each bar represent the number of sequences in each continent by serotype.

We first examined whether splitting the phylogenetic trees of each DENV serotype beyond genotypes led to increased temporal and spatial resolution. While some continents are now dominated by a single major lineage (e.g., DENV-3 and DENV-4 in South America), most continents have at least 2 major lineages designated for each serotype (Fig 3). For example, almost all DENV-1 whole genome sequences in the Americas in this data set are genotype V, but we can now split the genotype V viruses circulating in this region into 7 major lineages (Fig 3).

**Fig 4:**
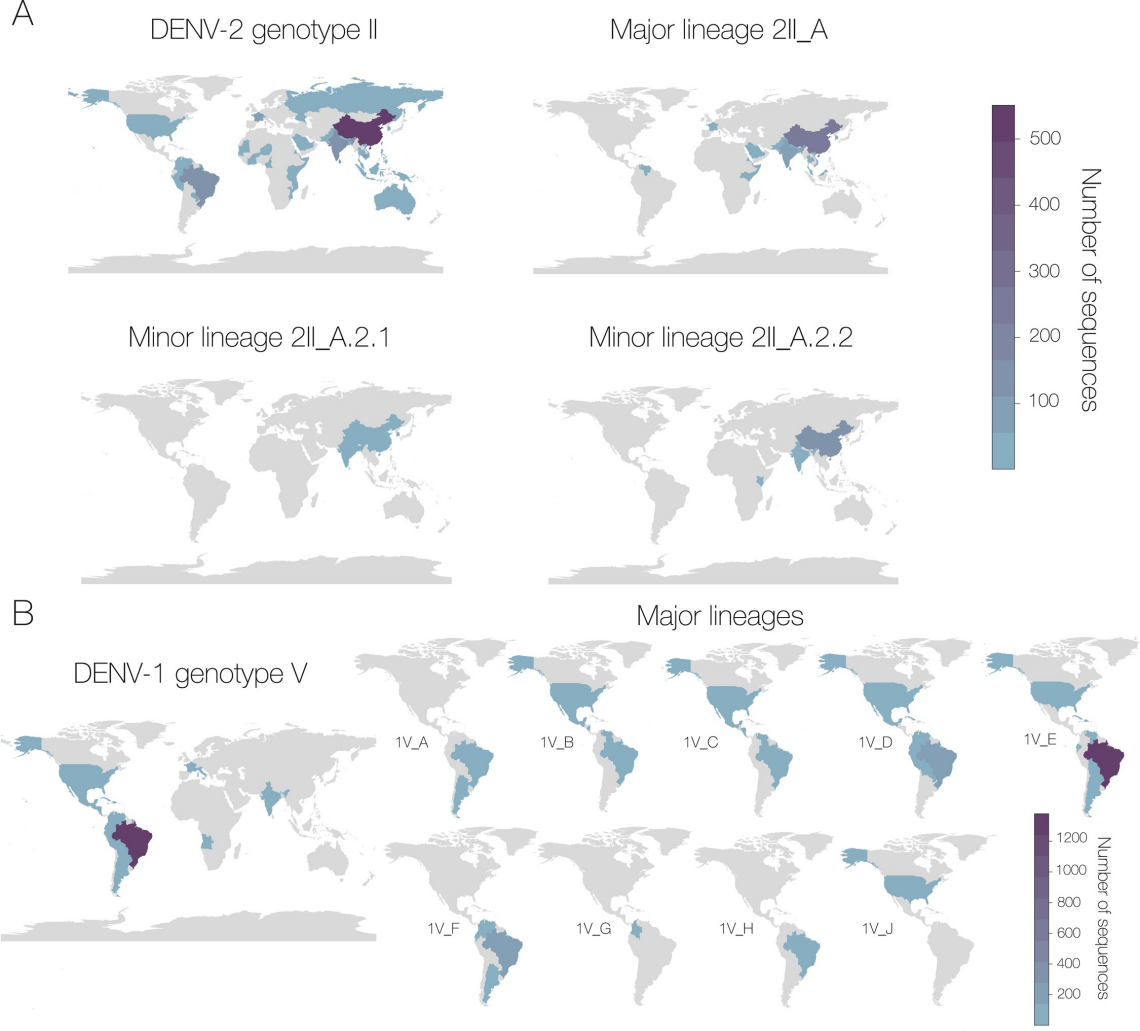
Examples of increased geographical resolution using the new classifications of dengue virus lineages. (A) Each map shows the number of sequences in the data set in each country classified as, respectively, DENV serotype 2 genotype II, serotype 2 genotype II major lineage A, and then 2 minor lineages of A.2.1 and A.2.2. The color represents the number of genome sequences from blue to purple running from low to high. (B) Map shows the distribution of the whole of DENV-1 genotype V and all of its constituent major lineages in the Americas. Major lineage 1V_A also has sequences from India, 1V_B and 1V_E have some from France, and 1V_D and 1V_D have some from Italy, although these are not shown here in the interests of space. Color represents the number of genome sequences. Base map layer downloaded from the Global Administrative Database (https://gadm.org/download_world.html).

To further explore this apparent increased resolution, we mapped the sampling location of every sequence in each level of lineage designation to identify whether geographic scope also narrows as classification level decreases. While some minor lineages are relatively widespread, we find several examples of increased geographic resolution with increased phylogenetic nesting (Fig 4A). For example, DENV-2 genotype II, also known as the Cosmopolitan genotype, has been identified in all regions where DENV circulates. When we assign sequences from this genotype to major lineages, the major lineage DENV-2II_A sampling locations occur only in the eastern hemisphere. When further exploring the minor lineages, 2II_A.2.1 and 2II_A.2.2 have been detected in south and east Asia, only 2II_A.2.2 has been detected in Africa, specifically Kenya (Fig 4A). In this scenario, if a related DENV was sequenced in East Africa, the minor lineage assignment would immediately provide clues about whether it was a potential new introduction from Asia to Africa (i.e., 2II_A.2.1) or whether it may have arisen autochthonously (see Box 1) within the region (i.e., 2II_A.2.2). In comparison, under the existing system, the hypothetical new lineage would simply be assigned to DENV-2 genotype II, which is one of the most diverse and widespread genotypes of DENV and little additional information would be gleaned without conducting phylogenetic analysis. We also show how the major lineages of DENV-1 genotype V, which dominates DENV-1 transmission in the Americas (Fig 1), provide additional geographic resolution in this region (Fig 4B). For example, DENV-1V_B is sampled in Nicaragua, the US, Mexico, Venezuela, and Guatemala; whereas DENV-1V_E is dominated by sequences sampled in Brazil. Notably, DENV-1V_G is only found in Colombia and Trinidad and Tobago, 1V_H in Brazil, and 1V_J in the US. These major lineages therefore provide information on different patterns of circulation of DENV-1 in the Americas.

In the absence of a global lineage classification system, individual research groups have labeled lineages using their own nomenclature systems to aid research and surveillance efforts. Although changing names can be challenging, it is important to have a standardized nomenclature system to aid discussion between different regions. This makes it easier to rapidly identify which lineages are the same in different countries and therefore which are spreading internationally, possibly indicating a relevant phenotypic property. For example, there has been a new introduction of DENV-3 genotype III from Asia into the Caribbean, which has since spread across the Americas and has been reintroduced into Asia and Africa [3,5,32,33]. This lineage spread may be connected to the large DENV-3 outbreaks in 2023 in the region, and so there is a risk of different research groups naming this lineage separately (e.g., “DENV-3 GIII-American-II lineage”) making it harder to detect the wider pattern of spread and phylogenetic relatedness. In the new system, this introduction has been designated a minor lineage 3III_B.3.2 (S4A Fig) and is distinct from another older Caribbean introduction, designated 3III_B.3.1 [34].

Further, our system has equivalents to many of the existing sublineages individually defined by other research groups. For example, 4 lineages of DENV-3 genotype III have been described in Brazil [10]. BR-I and BR-II fall into DENV-3III_C.2, and while the single sequence in BR-III is not in our data set as it is only an E sequence, its closest whole genome relative (GenBank accession: FJ898462) is also in 3III_C.2. Some sequences in BR-I are also assigned a sublineage, DENV-3III_C.2.2. The single sequence representing BR-IV is assigned to 3III_C.1. While three of the named Brazil lineages fall into the same major lineage in our system, it still provides a separation from the newly introduced 3III_B.2 lineage of the same genotype that we describe above (S4A Fig). Similarly, in DENV-2 genotype III, there are lineages in Brazil defined as BR1-4 [11,27,29]. In our system, BR1 is DENV2-2III_B, BR2 does not get assigned to a major lineage because it does not meet the size threshold required and so it remains part of the larger 2III, BR3 is DENV-2III_C, and BR4 is 2III_C.1.1 (S4B Fig). We note that only BR4 in DENV-2 is a 1:1 match with our lineage system and thus there may still be reason for research groups to use finer resolution of lineage classification for their specific needs. Our goal is not to restrict these activities, rather to argue for the use of common classifications for external communications. To aid this effort, we included lineage assignments for every whole genome sequence in our data set (S2 Table).

While we deliberately do not take phenotypic differences into account in the designation of lineages, it is important that they are captured by the neutral designation process. For example, in a study of DENV-2 in Nicaragua, the authors describe 3 sublineages which underwent lineage turnover from NI-1 to NI-2A and then to NI-2B [9]. They also describe an apparent increase in relative fitness of NI-2B compared to NI-1. We compared these lineages to our standardized nomenclature and found that NI-1 corresponds mostly to DENV-2III_D.1.3 (and some to 2III_D.1), NI-2A to 2III_D.1, and NI-2B to 2III_D.1.1 (S4B Fig). We therefore capture the lineage replacement and the apparent phenotypic difference between NI-1 and NI-2B (going from 2III_D.1.3/2III_D.1 to 2III_D.1.1). Further, 2III_D.1.1 (NI-2B), while mostly sampled in Nicaragua, has a subclade (see Box 1) which was sampled in Cuba and Costa Rica from 2019 to 2022 (circled in S4B Fig), highlighting the importance of a standardized naming system between countries. A more recent paper [28] also described NI-3 sublineages, which correspond to 2III_D.1.2 in our system.

In order to explore potential phenotypic differences between lineages in a more systematic way, we used antigenic distance data (see Box 1) generated from different serotypes and genotypes in Thailand over a 20-year period [35]. We began by comparing within-classification pairwise antigenic distances by serotype (S5A Fig) and found that while there was a slight decrease between serotype, genotype, and major lineage, it was not significant or consistent. Indeed, DENV-1 had a gradual decrease in antigenic distance across all 3 levels, DENV-2 only decreased at the major lineage level, DENV-3 mostly decreased at the genotype level, and DENV-4 had no noticeable difference between classification levels. We then mapped the antigenic distances in 3D space (S5B and S5C Fig) and found that major lineages did not form distinct clusters. These results, however, may be complicated by not having a broad representation of the global major lineages. Therefore, we may expect to find more noticeable differences in pairwise antigenic distances among the serotype, genotype, and major lineage levels if this antigenic data set also included more diverse viruses (e.g., lineages from the Americas). Our results could also indicate that antigenic distance is more complex than a simple phylogenetically clustered trait. Indeed, the original authors found that antigenic distance varied more over time than between other circulating clades [35]. Regardless, our lineage system still provides a way to identify distinct lineages that may have an epidemiological advantage, which will be essential for evaluating the impact of antigenic distance on the immune landscape and therefore real-world effectiveness of new dengue vaccines.

By capturing known phenotypic diversity, providing additional geographic resolution, and standardizing discussion of important lineages, the lineage system proposed here builds on the success of the currently used genotypes and provides a necessary tool for monitoring DENV as it continues to spread worldwide.

### System stability and assignment validation

After designing this system and showing that it is useful to describe existing genomic diversity and to standardize discussions of DENV evolution at a higher resolution than before, we performed a series of validation checks. A key element of any lineage system is that it is reliable with regards to the sampling structure of the data set and genome coverage. When testing both, we found that the proposed rules generate stable lineages with low coverage genomes and with different subsamples.

It can be challenging to obtain high coverage DENV genomes as viral load tends to decrease rapidly after the short viremic phase that often occurs prior to sample collection [42]. Further, while capacity for whole genome sequencing is increasing globally, mostly due to the genomic capacity accrued by public health and research institutes during the SARS-CoV-2 pandemic, many groups will preferentially sequence only the E coding region as this is all that is required for genotyping, and it is faster than whole genome sequencing. We therefore simulated low coverage genomes from a subset of the data set (*n* = 309) by replacing nucleotides with N’s in runs of 200 to mimic amplicon drop-outs (see Box) at different percentages from 90% to 10% in 10% intervals, then 5% and 1%, and re-assigned them using the Genome Detective typing tool (see Box 2). The overall assignment accuracy for serotypes, genotypes, and major and minor lineages was high, even with very low genome coverage (S6A Fig). We found major lineage assignments to be 97% accurate at 5% genome coverage, and it drops to 87% accuracy at 1% genome coverage. For minor lineages, assignment accuracy is 93% and 76% at 5% and 1% genome coverage, respectively. Therefore, we recommend using DENV virus genome coverages at 5% and higher for accurate lineage assignment.

**Box 2. Lineage assignment tools**

The previous sections of this paper discussed the process of lineage designation—the development of a standardized new lineages classification system based on expert opinion. In addition, there is assignation, the process of providing a lineage call to a new sequence, which should be possible to do easily by research groups and public health professionals globally. Here, we provide options for lineage assignment using Genome Detective, GLUE, and NextClade. In the supplementary information, we also provide alignments and phylogenies of representative sequences from each of the lineages so that the reader may also try their own methods.

The Genome Detective Platform [23] is a microbial bioinformatics software suite that includes a generic framework for phylogenetic subtyping tools, allowing the creation of subtyping tools for any virus species. Currently, Genome Detective includes subtyping tools for 19 virus species, developed with subject matter experts globally [22,36–41]. A DENV subtyping tool was first developed in 2019 [22], and since version 4.0 it was updated to use the lineage designation scheme introduced in this work (https://www.genomedetective.com/app/typingtool/dengue/, see Methods and S8–S10 Figs).

The GLUE framework is an open software environment for managing and analyzing virus sequence data [42]. It facilitates the development of “projects” that contain data and algorithms necessary for conducting comparative genomics investigations of specific viruses. GLUE incorporates a protocol for rapid phylogeny-based genotyping, maximum likelihood clade assignment (MLCA). Given a reference phylogeny and its corresponding alignment, MLCA uses maximum likelihood to efficiently estimate the optimal placement of a query sequence within the phylogeny. This approach has been used to implement subtyping tools for various viruses, including hepatitis C virus, rabies virus, and bluetongue virus [42–44]. A GLUE project focused on DENV (Dengue-GLUE) is available for local installation and incorporates an MLCA-based subtyping procedure utilizing the lineage designation scheme introduced here (https://github.com/giffordlabcvr/Dengue-GLUE).

Nextclade is a robust pathogen classification tool designed to assign genome sequences to clades or variants by aligning them with a reference sequence (Aksamentov et al. 2021). This tool is available both as a command-line utility and as a web application, making it highly effective for managing large sequence data sets and facilitating integration into bioinformatics protocols related to genomic surveillance. The build for this dengue classification system can be found here: https://clades.nextstrain.org/.

We further tested the tool and the system by trimming whole genome sequences keeping only the E coding region and again assigned them using Genome Detective. Serotypes, major lineages, and minor lineages were all correctly assigned using E sequences only. Seven out of 309 had incorrect genotype assignments, which were all related to but not a part of DENV2 genotype I. This is therefore an accuracy of 98% for genotypes and 100% for other classification levels using only complete E sequences.

We also tested the impact of sampling artifacts on lineage designation by randomly subsampling the data set 10 times and rebuilding the trees to see if the same monophyletic clades emerged. We found that they generally did (S6B Fig), with DENV-1 having the highest mean of different clades (0.01, 95% CI: 0.0 to 0.034), and DENV-2 having no different clades in any of the subsamples. DENV-3 and -4 were in between with 0.004 (95% CI: 0.0 to 0.017) and 0.006 (95% CI: 0.0 to 0.023), respectively.

Each level of classification can therefore be reliably assigned using a freely available, user-friendly tool even with only E sequences and with very low coverage genomes. Our lineage system is also robust to the sampling bias inherent in almost any genomic data set. This system is therefore robust and reliable for real DENV genomic data sets.

### Case studies with additional sequencing data

To ensure that this system is applicable to new data at an appropriate resolution, we tested it using data not included in the original designation process. We used whole genome sequences from Vietnam and Brazil to check that the system works in different global regions, and E sequences from Tanzania to ensure that it works with partial genomes.

**Fig 5:**
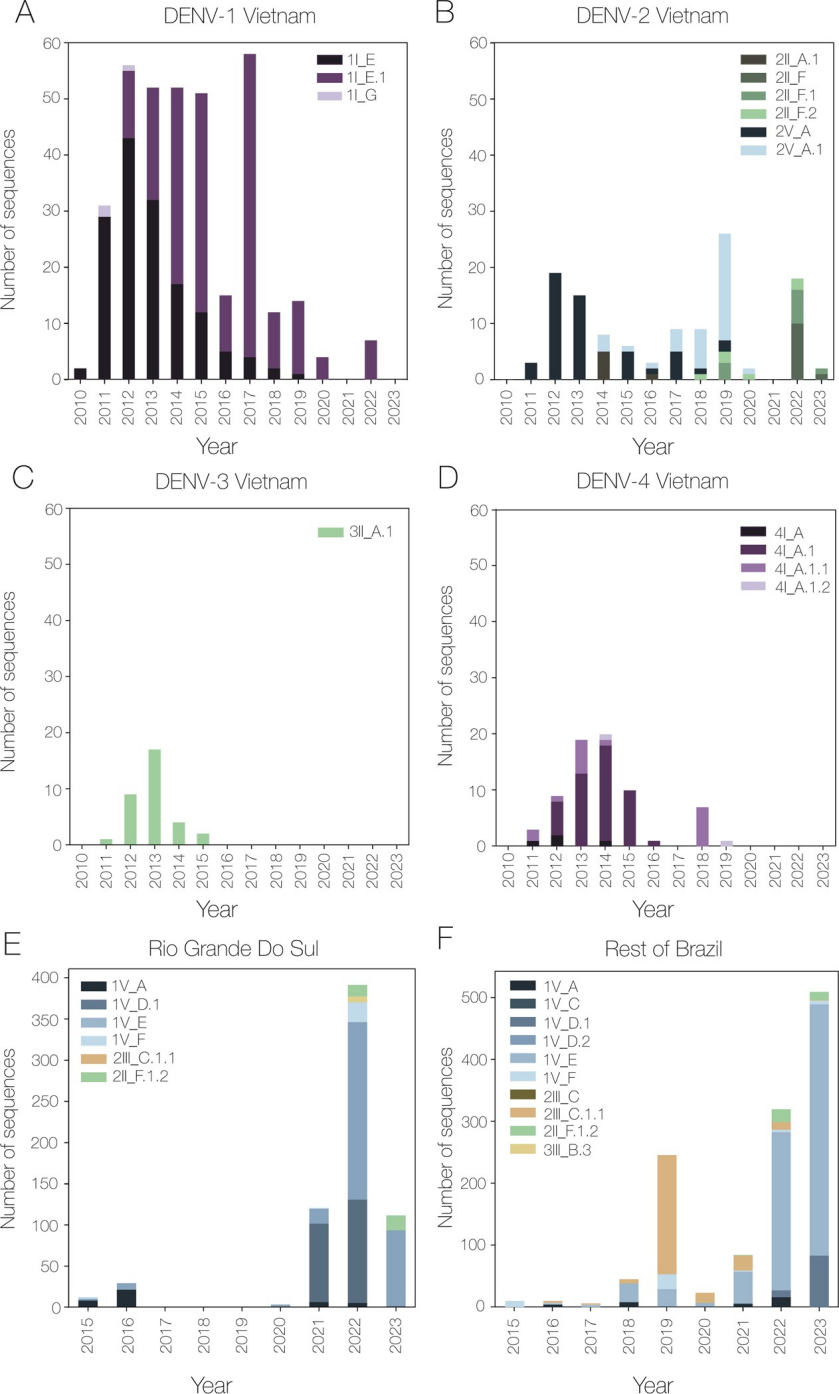
Number of dengue virus whole genome sequences mostly from Ho Chi Minh City, Vietnam, assigned to each lineage over time. Bar graphs shown for (A) DENV-1, (B) DENV-2, (C) DENV-3, and (D) DENV-4. (E) Time series of whole genome sequences from Rio Grande do Sul, Brazil, by year. (F) Lineage assignments of whole genome sequences from the rest of Brazil in this data set (non-case study sequences from Rio Grande Do Sul have been removed).

Sequences from every country were successfully assigned a major lineage, and many also had minor lineages (S7 Fig). In Vietnam, there was mostly a single genotype in each of the 4 serotypes that accumulated higher resolution as time went on—this suggests that transmission was driven by continued circulation of the same lineages rather than reintroduction (Fig 5A–5D). In the south of Brazil, we found the opposite: lineage frequency dynamics were similar to the rest of the country with a slight lag, suggesting importations from elsewhere in Brazil (Fig 5E and 5F). Despite our training data set containing fewer sequences from Africa compared to Asia and the Americas, sequences from Tanzania were successfully assigned. The lineages identified were mostly similar to those found in Asia, especially China. Few minor lineages were assigned, however, suggesting that while major lineages can be successfully assigned with partial sequences, there may be some resolution missing compared to using whole genomes. Full case study reports can be found in the supplementary text. We note that the Brazil and Vietnam sequences have now been included in the designation set, and additional lineages have been designated based on them.

### Limitations and future sustainability

There are limitations to the system we have proposed here. First, there is likely unrecognized diversity due to global inequity in sequencing capacity, especially in Africa (S11A Fig). This unrecognized diversity also limits the conclusions we can draw with lineage assignments, as similar circulating lineages in under-sampled regions may not be epidemiologically connected to one other, and may be independent introductions. This is compounded by our decision to use whole genomes to build the system and not explicitly include E coding region sequences, which tend to be older. Our lineages are therefore influenced by the number of whole genome sequences available (S11B Fig), and indeed the number of lineages is strongly correlated with the number of sequences on the country level (*p* < 0.001, S11C Fig). We hope that as sequencing capacity continues to build and expand globally, this disparity will decrease, while still being able to define new lineages when new diversity is captured.

On a local level, our system does not always provide enough resolution for specific epidemiological questions—some minor lineages are still relatively widespread. This is due to trying to have fewer lineages at the start of the system implementation to ease discussion, and thereby help with uptake. We also note that the new system is not a 1:1 relationship with existing regionally defined lineages. This is partially because, in some cases, previous lineages were defined based on a single sequence or a small group of sequences, which does not meet our criteria for lineage designation, but may be helpful on a regional level for defining introductions. Therefore, this system does not replace local-level phylogenetic analysis, and simply provides a first, quick pass at describing the diversity in a data set.

Any lineage system must be easy to maintain and have an intrinsic stability, especially as the DENV genomic data set continues to grow. We deliberately chose rules to define lineages which are computer-readable (and indeed initially designated them using custom scripts) to reduce the person-time required to generate new lineages.

We envision 2 main ways that new lineages could be suggested going forward. First, individual research teams can submit a github issue to a public github repository (information at https://dengue-lineages.org/) using a standard form. If accepted, these suggestions will be given a “putative” designation and the label claimed until they have been reviewed so that the study can proceed with relevant lineage information. These putative lineages will then be formally incorporated into the nomenclature system during an annual review process. The second method of new lineage designation will be during this annual review, where proposals for previously undesignated diversity would be generated using automated scripts. Alternatively, these proposals could be generated with an automated lineage designation tool such as recently described [43]. We did not use this tool here because our initial designation required some manual curation and integration into the current serotype/genotype system, but this will not be required in the future and so this tool may be useful. We hope that these 2 methods of lineage proposal balance the need for up-to-date information for those conducting surveillance, without requiring a large amount of effort to maintain.

These new lineage proposals would then be submitted to a designated international committee for decision-making. At this stage, the lineage designation rules and thresholds would also be reviewed. We note in particular the phylogenetic distance threshold, which we selected in the interests of stability and keeping the number of lineages manageable. However, part of the reason these long branches exist is the relative undersampling of DENV in some geographic regions. In an ideal world with more routine DENV sequencing, there would be fewer very long branches in the phylogeny, and we hope this may be the case going forwards. We would not, however, change thresholds for existing lineages so as not to change results of already completed work, or change the defining nodes of existing lineages or genotypes to ensure stability.

Finally, in the interests of usability and fitting better into existing work, we provided assignment tools already used for subtyping DENV and other viruses—Genome Detective, GLUE, and NextClade. We note that our system is open and we are supportive of the development of any other tools to assign sequences to these lineages. We believe this will also help with sustainability, as opposed to designing a new tool specifically for this lineage system which would also require maintenance.

## Discussion

DENV is a globally important virus that causes high levels of morbidity each year. With increasing globalization and climate change, viruses can traverse continents with ease, leading to increased global genetic mixing. As worldwide genomic sequencing capacity increases, genomic epidemiology becomes a more powerful tool for understanding how DENV spreads and causes outbreaks. The recent influx of genomes must be categorized beyond existing genotypes to provide rapid and understandable epidemiological conclusions.

Here, we present a lineage system, drawing inspiration from the design and uses of similar systems implemented for SARS-CoV-2 [12], rabies [44], and mpox viruses [45]. We propose major and minor lineages in addition to slightly adjusting the existing genotypes to capture current diversity. We ensure that these lineages are mostly stable despite phylogenetic uncertainty and low coverage genomes by having a high inferred substitution threshold for defining a lineage.

Our system assigns E-only sequences at least to major lineages and in some cases to minor lineages. This is important as many previous studies only include E sequences, and there are still approximately double the number of E sequences as whole genomes on GenBank. We still encourage the sequencing of whole genomes going forward as they contain more genetic information for phylogenetic placement and analyses, as well as requiring a shorter time period of sampling to accurately estimate rates of evolution [46]. Further, natural selection acts on the whole genome and so losing information on how the nonstructural proteins evolve may hamper efforts to design and monitor the effectiveness of potential antivirals and vaccines. For these reasons, while a lot of important conclusions can and have been obtained with E sequencing, we encourage, where possible, for the whole genome sequencing of DENV.

Our proposed lineage system provides additional resolution for the discussion of potentially important global DENV diversity and provides a conceptual framework that could be extended to incorporate hierarchical lineage classifications for other arboviruses with broadly defined genotypes (e.g., chikungunya and West Nile viruses). As DENV continues to circulate, the volume of genomic data increases, and new interventions are rolled out that may lead to important viral adaptation, this will become an imperative. By creating stable lineages that work well in different dengue-endemic regions, our system has the potential to enhance DENV genomic surveillance and epidemiology across the globe (https://dengue-lineages.org/).

## Methods

### Ethics statement

The Institutional Review Boards (IRB) from the Yale University Human Research Protection Program determined that pathogen genomic sequencing of de-identified remnant diagnostic samples as conducted in this study is not research involving human subjects (Yale IRB Protocol ID: 2000033281).

### Data set generation

We downloaded all DENV sequences with more than 70% of the genome covered and a year of collection listed released on GenBank until the 28 July 2023 and released on GISAID between 1 January 2022 and 28 July 2023. We matched sequences between these 2 databases to remove duplicates to create a near-complete publicly available full whole genome data set (DENV-1 = 5,657, DENV-2 = 4,106, DENV-3 = 2,166, DENV-4 = 1,045). We then aligned all of the sequences by serotype using MAFFT v.7.490 [47] and manually curated it in Geneious v.2022.1.1, including trimming the untranslated regions (UTRs). We removed sequences which were extremely divergent and those with frame-breaking insertions (DENV-1 = 2, DENV-2 = 1, DENV-3 = 1). We later updated this data set with additional GenBank sequences up until the 8 July 2024 to keep it as up to date as possible during the review process.

We then inferred first pass maximum likelihood trees by serotype using IQTree v2.1.4 [48]. We used the program’s model selector on the smallest data set (DENV-4) to gauge the best nucleotide substitution model to use, and it returned a transition model with empirical base frequencies and a free rate model with 6 categories (TIM +F+R6). We then rooted this tree using a molecular clock assumption in TempEST [49] and a heuristic residual mean squared model. We used the root-to-tip plot produced by this rooted tree to prune molecular clock outliers, a reliable indicator of quality control issues [50]. Most of the outliers that we found here were resequencing of commonly used virus stocks.

The final data set sizes used for developing the lineage system were DENV-1 = 6,975, DENV-2 = 5,390, DENV-3 = 2,519, and DENV-4 = 1,215. We assigned each of these sequences to existing genotypes using Genome Detective Dengue Virus Typing tool [22].

### Lineage system design

Our aims in the design of this system were to break up large clades in the genotypes to provide sufficient resolution to capture epidemiologically relevant patterns; but, drawing on our experience from SARS-CoV-2 nomenclature, not to have many fine-scaled lineages which are hard to discuss, and require regular updating.

With the above datasets, we inferred a new maximum likelihood tree for each serotype separately using the TIM+F+R6 nucleotide substitution model as suggested by IQTree’s model finder.

We separated clades using custom preorder tree traversal scripts using the criteria of (**1**) 15 sequences; (**2**) a branch length of 25 substitutions for major lineages and 20 for minor lineages; and (**3**) presence of a sister lineage. Branch length was calculated by multiplying the IQTree divergence length in substitutions by the relevant alignment length. The length and size thresholds were obtained by testing different combinations to obtain an optimal level of resolution where major clades were split up, but ideally no more than 2 sublevels of minor lineages were present. Major and minor lineages were assigned using the same rules and at the same time but were given different nomenclature.

At this point, we performed a second designation step to ensure that as many potentially epidemiologically significant lineages as possible have been captured by at least a major lineage. The aim here was to avoid a situation where a lineage, possibly from a country or region which is undersampled, could cause an outbreak in the near future without having any assignment beyond a genotype. To do so, we found all clusters containing only sequences sampled after 2000 without a major lineage designation, and designated further major lineages using a more generous threshold of a minimum size of 5 sequences and a minimum branch length of 10 substitutions. Any clusters that did not meet these criteria, even those with sequences after 2000, were left unassigned, as they would not be very stable between tree building iterations if they did not meet the substitution threshold.

Finally, we performed some manual curation. As the current data set sizes between serotypes vary by a factor of 5, rules which work well for DENV-3 and DENV-4 can lead to a high level of nesting in DENV-1 and DENV-2. For DENV-1, we moved the defining node for 1V_B closer to the present to enable us to maintain the strict hierarchy of the lineages and add up to major lineage G to break up the diversity. We also did this for 1I_A and added up lineages up to K, 1VI_A to add a sister lineage B (this was a lineage generated by the second designation step where sister lineages were not mandatory), 2II_B to add lineages up to G, and 2III_B to add lineages C and D. It is worth noting that for all of the manual changes we made, a lineage never has fewer than 10 substitutions along the branch before it.

All tree visualizations were generated using baltic (https://github.com/evogytis/baltic).

### Generating representative trees

For the assignment tools, it was necessary to make representative trees and alignments of each of the levels of designation. To do this, we used the phylogenetic distance matrix, calculated using the python package DendroPy [51], which gives pairwise phylogenetic distance between each pair of sequences. We took 5 sequences with coverage of over 90% which were furthest apart from each other in each of the genotypes, major lineages, and minor lineages.

It was also important to ensure that the most basal sequence of each clade was present; otherwise, other basal sequences may be under-assigned. We did this first by checking if the lineage-defining node had an immediate descendent node which was a tip, and this node was added to the representative data set. If there was not a node which was a tip, the sequence in the lineage with the lowest distance to the root (i.e., fewest SNPs) was included.

Alignments were generated, and trees were obtained by pruning them from the larger trees using jclusterfunk (https://github.com/snake-flu/jclusterfunk).

### Genome detective

The subtyping tool for genome detective (https://www.genomedetective.com/app/typingtool/dengue/) takes a fasta file or sequences as a text input (S8 Fig**)** and has 2 main steps to assign a lineage to a sequence.

The first step is species identification, which for the DENV subtyping tool is to identify the serotype. Basic Local Alignment Search Tool (BLAST) N and BLASTX are used to identify a maximum of 3 potential hits. For each of these potential hits, we then use the Advanced Genome Aligner (AGA) [52] to align the query against the reference and compute the overlap and concordance score. Finally, we select the reference with the highest product of overlap and concordance score. If this species corresponds to the species for which the tool was developed, we proceed to the clade identification. In practice, the species identification step will sometimes identify at a taxonomic level deeper than species, if there is a reference sequence in NCBI RefSeq for this deeper level. In particular, for the DENV subtyping tool, this first step will identify the serotype.

Once the serotype has been identified, maximum likelihood phylogenies containing the query sequence and representative sequences for each lineage are constructed to identify the clade that is most likely to contain the query. The most recent dengue subtyping tool uses IQ-TREE 2 for its analysis, a change from previous versions which used PAUP* [53]. This is because the former is not a pure hill-climbing algorithm and so is less likely to get stuck in a local optimum. Every clade we wish to identify is defined by approximately 5 representative sequences (see above). We compare the bootstrap value of the node containing some or all of the representative sequences and the query, and identify the most likely cluster. Then, we compare the support values for nodes containing only the representative sequences (outer support) against the representative group plus the query (inner support). This allows us to estimate how likely it is that the query sequence falls inside the cluster. If the bootstrap support for the most likely cluster is lower than 50%, or the most likely cluster is the outgroup, the sequence cannot be assigned. If the bootstrap support for the most likely cluster is higher than 50%, but the inner support is not significantly higher than the outer support, the sequence is assigned as “related to, but not part of” the cluster. If the bootstrap support is higher than 50%, and the inner support is significantly higher than the outer support, the sequence is assigned to the most likely cluster.

Hierarchical clade identification is a stepwise process. Once a clade is identified, the process will reiterate to identify the subclade, if any subclades exist. In other words, once the genotype (e.g., 3II) has been identified, the tool will proceed to try assigning the major lineage (e.g., 3II_A), and once this has been assigned, it will continue to the minor lineage (e.g., 3II_A.1), and then to further minor lineages (e.g., 3 II_A.1.2). Unlike previously developed subtyping tools, which could only identify clade and subclade, the current DENV tool can support an arbitrary amount of assignment levels. This is necessary to enable an evolving lineage system particularly useful when deeper levels of minor lineage are possible. Outputs are a live-updating page with lineage assignments (S9 Fig), and on clicking the sequence ID, details of the phylogenetic analysis which lead to the lineage assignment (S10 Fig).

### GLUE

The GLUE framework is an open software environment for the management and analysis of sequence data [42]. GLUE implements a protocol for rapid phylogeny-based genotyping called “maximum likelihood clade assignment” (MLCA). The MLCA method uses maximum likelihood (ML) to optimally place a query sequence within a previously generated reference phylogeny, and then classifies the sequence based on its positioning relative to reference sequences. Although MLCA computes a clade assignment for each query sequence individually, it can also be applied to batches of sequences.

The arguments passed to MLCA are (1) the query sequence; (2) a reference phylogeny containing at least 1 reference sequence for each clade category; and (3) the multiple sequence alignment used to construct the phylogeny. The algorithm first uses the MAFFT program [49] to add the query sequence to the alignment. The extended alignment and the reference phylogeny are then passed as arguments to the RAxML program’s Evolutionary Placement Algorithm (EPA) [56], which suggests the optimal placement of a query sequence within a fixed reference tree. Finally, the output phylogeny from RAxML is parsed by the GLUE engine, and a classification is assigned based on topological relationships and evolutionary distances relative to reference sequences.

A GLUE project for DENV (Dengue-GLUE) was constructed, and this project was used to implement an MLCA-based genotyping procedure for DENV. We constructed an ML reference phylogeny for each dengue serotype using the reference sequences established in this study. These phylogenies, along with their corresponding clade categories, were then integrated into the Dengue-GLUE framework. When a query sequence is submitted to Dengue-GLUE’s genotyping system, it is initially assigned to a DENV serotype using BLAST [54]. Subsequently, the sequence is classified via MLCA, based on the reference phylogeny for that particular serotype.

### NextClade

Nextclade assignments are based on the substitutions that define lineages. As this system was not explicitly based on specific substitutions, nucleotide and amino acid substitution calls were performed for all branches of the representative trees using Augur Ancestral and Augur Translate tools, respectively [55]. The substitutions were relative to a reference sequence for each serotype, downloaded from GenBank: NC_001477.1 (DENV-1), NC_001474.2 (DENV-2), NC_001475.2 (DENV-3), and NC_002640.1 (DENV-4). Substitutions were only called on the polyprotein, and so the ends of the reference sequences were masked.

The identification of substitutions associated with previously classified branches was done manually to ensure accurate substitution association with the developed classification. Unique substitutions were prioritized due to their ability to differentiate clades. However, in the absence of or presence of only a single unique substitution, homoplasies were also considered.

A substitution table, adhering to the standards set by the Augur Clades tool, was generated. The annotated phylogenetic tree was exported using Augur Export v2 and combined with quality parameters, alignment, and reference files for the construction of the Nextclade v3 data set.

### Case studies

Clinical specimens from Vietnam were sampled mostly from Southern Vietnam between 2010 and 2023. They were sequenced at the Yale School of Public Health using the recently developed amplicon sequencing DengueSeq protocol [19]. Libraries were prepared using the Illumina COVIDSeq test (RUO version) with the pan-serotype primer pools, and sequenced on the Illumina NovaSeq 6000 or X Plus (paired-end 150) at the Yale Center for Genome Analysis. Consensus sequences were generated by mapping reads to dengue reference genomes through the DengueSeq bioinformatics pipeline using default settings (minimum frequency threshold of 0.75 and minimum depth of 10 to call consensus).

Methods for generating sequences from Brazil can be found here [56] and from Tanzania can be found here [57].

To assign lineages to each of the case study data sets, we used the representative sequences described above. We always split up serotypes for each analysis. We aligned new data with representative sequences using MAFFT v7.490, and then built a maximum likelihood tree using IQTree using the representative tree as a constraints tree and the TIM+F+R6 substitution model, as above. We rooted trees using the same roots as we inferred using the molecular clock assumption as above.

Finally, we annotated the resultant rooted trees using custom scripts to find the lineage-defining nodes. Lineages were assigned to new sequences based on them being descendants of these lineage-defining nodes.

### Validation tests

For the completeness analysis, we took a subset of the full data set (*n* = 309) evenly sampled from different lineages and serotypes. We replaced bases with Ns in runs of 200 to replicate amplicon drop-outs. We therefore tested genomes which we artificially lowered from 10% to 90% coverage in 10% intervals, and 5% and 1%. We also artificially cropped the whole genome sequences to only the E coding region. We assigned these sequences using the genome detective dengue subtyping tool.

For the stability analysis, we built trees with 10 different subsamples of the data, and assessed whether the same lineages would be designated using the algorithm developed here using the same custom python scripts.

### Connecting new lineages to currently used lineages

For DENV-2 lineages from Brazil denoted BR1-4 [27,29] and NI-3 from Nicaragua [28], we were able to use tip labels on tree figures in the papers to compare to assigned sequences in the new system. For NI-1 and NI-2 lineages, sampled in Nicaragua [9], we used lineage defining amino acid substitutions (Table 2 in [9]). We took the sequences from the paper, identified substitutions at the relevant position, and put them in sets based on having the same amino acid substitution in common.

### Data availability

All sequences used to design the lineage system are from Genbank and GISAID, accession numbers in S2 Table. Custom scripts and alignments of representative sequences from Genbank can be found on our github (https://github.com/DENV-lineages/lineages-paper).

Sequences for the Vietnam case study can be found on Genbank under accession numbers PP269455-PP270050, in bioproject PRJNA1072696. For the case study from Tanzania, sequences were drawn from [57], and can be found on Genbank under accession numbers OM920035-OM920066 for DENV-3 and OM920075-OM920415 for DENV-1. Sequences for the Brazil case study can be found on GISAID under accession numbers EPI_ISL_17733558 ‐ EPI_ISL_191469691.

## Supporting information

S1 Table Roman numeral equivalents for geographical names of DENV-2 genotypes. (PDF)

S2 Table Genbank and GISAID accession numbers of sequences used with assignments. (CSV)

S3 Table Information on each of the major and minor lineages. (CSV)

S4 Table GISAID acknowledgements table. (PDF)

S1 Fig Pairwise genetic distance of new genotypes. (A) Distribution of pairwise genetic distance within each genotype, colored by serotype. (B) Regression of the number of sequences in each genotype compared to the average pairwise genetic distance.
(PDF)

S2 Fig New lineage system for serotypes 1, 2, and 4. Each row is a serotype and each column, respectively, is new genotype, major lineage and minor lineage. Note that there are many more minor lineages for serotypes 1 and 2, as they have much larger data sets currently compared to serotype 4. Serotype 3 shown in Fig 2.
(PDF)

S3 Fig Schematic displaying assigning new lineages. All 4 putative lineages displayed in the top 2 trees are valid lineages as they meet all 3 criteria of branch length, clade size, and having a sister lineage at the same distance from the root in terms of node number. Invalid lineages are shown along the bottom, with the focal putative lineage shown in purple.
(PDF)

S4 Fig Comparison of previously used sublineages to proposed system. (A) Maximum likelihood tree of DENV-3 genotype III, colored by new lineage designation, with lineages BRI-IV and novel Caribbean introduction indicated. (B) Maximum likelihood tree of DENV-2 genotype III, colored by new lineage designation, with lineages BR1-4 and NI-1 to NI-3 indicated. The circled clade indicates recent circulation of NI-2B/2III_D.1.1, which is suggested to have a transmission advantage [9], in Cuba and Puerto Rico.
(PDF)

S5 Fig Antigenic distance at different levels of classification. (A) Distribution of antigenic distance in each level of classification, with serotype in green, genotype in purple and major lineage in yellow. Minor lineage is excluded due to a lack of antigenic data across minor lineages. (B) 3D map of sequences in antigenic space by serotype, colored by genotype. (C) 3D map of sequences in antigenic space by serotype, colored by major lineage
(PDF)

S6 Fig Validation. (A) Average correctness of genome detective assignments at different classification levels using artificially downsampled sequences across 5 replicates. Each line corresponds to a different classification level. Error bar is not visible. (B) Assessment of clade stability compared to different subsamples of the sequence data set.
(PDF)

S7 Fig Phylogenies of lineage assignments for case studies. (A) DENV-1-4 whole genome sequences from Vietnam, time series 2010–2023. (B) DENV-1 and DENV-2 whole genome sequences from Brazil, time series from 2015–2023. (C) DENV-1 and DENV-3 E sequences from Tanzania.
(PDF)

S8 Fig Genome Detective dengue subtyping tool starting page. The user can either upload a fasta file, or manually add sequences in the text field.
(PDF)

S9 Fig Genome Detective dengue subtyping tool results overview. The results overview shows a short summary of the different assignments.
(PDF)

S10 Fig Genome Detective dengue subtyping tool phylogenetic analysis details. An example of the analysis details for a major lineage.
(PDF)

S11 Fig Sampling distribution of DENV whole genome sequences. (A) Sampling location of whole genome sequences by country. (B) Number of lineages sampled in each country. (C) Linear regression of the number of whole genome sequences against the number of lineages in each country (*p* < 0.001). Base map layer downloaded from the Global Administrative Database (https://gadm.org/download_world.html).
(PDF)

S12 Fig Case study 1: Temporal dengue virus lineage distributions from Vietnam. Number of dengue virus whole genome sequences mostly from Ho Chi Minh City, Vietnam assigned to each lineage over time for (A) DENV-1, (B) DENV-2, (C) DENV-3, and (D) DENV-4.
(PDF)

S13 Fig Case study 2: Temporal dengue virus lineage distributions from Brazil. (A) Time series of whole genome sequences from Rio Grande do Sul, Brazil by year. (B) Lineage assignments of whole genome sequences from the rest of Brazil in this dataset (non-case study sequences from Rio Grande Do Sul have been removed).
(PDF)

S14 Fig Case study 3: Geographical dengue virus lineage distributions assigned to sequences from Tanzania. (A) Major lineage 1III_A which all DENV-1 sequences in these data sets are assigned to. (B) Major lineage 3III_B which all DENV-3 sequences are assigned to. All maps include all sublineages of each lineage, and colors show the number of whole genome sequences in the training dataset which are in each country or territory. Base map layer downloaded from the Global Administrative Database (https://gadm.org/download_world.html).
(PDF)

## Abbreviations

AGA: Advanced Genome Aligner
BLAST: Basic Local Alignment Search Tool
DENV: dengue virus
EPA: Evolutionary Placement Algorithm
MLCA: maximum likelihood clade assignment
UTR: untranslated region
