# Supplementary material for: A new lineage nomenclature to aid genomic surveillance of dengue virus
Source: PLoS Biol. 2024 Sep 16;22(9):e3002834. doi: 10.1371/journal.pbio.3002834 (PMC11426435; doi:10.1371/journal.pbio.3002834)
Supplement: S1 Table — (PDF) [file pbio.3002834.s001.pdf]

Geographical name of genotype	Roman numeral of genotype
American	I
Cosmopolitan	II
Southern Asian-American	III
Asian II	IV
Asian I	V
Sylvatic	VI
