# Supplementary material for: A new lineage nomenclature to aid genomic surveillance of dengue virus
Source: PLoS Biol. 2024 Sep 16;22(9):e3002834. doi: 10.1371/journal.pbio.3002834 (PMC11426435; doi:10.1371/journal.pbio.3002834)
Supplement: S4 Table — (PDF) [file pbio.3002834.s004.pdf]

We gratefully acknowledge the following Authors from the Originating laboratories responsible for obtaining the specimens, as well as the Submitting laboratories where the genome data were generated and shared via GISAID, on which this research is based.

All Submitters of data may be contacted directly via www.gisaid.org

Authors are sorted alphabetically.

Accession ID	Originating Laboratory	Submitting Laboratory	Authors
EPI_ISL_10238963, EPI_ISL_10239118, EPI_ISL_10239375	Centers for Disease Control and Prevention	Centers for Disease Control and Prevention	Santiago,G.A., Flores,B., Gonzalez-Morales,G., Charriez,K.N., Marcelo-Nique,A., Garcia-Mendoza,M.P. and Munoz-Jordan,J.L.
EPI_ISL_10331061, EPI_ISL_10331624, EPI_ISL_10331626, EPI_ISL_10331627, EPI_ISL_10331628, EPI_ISL_10331629, EPI_ISL_10331630, EPI_ISL_10333565, EPI_ISL_10333566, EPI_ISL_10334285, EPI_ISL_10334633, EPI_ISL_10334634, EPI_ISL_10334635, EPI_ISL_10334636			
see above	Centers for Disease Control and Prevention	Centers for Disease Control and Prevention	Santiago,G.A., Gonzalez-Morales,G., Charriez,K., Flores,B., Stanek,D., Morrison,A. and Munoz-Jordan,J.L.
EPI_ISL_10656152, EPI_ISL_10656413	French National Center of Arbovirus - Institut de recherche biomédicale des armées (IRBA)	French National Center of Arbovirus - Institut de recherche biomédicale des armées (IRBA)	Leparc-Goffart,I., Grard,G., Durand,G.A., Piorkowski,G., Noel,H. and de Lamballerie,X.
EPI_ISL_10707292	Indian Institute of Technology (IIT) Roorkee	Indian Institute of Technology (IIT) Roorkee	Bhutkar,M.R.
EPI_ISL_10724043	University of Hyderabad	University of Hyderabad	Maisnam,D. and Musturi,V.
EPI_ISL_10952927, EPI_ISL_10968441	General Sir John Kotelawala Defence University, Faculty of Medicine	General Sir John Kotelawala Defence University, Faculty of Medicine	Abeygoonawardena,H., De Silva,A.D., Wijesinghe,N., Navaratne,V., Dilshani,P., Sundralingam,T. and Punchihewa,C.
EPI_ISL_10981739	University of Hyderabad	University of Hyderabad	Maisnam,D. and Musturi,V.
EPI_ISL_11104976, EPI_ISL_11104977, EPI_ISL_11104978, EPI_ISL_11104979	US Army Medical Directorate of the Armed Forces Research Institute of Medical Sciences (USAMD-AFRIMS)	US Army Medical Directorate of the Armed Forces Research Institute of Medical Sciences (USAMD-AFRIMS)	Jones,A.R., Rutvisuttinunt,W., Chinnawirotpisan,P., Manasatienkit,W., Ongajchaowlerd,P., Ellison,D.W., Kalayanarooj,S., Suntarattiwong,P., Fernandez,S. and Klungthong,C.
EPI_ISL_11177149	Ahmadu Bello University	Ahmadu Bello University	Tizhe,D.T., Kwaga,J.K.P. and Kia,G.S.N.
EPI_ISL_11254706, EPI_ISL_11254719, EPI_ISL_11255073, EPI_ISL_11255150, EPI_ISL_11255151, EPI_ISL_11255347, EPI_ISL_11255348, EPI_ISL_11255349, EPI_ISL_11255350, EPI_ISL_11256147, EPI_ISL_11256148, EPI_ISL_11256156, EPI_ISL_11256326, EPI_ISL_11256327, EPI_ISL_11256478, EPI_ISL_11256479, EPI_ISL_11256480, EPI_ISL_11256481, EPI_ISL_11256482, EPI_ISL_11256483, EPI_ISL_11256484, EPI_ISL_11256485, EPI_ISL_11256486, EPI_ISL_11256643, EPI_ISL_11256644, EPI_ISL_11256645, EPI_ISL_11256698, EPI_ISL_11256709, EPI_ISL_11256742, EPI_ISL_11256763, EPI_ISL_11256765, EPI_ISL_11256766, EPI_ISL_11256767, EPI_ISL_11256773, EPI_ISL_11256791, EPI_ISL_11256795, EPI_ISL_11256805, EPI_ISL_11256812, EPI_ISL_11256835, EPI_ISL_11256839, EPI_ISL_11256842, EPI_ISL_11256854, EPI_ISL_11256858, EPI_ISL_11256867, EPI_ISL_11256882, EPI_ISL_11256896, EPI_ISL_11256902, EPI_ISL_11256909, EPI_ISL_11256915, EPI_ISL_11256919, EPI_ISL_11256922, EPI_ISL_11256931, EPI_ISL_11256932, EPI_ISL_11256933, EPI_ISL_11256938, EPI_ISL_11256947, EPI_ISL_11256957, EPI_ISL_11256972, EPI_ISL_11256988, EPI_ISL_11256991, EPI_ISL_11256995, EPI_ISL_11256996, EPI_ISL_11257096, EPI_ISL_11257105, EPI_ISL_11257111, EPI_ISL_11257112, EPI_ISL_11257114, EPI_ISL_11257118, EPI_ISL_11257123, EPI_ISL_11257133, EPI_ISL_11257156, EPI_ISL_11257174, EPI_ISL_11257177, EPI_ISL_11257183, EPI_ISL_11257185, EPI_ISL_11257186, EPI_ISL_11257196, EPI_ISL_11257200, EPI_ISL_11257207, EPI_ISL_11257208, EPI_ISL_11257210, EPI_ISL_11257218, EPI_ISL_11257244, EPI_ISL_11257249, EPI_ISL_11257254, EPI_ISL_11257308, EPI_ISL_11257328, EPI_ISL_11257338, EPI_ISL_11257347, EPI_ISL_11257352, EPI_ISL_11257354, EPI_ISL_11257358, EPI_ISL_11257366, EPI_ISL_11257377, EPI_ISL_11257398, EPI_ISL_11257425, EPI_ISL_11257488, EPI_ISL_11257497, EPI_ISL_11257503, EPI_ISL_11257518, EPI_ISL_11257519, EPI_ISL_11257520, EPI_ISL_11257544, EPI_ISL_11257546, EPI_ISL_11257551, EPI_ISL_11257571, EPI_ISL_11257587, EPI_ISL_11257653, EPI_ISL_11257659, EPI_ISL_11257679, EPI_ISL_11257680, EPI_ISL_11257682, EPI_ISL_11257708, EPI_ISL_11257709, EPI_ISL_11300437, EPI_ISL_11300438, EPI_ISL_11301540, EPI_ISL_11301541, EPI_ISL_11309849, EPI_ISL_11309850, EPI_ISL_11309945, EPI_ISL_11309946, EPI_ISL_11309974, EPI_ISL_11310228, EPI_ISL_11310229, EPI_ISL_11310230, EPI_ISL_11310557, EPI_ISL_11310558, EPI_ISL_11310559, EPI_ISL_11311037, EPI_ISL_11311353, EPI_ISL_11311450, EPI_ISL_11311451, EPI_ISL_11311452, EPI_ISL_11311921, EPI_ISL_11311922, EPI_ISL_11311923, EPI_ISL_11311924, EPI_ISL_11311925, EPI_ISL_11311926, EPI_ISL_11311927, EPI_ISL_11311928, EPI_ISL_11312524, EPI_ISL_11312525, EPI_ISL_11312526, EPI_ISL_11312527, EPI_ISL_11312528, EPI_ISL_11312529, EPI_ISL_11312530, EPI_ISL_11312623, EPI_ISL_11313367, EPI_ISL_11313368, EPI_ISL_11313369, EPI_ISL_11313370, EPI_ISL_11313371, EPI_ISL_11313372, EPI_ISL_11313373, EPI_ISL_11313374, EPI_ISL_11313375, EPI_ISL_11313376, EPI_ISL_11313377, EPI_ISL_11313378, EPI_ISL_11313379, EPI_ISL_11313675, EPI_ISL_11313676, EPI_ISL_11313677, EPI_ISL_11313678, EPI_ISL_11313679, EPI_ISL_11313680, EPI_ISL_11313681, EPI_ISL_11313682, EPI_ISL_11313683, EPI_ISL_11314027, EPI_ISL_11314028, EPI_ISL_11314029, EPI_ISL_11314030, EPI_ISL_11314031, EPI_ISL_11314364, EPI_ISL_11314365, EPI_ISL_11314366, EPI_ISL_11314367, EPI_ISL_11314368, EPI_ISL_11314369, EPI_ISL_11314370, EPI_ISL_11314371, EPI_ISL_11314372, EPI_ISL_11315090, EPI_ISL_11315091, EPI_ISL_11316855, EPI_ISL_11317555, EPI_ISL_11317556, EPI_ISL_11317557, EPI_ISL_11317558, EPI_ISL_11317559, EPI_ISL_11317560, EPI_ISL_11317561, EPI_ISL_11317956, EPI_ISL_11317957, EPI_ISL_11317958, EPI_ISL_11317959, EPI_ISL_11317960, EPI_ISL_11317961, EPI_ISL_11318993, EPI_ISL_11318994, EPI_ISL_11318995, EPI_ISL_11318996, EPI_ISL_11318997, EPI_ISL_11318998, EPI_ISL_11318999, EPI_ISL_11319000, EPI_ISL_11319001, EPI_ISL_11319002, EPI_ISL_11319324, EPI_ISL_11319325, EPI_ISL_11319326, EPI_ISL_11319327, EPI_ISL_11319328, EPI_ISL_11319329, EPI_ISL_11319330, EPI_ISL_11319331, EPI_ISL_11319332, EPI_ISL_11319333, EPI_ISL_11319334, EPI_ISL_11319335, EPI_ISL_11319336, EPI_ISL_11319337, EPI_ISL_11319338, EPI_ISL_11319339, EPI_ISL_11319340, EPI_ISL_11319341, EPI_ISL_11319342, EPI_ISL_11319343, EPI_ISL_11319344, EPI_ISL_11319345, EPI_ISL_11319346, EPI_ISL_11319347, EPI_ISL_11319348, EPI_ISL_11319349, EPI_ISL_11319350, EPI_ISL_11319351, EPI_ISL_11319352, EPI_ISL_11319353, EPI_ISL_11319354, EPI_ISL_11319355, EPI_ISL_11319522, EPI_ISL_11319523, EPI_ISL_11319524, EPI_ISL_11319768, EPI_ISL_11319769, EPI_ISL_11319770, EPI_ISL_11319771, EPI_ISL_11319772, EPI_ISL_11319773, EPI_ISL_11319774, EPI_ISL_11319775, EPI_ISL_11319776, EPI_ISL_11319777, EPI_ISL_11319778, EPI_ISL_11319779, EPI_ISL_11319780, EPI_ISL_11319781, EPI_ISL_11319782, EPI_ISL_11319783, EPI_ISL_11319784, EPI_ISL_11319785, EPI_ISL_11319786, EPI_ISL_11319787, EPI_ISL_11319788, EPI_ISL_11319789, EPI_ISL_11320119, EPI_ISL_11320120, EPI_ISL_11320121, EPI_ISL_11320122, EPI_ISL_11320123, EPI_ISL_11320124, EPI_ISL_11320257, EPI_ISL_11320258, EPI_ISL_11320259, EPI_ISL_11320260, EPI_ISL_11320261, EPI_ISL_11320262, EPI_ISL_11320263, EPI_ISL_11320264, EPI_ISL_11320265, EPI_ISL_11320266, EPI_ISL_11320267, EPI_ISL_11320268, EPI_ISL_11320269, EPI_ISL_11320270, EPI_ISL_11320271, EPI_ISL_11320272, EPI_ISL_11320273, EPI_ISL_11320274, EPI_ISL_11320275, EPI_ISL_11320276, EPI_ISL_11320277, EPI_ISL_11320278, EPI_ISL_11320279, EPI_ISL_11320280, EPI_ISL_11320281, EPI_ISL_11320282, EPI_ISL_11320283, EPI_ISL_11320284, EPI_ISL_11320285, EPI_ISL_11320286, EPI_ISL_11320287, EPI_ISL_11320288, EPI_ISL_11320289, EPI_ISL_11320290, EPI_ISL_11320291, EPI_ISL_11320292, EPI_ISL_11320293, EPI_ISL_11320294, EPI_ISL_11320295, EPI_ISL_11320296, EPI_ISL_11320297			
see above	US Army Medical Directorate of the Armed Forces Research Institute of Medical Sciences (USAMD-AFRIMS)	US Army Medical Directorate of the Armed Forces Research Institute of Medical Sciences (USAMD-AFRIMS)	Klungthong,C., Phonpakobsin,T., Thaisomboonsuk,B., Chinnawirotpisan,P., Manasatienkit,W., Ong-ajchaowlerd,P., Rodpradit,P., Nisalak,A., Kalayanarooj,S., Punpanich Vandepitt,W., Macareo,L.R., Gibbons,R.V., Jarman,R.G. and Fernandez,S.
EPI_ISL_11330490, EPI_ISL_11330583, EPI_ISL_11330672	Shree Guru Gobind Singh Tricentenary University (SGT)	Shree Guru Gobind Singh Tricentenary University (SGT)	Sharma,M.
EPI_ISL_11536036, EPI_ISL_11536037, EPI_ISL_11536765, EPI_ISL_11537627, EPI_ISL_11537628, EPI_ISL_11537629, EPI_ISL_11537630, EPI_ISL_11537631, EPI_ISL_11537632, EPI_ISL_11537633, EPI_ISL_11537634, EPI_ISL_11537635, EPI_ISL_11538419, EPI_ISL_11538420			
see above	US Naval Medical Research Unit 6	US Naval Medical Research Unit 6	Espejo,V.I., Troncos,G.M., Cruz,C.D., Olivos,J.M., Ramos,R.E., Chuquirachi,F.J., Ocana,V.R., Holguin,C.E., Cedano,L.E., Ampuero,J.S., Tinoco,Y.O. and Graf,P.C.
EPI_ISL_11538421	US Naval Medical Research Unit 6	US Naval Medical Research Unit 6	Espejo,V.I., Troncos,G.M., Cruz,C.D., Olivos,J.M., Ramos,R.E., Chuquirachi,F.J., Ocana,V.R., Holguin,C.E., Ampuero,J.S., Tinoco,Y.O. and Graf,P.C.
EPI_ISL_11538422, EPI_ISL_11538423, EPI_ISL_11538424	US Naval Medical Research Unit 6	US Naval Medical Research Unit 6	Espejo,V.I., Troncos,G.M., Cruz,C.D., Olivos,J.M., Ramos,R.E., Chuquirachi,F.J., Ocana,V.R., Holguin,C.E., Cedano,L.E., Ampuero,J.S., Tinoco,Y.O. and Graf,P.C.
EPI_ISL_11538425	US Naval Medical Research Unit 6	US Naval Medical Research Unit 6	Espejo,V.I., Troncos,G.M., Cruz,C.D., Olivos,J.M., Ramos,R.E., Chuquirachi,F.J., Ocana,V.R., Holguin,C.E., Ampuero,J.S., Tinoco,Y.O. and Graf,P.C.
EPI_ISL_11539117, EPI_ISL_11539118, EPI_ISL_11539119, EPI_ISL_11539120, EPI_ISL_11539121, EPI_ISL_11539122	US Naval Medical Research Unit 6	US Naval Medical Research Unit 6	Espejo,V.I., Troncos,G.M., Cruz,C.D., Olivos,J.M., Ramos,R.E., Chuquirachi,F.J., Ocana,V.R., Holguin,C.E., Cedano,L.E., Ampuero,J.S., Tinoco,Y.O. and Graf,P.C.
EPI_ISL_11539123, EPI_ISL_11539124, EPI_ISL_11539125, EPI_ISL_11539126, EPI_ISL_11539127, EPI_ISL_11540732	US Naval Medical Research Unit 6	US Naval Medical Research Unit 6	Espejo,V.I., Troncos,G.M., Cruz,C.D., Olivos,J.M., Ramos,R.E., Chuquirachi,F.J., Ocana,V.R., Holguin,C.E., Ampuero,J.S., Tinoco,Y.O. and Graf,P.C.
EPI_ISL_11542198	Bangladesh Council of Scientific and Industrial Research (BCSIR)	Bangladesh Council of Scientific and Industrial Research (BCSIR)	Sarkar,M.M., Rabbi,M.F., Akter,S., Islam,M.S., Banu,T.A., Jahan,I., Miah,M.I., Goswami,B., Habib,M.A., Akhteruzzaman,S., Shaikh,M.A., Rubaiya,A., Ilias,M. and Khan,M.S.
EPI_ISL_11542199, EPI_ISL_11542200, EPI_ISL_11542201, EPI_ISL_11542202, EPI_ISL_11542203, EPI_ISL_11542204, EPI_ISL_11542205, EPI_ISL_11542206, EPI_ISL_11542207, EPI_ISL_11542208, EPI_ISL_11542209, EPI_ISL_11542210, EPI_ISL_11542211			
see above	Bangladesh Council of Scientific and Industrial Research (BCSIR)	Bangladesh Council of Scientific and Industrial Research (BCSIR)	Sarkar,M.M., Islam,M.S., Miah,M.I., Akter,S., Banu,T.A., Goswami,B., Jahan,I., Habib,M.A., Shaikh,M.A., Rubaiya,A., Ilias,M. and Khan,M.S.
EPI_ISL_11579708, EPI_ISL_11579724, EPI_ISL_11579725, EPI_ISL_11579726, EPI_ISL_11579754, EPI_ISL_11579755, EPI_ISL_11579756, EPI_ISL_11579757, EPI_ISL_11579758, EPI_ISL_11579759, EPI_ISL_11579760, EPI_ISL_11579761, EPI_ISL_11579762, EPI_ISL_11579763, EPI_ISL_11579764, EPI_ISL_11579765, EPI_ISL_11579766, EPI_ISL_11579767, EPI_ISL_11579768, EPI_ISL_11579769, EPI_ISL_11579770, EPI_ISL_11579771			
see above	Faculdade de Medicina de Sao Jose do Rio Preto, Parasitarias e Dermatologicas	Faculdade de Medicina de Sao Jose do Rio Preto, Parasitarias e Dermatologicas	Marques,B.C., Candido,D.S., Estofolete,C.F., Sacchetto,L., Banho,C.A., Dutra,K.R., Salles,F.C., Dourado,F.S., Jesus,J.G., Sabino,E.C., Faria,N. and Nogueira,M.L.
EPI_ISL_11579772, EPI_ISL_11579773, EPI_ISL_11579774, EPI_ISL_11579775, EPI_ISL_11579776, EPI_ISL_11579777, EPI_ISL_11579778, EPI_ISL_11579779, EPI_ISL_11579780, EPI_ISL_11579781, EPI_ISL_11579782, EPI_ISL_11580637			
see above	Bangladesh Council of Scientific and Industrial Research (BCSIR)	Bangladesh Council of Scientific and Industrial Research (BCSIR)	Sarkar,M.M., Islam,M.S., Miah,M.I., Akter,S., Banu,T.A., Goswami,B., Jahan,I., Habib,M.A., Shaikh,M.A., Rubaiya,A., Ilias,M. and Khan,M.S.
EPI_ISL_11629403	Bangladesh Council of Scientific and Industrial Research (BCSIR)	Bangladesh Council of Scientific and Industrial Research (BCSIR)	Sarkar,M.M., Rabbi,M.F., Akter,S., Islam,M.S., Banu,T.A., Jahan,I., Miah,M.I., Goswami,B., Habib,M.A., Akhteruzzaman,S., Shaikh,M.A., Rubaiya,A., Ilias,M. and Khan,M.S.
EPI_ISL_11631874, EPI_ISL_11631875, EPI_ISL_11631876, EPI_ISL_11631877, EPI_ISL_11631878, EPI_ISL_11631879, EPI_ISL_11631880, EPI_ISL_11631881, EPI_ISL_11631882	Bangladesh Council of Scientific and Industrial Research (BCSIR)	Bangladesh Council of Scientific and Industrial Research (BCSIR)	Sarkar,M.M., Islam,M.S., Miah,M.I., Akter,S., Banu,T.A., Goswami,B., Jahan,I., Habib,M.A., Shaikh,M.A., Rubaiya,A., Ilias,M. and Khan,M.S.
EPI_ISL_11971623	Institut de recherche biomédicale des armées (IRBA)	Institut de recherche biomédicale des armées (IRBA)	Durand,G.A., Grard,G., Leparc-Goffart,I. and Piorkowski,G.
EPI_ISL_12001738, EPI_ISL_12001740, EPI_ISL_12001744, EPI_ISL_12001835, EPI_ISL_12001836, EPI_ISL_12001837, EPI_ISL_12001838, EPI_ISL_12001917, EPI_ISL_12002622	Institut Pasteur de Dakar	Institut Pasteur de Dakar	Dieng,I., Diallo,A., Ndiaye,M., Mahmadi,M., Diagne,M.M., Sanke,S., Ndione,M.H.D., Gaye,A., Sagne,S.N., Heraud,J.M., Sall,A.A., Fall,G., Loucoubar,C., Faye,O. and Faye,O.
EPI_ISL_12235795, EPI_ISL_12235796	Nagasaki University	Nagasaki University	Ushijima,Y., Abe,H., Mbadinga,M.J.V.M., Nguema-Ondo,G., Bikangui,R., Agnandji,S.T., Lell,B. and Yasuda,J.
EPI_ISL_12387067	DDBJ	DDBJ	Arunraj,D.
EPI_ISL_12391669	Bernhard Nocht Institute for Tropical Medicine	Bernhard Nocht Institute for Tropical Medicine	Tchibozo,C., Hounknownanrin,G., Bialonski,A., Schmidt-Chanasit,J., Joest,H. and Yadouleton,A.
EPI_ISL_12665303, EPI_ISL_12665304, EPI_ISL_12665305	US Naval Medical Research Unit 6	US Naval Medical Research Unit 6	Espejo,V.I., Troncos,G.M., Cruz,C.D., Gomez-Camargo,D., Cano-Perez,E., Ampuero,J.S., Tinoco,Y.O., Silva,M.E. and Graf,P.C.
EPI_ISL_12869518, EPI_ISL_12869609, EPI_ISL_12869610, EPI_ISL_12869611, EPI_ISL_12869612, EPI_ISL_12869613, EPI_ISL_12869614, EPI_ISL_12869615, EPI_ISL_12869696, EPI_ISL_12869697, EPI_ISL_12869698, EPI_ISL_12869699, EPI_ISL_12869700, EPI_ISL_12869701, EPI_ISL_12869702, EPI_ISL_12869703, EPI_ISL_12869704, EPI_ISL_12869705, EPI_ISL_12869706, EPI_ISL_12869707, EPI_ISL_12869708, EPI_ISL_12869772, EPI_ISL_12869773, EPI_ISL_12869774, EPI_ISL_12869775, EPI_ISL_12869776, EPI_ISL_12869777			
see above	Eijkman Institute for Molecular Biology	Eijkman Institute for Molecular Biology	Masyeni,S., Hayati,R.F., Santoso,M.S. and Sasmono,R.T.
EPI_ISL_12996324	National Institute of Cholera and Enteric Diseases	National Institute of Cholera and Enteric Diseases	Verma,P. and Sadhukhan,P.C.
EPI_ISL_13021966, EPI_ISL_13022216, EPI_ISL_13022217, EPI_ISL_13022218, EPI_ISL_13022219, EPI_ISL_13022220, EPI_ISL_13022221, EPI_ISL_13022222	UMR PIMIT The Mixed Research Unit (UMR), Infectious Processes in Tropical Island Environments (PIMIT)	UMR PIMIT The Mixed Research Unit (UMR), Infectious Processes in Tropical Island Environments (PIMIT)	Hafsia,S., Barbar,T., Wilkinson,D.A., Atyame,C., Biscornet,L., Bibi,J., Louange,M., Gedeon,J., De Santis,O., Flahault,A., Cabie,A., Bertolotti,A. and Mavingui,P.
EPI_ISL_13101925	General Sir John Kotelawala Defence University, Faculty of Medicine	General Sir John Kotelawala Defence University, Faculty of Medicine	Abeygoonawardena,H., De Silva,A.D., Wijesinghe,N., Navaratne,V., Dilshani,P., Sundralingam,T. and Punchihewa,C.

EPI_ISL_13154917	US Naval Medical Research Unit 6	US Naval Medical Research Unit 6	Cruz,C.D., Alba,M., Mores,C., Torre,A.J., Pollet,S. and Leguia,M.
EPI_ISL_13167664	Pondicherry Institute of Medical Sciences (PIMS)	Pondicherry Institute of Medical Sciences (PIMS)	Bhat,S., Nair,S., Natarajan,V. and Pandian,B.
EPI_ISL_13168052, EPI_ISL_13168053, EPI_ISL_13168765, EPI_ISL_13168766, EPI_ISL_13168767, EPI_ISL_13168768, EPI_ISL_13168769, EPI_ISL_13168770, EPI_ISL_13168899, EPI_ISL_13168992, EPI_ISL_13169617, EPI_ISL_13169687, EPI_ISL_13169688, EPI_ISL_13169689, EPI_ISL_13169690, EPI_ISL_13169691, EPI_ISL_13170382, EPI_ISL_13170383, EPI_ISL_13172332, EPI_ISL_13172333, EPI_ISL_13172334, EPI_ISL_13172335, EPI_ISL_13172336, EPI_ISL_13172337, EPI_ISL_13172338			
see above	King George's Medical University	King George's Medical University	Prakash,O., Khan,D.N., Verma,A.K., Ramakrishna,V. and Jain,A.
EPI_ISL_13198227, EPI_ISL_13198228, EPI_ISL_13198229, EPI_ISL_13198230, EPI_ISL_13198384, EPI_ISL_13198492, EPI_ISL_13199356, EPI_ISL_13199357, EPI_ISL_13199358, EPI_ISL_13199390, EPI_ISL_13199579, EPI_ISL_13199681			
see above	Pondicherry Institute of Medical Sciences (PIMS)	Pondicherry Institute of Medical Sciences (PIMS)	Bhat,S., Nair,S., Natarajan,V. and Pandian,B.
EPI_ISL_13321094, EPI_ISL_13321190, EPI_ISL_13321316, EPI_ISL_13321317, EPI_ISL_13321666, EPI_ISL_13321667, EPI_ISL_13321668, EPI_ISL_13321669, EPI_ISL_13321670, EPI_ISL_13321671, EPI_ISL_13321672, EPI_ISL_13321673, EPI_ISL_13321674, EPI_ISL_13321675, EPI_ISL_13321676, EPI_ISL_13321677, EPI_ISL_13321960, EPI_ISL_13321961, EPI_ISL_13321962, EPI_ISL_13321963, EPI_ISL_13321964, EPI_ISL_13321965, EPI_ISL_13321966, EPI_ISL_13321967, EPI_ISL_13321968, EPI_ISL_13321969, EPI_ISL_13321970, EPI_ISL_13321971, EPI_ISL_13321972, EPI_ISL_13321973, EPI_ISL_13321974, EPI_ISL_13321975, EPI_ISL_13321976, EPI_ISL_13337360, EPI_ISL_13337805, EPI_ISL_13337815, EPI_ISL_13337910, EPI_ISL_13340696, EPI_ISL_13340697, EPI_ISL_13340698, EPI_ISL_13340699, EPI_ISL_13340700, EPI_ISL_13340701, EPI_ISL_13340702, EPI_ISL_13340703, EPI_ISL_13340704			
see above	Chulalongkorn University, Faculty of Medicine	Chulalongkorn University, Faculty of Medicine	Khongwichit,S., Chuchaona,W., Vongpunsawad,S. and Poovorawan,Y.
EPI_ISL_13465463, EPI_ISL_13469902, EPI_ISL_13496557, EPI_ISL_13496558, EPI_ISL_13512178, EPI_ISL_13512179	Guangzhou Center for Disease Control and Prevention	Guangzhou Center for Disease Control and Prevention	Su,W., Cao,Y. and Lu,W.
EPI_ISL_13512580	Guangzhou Center for Disease Control and Prevention	Guangzhou Center for Disease Control and Prevention	Su,W., Jiang,L. and Lu,W.
EPI_ISL_13531025, EPI_ISL_13531026, EPI_ISL_13531027, EPI_ISL_13531028, EPI_ISL_13531029, EPI_ISL_13531030, EPI_ISL_13531031, EPI_ISL_13531032, EPI_ISL_13531033, EPI_ISL_13531034, EPI_ISL_13531035, EPI_ISL_13531036, EPI_ISL_13531037, EPI_ISL_13531038, EPI_ISL_13531039, EPI_ISL_13531040, EPI_ISL_13531041, EPI_ISL_13531042, EPI_ISL_13531046, EPI_ISL_13531088, EPI_ISL_13531089			
see above	Guangzhou Center for Disease Control and Prevention	Guangzhou Center for Disease Control and Prevention	Su,W., Cao,Y. and Lu,W.
EPI_ISL_13564328, EPI_ISL_13564401	Guangzhou Center for Disease Control and Prevention	Guangzhou Center for Disease Control and Prevention	Su,W., Jiang,L. and Lu,W.
EPI_ISL_13564402	Guangzhou Center for Disease Control and Prevention	Guangzhou Center for Disease Control and Prevention	Su,W., Cao,Y. and Lu,W.
EPI_ISL_13564403, EPI_ISL_13564404	Guangzhou Center for Disease Control and Prevention	Guangzhou Center for Disease Control and Prevention	Su,W., Jiang,L. and Lu,W.
EPI_ISL_13564405	Guangzhou Center for Disease Control and Prevention	Guangzhou Center for Disease Control and Prevention	Su,W., Cao,Y. and Lu,W.
EPI_ISL_13564406, EPI_ISL_13564407, EPI_ISL_13564408, EPI_ISL_13564409, EPI_ISL_13564410	Guangzhou Center for Disease Control and Prevention	Guangzhou Center for Disease Control and Prevention	Su,W., Jiang,L. and Lu,W.
EPI_ISL_13564411	Guangzhou Center for Disease Control and Prevention	Guangzhou Center for Disease Control and Prevention	Su,W., Cao,Y. and Lu,W.
EPI_ISL_13564412, EPI_ISL_13564413, EPI_ISL_13564414	Guangzhou Center for Disease Control and Prevention	Guangzhou Center for Disease Control and Prevention	Su,W., Jiang,L. and Lu,W.
EPI_ISL_13564415, EPI_ISL_13564416	Guangzhou Center for Disease Control and Prevention	Guangzhou Center for Disease Control and Prevention	Su,W., Cao,Y. and Lu,W.
EPI_ISL_13564417	Guangzhou Center for Disease Control and Prevention	Guangzhou Center for Disease Control and Prevention	Su,W., Jiang,L. and Lu,W.
EPI_ISL_13564418	Guangzhou Center for Disease Control and Prevention	Guangzhou Center for Disease Control and Prevention	Su,W., Jiang,L. and Cao,Y.
EPI_ISL_13564419, EPI_ISL_13564420, EPI_ISL_13564421, EPI_ISL_13564422	Guangzhou Center for Disease Control and Prevention	Guangzhou Center for Disease Control and Prevention	Su,W., Jiang,L. and Lu,W.
EPI_ISL_13564423	Guangzhou Center for Disease Control and Prevention	Guangzhou Center for Disease Control and Prevention	Su,W., Cao,Y. and Lu,W.
EPI_ISL_13564424	Guangzhou Center for Disease Control and Prevention	Guangzhou Center for Disease Control and Prevention	Su,W., Jiang,L. and Lu,W.
EPI_ISL_13564625	Guangzhou Center for Disease Control and Prevention	Guangzhou Center for Disease Control and Prevention	Su,W., Jiang,L. and Cao,Y.
EPI_ISL_13564626, EPI_ISL_13564627, EPI_ISL_13564628, EPI_ISL_13564629, EPI_ISL_13564630	Guangzhou Center for Disease Control and Prevention	Guangzhou Center for Disease Control and Prevention	Su,W., Jiang,L. and Lu,W.
EPI_ISL_13564631	Guangzhou Center for Disease Control and Prevention	Guangzhou Center for Disease Control and Prevention	Su,W., Cao,Y. and Lu,W.
EPI_ISL_13564632	Guangzhou Center for Disease Control and Prevention	Guangzhou Center for Disease Control and Prevention	Su,W., Jiang,L. and Lu,W.
EPI_ISL_13564633	Haizhu District Center for Disease Control and Prevention	Guangdong Center for Disease Control and Prevention	Wu,D., Zhang,H., Zhou,H. and Li,H.
EPI_ISL_13564913	Guangzhou Center for Disease Control and Prevention	Guangzhou Center for Disease Control and Prevention	Su,W., Jiang,L. and Lu,W.
EPI_ISL_13564914	Guangzhou Center for Disease Control and Prevention	Guangzhou Center for Disease Control and Prevention	Su,W., Cao,Y. and Lu,W.
EPI_ISL_13565025, EPI_ISL_13566018, EPI_ISL_13566020, EPI_ISL_13570033, EPI_ISL_13570625, EPI_ISL_13573945	Guangzhou Center for Disease Control and Prevention	Guangzhou Center for Disease Control and Prevention	Su,W., Jiang,L. and Lu,W.
EPI_ISL_13573946	Guangzhou Center for Disease Control and Prevention	Guangzhou Center for Disease Control and Prevention	Su,W., Cao,Y. and Lu,W.
EPI_ISL_13573947, EPI_ISL_13612002	Guangzhou Center for Disease Control and Prevention	Guangzhou Center for Disease Control and Prevention	Su,W., Jiang,L. and Lu,W.
EPI_ISL_13631826, EPI_ISL_13631827, EPI_ISL_13632153, EPI_ISL_13632154, EPI_ISL_13632155, EPI_ISL_13632157, EPI_ISL_13632158, EPI_ISL_13632159, EPI_ISL_13632160, EPI_ISL_13632161, EPI_ISL_13632162, EPI_ISL_13632163, EPI_ISL_13632164, EPI_ISL_13632165			
see above	National Institute of Health	National Institute of Health	Umair,M., Hakim,R., Bibi,S. and Rehman,Z.
EPI_ISL_13632166, EPI_ISL_13632167, EPI_ISL_13632168, EPI_ISL_13632169, EPI_ISL_13632170, EPI_ISL_13632171, EPI_ISL_13632172, EPI_ISL_13632173, EPI_ISL_13632174, EPI_ISL_13632175, EPI_ISL_13632176, EPI_ISL_13632177, EPI_ISL_13632178, EPI_ISL_13632179, EPI_ISL_13632180, EPI_ISL_13632181, EPI_ISL_13632182, EPI_ISL_13632183, EPI_ISL_13632184, EPI_ISL_13632185, EPI_ISL_13632186, EPI_ISL_13632187, EPI_ISL_13632188, EPI_ISL_13632189, EPI_ISL_13632190, EPI_ISL_13632191, EPI_ISL_13632192, EPI_ISL_13632193			
see above	Sichuan Center of Disease Control and Prevention	Sichuan Center of Disease Control and Prevention	Li,W., Jiang,M.F., Zhong,H.R., Cao,Y.O. and Feng,Y.L.
EPI_ISL_13738963, EPI_ISL_13738964, EPI_ISL_13765791, EPI_ISL_13765792, EPI_ISL_13765793, EPI_ISL_13765794, EPI_ISL_13766664, EPI_ISL_13766665, EPI_ISL_13767732, EPI_ISL_13767733, EPI_ISL_13767734, EPI_ISL_13767735, EPI_ISL_13767736, EPI_ISL_13767737, EPI_ISL_13767738, EPI_ISL_13770576, EPI_ISL_13770577, EPI_ISL_13770578, EPI_ISL_13770579, EPI_ISL_13770580, EPI_ISL_13770581, EPI_ISL_13770582, EPI_ISL_13770583, EPI_ISL_13770584, EPI_ISL_13770585, EPI_ISL_13770586, EPI_ISL_13770587, EPI_ISL_13770588, EPI_ISL_13770589, EPI_ISL_13770590, EPI_ISL_13770591			
see above	King George's Medical University	King George's Medical University	Prakash,O., Khan,D.N., Verma,A.K., Ramakrishna,V. and Jain,A.
EPI_ISL_13859884	Vector Control Research Centre	Vector Control Research Centre	Sankari,T., Jayasree,A., Rameela Sanya,K., Varghese,B., Kumar,M., Kumar,A. and Niranjan,R.
EPI_ISL_14081633, EPI_ISL_14081634, EPI_ISL_14081635, EPI_ISL_14081636, EPI_ISL_14081637, EPI_ISL_14081698, EPI_ISL_14081699, EPI_ISL_14081700, EPI_ISL_14081701, EPI_ISL_14081702, EPI_ISL_14083175, EPI_ISL_14083176, EPI_ISL_14083177, EPI_ISL_14083178, EPI_ISL_14083179, EPI_ISL_14083180, EPI_ISL_14083181, EPI_ISL_14083182, EPI_ISL_14083183, EPI_ISL_14083184			
see above	Oxford University Clinical Research Unit	Oxford University Clinical Research Unit	Cao,T.T., Dong,T.H., Nguyen,N.M., Huynh,D.T., Ho,C.Q., Nguyen,P.T., Nguyen,C.V., Phan,Q.T., Huynh,T.T., Luong,T.T., Nguyen,H.V., Duong,T.B., Duong,K.T., Tran,V.T. and Yacoub,S.
EPI_ISL_14253126, EPI_ISL_14253359, EPI_ISL_14253360, EPI_ISL_14253571	ICMR-National Institute of Cholera and Enteric Diseases	ICMR-National Institute of Cholera and Enteric Diseases	Baskey,U., Verma,P. and Sadhukhan,P.C.
EPI_ISL_14287342, EPI_ISL_14287343, EPI_ISL_14287344, EPI_ISL_14287345, EPI_ISL_14287346, EPI_ISL_14287620, EPI_ISL_14288925, EPI_ISL_14288926, EPI_ISL_14288927, EPI_ISL_14289595, EPI_ISL_14289596, EPI_ISL_14289597, EPI_ISL_14289598, EPI_ISL_14289599, EPI_ISL_14289600, EPI_ISL_14289601, EPI_ISL_14289602, EPI_ISL_14289603, EPI_ISL_14289604, EPI_ISL_14289605, EPI_ISL_14289606			
see above	ICMR-National Institute of Cholera and Enteric Diseases	ICMR-National Institute of Cholera and Enteric Diseases	Baskey,U., Verma,P., Ghosh,A. and Sadhukhan,P.C.
EPI_ISL_14675766, EPI_ISL_14675767	Laboratory of Tropical Disease Control of Ministry of Education, Sun Yat-sen University,	Laboratory of Tropical Disease Control of Ministry of Education, Sun Yat-sen University,	Hu,M., Wu,T., Yang,Y., Chen,T., Hao,J., Wei,Y., Luo,T., Wu and Li,Y.P.
EPI_ISL_14810174, EPI_ISL_14810175, EPI_ISL_14810176, EPI_ISL_14810177, EPI_ISL_14810178, EPI_ISL_14810179, EPI_ISL_14810180, EPI_ISL_14810181, EPI_ISL_14810182, EPI_ISL_14810273, EPI_ISL_14810368			
see above	National Institute of Cholera and Enteric	National Institute of Cholera and Enteric Diseases	Sadhukhan,P.C. and Verma,P.

	Diseases		
EPI_ISL_14818767	Bernhard Nocht Institute for Tropical Medicine (BNITM)	Bernhard Nocht Institute for Tropical Medicine (BNITM)	Tchibozo,C., Hounknownanrin,G., Yadouleton,A., Bialonski,A., Agboli,E., Luhken,R., Schmidt-Chanasit,J. and Jost,H.
EPI_ISL_14853337	Guangzhou Center for Disease Control and Prevention	Guangzhou Center for Disease Control and Prevention	Su,W., Cao,Y. and Lu,W.
EPI_ISL_14907012, EPI_ISL_14907478, EPI_ISL_14907479, EPI_ISL_14907480, EPI_ISL_14907481, EPI_ISL_14907482, EPI_ISL_14907483, EPI_ISL_14907615, EPI_ISL_14907616, EPI_ISL_14907617, EPI_ISL_14907618, EPI_ISL_14907619, EPI_ISL_14907620, EPI_ISL_14907621, EPI_ISL_14908177, EPI_ISL_14908230, EPI_ISL_14908231, EPI_ISL_14908232			
see above	The National Environment Agency	The National Environment Agency	Hapuarachchi,H.C., Setoh,Y.X., Wong,J.C.C. and Ng,L.C.
EPI_ISL_14908638, EPI_ISL_14908639, EPI_ISL_14908640, EPI_ISL_14908641, EPI_ISL_14908642, EPI_ISL_14908643, EPI_ISL_14908644, EPI_ISL_14908645, EPI_ISL_14908646, EPI_ISL_14908647, EPI_ISL_14908648, EPI_ISL_14908649, EPI_ISL_14908650, EPI_ISL_14908651, EPI_ISL_14908652, EPI_ISL_14908653, EPI_ISL_14908654, EPI_ISL_14908655, EPI_ISL_14908656, EPI_ISL_14908657, EPI_ISL_14908658, EPI_ISL_14908659, EPI_ISL_14908660, EPI_ISL_14908661, EPI_ISL_14908662, EPI_ISL_14908663, EPI_ISL_14908664, EPI_ISL_14908665, EPI_ISL_14908666, EPI_ISL_14908667, EPI_ISL_14908668, EPI_ISL_14908669, EPI_ISL_14908670, EPI_ISL_14908671, EPI_ISL_14908672, EPI_ISL_14908673, EPI_ISL_14908674, EPI_ISL_14908675, EPI_ISL_14908676, EPI_ISL_14908677, EPI_ISL_14908678, EPI_ISL_14908679, EPI_ISL_14908680, EPI_ISL_14908681, EPI_ISL_14908682, EPI_ISL_14908683, EPI_ISL_14908684, EPI_ISL_14908685, EPI_ISL_14908686, EPI_ISL_14908687, EPI_ISL_14908688, EPI_ISL_14908689, EPI_ISL_14908690, EPI_ISL_14908691, EPI_ISL_14908692, EPI_ISL_14908693, EPI_ISL_14908694, EPI_ISL_14908695, EPI_ISL_14908696, EPI_ISL_14908697, EPI_ISL_14908698, EPI_ISL_14908699, EPI_ISL_14908700, EPI_ISL_14908701, EPI_ISL_14908702, EPI_ISL_14908703, EPI_ISL_14908704, EPI_ISL_14908705, EPI_ISL_14908706, EPI_ISL_14908707, EPI_ISL_14908708, EPI_ISL_14908709, EPI_ISL_14908710, EPI_ISL_14908711, EPI_ISL_14908712, EPI_ISL_14908713, EPI_ISL_14908714, EPI_ISL_14908715, EPI_ISL_14908716, EPI_ISL_14908717, EPI_ISL_14908718, EPI_ISL_14908719, EPI_ISL_14908720, EPI_ISL_14908721, EPI_ISL_14908722, EPI_ISL_14908723, EPI_ISL_14908724, EPI_ISL_14908725, EPI_ISL_14908726, EPI_ISL_14908727, EPI_ISL_14908728, EPI_ISL_14908729, EPI_ISL_14908730, EPI_ISL_14908731, EPI_ISL_14908732, EPI_ISL_14908733, EPI_ISL_14908734, EPI_ISL_14908735, EPI_ISL_14908736, EPI_ISL_14908737, EPI_ISL_14908738, EPI_ISL_14908739, EPI_ISL_14908740, EPI_ISL_14908741, EPI_ISL_14908742, EPI_ISL_14908743, EPI_ISL_14908744, EPI_ISL_14908745, EPI_ISL_14908746, EPI_ISL_14908747, EPI_ISL_14908748, EPI_ISL_14908749, EPI_ISL_14908750, EPI_ISL_14908751, EPI_ISL_14908752, EPI_ISL_14908753, EPI_ISL_14908754, EPI_ISL_14908755, EPI_ISL_14908756, EPI_ISL_14908757, EPI_ISL_14908758, EPI_ISL_14908759, EPI_ISL_14908760, EPI_ISL_14908761, EPI_ISL_14908762, EPI_ISL_14908763			
see above	Instituto Adolfo Lutz - Vector Transmission Diseases	Instituto Adolfo Lutz - Strategic Laboratory	Claudio Tavares Sacchi, Karoline Rodrigues Campos, Marlon Benedito Nascimento Santos, Alex Domingos Reis, Juliana da Silva Nogueira, Adriana Yurika Maeda
EPI_ISL_14908764	LACEN do Estado do Amazonas	Instituto Adolfo Lutz - Strategic Laboratory	Claudio Tavares Sacchi, Karoline Rodrigues Campos, Marlon Benedito Nascimento Santos, Alex Domingos Reis, Juliana da Silva Nogueira, Adriana Yurika Maeda
EPI_ISL_14908765, EPI_ISL_14908766, EPI_ISL_14908767, EPI_ISL_14908768, EPI_ISL_14908769, EPI_ISL_14908770, EPI_ISL_14908771, EPI_ISL_14908772, EPI_ISL_14908773, EPI_ISL_14908774, EPI_ISL_14908775, EPI_ISL_14908776, EPI_ISL_14908777, EPI_ISL_14908778, EPI_ISL_14908779, EPI_ISL_14908780, EPI_ISL_14908781, EPI_ISL_14908782, EPI_ISL_14908783, EPI_ISL_14908784, EPI_ISL_14908785, EPI_ISL_14908786, EPI_ISL_14908787, EPI_ISL_14908788, EPI_ISL_14908789, EPI_ISL_14908790, EPI_ISL_14908791, EPI_ISL_14908792, EPI_ISL_14908793, EPI_ISL_14908794, EPI_ISL_14908795, EPI_ISL_14908796, EPI_ISL_14908797, EPI_ISL_14908798, EPI_ISL_14908799, EPI_ISL_14908800, EPI_ISL_14908801, EPI_ISL_14908802, EPI_ISL_14908803, EPI_ISL_14908804, EPI_ISL_14908805, EPI_ISL_14908806, EPI_ISL_14908807, EPI_ISL_14908808, EPI_ISL_14908809, EPI_ISL_14908810, EPI_ISL_14908811, EPI_ISL_14908812, EPI_ISL_14908813, EPI_ISL_14908814, EPI_ISL_14908815, EPI_ISL_14908816, EPI_ISL_14908817, EPI_ISL_14908818, EPI_ISL_14908819, EPI_ISL_14908820, EPI_ISL_14908821, EPI_ISL_14908822, EPI_ISL_14908823, EPI_ISL_14908824, EPI_ISL_14908825, EPI_ISL_14908826, EPI_ISL_14908827, EPI_ISL_14908828, EPI_ISL_14908829, EPI_ISL_14908830, EPI_ISL_14908831, EPI_ISL_14908832, EPI_ISL_14908833, EPI_ISL_14908834, EPI_ISL_14908835, EPI_ISL_14908836, EPI_ISL_14908837, EPI_ISL_14908838, EPI_ISL_14908839, EPI_ISL_14908840, EPI_ISL_14908841, EPI_ISL_14908842, EPI_ISL_14908843, EPI_ISL_14908844, EPI_ISL_14908845, EPI_ISL_14908846, EPI_ISL_14908847, EPI_ISL_14908848, EPI_ISL_14908849, EPI_ISL_14908850, EPI_ISL_14908851, EPI_ISL_14908852, EPI_ISL_14908853, EPI_ISL_14908854, EPI_ISL_14908855, EPI_ISL_14908856, EPI_ISL_14908857, EPI_ISL_14908858, EPI_ISL_14908859, EPI_ISL_14908860, EPI_ISL_14908861, EPI_ISL_14908862, EPI_ISL_14908863, EPI_ISL_14908864, EPI_ISL_14908865, EPI_ISL_14908866, EPI_ISL_14908867, EPI_ISL_14908868, EPI_ISL_14908869, EPI_ISL_14908870, EPI_ISL_14908871, EPI_ISL_14908872, EPI_ISL_14908873, EPI_ISL_14908874, EPI_ISL_14908875, EPI_ISL_14908876, EPI_ISL_14908877, EPI_ISL_14908878, EPI_ISL_14908879, EPI_ISL_14908880, EPI_ISL_14908881, EPI_ISL_14908882, EPI_ISL_14908883, EPI_ISL_14908884, EPI_ISL_14908885, EPI_ISL_14908886, EPI_ISL_14908887, EPI_ISL_14908888, EPI_ISL_14908889, EPI_ISL_14908890, EPI_ISL_14908891, EPI_ISL_14908892, EPI_ISL_14908893, EPI_ISL_14908894, EPI_ISL_14908895, EPI_ISL_14908896, EPI_ISL_14908897, EPI_ISL_14908898, EPI_ISL_14908899, EPI_ISL_14908900, EPI_ISL_14908901, EPI_ISL_14908902, EPI_ISL_14908903			
see above	Instituto Adolfo Lutz - Vector Transmission Diseases	Instituto Adolfo Lutz - Strategic Laboratory	Claudio Tavares Sacchi, Karoline Rodrigues Campos, Marlon Benedito Nascimento Santos, Alex Domingos Reis, Juliana da Silva Nogueira, Adriana Yurika Maeda
EPI_ISL_14908904	Dengue Branch, Centers for Disease Control and Prevention	Dengue Branch, Centers for Disease Control and Prevention	Santiago,G.A., Gonzalez-Morales,G., Charriez,K., Flores,B., Stanek,D., Morrison,A. and Munoz-Jordan,J.L.
EPI_ISL_14916174, EPI_ISL_14916175, EPI_ISL_14916176, EPI_ISL_14916177, EPI_ISL_14916178, EPI_ISL_14916179, EPI_ISL_14916180, EPI_ISL_14916181, EPI_ISL_14916182, EPI_ISL_14916183, EPI_ISL_14916184, EPI_ISL_14916185			
see above	National Institute of Cholera and Enteric Diseases	National Institute of Cholera and Enteric Diseases	Sadhukhan,P.C. and Verma,P.
EPI_ISL_14931901, EPI_ISL_14932897	Punjab University	Punjab University	Munir,R., Rafique,S., Ali,A., Idrees,M. and Amin,I.
EPI_ISL_14946121	Instituto Nacional de Salud	Instituto Nacional de Salud	Rosales-Munar,A., Rivera,J., Rengifo,A., Diaz-Herrera,T.H., Parra,E., Laiton-Donato,K., Alvarez-Diaz,D.A., Usme-Ciro,J.A. and Caldas,M.L.
EPI_ISL_15017943, EPI_ISL_15017944, EPI_ISL_15018597, EPI_ISL_15018598, EPI_ISL_15019070, EPI_ISL_15019071, EPI_ISL_15019072	Oxford University Clinical Research Unit	Oxford University Clinical Research Unit	Cao,T.T., Dong,T.H., Nguyen,N.M., Huynh,D.T., Ho,C.Q., Nguyen,P.T., Nguyen,C.V., Phan,Q.T., Huynh,T.T., Luong,T.T., Nguyen,H.V., Duong,T.B., Duong,K.T., Tran,V.T. and Yacoub,S.
EPI_ISL_15052681, EPI_ISL_15052682, EPI_ISL_15052683, EPI_ISL_15052684, EPI_ISL_15052685, EPI_ISL_15052686	Instituto Nacional de Salud	Instituto Nacional de Salud	Rosales-Munar,A., Rivera,J., Rengifo,A., Diaz-Herrera,T.H., Parra,E., Laiton-Donato,K., Alvarez-Diaz,D.A., Usme-Ciro,J.A. and Caldas,M.L.
EPI_ISL_15052687	Instituto Nacional de Salud	Instituto Nacional de Salud	Rosales-Munar,A., Rivera,J., Rengifo,A., Diaz-Herrera,T., Parra,E., Laiton-Donato,K., Alvarez-Diaz,D., Usme-Ciro,J. and Caldas,M.
EPI_ISL_15067517, EPI_ISL_15067890, EPI_ISL_15068082, EPI_ISL_15068185, EPI_ISL_15068186, EPI_ISL_15068187, EPI_ISL_15068188, EPI_ISL_15068201, EPI_ISL_15072557, EPI_ISL_15072558, EPI_ISL_15072559, EPI_ISL_15072669, EPI_ISL_15072670, EPI_ISL_15072713			
see above	Center for Disease Control and Prevention	Center for Disease Control and Prevention	Guo,L., Xu,M., Chen,Q., Yang,B. and Wang,J.
EPI_ISL_15078295, EPI_ISL_15078717, EPI_ISL_15078718, EPI_ISL_15078719, EPI_ISL_15078720, EPI_ISL_15078958, EPI_ISL_15079570, EPI_ISL_15079848, EPI_ISL_15079849, EPI_ISL_15079850, EPI_ISL_15079851, EPI_ISL_15082327, EPI_ISL_15083530, EPI_ISL_15083531, EPI_ISL_15083586, EPI_ISL_15083636, EPI_ISL_15083681, EPI_ISL_15083926, EPI_ISL_15083929, EPI_ISL_15083935, EPI_ISL_15084659, EPI_ISL_15085326, EPI_ISL_15085528, EPI_ISL_15085529, EPI_ISL_15088380, EPI_ISL_15088390, EPI_ISL_15088514, EPI_ISL_15088515, EPI_ISL_15088516, EPI_ISL_15088517, EPI_ISL_15088518, EPI_ISL_15088519, EPI_ISL_15088520, EPI_ISL_15088521, EPI_ISL_15088522, EPI_ISL_15088523, EPI_ISL_15088524, EPI_ISL_15088525, EPI_ISL_15088526			
see above	Oxford University Clinical Research Unit	Oxford University Clinical Research Unit	Duong,K.T., Tran,V.T., Dong,T.H., Farrar,J., Tran,N.V., Tran,T.T., Guzman,M.G., Wills,B.A. and Simmons,C.P.
EPI_ISL_15292346, EPI_ISL_15292347, EPI_ISL_15292424, EPI_ISL_15292425, EPI_ISL_15292426	Institute for Medical Research	Infectious Disease Research Centre	Suppiah,J., Ramly,M.N. and Robert,F.
EPI_ISL_15292427	Institute for Medical Research, National Institutes of Health	Institute for Medical Research, National Institutes of Health	Suppiah,J., Ramly,M.N. and Robert,F.
EPI_ISL_15292428, EPI_ISL_15292429	Institute for Medical Research	Infectious Disease Research Centre	Suppiah,J., Ramly,M.N. and Robert,F.
EPI_ISL_15292430, EPI_ISL_15292431, EPI_ISL_15292432, EPI_ISL_15292433	Institute for Medical Research, National Institutes of Health	Institute for Medical Research, National Institutes of Health	Suppiah,J., Ramly,M.N. and Robert,F.
EPI_ISL_15292434	Institute for Medical Research	Infectious Disease Research Centre	Suppiah,J., Ramly,M.N. and Robert,F.
EPI_ISL_15314344	Institute for Medical Research, National Institutes of Health	Institute for Medical Research, National Institutes of Health	Suppiah,J., Ramly,M.N. and Robert,F.
EPI_ISL_15381632, EPI_ISL_15381633, EPI_ISL_15381634	Bangalore University	Bangalore University	Doddamane,M. and Ramachandra,S.
EPI_ISL_15416610, EPI_ISL_15416611, EPI_ISL_15416612, EPI_ISL_15416613, EPI_ISL_15416928, EPI_ISL_15416929, EPI_ISL_15416930, EPI_ISL_15416931, EPI_ISL_15416983, EPI_ISL_15416984, EPI_ISL_15416985, EPI_ISL_15417893, EPI_ISL_15417894, EPI_ISL_15417895, EPI_ISL_15417896, EPI_ISL_15417897, EPI_ISL_15417898			
see above	Hangzhou Center for Disease Control and Prevention	Hangzhou Center for Disease Control and Prevention	Yu,H.
EPI_ISL_15417899, EPI_ISL_15417900	Shenzhen Center for Disease Control and Prevention	Shenzhen Center for Disease Control and Prevention	Yang,F.
EPI_ISL_15417901, EPI_ISL_15417902, EPI_ISL_15417903, EPI_ISL_15417904, EPI_ISL_15417905, EPI_ISL_15417906, EPI_ISL_15417907, EPI_ISL_15417908, EPI_ISL_15417909, EPI_ISL_15417910, EPI_ISL_15417911, EPI_ISL_15417912, EPI_ISL_15417913, EPI_ISL_15417914, EPI_ISL_15417915, EPI_ISL_15417916, EPI_ISL_15417917, EPI_ISL_15417918, EPI_ISL_15417919, EPI_ISL_15417920, EPI_ISL_15417921, EPI_ISL_15417922, EPI_ISL_15417923, EPI_ISL_15417924, EPI_ISL_15417925, EPI_ISL_15417926, EPI_ISL_15417927, EPI_ISL_15417928, EPI_ISL_15417929, EPI_ISL_15417930, EPI_ISL_15417931, EPI_ISL_15417932, EPI_ISL_15417933, EPI_ISL_15417934, EPI_ISL_15417935, EPI_ISL_15417936, EPI_ISL_15417937, EPI_ISL_15417938, EPI_ISL_15417939, EPI_ISL_15417940, EPI_ISL_15417941, EPI_ISL_15417942, EPI_ISL_15417943, EPI_ISL_15417944, EPI_ISL_15417945, EPI_ISL_15417946, EPI_ISL_15417947, EPI_ISL_15419452, EPI_ISL_15420000, EPI_ISL_15420001, EPI_ISL_15420002, EPI_ISL_15420003, EPI_ISL_15420004, EPI_ISL_15420005, EPI_ISL_15420006, EPI_ISL_15420007, EPI_ISL_15420008			
see above	Hangzhou Center for Disease Control and Prevention	Hangzhou Center for Disease Control and Prevention	Yu,H.
EPI_ISL_15572066	Jashore University of Science and Technology	Jashore University of Science and Technology	Rahman,M., Sarker,M.M.H. and Khan,S.
EPI_ISL_15576656, EPI_ISL_15576657	Jashore University of Science and Technology	Jashore University of Science and Technology	Rahman,M.S., Sarker,M.M.H. and Khan,S.
EPI_ISL_15609136, EPI_ISL_15609137, EPI_ISL_15609138, EPI_ISL_15609139	National Institute of Cholera and Enteric Diseases	National Institute of Cholera and Enteric Diseases	Verma,P. and Sadhukhan,D.P.
EPI_ISL_15609140, EPI_ISL_15609141, EPI_ISL_15609145, EPI_ISL_15609146, EPI_ISL_15609147	ICMR- National Institute of Cholera and Enteric Diseases	ICMR- National Institute of Cholera and Enteric Diseases	Verma,P. and Sadhukhan,D.P.
EPI_ISL_15609148, EPI_ISL_15609149, EPI_ISL_15609150	National Institute of Cholera and Enteric Diseases	National Institute of Cholera and Enteric Diseases	Verma,P. and Sadhukhan,D.P.
EPI_ISL_15693125, EPI_ISL_15693223, EPI_ISL_15693224, EPI_ISL_15693225, EPI_ISL_15693304, EPI_ISL_15693396, EPI_ISL_15693935, EPI_ISL_15693936, EPI_ISL_15693937, EPI_ISL_15693938, EPI_ISL_15694080, EPI_ISL_15694081, EPI_ISL_15694082, EPI_ISL_15694083, EPI_ISL_15694084, EPI_ISL_15694085, EPI_ISL_15694086, EPI_ISL_15694087, EPI_ISL_15694088, EPI_ISL_15694089, EPI_ISL_15694091, EPI_ISL_15694092, EPI_ISL_15694093, EPI_ISL_15694094, EPI_ISL_15694095, EPI_ISL_15694096, EPI_ISL_15694097, EPI_ISL_15694098, EPI_ISL_15694099, EPI_ISL_15694100, EPI_ISL_15694101, EPI_ISL_15694102, EPI_ISL_15694103, EPI_ISL_15694104, EPI_ISL_15694105, EPI_ISL_15694106, EPI_ISL_15694107, EPI_ISL_15694527, EPI_ISL_15694528			
see above	Oxford University Clinical Research Unit	Oxford University Clinical Research Unit	Duong,K.T.H., Tran,V.T., Dong,T.H.T., Tran,N.V., Tran,T., Wills,B.A. and Simmons,C.P.
EPI_ISL_15732740, EPI_ISL_15733266, EPI_ISL_15733891, EPI_ISL_15737306, EPI_ISL_15738309, EPI_ISL_15739460, EPI_ISL_15739461, EPI_ISL_15739462	National Institute of Health	National Institute of Health	Umair,M., Rehman,Z., Haider,S.A., Salman,M. and Ikram,A.
EPI_ISL_15739463, EPI_ISL_15739464, EPI_ISL_15739782, EPI_ISL_15739783, EPI_ISL_15739784, EPI_ISL_15739785, EPI_ISL_15739786, EPI_ISL_15739787, EPI_ISL_15739788, EPI_ISL_15739789, EPI_ISL_15739790, EPI_ISL_15739791, EPI_ISL_15739792			
see above	Department of Virology	Department of Virology	Sharma,V., Satapathy,P., Kang,M., Sarkar,S., Dhingra,S., Bora,I., Kaur,K., Goel,K., Aggarwal,A., Arora,N. and Ratho,R.K.
EPI_ISL_15823980	Institute of Medical Sciences	Institute of Medical Sciences	Dinkar,A., Singh,J. and Prakash,P.
EPI_ISL_15917022, EPI_ISL_15917023, EPI_ISL_15917114, EPI_ISL_15917153, EPI_ISL_15917154, EPI_ISL_15917736, EPI_ISL_15917737, EPI_ISL_15917798, EPI_ISL_15917799, EPI_ISL_15917800, EPI_ISL_15917801			
see above	National Public Health Center	National Public Health Center	Nagy,O. and Takacs,M.
EPI_ISL_15923145, EPI_ISL_15926913, EPI_ISL_15926989	National Institute of Health	National Institute of Health	Umair,M., Rehman,Z., Haider,S.A., Salman,M. and Ikram,A.
EPI_ISL_15952840, EPI_ISL_15952841, EPI_ISL_15952842	Bangalore University	Bangalore University	Doddamane,M. and Ramachandra,S.
EPI_ISL_15997588, EPI_ISL_15997589, EPI_ISL_15997590, EPI_ISL_15997591, EPI_ISL_15997592, EPI_ISL_15997593, EPI_ISL_15997594, EPI_ISL_15997595, EPI_ISL_15997596, EPI_ISL_15997597, EPI_ISL_15997598, EPI_ISL_15997599, EPI_ISL_15997600			
see above	Fundação Oswaldo Cruz - Instituto Leônidas e	Fundação Oswaldo Cruz - Instituto Leônidas e Maria	Nascimento,V.A., Souza,V.C., Goncalves,L., Nascimento,F., Mejia,M., Silva,D. and Naveca,F.G.

	Maria Deane (FIOCRUZ)	Deane (FIOCRUZ)	
EPI_ISL_16014609, EPI_ISL_16014610, EPI_ISL_16014676, EPI_ISL_16014677, EPI_ISL_16015137, EPI_ISL_16015138	King George's Medical University	King George's Medical University	Prakash,O., Khan,D.N., Verma,A.K., Mishra,V.K., Srivastava,A.K. and Jain,A.
EPI_ISL_16072648	University of Sao Paulo	University of Sao Paulo	La Serra,L., Cazarotti,R.L.S., Scrich,V.M., Beuno,L.M., Figueiredo,L.T.M., Santos,G.S., de Carvalho,A.N., Amaral,R. and Cassiano,M.H.A.
EPI_ISL_16073484, EPI_ISL_16073485	Guangzhou Center for Disease Control and Prevention	Guangzhou Center for Disease Control and Prevention	Su,W., Cao,Y. and Lu,W.
EPI_ISL_16092157, EPI_ISL_16092158, EPI_ISL_16092166, EPI_ISL_16092323, EPI_ISL_16092324, EPI_ISL_16092325, EPI_ISL_16092326, EPI_ISL_16092327, EPI_ISL_16092328, EPI_ISL_16092329, EPI_ISL_16092330, EPI_ISL_16092331, EPI_ISL_16092332, EPI_ISL_16092333, EPI_ISL_16092334, EPI_ISL_16092335, EPI_ISL_16092336, EPI_ISL_16092337, EPI_ISL_16092338, EPI_ISL_16092339, EPI_ISL_16092340, EPI_ISL_16092341, EPI_ISL_16092342, EPI_ISL_16092343, EPI_ISL_16092344, EPI_ISL_16092345, EPI_ISL_16092346, EPI_ISL_16092347			
see above	Oxford University Clinical Research Unit	Oxford University Clinical Research Unit	Tran,V.T., Duong,K.T.H., Nguyen,N.M., Huynh,D.T.L., Ho,C.Q., Nguyen,P.T., Nguyen,C.V.V., Phan,Q.T., Huynh,T., Luong,T.H., Nguyen,H.V., Duong,T.B., Cao,T., Dong,T.H., Nguyen,H.V., McBride,A., Wills,B., Simmons,C.P. and Yacoub,S.
EPI_ISL_16092348, EPI_ISL_16092349, EPI_ISL_16092350	National Institutes of Health (NIH)	National Institutes of Health (NIH) - Turning Discovery into Health	Bohl,J.A., Lay,S., Chea,S., Ahyong,V., Parker,D.M., Gallagher,S., Fintzi,J., Man,S., Ponce,A., Sreng,S., Kong,D., Oliveira,F., Kalantar,K., Tan,M., Fahsbender,L., Sheu,J., Neff,N., Detweiler,A.M., Yek,C., Ly,S., Sath,R., Huch,C., Kry,H., Leang,R., Huy,R., Lon,C., Tato,C.M., DeRisi,J.L. and Manning,J.E.
EPI_ISL_16178334, EPI_ISL_16178483, EPI_ISL_16179152, EPI_ISL_16179858, EPI_ISL_16179897	General Sir John Kotelawala Defence University, Faculty of Medicine	General Sir John Kotelawala Defence University, Faculty of Medicine	Dassanayake,A.K.K., De Silva,A.D., Abeygoonawardena,H., Kariyawasam,J.C., Chathuranga,T., Sundralingam,T., Dilshani,P., Gunasekara,H., Premawansa,G., Premawansa,S., Weiskopf,D., Sette,A. and Punchihewa,C.
EPI_ISL_16191497	University of Hyderabad	University of Hyderabad	Maisnam,D. and Musturi,V.
EPI_ISL_16212171	General Sir John Kotelawala Defence University, Faculty of Medicine	General Sir John Kotelawala Defence University, Faculty of Medicine	Abeygoonawardena,H., de Silva,A.D., Wijesinghe,N., Navaratne,V., Dilshani,P., Sundralingam,T. and Punchihewa,C.
EPI_ISL_16212210, EPI_ISL_16212211, EPI_ISL_16212212, EPI_ISL_16215353, EPI_ISL_16215354, EPI_ISL_16215355, EPI_ISL_16215356	Universidade Federal de Mato Grosso (UFMT)	Universidade Federal de Mato Grosso (UFMT)	Santos,M.A.M., Pavon,J.A.R., Viniski,A.E., Souza,C.L.C., Oliveira,E.C., Medeiros,D.B.A., Silva,S.P., Nunes,M.R.T. and Slhessarenko,R.D.
EPI_ISL_16222604	General Sir John Kotelawala Defence University, Faculty of Medicine	General Sir John Kotelawala Defence University, Faculty of Medicine	Abeygoonawardena,H., De Silva,A.D., Dassanayake,A.K.K., Kariyawasam,J.C., Chathuranga,T., Sundralingam,T., Dilshani,P., Gunasekara,H., Premawansa,G., Premawansa,S., Weiskopf,D., Sette,A. and Punchihewa,C.
EPI_ISL_16222605	General Sir John Kotelawala Defence University, Faculty of Medicine	General Sir John Kotelawala Defence University, Faculty of Medicine	De Silva,A.D., Dassanayake,A.K.K., Abeygoonawardena,H., Kariyawasam,J.C., Chathuranga,T., Sundralingam,T., Dilshani,P., Gunasekara,H., Premawansa,G., Premawansa,S., Weiskopf,D., Sette,A. and Punchihewa,C.
EPI_ISL_16222606	General Sir John Kotelawala Defence University, Faculty of Medicine	General Sir John Kotelawala Defence University, Faculty of Medicine	Abeygoonawardena,H., De Silva,A.D., Dassanayake,A.K.K., Kariyawasam,J.C., Chathuranga,T., Sundralingam,T., Dilshani,P., Gunasekara,H., Premawansa,G., Premawansa,S., Weiskopf,D., Sette,A. and Punchihewa,C.
EPI_ISL_16222607, EPI_ISL_16222608, EPI_ISL_16222609, EPI_ISL_16222610	General Sir John Kotelawala Defence University, Faculty of Medicine	General Sir John Kotelawala Defence University, Faculty of Medicine	De Silva,A.D., Dassanayake,A.K.K., Abeygoonawardena,H., Kariyawasam,J.C., Chathuranga,T., Sundralingam,T., Dilshani,P., Gunasekara,H., Premawansa,G., Premawansa,S., Weiskopf,D., Sette,A. and Punchihewa,C.
EPI_ISL_16222611, EPI_ISL_16222612, EPI_ISL_16222613	General Sir John Kotelawala Defence University, Faculty of Medicine	General Sir John Kotelawala Defence University, Faculty of Medicine	Abeygoonawardena,H., De Silva,A.D., Dassanayake,A.K.K., Kariyawasam,J.C., Chathuranga,T., Sundralingam,T., Dilshani,P., Gunasekara,H., Premawansa,G., Premawansa,S., Weiskopf,D., Sette,A. and Punchihewa,C.
EPI_ISL_16282284	Universidade Federal de Mato Grosso (UFMT)	Universidade Federal de Mato Grosso (UFMT)	Santos,M.A.M., Pavon,J.A.R., Viniski,A.E., Souza,C.L.C., Oliveira,E.C., Medeiros,D.B.A., Silva,S.P., Nunes,M.R.T. and Slhessarenko,R.D.
EPI_ISL_16287706, EPI_ISL_16287707, EPI_ISL_16287708, EPI_ISL_16287709, EPI_ISL_16287710, EPI_ISL_16287711, EPI_ISL_16287712, EPI_ISL_16287713, EPI_ISL_16287714	All India Institute of Medical Sciences Rishikesh	All India Institute of Medical Sciences Rishikesh	Badoni,G., Gupta,P., Ratho,R.K., Kaistha,N. and Pai,M.O.
EPI_ISL_16287715	All India Institute of Medical Sciences Rishikesh	All India Institute of Medical Sciences Rishikesh	Badoni,G.
EPI_ISL_16287716, EPI_ISL_16299723, EPI_ISL_16299844, EPI_ISL_16299903, EPI_ISL_16299904, EPI_ISL_16299905	All India Institute of Medical Sciences Rishikesh	All India Institute of Medical Sciences Rishikesh	Badoni,G., Gupta,P., Ratho,R.K., Kaistha,N. and Pai,M.O.
EPI_ISL_16312445, EPI_ISL_16312645, EPI_ISL_16312646, EPI_ISL_16312647, EPI_ISL_16312648, EPI_ISL_16312649, EPI_ISL_16312718, EPI_ISL_16312739, EPI_ISL_16312740, EPI_ISL_16312741, EPI_ISL_16312742, EPI_ISL_16312743, EPI_ISL_16312744, EPI_ISL_16312745, EPI_ISL_16312746, EPI_ISL_16312747, EPI_ISL_16312748, EPI_ISL_16312749, EPI_ISL_16312750, EPI_ISL_16312751, EPI_ISL_16312752, EPI_ISL_16312753, EPI_ISL_16312754, EPI_ISL_16312755, EPI_ISL_16312756, EPI_ISL_16312757, EPI_ISL_16312758, EPI_ISL_16312759, EPI_ISL_16312760, EPI_ISL_16312761, EPI_ISL_16312762			
see above	Centers for Disease Control and Prevention	Centers for Disease Control and Prevention	Santiago,G.A., Gonzalez-Morales,G., Flores,B., Charriez,K.N., Reller,M., Matute,A., Reyes,Y., Centeno-Cuadra,E., Bucardo,F. and Munoz-Jordan,J.L.
EPI_ISL_16350698, EPI_ISL_16350704, EPI_ISL_16350805, EPI_ISL_16352470	All India Institute of Medical Sciences Rishikesh	All India Institute of Medical Sciences Rishikesh	Badoni,G., Gupta,P., Ratho,R.K., Kaistha,N. and Pai,M.O.
EPI_ISL_16480244	Universidade Federal de Mato Grosso (UFMT)	Universidade Federal de Mato Grosso (UFMT)	Santos,M.A.M., Pavon,J.A.R., Viniski,A.E., Souza,C.L.C., Oliveira,E.C., Medeiros,D.B.A., Silva,S.P., Nunes,M.R.T. and Slhessarenko,R.D.
EPI_ISL_16482531	Federal University of Mato Grosso	Federal University of Mato Grosso	Santos, M.A.M., Pavon,J.A.R., Viniski,A.E., Souza,C.L.C., Oliveira,E.C., Medeiros,D.B.A., Silva,S.P., Nunes,M.R.T. and Slhessarenko, R.D.
EPI_ISL_17410726	Centers for Disease Control and Prevention, Arboviral Diseases Branch	Centers for Disease Control and Prevention, Arboviral Diseases Branch	Hughes,H.R. and Russell,B.J.
EPI_ISL_17448340, EPI_ISL_17448341, EPI_ISL_17448342, EPI_ISL_17448343, EPI_ISL_17448344, EPI_ISL_17448345, EPI_ISL_17448346, EPI_ISL_17448347	Unit of Omics, Vector Control Research Centre	Unit of Omics, Vector Control Research Centre	Sankari,T., Jayasree,A., Rameela Sanya,K., Varghese,B., Kumar,M., Kumar,A. and Niranjan,R.
EPI_ISL_17448597	Virginia-Maryland College of Veterinary Medicine, Biomedical Sciences and Pathobiology,	Virginia-Maryland College of Veterinary Medicine, Biomedical Sciences and Pathobiology,	Marano,J.M. and Weger-Lucarelli,J.
EPI_ISL_17450850	Infection and Immunology, Translational Health Science and Technology Institute	Infection and Immunology, Translational Health Science and Technology Institute	Maurya,R., Agrawal,T., Saraswat,S., Ananthraj,A., Chandele,A., Kumar,S., Kanakan,A., Chattopadhyay,P., Swaminathan,A., Saifi,S., Shashi,P., Pandey,R. and Medigeshi,G.R.
EPI_ISL_17450851	Infection and Immunology, Translational Health Science and Technology Institute	Infection and Immunology, Translational Health Science and Technology Institute	Medigeshi,G., Pandey,R., Agrawal,T., Saraswat,S., Ananthraj,A., Chandele,A., Maurya,R., Kanakan,A., Chattopadhyay,P., Swaminathan,A. and Saifi,S.
EPI_ISL_17450852	Infection and Immunology, Translational Health Science and Technology Institute	Infection and Immunology, Translational Health Science and Technology Institute	Maurya,R., Agrawal,T., Saraswat,S., Ananthraj,A., Chandele,A., Kumar,S., Kanakan,A., Chattopadhyay,P., Swaminathan,A., Saifi,S., Shashi,P., Pandey,R. and Medigeshi,G.R.
EPI_ISL_17450853	Infection and Immunology, Translational Health Science and Technology Institute	Infection and Immunology, Translational Health Science and Technology Institute	Agrawal,T., Saraswat,S., Shashi,P., Ananthraj,A., Kumar,S., Chandele,A., Lodha,R., Singh,B. and Medigeshi,G.R.
EPI_ISL_17450854	Infection and Immunology, Translational Health Science and Technology Institute	Infection and Immunology, Translational Health Science and Technology Institute	Maurya,R., Agrawal,T., Saraswat,S., Ananthraj,A., Chandele,A., Kumar,S., Kanakan,A., Chattopadhyay,P., Swaminathan,A., Saifi,S., Shashi,P., Pandey,R. and Medigeshi,G.R.
EPI_ISL_17450855	Infection and Immunology, Translational Health Science and Technology Institute	Infection and Immunology, Translational Health Science and Technology Institute	Agrawal,T., Saraswat,S., Shashi,P., Ananthraj,A., Kumar,S., Chandele,A., Lodha,R., Singh,B. and Medigeshi,G.R.
EPI_ISL_17450856	Infection and Immunology, Translational Health Science and Technology Institute	Infection and Immunology, Translational Health Science and Technology Institute	Maurya,R., Agrawal,T., Saraswat,S., Ananthraj,A., Chandele,A., Kumar,S., Kanakan,A., Chattopadhyay,P., Swaminathan,A., Saifi,S., Shashi,P., Pandey,R. and Medigeshi,G.R.
EPI_ISL_17450857, EPI_ISL_17450858	Infection and Immunology, Translational Health Science and Technology Institute	Infection and Immunology, Translational Health Science and Technology Institute	Medigeshi,G., Pandey,R., Agrawal,T., Saraswat,S., Ananthraj,A., Chandele,A., Maurya,R., Kanakan,A., Chattopadhyay,P., Swaminathan,A. and Saifi,S.
EPI_ISL_17450859	Infection and Immunology, Translational Health Science and Technology Institute	Infection and Immunology, Translational Health Science and Technology Institute	Agrawal,T., Saraswat,S., Shashi,P., Ananthraj,A., Kumar,S., Chandele,A., Lodha,R., Singh,B. and Medigeshi,G.R.
EPI_ISL_17450860	Infection and Immunology, Translational Health Science and Technology Institute	Infection and Immunology, Translational Health Science and Technology Institute	Maurya,R., Agrawal,T., Saraswat,S., Ananthraj,A., Chandele,A., Kumar,S., Kanakan,A., Chattopadhyay,P., Swaminathan,A., Saifi,S., Shashi,P., Pandey,R. and Medigeshi,G.R.
EPI_ISL_17450861	Infection and Immunology, Translational Health Science and Technology Institute	Infection and Immunology, Translational Health Science and Technology Institute	Agrawal,T., Saraswat,S., Shashi,P., Ananthraj,A., Kumar,S., Chandele,A., Lodha,R., Singh,B. and Medigeshi,G.R.
EPI_ISL_17450862, EPI_ISL_17450863	Infection and Immunology, Translational Health Science and Technology Institute	Infection and Immunology, Translational Health Science and Technology Institute	Maurya,R., Agrawal,T., Saraswat,S., Ananthraj,A., Chandele,A., Kumar,S., Kanakan,A., Chattopadhyay,P., Swaminathan,A., Saifi,S., Shashi,P., Pandey,R. and Medigeshi,G.R.
EPI_ISL_17450864	Infection and Immunology, Translational Health Science and Technology Institute	Infection and Immunology, Translational Health Science and Technology Institute	Agrawal,T., Saraswat,S., Shashi,P., Ananthraj,A., Kumar,S., Chandele,A., Lodha,R., Singh,B. and Medigeshi,G.R.
EPI_ISL_17450865	Infection and Immunology, Translational Health Science and Technology Institute	Infection and Immunology, Translational Health Science and Technology Institute	Kumar,P., Shashi,P., Singh,B., Verma,C.K., Tripathi,A., Chandele,A., Lodha,R. and Medigeshi,G.R.
EPI_ISL_17450866	Infection and Immunology, Translational Health Science and Technology Institute	Infection and Immunology, Translational Health Science and Technology Institute	Maurya,R., Agrawal,T., Saraswat,S., Ananthraj,A., Chandele,A., Kumar,S., Kanakan,A., Chattopadhyay,P., Swaminathan,A., Saifi,S., Shashi,P., Pandey,R. and Medigeshi,G.R.
EPI_ISL_17450867	Infection and Immunology, Translational Health Science and Technology Institute	Infection and Immunology, Translational Health Science and Technology Institute	Maurya,R., Agrawal,T., Saraswat,S., Ananthraj,A., Chandele,A., Kumar,S., Kanakan,A., Chattopadhyay,P., Swaminathan,A., Saifi,S., Shashi,P., Singh,B., Pandey,R. and Medigeshi,G.R.
EPI_ISL_17450868, EPI_ISL_17450869, EPI_ISL_17450870, EPI_ISL_17450871	Infection and Immunology, Translational Health Science and Technology Institute	Infection and Immunology, Translational Health Science and Technology Institute	Maurya,R., Agrawal,T., Saraswat,S., Ananthraj,A., Chandele,A., Kumar,S., Kanakan,A., Chattopadhyay,P., Swaminathan,A., Saifi,S., Shashi,P., Pandey,R. and Medigeshi,G.R.
EPI_ISL_17450872	Infection and Immunology, Translational Health Science and Technology Institute	Infection and Immunology, Translational Health Science and Technology Institute	Maurya,R., Agrawal,T., Saraswat,S., Ananthraj,A., Chandele,A., Kumar,S., Kanakan,A., Chattopadhyay,P., Swaminathan,A., Saifi,S., Shashi,P., Singh,B., Pandey,R. and Medigeshi,G.R.
EPI_ISL_17450873, EPI_ISL_17450874, EPI_ISL_17450875	Infection and Immunology, Translational Health Science and Technology Institute	Infection and Immunology, Translational Health Science and Technology Institute	Maurya,R., Agrawal,T., Saraswat,S., Ananthraj,A., Chandele,A., Kumar,S., Kanakan,A., Chattopadhyay,P., Swaminathan,A., Saifi,S., Shashi,P., Pandey,R. and Medigeshi,G.R.

EPI_ISL_17450876	Infection and Immunology, Translational Health Science and Technology Institute	Infection and Immunology, Translational Health Science and Technology Institute	Agrawal,T., Saraswat,S., Shashi,P., Ananthraj,A., Kumar,S., Chandele,A., Lodha,R., Singh,B. and Medigeshi,G.R.
EPI_ISL_17450877, EPI_ISL_17450878	Infection and Immunology, Translational Health Science and Technology Institute	Infection and Immunology, Translational Health Science and Technology Institute	Maurya,R., Agrawal,T., Saraswat,S., Ananthraj,A., Chandele,A., Kumar,S., Kanakan,A., Chattopadhyay,P., Swaminathan,A., Saifi,S., Shashi,P., Pandey,R. and Medigeshi,G.R.
EPI_ISL_17450879, EPI_ISL_17450880	Infection and Immunology, Translational Health Science and Technology Institute	Infection and Immunology, Translational Health Science and Technology Institute	Agrawal,T., Saraswat,S., Shashi,P., Ananthraj,A., Kumar,S., Chandele,A., Lodha,R., Singh,B. and Medigeshi,G.R.
EPI_ISL_17450881	Infection and Immunology, Translational Health Science and Technology Institute	Infection and Immunology, Translational Health Science and Technology Institute	Maurya,R., Agrawal,T., Saraswat,S., Ananthraj,A., Chandele,A., Kumar,S., Kanakan,A., Chattopadhyay,P., Swaminathan,A., Saifi,S., Shashi,P., Pandey,R. and Medigeshi,G.R.
EPI_ISL_17450882, EPI_ISL_17450883, EPI_ISL_17450884, EPI_ISL_17450885, EPI_ISL_17450886	Infection and Immunology, Translational Health Science and Technology Institute	Infection and Immunology, Translational Health Science and Technology Institute	Agrawal,T., Saraswat,S., Shashi,P., Ananthraj,A., Kumar,S., Chandele,A., Lodha,R., Singh,B. and Medigeshi,G.R.
EPI_ISL_17450887, EPI_ISL_17450888, EPI_ISL_17450889, EPI_ISL_17450890	Infection and Immunology, Translational Health Science and Technology Institute	Infection and Immunology, Translational Health Science and Technology Institute	Maurya,R., Agrawal,T., Saraswat,S., Ananthraj,A., Chandele,A., Kumar,S., Kanakan,A., Chattopadhyay,P., Swaminathan,A., Saifi,S., Shashi,P., Pandey,R. and Medigeshi,G.R.
EPI_ISL_17450891	Infection and Immunology, Translational Health Science and Technology Institute	Infection and Immunology, Translational Health Science and Technology Institute	Agrawal,T., Saraswat,S., Shashi,P., Ananthraj,A., Kumar,S., Chandele,A., Lodha,R., Singh,B. and Medigeshi,G.R.
EPI_ISL_17450892	Infection and Immunology, Translational Health Science and Technology Institute	Infection and Immunology, Translational Health Science and Technology Institute	Maurya,R., Agrawal,T., Saraswat,S., Ananthraj,A., Chandele,A., Kumar,S., Kanakan,A., Chattopadhyay,P., Swaminathan,A., Saifi,S., Shashi,P., Singh,B., Pandey,R. and Medigeshi,G.R.
EPI_ISL_17450893	Infection and Immunology, Translational Health Science and Technology Institute	Infection and Immunology, Translational Health Science and Technology Institute	Maurya,R., Agrawal,T., Saraswat,S., Ananthraj,A., Chandele,A., Kumar,S., Kanakan,A., Chattopadhyay,P., Swaminathan,A., Saifi,S., Shashi,P., Pandey,R. and Medigeshi,G.R.
EPI_ISL_17450894, EPI_ISL_17450895, EPI_ISL_17450896, EPI_ISL_17450897, EPI_ISL_17450898, EPI_ISL_17450899	Infection and Immunology, Translational Health Science and Technology Institute	Infection and Immunology, Translational Health Science and Technology Institute	Agrawal,T., Saraswat,S., Shashi,P., Ananthraj,A., Kumar,S., Chandele,A., Lodha,R., Singh,B. and Medigeshi,G.R.
EPI_ISL_17450900	Infection and Immunology, Translational Health Science and Technology Institute	Infection and Immunology, Translational Health Science and Technology Institute	Maurya,R., Agrawal,T., Saraswat,S., Ananthraj,A., Chandele,A., Kumar,S., Shashi,P., Singh,B., Lodha,R., Pandey,R. and Medigeshi,G.R.
EPI_ISL_17450901, EPI_ISL_17450902	Infection and Immunology, Translational Health Science and Technology Institute	Infection and Immunology, Translational Health Science and Technology Institute	Maurya,R., Agrawal,T., Saraswat,S., Ananthraj,A., Chandele,A., Kumar,S., Kanakan,A., Chattopadhyay,P., Swaminathan,A., Saifi,S., Shashi,P., Pandey,R. and Medigeshi,G.R.
EPI_ISL_17450903, EPI_ISL_17450904	Infection and Immunology, Translational Health Science and Technology Institute	Infection and Immunology, Translational Health Science and Technology Institute	Agrawal,T., Saraswat,S., Shashi,P., Ananthraj,A., Kumar,S., Chandele,A., Lodha,R., Singh,B. and Medigeshi,G.R.
EPI_ISL_17450905	Infection and Immunology, Translational Health Science and Technology Institute	Infection and Immunology, Translational Health Science and Technology Institute	Maurya,R., Agrawal,T., Saraswat,S., Ananthraj,A., Chandele,A., Kumar,S., Kanakan,A., Chattopadhyay,P., Swaminathan,A., Saifi,S., Shashi,P., Pandey,R. and Medigeshi,G.R.
EPI_ISL_17450906	Infection and Immunology, Translational Health Science and Technology Institute	Infection and Immunology, Translational Health Science and Technology Institute	Agrawal,T., Saraswat,S., Shashi,P., Ananthraj,A., Kumar,S., Chandele,A., Lodha,R., Singh,B. and Medigeshi,G.R.
EPI_ISL_17450907	Infection and Immunology, Translational Health Science and Technology Institute	Infection and Immunology, Translational Health Science and Technology Institute	Kumar,P., Shashi,P., Singh,B., Verma,C.K., Tripathi,A., Chandele,A., Lodha,R. and Medigeshi,G.R.
EPI_ISL_17450908, EPI_ISL_17450909	Infection and Immunology, Translational Health Science and Technology Institute	Infection and Immunology, Translational Health Science and Technology Institute	Verma,C.K., Shashi,P., Singh,B., Kumar,P., Tripathi,A., Chandele,A., Lodha,R. and Medigeshi,G.R.
EPI_ISL_17450910, EPI_ISL_17450911	Infection and Immunology, Translational Health Science and Technology Institute	Infection and Immunology, Translational Health Science and Technology Institute	Maurya,R., Agrawal,T., Saraswat,S., Ananthraj,A., Chandele,A., Kumar,S., Kanakan,A., Chattopadhyay,P., Swaminathan,A., Saifi,S., Shashi,P., Pandey,R. and Medigeshi,G.R.
EPI_ISL_17450912	Infection and Immunology, Translational Health Science and Technology Institute	Infection and Immunology, Translational Health Science and Technology Institute	Agrawal,T., Saraswat,S., Shashi,P., Ananthraj,A., Kumar,S., Chandele,A., Lodha,R., Singh,B. and Medigeshi,G.R.
EPI_ISL_17450913	Infection and Immunology, Translational Health Science and Technology Institute	Infection and Immunology, Translational Health Science and Technology Institute	Kumar,P., Shashi,P., Singh,B., Verma,C.K., Tripathi,A., Chandele,A., Lodha,R. and Medigeshi,G.R.
EPI_ISL_17498037, EPI_ISL_17498038	Center for devices and radiological health - US Food and Drug Administration	Center for devices and radiological health - US Food and Drug Administration	Rios,M., Fares-Gusmao,R., Volkova,E., Grinev,A., Assis,F., Chancey,C., Ok,S., Rocha,B., Sippert,E., Tallon,L., Sadzewicz,L., Vavikolanu,K., Mehta,A., Aluvathingal,J., Nadendla,S., Yan,Y. and Sichtig,H.
EPI_ISL_17498073, EPI_ISL_17498074, EPI_ISL_17498075, EPI_ISL_17498076, EPI_ISL_17498077, EPI_ISL_17498078, EPI_ISL_17498079, EPI_ISL_17498080, EPI_ISL_17498081, EPI_ISL_17498082, EPI_ISL_17498083, EPI_ISL_17498084, EPI_ISL_17498085, EPI_ISL_17498086, EPI_ISL_17498087, EPI_ISL_17498088, EPI_ISL_17498089, EPI_ISL_17498090, EPI_ISL_17498091, EPI_ISL_17498092, EPI_ISL_17498093, EPI_ISL_17498094, EPI_ISL_17498095, EPI_ISL_17498096, EPI_ISL_17498097, EPI_ISL_17498098, EPI_ISL_17498099, EPI_ISL_17498100, EPI_ISL_17498101, EPI_ISL_17498102, EPI_ISL_17498103, EPI_ISL_17498104, EPI_ISL_17498105, EPI_ISL_17498106, EPI_ISL_17498107, EPI_ISL_17498108, EPI_ISL_17498109, EPI_ISL_17498110, EPI_ISL_17498111, EPI_ISL_17498112, EPI_ISL_17498113, EPI_ISL_17498114, EPI_ISL_17498115, EPI_ISL_17498116, EPI_ISL_17498117, EPI_ISL_17498118, EPI_ISL_17498119, EPI_ISL_17498120, EPI_ISL_17498121, EPI_ISL_17498122, EPI_ISL_17498123, EPI_ISL_17498124, EPI_ISL_17498125, EPI_ISL_17498126, EPI_ISL_17498127, EPI_ISL_17498128, EPI_ISL_17498129, EPI_ISL_17498130, EPI_ISL_17498131, EPI_ISL_17498132, EPI_ISL_17498133, EPI_ISL_17498134, EPI_ISL_17498135, EPI_ISL_17498136, EPI_ISL_17498137, EPI_ISL_17498138, EPI_ISL_17498139, EPI_ISL_17498140, EPI_ISL_17498141, EPI_ISL_17498142, EPI_ISL_17498143, EPI_ISL_17498144, EPI_ISL_17498145, EPI_ISL_17498146, EPI_ISL_17498147, EPI_ISL_17498148, EPI_ISL_17498149, EPI_ISL_17498150, EPI_ISL_17498151, EPI_ISL_17498152, EPI_ISL_17498153, EPI_ISL_17498154, EPI_ISL_17498155, EPI_ISL_17498156, EPI_ISL_17498157, EPI_ISL_17498158, EPI_ISL_17498159, EPI_ISL_17498160			
see above	Centers for Disease Control and Prevention, Dengue Branch	Centers for Disease Control and Prevention, Dengue Branch	Santiago,G.A., Gonzalez-Morales,G., Flores,B., Charriez,K., Munoz-Jordan,J.L., Bunch,S., Cano,N., Jaber,R., Morrison,A., Panzera,C., Vergara,J., Zimler,R., Heberlein,L. and Kopp,E.
EPI_ISL_17498885	Infection and Immunology, Translational Health Science and Technology Institute	Infection and Immunology, Translational Health Science and Technology Institute	Verma,C.K., Shashi,P., Singh,B., Kumar,P., Tripathi,A., Chandele,A., Lodha,R. and Medigeshi,G.R.
EPI_ISL_17498976, EPI_ISL_17498977, EPI_ISL_17498978, EPI_ISL_17498979, EPI_ISL_17498980, EPI_ISL_17498981, EPI_ISL_17498982, EPI_ISL_17498983, EPI_ISL_17498984, EPI_ISL_17498985, EPI_ISL_17498986, EPI_ISL_17498987, EPI_ISL_17498988			
see above	Jilin Medical College	Jilin Medical College	Liang,C.
EPI_ISL_17499049	National Institutes of Allergy and Infectious Diseases, Laboratory of Malaria and Vector Research	National Institutes of Allergy and Infectious Diseases, Laboratory of Malaria and Vector Research	Oum,M., Chea,S., Lay,S., Bohl,J.A., Yek,C. and Manning,J.E.
EPI_ISL_17499127, EPI_ISL_17499128, EPI_ISL_17499129, EPI_ISL_17499130, EPI_ISL_17499131, EPI_ISL_17499132, EPI_ISL_17499133, EPI_ISL_17499134, EPI_ISL_17499135, EPI_ISL_17499136, EPI_ISL_17499137, EPI_ISL_17499138, EPI_ISL_17499139, EPI_ISL_17499140, EPI_ISL_17499141, EPI_ISL_17499142, EPI_ISL_17499143, EPI_ISL_17499144, EPI_ISL_17499145, EPI_ISL_17499146, EPI_ISL_17499147, EPI_ISL_17499148, EPI_ISL_17499149, EPI_ISL_17499150, EPI_ISL_17499151, EPI_ISL_17499152, EPI_ISL_17499153, EPI_ISL_17499154, EPI_ISL_17499155, EPI_ISL_17499156, EPI_ISL_17499157, EPI_ISL_17499158, EPI_ISL_17499159, EPI_ISL_17499160, EPI_ISL_17499161, EPI_ISL_17499162, EPI_ISL_17499163, EPI_ISL_17499164, EPI_ISL_17499165, EPI_ISL_17499166, EPI_ISL_17499167, EPI_ISL_17499168, EPI_ISL_17499169, EPI_ISL_17499170			
see above	U. S. Army Medical Component, Armed Forces Research Institute of Medical Sciences	U. S. Army Medical Component, Armed Forces Research Institute of Medical Sciences	Binay Thapa,B., Gyeltshen,S., Klungthong,C., Zangmo,S., Wangchuk,S., Chinnawirotpisan,P., Manasatienkij,W., Fernandez,S. and Jones,A.R.
EPI_ISL_17568981, EPI_ISL_17568982	General Sir John Kotelawala Defence University	General Sir John Kotelawala Defence University	Dassanayake,A.K.K., De Silva,A.D., Kariyawasam,J.C., De Silva,A., Fernando,A.N., Tippalagama,R. and Rajendran,A.
EPI_ISL_17572824, EPI_ISL_17572825, EPI_ISL_17572826, EPI_ISL_17572827, EPI_ISL_17572828, EPI_ISL_17572829	Unité de Mixte de Recherche, Processus Infectieux en Milieu Insulaire Tropical, Dynamique des Systèmes Infectieux Insulaires	Unité de Mixte de Recherche, Processus Infectieux en Milieu Insulaire Tropical, Dynamique des Systèmes Infectieux Insulaires	Hafsia,S., Barbar,T., Wilkinson,D.A., Atyame,C., Biscornet,L., Bibi,J., Louange,M., Gedeon,J., De Santis,O., Flahault,A., Cabie,A., Bertolotti,A. and Mavingui,P.
EPI_ISL_17595688, EPI_ISL_17595690, EPI_ISL_17595691, EPI_ISL_17595692	Takeda Vaccines, Inc.	Takeda Vaccines, Inc.	Wallace,D.
EPI_ISL_17599631, EPI_ISL_17599632, EPI_ISL_17599633, EPI_ISL_17599634, EPI_ISL_17599635, EPI_ISL_17599636, EPI_ISL_17599637, EPI_ISL_17599638	ICMR- National Institute for Research in Environmental Health	ICMR- National Institute for Research in Environmental Health	Sarma,D.K., Rathod,L., Mishra,S., Das,D. and Prakash,A.
EPI_ISL_17599669, EPI_ISL_17599670, EPI_ISL_17599671, EPI_ISL_17599672, EPI_ISL_17599673, EPI_ISL_17599674, EPI_ISL_17599675, EPI_ISL_17599676, EPI_ISL_17599677, EPI_ISL_17599678, EPI_ISL_17599679, EPI_ISL_17599680, EPI_ISL_17599681, EPI_ISL_17599682, EPI_ISL_17599683, EPI_ISL_17599684, EPI_ISL_17599685, EPI_ISL_17599686, EPI_ISL_17599687, EPI_ISL_17599688, EPI_ISL_17599689, EPI_ISL_17599690, EPI_ISL_17599691, EPI_ISL_17599692, EPI_ISL_17599693, EPI_ISL_17599694, EPI_ISL_17599695, EPI_ISL_17599696, EPI_ISL_17599697, EPI_ISL_17599698, EPI_ISL_17599699, EPI_ISL_17599700, EPI_ISL_17599701, EPI_ISL_17599702, EPI_ISL_17599703, EPI_ISL_17599704, EPI_ISL_17599705, EPI_ISL_17599706, EPI_ISL_17599707, EPI_ISL_17599708, EPI_ISL_17599709, EPI_ISL_17599710, EPI_ISL_17599711, EPI_ISL_17599712, EPI_ISL_17599713, EPI_ISL_17599714, EPI_ISL_17599715, EPI_ISL_17599716, EPI_ISL_17599717, EPI_ISL_17599718, EPI_ISL_17599719, EPI_ISL_17599720, EPI_ISL_17599721, EPI_ISL_17599722, EPI_ISL_17599723, EPI_ISL_17599724, EPI_ISL_17599725, EPI_ISL_17599726, EPI_ISL_17599727, EPI_ISL_17599728, EPI_ISL_17599729, EPI_ISL_17599730, EPI_ISL_17599731, EPI_ISL_17599732, EPI_ISL_17599752, EPI_ISL_17599790, EPI_ISL_17599828, EPI_ISL_17599864, EPI_ISL_17599904, EPI_ISL_17599944, EPI_ISL_17599984, EPI_ISL_17600022, EPI_ISL_17600061, EPI_ISL_17600101, EPI_ISL_17600105, EPI_ISL_17600106, EPI_ISL_17600107, EPI_ISL_17600108, EPI_ISL_17600109, EPI_ISL_17600110, EPI_ISL_17600111, EPI_ISL_17600112, EPI_ISL_17600113, EPI_ISL_17600114, EPI_ISL_17600115, EPI_ISL_17600116, EPI_ISL_17600117, EPI_ISL_17600118, EPI_ISL_17600119, EPI_ISL_17600120, EPI_ISL_17600121, EPI_ISL_17600122, EPI_ISL_17600123, EPI_ISL_17600124, EPI_ISL_17600125, EPI_ISL_17600126, EPI_ISL_17600127, EPI_ISL_17600128, EPI_ISL_17600129, EPI_ISL_17600130, EPI_ISL_17600131, EPI_ISL_17600132, EPI_ISL_17600133, EPI_ISL_17600134, EPI_ISL_17600135, EPI_ISL_17600136, EPI_ISL_17600137, EPI_ISL_17600138, EPI_ISL_17600139, EPI_ISL_17600140, EPI_ISL_17600141, EPI_ISL_17600142, EPI_ISL_17600143, EPI_ISL_17600144, EPI_ISL_17600145, EPI_ISL_17600146, EPI_ISL_17600147, EPI_ISL_17600148, EPI_ISL_17600149, EPI_ISL_17600150, EPI_ISL_17600151, EPI_ISL_17600152, EPI_ISL_17600153, EPI_ISL_17600154, EPI_ISL_17600155, EPI_ISL_17600156, EPI_ISL_17600157, EPI_ISL_17600158, EPI_ISL_17600159, EPI_ISL_17600160, EPI_ISL_17600161, EPI_ISL_17600162, EPI_ISL_17600163, EPI_ISL_17600164, EPI_ISL_17600165, EPI_ISL_17600166, EPI_ISL_17600167, EPI_ISL_17600168, EPI_ISL_17600169, EPI_ISL_17600170, EPI_ISL_17600171, EPI_ISL_17600172, EPI_ISL_17600173, EPI_ISL_17600174, EPI_ISL_17600175, EPI_ISL_17600176, EPI_ISL_17600177, EPI_ISL_17600178, EPI_ISL_17600179, EPI_ISL_17600180, EPI_ISL_17600181, EPI_ISL_17600182, EPI_ISL_17600183, EPI_ISL_17600184, EPI_ISL_17600185, EPI_ISL_17600186, EPI_ISL_17600187, EPI_ISL_17600188, EPI_ISL_17600189, EPI_ISL_17600190, EPI_ISL_17600191, EPI_ISL_17600192, EPI_ISL_17600193, EPI_ISL_17600194, EPI_ISL_17600195, EPI_ISL_17600196, EPI_ISL_17600197, EPI_ISL_17600198, EPI_ISL_17600199, EPI_ISL_17600200, EPI_ISL_17600201, EPI_ISL_17600202, EPI_ISL_17600203, EPI_ISL_17600204, EPI_ISL_17600205, EPI_ISL_17600206, EPI_ISL_17600207, EPI_ISL_17600208, EPI_ISL_17600209, EPI_ISL_17600210, EPI_ISL_17600211, EPI_ISL_17600212, EPI_ISL_17600213, EPI_ISL_17600214, EPI_ISL_17600215, EPI_ISL_17600216, EPI_ISL_17600217, EPI_ISL_17600218, EPI_ISL_17600219, EPI_ISL_17600220, EPI_ISL_17600221, EPI_ISL_17600222, EPI_ISL_17600223, EPI_ISL_17600224, EPI_ISL_17600225, EPI_ISL_17600226, EPI_ISL_17600227, EPI_ISL_17600228, EPI_ISL_17600229, EPI_ISL_17600230, EPI_ISL_17600231, EPI_ISL_17600232, EPI_ISL_17600233, EPI_ISL_17600234, EPI_ISL_17600235, EPI_ISL_17600236, EPI_ISL_17600237, EPI_ISL_17600238, EPI_ISL_17600239, EPI_ISL_17600240, EPI_ISL_17600241, EPI_ISL_17600242, EPI_ISL_17600243, EPI_ISL_17600244, EPI_ISL_17600245, EPI_ISL_17600246, EPI_ISL_17600247, EPI_ISL_17600248, EPI_ISL_17600249, EPI_ISL_17600250, EPI_ISL_17600251, EPI_ISL_17600252, EPI_ISL_17600253, EPI_ISL_17600254, EPI_ISL_17600255, EPI_ISL_17600256, EPI_ISL_17600257, EPI_ISL_17600258, EPI_ISL_17600259, EPI_ISL_17600260, EPI_ISL_17600261, EPI_ISL_17600262, EPI_ISL_17600263, EPI_ISL_17600264, EPI_ISL_17600265, EPI_ISL_17600266, EPI_ISL_17600267, EPI_ISL_17600268, EPI_ISL_17600269, EPI_ISL_17600270, EPI_ISL_17600271, EPI_ISL_17600272, EPI_ISL_17600273, EPI_ISL_17600274, EPI_ISL_17600275, EPI_ISL_17600276, EPI_ISL_17600277, EPI_ISL_17600278, EPI_ISL_17600279, EPI_ISL_17600280, EPI_ISL_17600281, EPI_ISL_17600282, EPI_ISL_17600283, EPI_ISL_17600284, EPI_ISL_17600285, EPI_ISL_17600286, EPI_ISL_17600287, EPI_ISL_17600288, EPI_ISL_17600289, EPI_ISL_17600290, EPI_ISL_17600291, EPI_ISL_17600292, EPI_ISL_17600293, EPI_ISL_17600294, EPI_ISL_17600295, EPI_ISL_17600296, EPI_ISL_17600297, EPI_ISL_17600298, EPI_ISL_17600299, EPI_ISL_17600300, EPI_ISL_17600301, EPI_ISL_17600302, EPI_ISL_17600303, EPI_ISL_17600304, EPI_ISL_17600305, EPI_ISL_17600306, EPI_ISL_17600307, EPI_ISL_17600308, EPI_ISL_17600309, EPI_ISL_17600310, EPI_ISL_17600311, EPI_ISL_17600312, EPI_ISL_17600313, EPI_ISL_17600314, EPI_ISL_17600315, EPI_ISL_17600316, EPI_ISL_17600317, EPI_ISL_17600318, EPI_ISL_17600319, EPI_ISL_17600320, EPI_ISL_17600321, EPI_ISL_17600322, EPI_ISL_17600323, EPI_ISL_17600324, EPI_ISL_17600325, EPI_ISL_17600326, EPI_ISL_17600327, EPI_ISL_17600328, EPI_ISL_17600329, EPI_ISL_17600330, EPI_ISL_17600331, EPI_ISL_17600332, EPI_ISL_17600333, EPI_ISL_17600334, EPI_ISL_17600335, EPI_ISL_17600336, EPI_ISL_17600337, EPI_ISL_17600338, EPI_ISL_17600339, EPI_ISL_17600340, EPI_ISL_17600341, EPI_ISL_17600342, EPI_ISL_17600343, EPI_ISL_17600344, EPI_ISL_17600345, EPI_ISL_17600346, EPI_ISL_17600347, EPI_ISL_17600348, EPI_ISL_17600349, EPI_ISL_17600350, EPI_ISL_17600351, EPI_ISL_17600352, EPI_ISL_17600353, EPI_ISL_17600354, EPI_ISL_17600355, EPI_ISL_17600356, EPI_ISL_17600357, EPI_ISL_17600358, EPI_ISL_17600359, EPI_ISL_17600360, EPI_ISL_17600361, EPI_ISL_17600362, EPI_ISL_17600363, EPI_ISL_17600364, EPI_ISL_17600365, EPI_ISL_17600366, EPI_ISL_17600367, EPI_ISL_17600368, EPI_ISL_17600369, EPI_ISL_17600370, EPI_ISL_17600371, EPI_ISL_17600372, EPI_ISL_17600373, EPI_ISL_17600374, EPI_ISL_17600375, EPI_ISL_17600376, EPI_ISL_17600377, EPI_ISL_17600378, EPI_ISL_17600379, EPI_ISL_17600380, EPI_ISL_17600381, EPI_ISL_17600382, EPI_ISL_17600383, EPI_ISL_17600384, EPI_ISL_17600385, EPI_ISL_17600386, EPI_ISL_17600387, EPI_ISL_17600388, EPI_ISL_17600389, EPI_ISL_17600390, EPI_ISL_17600391, EPI_ISL_17600392, EPI_ISL_17600393, EPI_ISL_17600394, EPI_ISL_17600395, EPI_ISL_17600396, EPI_ISL_17600397, EPI_ISL_17600398, EPI_ISL_17600399, EPI_ISL_17600400, EPI_ISL_17600401, EPI_ISL_17600402			
see above	National Health Laboratory	National Health Laboratory	Kelly,M.E., Msafiri,F., Affara,M., Gehre,F., Moremi,N., Mghamba,J., Misinzo,G., Gatei,W., Whistler,T., Joachim,A., Lema,N. and Santiago,G.A.
EPI_ISL_17600403	Universidad de Cordoba	Universidad de Cordoba	Rivero,R., Paternina,D., Tique,V. and Mattar,S.
EPI_ISL_17600404	Centers for Disease Control and Prevention, Dengue Branch	Centers for Disease Control and Prevention, Dengue Branch	Santiago,G.A., Gonzalez-Morales,G.L., Charriez,K.N., Flores,B., Chow,A., Newsum,M., Mendoza,S., Poujilvaia,K., Fitzpatrick,K. and Munoz-Jordan,J.L.
EPI_ISL_17600405	Centers for Disease Control and Prevention,	Centers for Disease Control and Prevention, Dengue	Santiago,G.A., Gonzalez-Morales,G., Flores,B., Charriez,K.N., Falcon,J. and Munoz-Jordan,J.L.

	Dengue Branch	Branch	
EPI_ISL_17600406	Centers for Disease Control and Prevention, Dengue Branch	Centers for Disease Control and Prevention, Dengue Branch	Santiago,G.A., Gonzalez-Morales,G.L., Flores,B., Charriez,K.N., Medina,J., Falcon,J., Brown,G. and Munoz-Jordan,J.L.
EPI_ISL_17600407, EPI_ISL_17600408	Centers for Disease Control and Prevention, Dengue Branch	Centers for Disease Control and Prevention, Dengue Branch	Santiago,G.A., Gonzalez-Morales,G.L., Charriez,K.N., Flores,B., Chow,A., Newsum,M., Mendoza,S., Poujilvaia,K., Fitzpatrick,K. and Munoz-Jordan,J.L.
EPI_ISL_17600409, EPI_ISL_17600410, EPI_ISL_17600411, EPI_ISL_17600412, EPI_ISL_17600413, EPI_ISL_17600414	Centers for Disease Control and Prevention, Dengue Branch	Centers for Disease Control and Prevention, Dengue Branch	Santiago,G.A., Gonzalez-Morales,G.L., Flores,B., Charriez,K.N., Medina,J., Falcon,J., Brown,G. and Munoz-Jordan,J.L.
EPI_ISL_17600462, EPI_ISL_17600463	Department of Epidemiology of Microbial Diseases, Yale School of Public Health	Department of Epidemiology of Microbial Diseases, Yale School of Public Health	Vogels,C.B.F., Breban,M.I., Chaguza,C., Hill,V., Paul,L., Michael,S., Bunch,S., Cano,N., Jaber,R., Morrison,A., Panzera,C., Stryker,I., Vergara,J., Zimler,R., Kopp,E., Herberlein,L. and Grubaugh,N.D.
EPI_ISL_17600464, EPI_ISL_17600465	Department of Epidemiology of Microbial Diseases, Yale School of Public Health	Department of Epidemiology of Microbial Diseases, Yale School of Public Health	Vogels,C.B.F., Breban,M.I., Chaguza,C., Hill,V., Ovalle Segura,J.A., Campechano Perez,C., Jabier,M., Mueses,S., Peguero,A., Cuevas,P., Sancehz Morfe,N., Ruiz,I., Paulino-Ramirez,R. and Grubaugh,N.D.
EPI_ISL_17600466	Department of Epidemiology of Microbial Diseases, Yale School of Public Health	Department of Epidemiology of Microbial Diseases, Yale School of Public Health	Vogels,C.B.F., Breban,M.I., Chaguza,C., Hill,V., Paul,L., Michael,S., Bunch,S., Cano,N., Jaber,R., Morrison,A., Panzera,C., Stryker,I., Vergara,J., Zimler,R., Kopp,E., Herberlein,L. and Grubaugh,N.D.
EPI_ISL_17600467	Department of Epidemiology of Microbial Diseases, Yale School of Public Health	Department of Epidemiology of Microbial Diseases, Yale School of Public Health	Vogels,C.B.F., Breban,M.I., Chaguza,C., Hill,V., Ovalle Segura,J.A., Campechano Perez,C., Jabier,M., Mueses,S., Peguero,A., Cuevas,P., Sancehz Morfe,N., Ruiz,I., Paulino-Ramirez,R. and Grubaugh,N.D.
EPI_ISL_17600468, EPI_ISL_17600469, EPI_ISL_17600470, EPI_ISL_17600471, EPI_ISL_17600472, EPI_ISL_17600473, EPI_ISL_17600474, EPI_ISL_17600475, EPI_ISL_17600476, EPI_ISL_17600477, EPI_ISL_17600478, EPI_ISL_17600479, EPI_ISL_17600480, EPI_ISL_17600481			
see above	Department of Epidemiology of Microbial Diseases, Yale School of Public Health	Department of Epidemiology of Microbial Diseases, Yale School of Public Health	Vogels,C.B.F., Breban,M.I., Chaguza,C., Hill,V., Paul,L., Michael,S., Bunch,S., Cano,N., Jaber,R., Morrison,A., Panzera,C., Stryker,I., Vergara,J., Zimler,R., Kopp,E., Herberlein,L. and Grubaugh,N.D.
EPI_ISL_17600482, EPI_ISL_17600483, EPI_ISL_17600484, EPI_ISL_17600485, EPI_ISL_17600486	Department of Epidemiology of Microbial Diseases, Yale School of Public Health	Department of Epidemiology of Microbial Diseases, Yale School of Public Health	Vogels,C.B.F., Breban,M.I., Chaguza,C., Hill,V., Ovalle Segura,J.A., Campechano Perez,C., Jabier,M., Mueses,S., Peguero,A., Cuevas,P., Sancehz Morfe,N., Ruiz,I., Paulino-Ramirez,R. and Grubaugh,N.D.
EPI_ISL_17600487, EPI_ISL_17600488, EPI_ISL_17600489, EPI_ISL_17600490, EPI_ISL_17600491, EPI_ISL_17600492, EPI_ISL_17600493, EPI_ISL_17600494, EPI_ISL_17600495, EPI_ISL_17600496, EPI_ISL_17600497, EPI_ISL_17600498, EPI_ISL_17600499, EPI_ISL_17600500, EPI_ISL_17600501, EPI_ISL_17600502, EPI_ISL_17600503, EPI_ISL_17600504, EPI_ISL_17600505, EPI_ISL_17600506, EPI_ISL_17600507, EPI_ISL_17600508, EPI_ISL_17600509, EPI_ISL_17600510, EPI_ISL_17600511, EPI_ISL_17600512, EPI_ISL_17600513, EPI_ISL_17600514, EPI_ISL_17600515, EPI_ISL_17600516, EPI_ISL_17600517, EPI_ISL_17600518, EPI_ISL_17600519, EPI_ISL_17600520, EPI_ISL_17600521, EPI_ISL_17600522, EPI_ISL_17600523, EPI_ISL_17600524, EPI_ISL_17600525, EPI_ISL_17600526, EPI_ISL_17600527			
see above	Department of Epidemiology of Microbial Diseases, Yale School of Public Health	Department of Epidemiology of Microbial Diseases, Yale School of Public Health	Vogels,C.B.F., Breban,M.I., Chaguza,C., Hill,V., Paul,L., Michael,S., Bunch,S., Cano,N., Jaber,R., Morrison,A., Panzera,C., Stryker,I., Vergara,J., Zimler,R., Kopp,E., Herberlein,L. and Grubaugh,N.D.
EPI_ISL_17600528, EPI_ISL_17600529	Department of Epidemiology of Microbial Diseases, Yale School of Public Health	Department of Epidemiology of Microbial Diseases, Yale School of Public Health	Vogels,C.B.F., Breban,M.I., Chaguza,C., Hill,V., Ovalle Segura,J.A., Campechano Perez,C., Jabier,M., Mueses,S., Peguero,A., Cuevas,P., Sancehz Morfe,N., Ruiz,I., Paulino-Ramirez,R. and Grubaugh,N.D.
EPI_ISL_17600530, EPI_ISL_17600531	Department of Epidemiology of Microbial Diseases, Yale School of Public Health	Department of Epidemiology of Microbial Diseases, Yale School of Public Health	Vogels,C.B.F., Breban,M.I., Chaguza,C., Hill,V., Norman,F., Galan,J.-C. and Grubaugh,N.D.
EPI_ISL_17600532, EPI_ISL_17600533	Department of Epidemiology of Microbial Diseases, Yale School of Public Health	Department of Epidemiology of Microbial Diseases, Yale School of Public Health	Vogels,C.B.F., Breban,M.I., Chaguza,C., Hill,V., Paul,L., Michael,S., Bunch,S., Cano,N., Jaber,R., Morrison,A., Panzera,C., Stryker,I., Vergara,J., Zimler,R., Kopp,E., Herberlein,L. and Grubaugh,N.D.
EPI_ISL_17600534	Department of Epidemiology of Microbial Diseases, Yale School of Public Health	Department of Epidemiology of Microbial Diseases, Yale School of Public Health	Vogels,C.B.F., Breban,M.I., Chaguza,C., Hill,V., Ovalle Segura,J.A., Campechano Perez,C., Jabier,M., Mueses,S., Peguero,A., Cuevas,P., Sancehz Morfe,N., Ruiz,I., Paulino-Ramirez,R. and Grubaugh,N.D.
EPI_ISL_17600535, EPI_ISL_17600536, EPI_ISL_17600537, EPI_ISL_17600538, EPI_ISL_17600539, EPI_ISL_17600540, EPI_ISL_17600541, EPI_ISL_17600542, EPI_ISL_17600543, EPI_ISL_17600544, EPI_ISL_17600545, EPI_ISL_17600546, EPI_ISL_17600547, EPI_ISL_17600548, EPI_ISL_17600549, EPI_ISL_17600550, EPI_ISL_17600551, EPI_ISL_17600552, EPI_ISL_17600553, EPI_ISL_17600554, EPI_ISL_17600555, EPI_ISL_17600556, EPI_ISL_17600557, EPI_ISL_17600558, EPI_ISL_17600559, EPI_ISL_17600560, EPI_ISL_17600561, EPI_ISL_17600562, EPI_ISL_17600563, EPI_ISL_17600564, EPI_ISL_17600565, EPI_ISL_17600566, EPI_ISL_17600567, EPI_ISL_17600568, EPI_ISL_17600569, EPI_ISL_17600570, EPI_ISL_17600571, EPI_ISL_17600572, EPI_ISL_17600573, EPI_ISL_17600574, EPI_ISL_17600575, EPI_ISL_17600576, EPI_ISL_17600577, EPI_ISL_17600578, EPI_ISL_17600579, EPI_ISL_17600580, EPI_ISL_17600581, EPI_ISL_17600582, EPI_ISL_17600583, EPI_ISL_17600584, EPI_ISL_17600585, EPI_ISL_17600586, EPI_ISL_17600587, EPI_ISL_17600588, EPI_ISL_17600589, EPI_ISL_17600590, EPI_ISL_17600591, EPI_ISL_17600592, EPI_ISL_17600593, EPI_ISL_17600594, EPI_ISL_17600595, EPI_ISL_17600596, EPI_ISL_17600597, EPI_ISL_17600598, EPI_ISL_17600599			
see above	Department of Epidemiology of Microbial Diseases, Yale School of Public Health	Department of Epidemiology of Microbial Diseases, Yale School of Public Health	Vogels,C.B.F., Breban,M.I., Chaguza,C., Hill,V., Paul,L., Michael,S., Bunch,S., Cano,N., Jaber,R., Morrison,A., Panzera,C., Stryker,I., Vergara,J., Zimler,R., Kopp,E., Herberlein,L. and Grubaugh,N.D.
EPI_ISL_17600600	Department of Epidemiology of Microbial Diseases, Yale School of Public Health	Department of Epidemiology of Microbial Diseases, Yale School of Public Health	Vogels,C.B.F., Breban,M.I., Chaguza,C., Hill,V., Ovalle Segura,J.A., Campechano Perez,C., Jabier,M., Mueses,S., Peguero,A., Cuevas,P., Sancehz Morfe,N., Ruiz,I., Paulino-Ramirez,R. and Grubaugh,N.D.
EPI_ISL_17600601, EPI_ISL_17600602, EPI_ISL_17600603, EPI_ISL_17600604, EPI_ISL_17600605, EPI_ISL_17600606, EPI_ISL_17600607, EPI_ISL_17600608, EPI_ISL_17600609, EPI_ISL_17600610, EPI_ISL_17600611, EPI_ISL_17600612, EPI_ISL_17600613, EPI_ISL_17600614, EPI_ISL_17600615, EPI_ISL_17600616, EPI_ISL_17600617, EPI_ISL_17600618, EPI_ISL_17600619, EPI_ISL_17600620, EPI_ISL_17600621, EPI_ISL_17600622, EPI_ISL_17600623, EPI_ISL_17600624, EPI_ISL_17600625, EPI_ISL_17600626, EPI_ISL_17600627, EPI_ISL_17600628, EPI_ISL_17600629, EPI_ISL_17600630, EPI_ISL_17600631, EPI_ISL_17600632, EPI_ISL_17600633, EPI_ISL_17600634, EPI_ISL_17600635, EPI_ISL_17600636, EPI_ISL_17600637, EPI_ISL_17600638, EPI_ISL_17600639, EPI_ISL_17600640, EPI_ISL_17600641, EPI_ISL_17600642, EPI_ISL_17600643, EPI_ISL_17600644, EPI_ISL_17600645, EPI_ISL_17600646, EPI_ISL_17600647, EPI_ISL_17600648, EPI_ISL_17600649, EPI_ISL_17600650, EPI_ISL_17600651, EPI_ISL_17600652, EPI_ISL_17600653, EPI_ISL_17600654, EPI_ISL_17600655, EPI_ISL_17600656, EPI_ISL_17600657, EPI_ISL_17600658, EPI_ISL_17600659, EPI_ISL_17600660, EPI_ISL_17600661, EPI_ISL_17600662, EPI_ISL_17600663, EPI_ISL_17600664, EPI_ISL_17600665, EPI_ISL_17600666, EPI_ISL_17600667, EPI_ISL_17600668, EPI_ISL_17600669, EPI_ISL_17600670, EPI_ISL_17600671, EPI_ISL_17600672, EPI_ISL_17600673, EPI_ISL_17600674, EPI_ISL_17600675, EPI_ISL_17600676, EPI_ISL_17600677, EPI_ISL_17600678, EPI_ISL_17600679, EPI_ISL_17600680, EPI_ISL_17600681, EPI_ISL_17600682, EPI_ISL_17600683, EPI_ISL_17600684, EPI_ISL_17600685, EPI_ISL_17600686, EPI_ISL_17600687, EPI_ISL_17600688, EPI_ISL_17600689, EPI_ISL_17600690, EPI_ISL_17600691, EPI_ISL_17600692, EPI_ISL_17600693, EPI_ISL_17600694, EPI_ISL_17600695, EPI_ISL_17600696, EPI_ISL_17600697, EPI_ISL_17600698, EPI_ISL_17600699, EPI_ISL_17600700, EPI_ISL_17600701, EPI_ISL_17600702, EPI_ISL_17600703, EPI_ISL_17600704, EPI_ISL_17600705, EPI_ISL_17600706, EPI_ISL_17600707, EPI_ISL_17600708, EPI_ISL_17600709, EPI_ISL_17600710, EPI_ISL_17600711, EPI_ISL_17600712, EPI_ISL_17600713, EPI_ISL_17600714, EPI_ISL_17600715, EPI_ISL_17600716, EPI_ISL_17600717, EPI_ISL_17600718, EPI_ISL_17600719, EPI_ISL_17600720, EPI_ISL_17600721, EPI_ISL_17600722, EPI_ISL_17600723, EPI_ISL_17600724, EPI_ISL_17600725, EPI_ISL_17600726, EPI_ISL_17600727, EPI_ISL_17600728, EPI_ISL_17600729, EPI_ISL_17600730, EPI_ISL_17600731, EPI_ISL_17600732, EPI_ISL_17600733, EPI_ISL_17600734, EPI_ISL_17600735, EPI_ISL_17600736, EPI_ISL_17600737, EPI_ISL_17600738, EPI_ISL_17600739, EPI_ISL_17600740, EPI_ISL_17600741, EPI_ISL_17600742, EPI_ISL_17600743, EPI_ISL_17600744, EPI_ISL_17600745, EPI_ISL_17600746, EPI_ISL_17600747, EPI_ISL_17600748, EPI_ISL_17600749, EPI_ISL_17600750, EPI_ISL_17600751, EPI_ISL_17600752, EPI_ISL_17600753, EPI_ISL_17600754, EPI_ISL_17600755, EPI_ISL_17600756, EPI_ISL_17600757, EPI_ISL_17600758, EPI_ISL_17600759, EPI_ISL_17600760, EPI_ISL_17600761, EPI_ISL_17600762, EPI_ISL_17600763, EPI_ISL_17600764, EPI_ISL_17600765, EPI_ISL_17600766, EPI_ISL_17600767, EPI_ISL_17600768, EPI_ISL_17600769, EPI_ISL_17600770, EPI_ISL_17600771, EPI_ISL_17600772, EPI_ISL_17600773, EPI_ISL_17600774, EPI_ISL_17600775, EPI_ISL_17600776, EPI_ISL_17600777, EPI_ISL_17600778, EPI_ISL_17600779, EPI_ISL_17600780, EPI_ISL_17600781, EPI_ISL_17600782, EPI_ISL_17600783, EPI_ISL_17600784, EPI_ISL_17600785, EPI_ISL_17600786, EPI_ISL_17600787, EPI_ISL_17600788, EPI_ISL_17600789, EPI_ISL_17600790, EPI_ISL_17600791, EPI_ISL_17600792, EPI_ISL_17600793, EPI_ISL_17600794, EPI_ISL_17600795, EPI_ISL_17600796, EPI_ISL_17600797, EPI_ISL_17600798, EPI_ISL_17600799, EPI_ISL_17600800, EPI_ISL_17600801			
see above	Department of Epidemiology of Microbial Diseases, Yale School of Public Health	Department of Epidemiology of Microbial Diseases, Yale School of Public Health	Vogels,C.B.F., Breban,M.I., Chaguza,C., Hill,V., Paul,L., Michael,S., Bunch,S., Cano,N., Jaber,R., Morrison,A., Panzera,C., Stryker,I., Vergara,J., Zimler,R., Kopp,E., Herberlein,L. and Grubaugh,N.D.
EPI_ISL_17600802, EPI_ISL_17600803, EPI_ISL_17600804	Department of Microbiology, King George's Medical University	Department of Microbiology, King George's Medical University	Mallikarjun,K., Santhosh,K.S. and Puneeth,T.G.
EPI_ISL_17600805, EPI_ISL_17600806, EPI_ISL_17600807, EPI_ISL_17600808, EPI_ISL_17600809, EPI_ISL_17600810, EPI_ISL_17600811, EPI_ISL_17600812, EPI_ISL_17600813, EPI_ISL_17600814, EPI_ISL_17600815, EPI_ISL_17600816, EPI_ISL_17600817, EPI_ISL_17600818, EPI_ISL_17600819, EPI_ISL_17600820, EPI_ISL_17600821, EPI_ISL_17600822			
see above	Eijkman Institute for Molecular Biology, Dengue Unit	Eijkman Institute for Molecular Biology, Dengue Unit	Sasmono,R.T., Nara,M.B., Santoso,M.S., Nugroho,D.K., Yohan,B., Purnama,A., Boro,A.M., Hayati,R.F., Gae,E.P., Denis,D. and Rana,B.
EPI_ISL_17600823	Infection and Immunology, Translational Health Science and Technology Institute	Infection and Immunology, Translational Health Science and Technology Institute	Verma,C.K., Singh,B., Shashi,P., Chandele,A., Lodha,R. and Medigeshi,G.R.
EPI_ISL_17600824	Infection and Immunology, Translational Health Science and Technology Institute	Infection and Immunology, Translational Health Science and Technology Institute	Singh,B., Shashi,P., Verma,C.K., Chandele,A., Lodha,R. and Medigeshi,G.R.
EPI_ISL_17600825, EPI_ISL_17600826	Infection and Immunology, Translational Health Science and Technology Institute	Infection and Immunology, Translational Health Science and Technology Institute	Verma,C.K., Singh,B., Shashi,P., Chandele,A., Lodha,R. and Medigeshi,G.R.
EPI_ISL_17600827, EPI_ISL_17600828, EPI_ISL_17600829, EPI_ISL_17600830, EPI_ISL_17600831, EPI_ISL_17600832, EPI_ISL_17600833, EPI_ISL_17600834	Infection and Immunology, Translational Health Science and Technology Institute	Infection and Immunology, Translational Health Science and Technology Institute	Singh,B., Shashi,P., Verma,C.K., Chandele,A., Lodha,R. and Medigeshi,G.R.
EPI_ISL_17600835, EPI_ISL_17600836, EPI_ISL_17600837, EPI_ISL_17600838, EPI_ISL_17600839, EPI_ISL_17600840, EPI_ISL_17600841	Institute of Medical Biology, Chinese Academy of Medical Sciences, Peking Union Medical College, Tsinghua University	Institute of Medical Biology, Chinese Academy of Medical Sciences, Peking Union Medical College, Tsinghua University	Zhou,Y., Zhou,Y., Liyang,S., Wang,P., An,J. and Liu,H.
EPI_ISL_17600842, EPI_ISL_17600843, EPI_ISL_17600844, EPI_ISL_17600845, EPI_ISL_17600846, EPI_ISL_17600847, EPI_ISL_17600848, EPI_ISL_17600849, EPI_ISL_17600850, EPI_ISL_17600851, EPI_ISL_17600852, EPI_ISL_17600853, EPI_ISL_17600854			
see above	Institute of Virology, Wenzhou University	Institute of Virology, Wenzhou University	Liu,Y.
EPI_ISL_17600855	Instituto Leonidas e Maria Deane, Fiocruz	Instituto Leonidas e Maria Deane, Fiocruz	Regina Maria Pinto de Figueiredo, Victor Costa de Souza, Luciana Goncalves, Dejanane Silva, Fernanda Oliveira do Nascimento, Matilde Mejia, Valdinete Alves do Nascimento and Felipe Naveca
EPI_ISL_17600856, EPI_ISL_17600857, EPI_ISL_17600858	Laboratório Central de Saúde Pública de Roraima	Instituto Leonidas e Maria Deane, Fiocruz	Victor Costa de Souza, Goncalves,L., Silva,D., Fernanda Oliveira do Nascimento, Mejia,M., Nascimento,V. and Naveca,F.
EPI_ISL_17600859, EPI_ISL_17600860, EPI_ISL_17600861, EPI_ISL_17600862	Laboratory of Malaria and Vector Research, National Institute of Allergy and Infectious Diseases	Laboratory of Malaria and Vector Research, National Institute of Allergy and Infectious Diseases	Bohl,J.A., Lay,S., Chea,S., Ahyong,V., Parker,D.M., Gallagher,S., Fintzi,J., Man,S., Ponce,A., Sreng,S., Kong,D., Oliveira,F., Kalantar,K., Tan,M., Fahsbender,L., Sheu,J., Neff,N., Detweiler,A.M., Yek,C., Ly,S., Sath,R., Huch,C., Kry,H., Leang,R., Huy,R., Lon,C., Tato,C.M., DeRisi,J.L. and Manning,J.E.
EPI_ISL_17600863	Laboratory of Malaria and Vector Research, National Institute of Allergy and Infectious Diseases	Laboratory of Malaria and Vector Research, National Institute of Allergy and Infectious Diseases	Oum,M., Lay,S., Chea,S., Bohl,J.A., Yek,C. and Manning,J.E.
EPI_ISL_17600864	Laboratory of Malaria and Vector Research, National Institute of Allergy and Infectious Diseases	Laboratory of Malaria and Vector Research, National Institute of Allergy and Infectious Diseases	Bohl,J.A., Lay,S., Chea,S., Ahyong,V., Parker,D.M., Gallagher,S., Fintzi,J., Man,S., Ponce,A., Sreng,S., Kong,D., Oliveira,F., Kalantar,K., Tan,M., Fahsbender,L., Sheu,J., Neff,N., Detweiler,A.M., Yek,C., Ly,S., Sath,R., Huch,C., Kry,H., Leang,R., Huy,R., Lon,C., Tato,C.M., DeRisi,J.L. and Manning,J.E.
EPI_ISL_17600865	Laboratory of Malaria and Vector Research, National Institute of Allergy and Infectious Diseases	Laboratory of Malaria and Vector Research, National Institute of Allergy and Infectious Diseases	Oum,M., Lay,S., Chea,S., Bohl,J.A., Yek,C. and Manning,J.E.
EPI_ISL_17600866, EPI_ISL_17600867, EPI_ISL_17600868, EPI_ISL_17600869, EPI_ISL_17600870	Laboratory of Malaria and Vector Research, National Institute of Allergy and Infectious Diseases	Laboratory of Malaria and Vector Research, National Institute of Allergy and Infectious Diseases	Bohl,J.A., Lay,S., Chea,S., Ahyong,V., Parker,D.M., Gallagher,S., Fintzi,J., Man,S., Ponce,A., Sreng,S., Kong,D., Oliveira,F., Kalantar,K., Tan,M., Fahsbender,L., Sheu,J., Neff,N., Detweiler,A.M., Yek,C., Ly,S., Sath,R., Huch,C., Kry,H., Leang,R., Huy,R., Lon,C., Tato,C.M., DeRisi,J.L. and Manning,J.E.
EPI_ISL_17600871	Laboratory of Malaria and Vector Research, National Institute of Allergy and Infectious	Laboratory of Malaria and Vector Research, National Institute of Allergy and Infectious Diseases	Oum,M., Lay,S., Chea,S., Bohl,J.A., Yek,C. and Manning,J.E.

	Diseases		
EPI_ISL_17600872	Laboratory of Malaria and Vector Research, National Institute of Allergy and Infectious Diseases	Laboratory of Malaria and Vector Research, National Institute of Allergy and Infectious Diseases	Bohl,J.A., Lay,S., Chea,S., Ahyong,V., Parker,D.M., Gallagher,S., Fintzi,J., Man,S., Ponce,A., Sreng,S., Kong,D., Oliveira,F., Kalantar,K., Tan,M., Fahsbender,L., Sheu,J., Neff,N., Detweiler,A.M., Yek,C., Ly,S., Sath,R., Huch,C., Kry,H., Leang,R., Huy,R., Lon,C., Tato,C.M., DeRisi,J.L. and Manning,J.E.
EPI_ISL_17600873	Laboratory of Malaria and Vector Research, National Institute of Allergy and Infectious Diseases	Laboratory of Malaria and Vector Research, National Institute of Allergy and Infectious Diseases	Oum,M., Lay,S., Chea,S., Bohl,J.A., Yek,C. and Manning,J.E.
EPI_ISL_17600874	Laboratory of Malaria and Vector Research, National Institute of Allergy and Infectious Diseases	Laboratory of Malaria and Vector Research, National Institute of Allergy and Infectious Diseases	Bohl,J.A., Lay,S., Chea,S., Ahyong,V., Parker,D.M., Gallagher,S., Fintzi,J., Man,S., Ponce,A., Sreng,S., Kong,D., Oliveira,F., Kalantar,K., Tan,M., Fahsbender,L., Sheu,J., Neff,N., Detweiler,A.M., Yek,C., Ly,S., Sath,R., Huch,C., Kry,H., Leang,R., Huy,R., Lon,C., Tato,C.M., DeRisi,J.L. and Manning,J.E.
EPI_ISL_17600888, EPI_ISL_17600889, EPI_ISL_17600890, EPI_ISL_17600891, EPI_ISL_17600892, EPI_ISL_17600893, EPI_ISL_17600894, EPI_ISL_17600895, EPI_ISL_17600896	Virology, Oswaldo Cruz Foundation	Virology, Oswaldo Cruz Foundation	Mendonca,M.C.L., Cavalcanti,A.C., Camargo,A.C., Rodrigues,C.D., Fabri,A.A., Santos,C.C. and Filippis,A.M.
EPI_ISL_17600897, EPI_ISL_17600898, EPI_ISL_17600899, EPI_ISL_17600900, EPI_ISL_17600901, EPI_ISL_17600902, EPI_ISL_17600903, EPI_ISL_17600904, EPI_ISL_17600905	Virus Laboratory, Indian Council of Medical Research, National Institute of Cholera and Enteric Diseases	Virus Laboratory, Indian Council of Medical Research, National Institute of Cholera and Enteric Diseases	Baskey,U., Verma,P., Ghosh,A. and Sadhukhan,P.C.
EPI_ISL_17609476	Center for Disease Control and Prevention of Henan Province	Center for Disease Control and Prevention of Henan Province	Li,D.X. and Li,Y.
EPI_ISL_17609477	Centers for Disease Control and Prevention, Arboviral Diseases Branch	Centers for Disease Control and Prevention, Arboviral Diseases Branch	Hughes,H.R. and Russell,B.J.
EPI_ISL_17609844	Laboratório Central do Estado do Paraná	Instituto Leonidas e Maria Deane, Fiocruz	Presibella,M., Marques,N.F., Riediger,I.N., Victor Costa de Souza, Goncalves,L., Silva,D., Fernanda Oliveira do Nascimento, Mejia,M., Nascimento,V. and Naveca,F.
EPI_ISL_17609855, EPI_ISL_17609856	King George's Medical University, Department of Microbiology	King George's Medical University, Department of Microbiology	Prakash,O.
EPI_ISL_17609857, EPI_ISL_17609858, EPI_ISL_17609859, EPI_ISL_17609860, EPI_ISL_17609861, EPI_ISL_17609862, EPI_ISL_17609863, EPI_ISL_17609864, EPI_ISL_17609865, EPI_ISL_17609866, EPI_ISL_17609867, EPI_ISL_17609868, EPI_ISL_17609869, EPI_ISL_17609870, EPI_ISL_17609871, EPI_ISL_17609872, EPI_ISL_17609873, EPI_ISL_17609874, EPI_ISL_17609875, EPI_ISL_17609876, EPI_ISL_17609877, EPI_ISL_17609878, EPI_ISL_17609879, EPI_ISL_17609880, EPI_ISL_17609881, EPI_ISL_17609882, EPI_ISL_17609883, EPI_ISL_17609884, EPI_ISL_17609885, EPI_ISL_17609886, EPI_ISL_17609887, EPI_ISL_17609888, EPI_ISL_17609889, EPI_ISL_17609890, EPI_ISL_17609891, EPI_ISL_17609892, EPI_ISL_17609893, EPI_ISL_17609894, EPI_ISL_17609895, EPI_ISL_17609896, EPI_ISL_17609897, EPI_ISL_17609898, EPI_ISL_17609899, EPI_ISL_17609900, EPI_ISL_17609901, EPI_ISL_17609902, EPI_ISL_17609903, EPI_ISL_17609904, EPI_ISL_17609905, EPI_ISL_17609906, EPI_ISL_17609907, EPI_ISL_17609908, EPI_ISL_17609909, EPI_ISL_17609910, EPI_ISL_17609911, EPI_ISL_17609912, EPI_ISL_17609913, EPI_ISL_17609914, EPI_ISL_17609915, EPI_ISL_17609916, EPI_ISL_17609917, EPI_ISL_17609918, EPI_ISL_17609919, EPI_ISL_17609920, EPI_ISL_17609921, EPI_ISL_17609922, EPI_ISL_17609923, EPI_ISL_17609924, EPI_ISL_17609925, EPI_ISL_17609926, EPI_ISL_17609927, EPI_ISL_17609928, EPI_ISL_17609929, EPI_ISL_17609930, EPI_ISL_17609931, EPI_ISL_17609932, EPI_ISL_17609933, EPI_ISL_17609934, EPI_ISL_17609935, EPI_ISL_17609936, EPI_ISL_17609937, EPI_ISL_17609938, EPI_ISL_17609939, EPI_ISL_17609940, EPI_ISL_17609941, EPI_ISL_17609942, EPI_ISL_17609943, EPI_ISL_17609944, EPI_ISL_17609945, EPI_ISL_17609946, EPI_ISL_17609947, EPI_ISL_17609948, EPI_ISL_17609949, EPI_ISL_17609950, EPI_ISL_17609951, EPI_ISL_17609952, EPI_ISL_17609953, EPI_ISL_17609954, EPI_ISL_17609955, EPI_ISL_17609956, EPI_ISL_17609957, EPI_ISL_17609958, EPI_ISL_17609959, EPI_ISL_17609960, EPI_ISL_17609961, EPI_ISL_17609962, EPI_ISL_17609963, EPI_ISL_17609964, EPI_ISL_17609965, EPI_ISL_17609966, EPI_ISL_17609967, EPI_ISL_17609968, EPI_ISL_17609969, EPI_ISL_17609970, EPI_ISL_17609971, EPI_ISL_17609972, EPI_ISL_17609973, EPI_ISL_17609974, EPI_ISL_17609975, EPI_ISL_17609976, EPI_ISL_17609977, EPI_ISL_17609978, EPI_ISL_17609979, EPI_ISL_17609980, EPI_ISL_17609981, EPI_ISL_17609982, EPI_ISL_17609983, EPI_ISL_17609984, EPI_ISL_17609985, EPI_ISL_17609986, EPI_ISL_17609987, EPI_ISL_17609988, EPI_ISL_17609989, EPI_ISL_17609990, EPI_ISL_17609991, EPI_ISL_17609992, EPI_ISL_17609993, EPI_ISL_17609994, EPI_ISL_17609995, EPI_ISL_17609996, EPI_ISL_17609997, EPI_ISL_17609998, EPI_ISL_17609999, EPI_ISL_17610000, EPI_ISL_17610013, EPI_ISL_17610071, EPI_ISL_17610089, EPI_ISL_17610090, EPI_ISL_17610091, EPI_ISL_17610092, EPI_ISL_17610093, EPI_ISL_17610094, EPI_ISL_17610095, EPI_ISL_17610096, EPI_ISL_17610097, EPI_ISL_17610098, EPI_ISL_17610099, EPI_ISL_17610100, EPI_ISL_17610101, EPI_ISL_17610102, EPI_ISL_17610103, EPI_ISL_17610104, EPI_ISL_17610105, EPI_ISL_17610106, EPI_ISL_17610107, EPI_ISL_17610108, EPI_ISL_17610109, EPI_ISL_17610110, EPI_ISL_17610111, EPI_ISL_17610112, EPI_ISL_17610113, EPI_ISL_17610114, EPI_ISL_17610115, EPI_ISL_17610116, EPI_ISL_17610117, EPI_ISL_17610118, EPI_ISL_17610119, EPI_ISL_17610120, EPI_ISL_17610121, EPI_ISL_17610122, EPI_ISL_17610123, EPI_ISL_17610124, EPI_ISL_17610125, EPI_ISL_17610126, EPI_ISL_17610127, EPI_ISL_17610128, EPI_ISL_17610129, EPI_ISL_17610130, EPI_ISL_17610131, EPI_ISL_17610132, EPI_ISL_17610133, EPI_ISL_17610134, EPI_ISL_17610135, EPI_ISL_17610136, EPI_ISL_17610137, EPI_ISL_17610138, EPI_ISL_17610139, EPI_ISL_17610140, EPI_ISL_17610141, EPI_ISL_17610142, EPI_ISL_17610143, EPI_ISL_17610144, EPI_ISL_17610145, EPI_ISL_17610146, EPI_ISL_17610147, EPI_ISL_17610148, EPI_ISL_17610149, EPI_ISL_17610150, EPI_ISL_17610151, EPI_ISL_17610152, EPI_ISL_17610153, EPI_ISL_17610154, EPI_ISL_17610155, EPI_ISL_17610156, EPI_ISL_17610157, EPI_ISL_17610158, EPI_ISL_17610159, EPI_ISL_17610160, EPI_ISL_17610161, EPI_ISL_17610162, EPI_ISL_17610163, EPI_ISL_17610164, EPI_ISL_17610165, EPI_ISL_17610166, EPI_ISL_17610167, EPI_ISL_17610168, EPI_ISL_17610169, EPI_ISL_17610170, EPI_ISL_17610171, EPI_ISL_17610172, EPI_ISL_17610173, EPI_ISL_17610174, EPI_ISL_17610175, EPI_ISL_17610176, EPI_ISL_17610177, EPI_ISL_17610178, EPI_ISL_17610179, EPI_ISL_17610180, EPI_ISL_17610181, EPI_ISL_17610182, EPI_ISL_17610183, EPI_ISL_17610184, EPI_ISL_17610185, EPI_ISL_17610186, EPI_ISL_17610187, EPI_ISL_17610188, EPI_ISL_17610189, EPI_ISL_17610190, EPI_ISL_17610191, EPI_ISL_17610192, EPI_ISL_17610193, EPI_ISL_17610194, EPI_ISL_17610195, EPI_ISL_17610196, EPI_ISL_17610197, EPI_ISL_17610198, EPI_ISL_17610199, EPI_ISL_17610200, EPI_ISL_17610201, EPI_ISL_17610202, EPI_ISL_17610203, EPI_ISL_17610204, EPI_ISL_17610205, EPI_ISL_17610206, EPI_ISL_17610207, EPI_ISL_17610208, EPI_ISL_17610209, EPI_ISL_17610210, EPI_ISL_17610211, EPI_ISL_17610212, EPI_ISL_17610213, EPI_ISL_17610214, EPI_ISL_17610215, EPI_ISL_17610216, EPI_ISL_17610217, EPI_ISL_17610218, EPI_ISL_17610219, EPI_ISL_17610220, EPI_ISL_17610221, EPI_ISL_17610222, EPI_ISL_17610223, EPI_ISL_17610224, EPI_ISL_17610225, EPI_ISL_17610226, EPI_ISL_17610227, EPI_ISL_17610228, EPI_ISL_17610229, EPI_ISL_17610230, EPI_ISL_17610231, EPI_ISL_17610232, EPI_ISL_17610233, EPI_ISL_17610234, EPI_ISL_17610235, EPI_ISL_17610236, EPI_ISL_17610237, EPI_ISL_17610238, EPI_ISL_17610239, EPI_ISL_17610240, EPI_ISL_17610241, EPI_ISL_17610242, EPI_ISL_17610243, EPI_ISL_17610244, EPI_ISL_17610245, EPI_ISL_17610246, EPI_ISL_17610247, EPI_ISL_17610248, EPI_ISL_17610249, EPI_ISL_17610250, EPI_ISL_17610251, EPI_ISL_17610252, EPI_ISL_17610253, EPI_ISL_17610254, EPI_ISL_17610255, EPI_ISL_17610256, EPI_ISL_17610257, EPI_ISL_17610258, EPI_ISL_17610259, EPI_ISL_17610260, EPI_ISL_17610261, EPI_ISL_17610262, EPI_ISL_17610263, EPI_ISL_17610264, EPI_ISL_17610265, EPI_ISL_17610266, EPI_ISL_17610267, EPI_ISL_17610268, EPI_ISL_17610269, EPI_ISL_17610270, EPI_ISL_17610271, EPI_ISL_17610272, EPI_ISL_17610273, EPI_ISL_17610274, EPI_ISL_17610275, EPI_ISL_17610276, EPI_ISL_17610277, EPI_ISL_17610278, EPI_ISL_17610279, EPI_ISL_17610280, EPI_ISL_17610281, EPI_ISL_17610282, EPI_ISL_17610283, EPI_ISL_17610284, EPI_ISL_17610285, EPI_ISL_17610286, EPI_ISL_17610287, EPI_ISL_17610288, EPI_ISL_17610289, EPI_ISL_17610290, EPI_ISL_17610291, EPI_ISL_17610292, EPI_ISL_17610293, EPI_ISL_17610294, EPI_ISL_17610295, EPI_ISL_17610296, EPI_ISL_17610297, EPI_ISL_17610298, EPI_ISL_17610299, EPI_ISL_17610300, EPI_ISL_17610301, EPI_ISL_17610302, EPI_ISL_17610303, EPI_ISL_17610304, EPI_ISL_17610305, EPI_ISL_17610306, EPI_ISL_17610307, EPI_ISL_17610308, EPI_ISL_17610309, EPI_ISL_17610310, EPI_ISL_17610311, EPI_ISL_17610312, EPI_ISL_17610313, EPI_ISL_17610314, EPI_ISL_17610315, EPI_ISL_17610316, EPI_ISL_17610317, EPI_ISL_17610318, EPI_ISL_17610319, EPI_ISL_17610320, EPI_ISL_17610321, EPI_ISL_17610322, EPI_ISL_17610323, EPI_ISL_17610324, EPI_ISL_17610325, EPI_ISL_17610326, EPI_ISL_17610327, EPI_ISL_17610328, EPI_ISL_17610329, EPI_ISL_17610330, EPI_ISL_17610331, EPI_ISL_17610332, EPI_ISL_17610333, EPI_ISL_17610334, EPI_ISL_17610335, EPI_ISL_17610336, EPI_ISL_17610337, EPI_ISL_17610338, EPI_ISL_17610339, EPI_ISL_17610340, EPI_ISL_17610341, EPI_ISL_17610342, EPI_ISL_17610343, EPI_ISL_17610344, EPI_ISL_17610345, EPI_ISL_17610346, EPI_ISL_17610347, EPI_ISL_17610348, EPI_ISL_17610349, EPI_ISL_17610350, EPI_ISL_17610351, EPI_ISL_17610352, EPI_ISL_17610353, EPI_ISL_17610354, EPI_ISL_17610355, EPI_ISL_17610356, EPI_ISL_17610357, EPI_ISL_17610358, EPI_ISL_17610359, EPI_ISL_17610360, EPI_ISL_17610361, EPI_ISL_17610362, EPI_ISL_17610363, EPI_ISL_17610364, EPI_ISL_17610365, EPI_ISL_17610366, EPI_ISL_17610367, EPI_ISL_17610368, EPI_ISL_17610369, EPI_ISL_17610370, EPI_ISL_17610371, EPI_ISL_17610372, EPI_ISL_17610373, EPI_ISL_17610374, EPI_ISL_17610375, EPI_ISL_17610376, EPI_ISL_17610377, EPI_ISL_17610378, EPI_ISL_17610379, EPI_ISL_17610380, EPI_ISL_17610381, EPI_ISL_17610382, EPI_ISL_17610383, EPI_ISL_17610384, EPI_ISL_17610385, EPI_ISL_17610386, EPI_ISL_17610411, EPI_ISL_17610412, EPI_ISL_17610413, EPI_ISL_17610414, EPI_ISL_17610415, EPI_ISL_17610416, EPI_ISL_17610417, EPI_ISL_17610418, EPI_ISL_17610419, EPI_ISL_17610420, EPI_ISL_17610421, EPI_ISL_17610422, EPI_ISL_17610423, EPI_ISL_17610424, EPI_ISL_17610425, EPI_ISL_17610426, EPI_ISL_17610427, EPI_ISL_17610428, EPI_ISL_17610429, EPI_ISL_17610430, EPI_ISL_17610431, EPI_ISL_17610432, EPI_ISL_17610433, EPI_ISL_17610434, EPI_ISL_17610435, EPI_ISL_17610436, EPI_ISL_17610437, EPI_ISL_17610438, EPI_ISL_17610439, EPI_ISL_17610440, EPI_ISL_17610441, EPI_ISL_17610442, EPI_ISL_17610443, EPI_ISL_17610444, EPI_ISL_17610445, EPI_ISL_17610446, EPI_ISL_17610447, EPI_ISL_17610448, EPI_ISL_17610449, EPI_ISL_17610450, EPI_ISL_17610451, EPI_ISL_17610452, EPI_ISL_17610453, EPI_ISL_17610454			
see above	London School of Hygiene and Tropical Medicine	London School of Hygiene and Tropical Medicine	Ashall,J., Shah,S., Biggs,J.R., Chang,R., Jafari,Y., Brady,O.J., Mai,H.K., Lien,L.T., Thai,H.D., Nhuyen,H.A.T., Anh,D., Iwasaki,C., Kitamura,N., Van Loock,M., Herrera-Taracena,G., Rasschaert,F., Van Wesenbeeck,L., Yoshida,L.-M., Hafalla,J.C.R., Hue,S. and Hibberd,M.L.
EPI_ISL_17610808, EPI_ISL_17610809, EPI_ISL_17610810, EPI_ISL_17610811, EPI_ISL_17610812, EPI_ISL_17610813, EPI_ISL_17610814, EPI_ISL_17610815, EPI_ISL_17610816, EPI_ISL_17610817, EPI_ISL_17610818, EPI_ISL_17610819, EPI_ISL_17610820, EPI_ISL_17610821, EPI_ISL_17610822, EPI_ISL_17610823, EPI_ISL_17610824			
see above	Sapporo Medical University, School of Medicine, Department of Hygiene	Sapporo Medical University, School of Medicine, Department of Hygiene	Jahan,A., Shyamal,K., Aung,M.S. and Kobayashi,N.
EPI_ISL_17611178, EPI_ISL_17611179, EPI_ISL_17611180, EPI_ISL_17611181, EPI_ISL_17611182, EPI_ISL_17611183	University of Nebraska Medical Center, Department of Environmental, Agricultural and Occupational Health	University of Nebraska Medical Center, Department of Environmental, Agricultural and Occupational Health	Agbodzi,B., Yousseu,F.B.S., Simo,F.B.N., Kumordjie,S., Yeboah,C., Mosore,M.-T., Bentil,R.E., Attram,N., Nimo-Paintsil,S., Fox,A.T., Bonney,J.H.K., Ampofo,W., Sanders,T., Wiley,M.R., Demanou,M. and Letizia,A.G.
EPI_ISL_17611185	Uppsala University, Zoonosis Science Center	Uppsala University, Zoonosis Science Center	Nguyen-Tien,T., Ling,J. and Lindalh,J.
EPI_ISL_17611186	Virus Laboratory, Indian Council of Medical Research, National Institute of Cholera and Enteric Diseases	Virus Laboratory, Indian Council of Medical Research, National Institute of Cholera and Enteric Diseases	Baskey,U., Verma,P., Ghosh,A. and Sadhukhan,P.C.
EPI_ISL_17658722	Yanbian University	Yanbian University	Li,C.
EPI_ISL_17658723, EPI_ISL_17658724, EPI_ISL_17658725, EPI_ISL_17658726, EPI_ISL_17658727, EPI_ISL_17658728	Department of Epidemiology of Microbial Diseases, Yale School of Public Health	Department of Epidemiology of Microbial Diseases, Yale School of Public Health	Vogels,C.B.F., Breban,M.I., Chaguza,C., Hill,V., Paul,L., Michael,S., Bunch,S., Cano,N., Jaber,R., Morrison,A., Panzera,C., Stryker,I., Vergara,J., Zimler,R., Kopp,E., Herberlein,L. and Grubaugh,N.D.
EPI_ISL_17658729, EPI_ISL_17658730, EPI_ISL_17658731, EPI_ISL_17658732, EPI_ISL_17658733, EPI_ISL_17658734, EPI_ISL_17658735, EPI_ISL_17658736, EPI_ISL_17658737, EPI_ISL_17658738, EPI_ISL_17658739, EPI_ISL_17658740, EPI_ISL_17658741			
see above	Institute of Medical Biology, Chinese Academy of Medical Sciences, Peking Union Medical College, Tsinghua University	Institute of Medical Biology, Chinese Academy of Medical Sciences, Peking Union Medical College, Tsinghua University	Zhou,Y., Zhou,Y., Liyang,S., Wang,P., An,J. and Liu,H.
EPI_ISL_17658742	National Institutes of Health, National Center for Biotechnology Information	National Institutes of Health, National Center for Biotechnology Information	Osatomi,K. and Sumiyoshi,H.
EPI_ISL_17658743	National Institutes of Health, National Center for Biotechnology Information	National Institutes of Health, National Center for Biotechnology Information	Durbin,A.P., Karron,R.A., Sun,W., Vaughn,D.W., Reynolds,M.J., Perreault,J.R., Thumar,B., Men,R., Lai,C.J., Elkins,W.R., Chanock,R.M., Murphy,B.R. and Whitehead,S.S.
EPI_ISL_17658986	Virology Unit, Institut Pasteur de Dakar	Virology Unit, Institut Pasteur de Dakar	Lagare,A., Faye,M., Fintan,G., Fall,G., Ousmane,H., Ibrahim,E.T., Diagne,M.M., Amadou,S., Sankhe,S., Ibrahim,L., Seini,H., Faye,O. and Jambou,R.
EPI_ISL_17658987	Department of Microbiology, Calcutta School of Tropical Medicine	Department of Microbiology, Calcutta School of Tropical Medicine	Tripathi,A. and Mukherjee,S.
EPI_ISL_17658988	Virology Unit, Institut Pasteur de Dakar	Virology Unit, Institut Pasteur de Dakar	Dieng,I.
EPI_ISL_17658989	Department of Microbiology, King George's Medical University	Department of Microbiology, King George's Medical University	Mallikarjun,K., Santhosh,K.S. and Puneeth,T.G.
EPI_ISL_17673145, EPI_ISL_17673146, EPI_ISL_17673147, EPI_ISL_17673148, EPI_ISL_17673149, EPI_ISL_17673150	Instituto Adolfo Lutz - Strategic Laboratory	Instituto Adolfo Lutz - Strategic Laboratory	Claudio Tavares Sacchi, Karoline Rodrigues Campos, Marlon Benedito Nascimento Santos, Juliana Silva Nogueira
EPI_ISL_17673494	Vector-borne Diseases Laboratory, Instituto Adolfo Lutz	Vector-borne Diseases Laboratory, Instituto Adolfo Lutz	Cunha,M.S., de Moura Coletti,T., Guerra,J.M., Ponce,C.C., Fernandes,N.C.C.A., Resio,R.A., Claro,I.M., Salles,F., Lima Neto,D.F. and Sabino,E.
EPI_ISL_17673495	Instituto Adolfo Lutz - Strategic Laboratory	Instituto Adolfo Lutz - Strategic Laboratory	Sacchi,C.T., Campos,K.R., Santos,M.B., Reis,A.D., Nogueira,J.S. and Maeda,A.Y.
EPI_ISL_17673496, EPI_ISL_17673497, EPI_ISL_17673498, EPI_ISL_17673499, EPI_ISL_17673500, EPI_ISL_17673501, EPI_ISL_17673502, EPI_ISL_17673503, EPI_ISL_17673504, EPI_ISL_17673505, EPI_ISL_17673506, EPI_ISL_17673507, EPI_ISL_17673508, EPI_ISL_17673509, EPI_ISL_17673510, EPI_ISL_17673511, EPI_ISL_17673512, EPI_ISL_17673513, EPI_ISL_17673514, EPI_ISL_17673515, EPI_ISL_17673516, EPI_ISL_17673517, EPI_ISL_17673518, EPI_ISL_17673519, EPI_ISL_17673520, EPI_ISL_17673521, EPI_ISL_17673522, EPI_ISL_17673523, EPI_ISL_17673524, EPI_ISL_17673525, EPI_ISL_17673526, EPI_ISL_17673527, EPI_ISL_17673528, EPI_ISL_17673529, EPI_ISL_17673530, EPI_ISL_17673531, EPI_ISL_17673532, EPI_ISL_17673533, EPI_ISL_17673534, EPI_ISL_17673535, EPI_ISL_17673536			
see above	Instituto Adolfo Lutz - Strategic Laboratory	Instituto Adolfo Lutz - Strategic Laboratory	Claudio Tavares Sacchi, Karoline Rodrigues Campos, Marlon Benedito Nascimento Santos, Juliana Silva Nogueira
EPI_ISL_17689847	LESP Chiapas	Instituto de Diagnostico y Referencia Epidemiologicos (INDRE)	Claudia Wong-Arámbula; Abril Rodríguez-Maldonado; Silvia Rivero-Arredondo; Ruth Madera-Sandoval; Joaquín Quiroz-Mercado; Daniel Duran-Ayala; Luz Torres Rodríguez; Claudia Rosales-Jiménez; Carlos Fuentes-Cuevas; Ernesto Ramírez-González; Fernando González-Domínguez; Lucia Hernández-Rivas, Irma López-Martínez; Mauricio Vázquez-Pichardo.
EPI_ISL_17689848	LESP Chihuahua	Instituto de Diagnostico y Referencia Epidemiologicos (INDRE)	Claudia Wong-Arámbula; Abril Rodríguez-Maldonado; Silvia Rivero-Arredondo; Ruth Madera-Sandoval; Joaquín Quiroz-Mercado; Daniel Duran-Ayala; Luz Torres Rodríguez; Claudia Rosales-Jiménez; Carlos Fuentes-Cuevas; Ernesto Ramírez-González; Fernando González-Domínguez; Lucia Hernández-Rivas, Irma López-Martínez; Mauricio Vázquez-Pichardo.
EPI_ISL_17689849	LESP Sinaloa	Instituto de Diagnostico y Referencia Epidemiologicos (INDRE)	Claudia Wong-Arámbula; Abril Rodríguez-Maldonado; Silvia Rivero-Arredondo; Ruth Madera-Sandoval; Joaquín Quiroz-Mercado; Daniel Duran-Ayala; Luz Torres Rodríguez; Claudia Rosales-Jiménez; Carlos Fuentes-Cuevas; Ernesto Ramírez-González; Fernando González-Domínguez; Lucia Hernández-Rivas, Irma López-Martínez; Mauricio Vázquez-Pichardo.
EPI_ISL_17689850	LESP Sonora	Instituto de Diagnostico y Referencia Epidemiologicos (INDRE)	Claudia Wong-Arámbula; Abril Rodríguez-Maldonado; Silvia Rivero-Arredondo; Ruth Madera-Sandoval; Joaquín Quiroz-Mercado; Daniel Duran-Ayala; Luz Torres Rodríguez; Claudia Rosales-Jiménez; Carlos Fuentes-Cuevas; Ernesto Ramírez-González; Fernando González-Domínguez; Lucia Hernández-Rivas, Irma López-Martínez; Mauricio Vázquez-Pichardo.

EPI_ISL_17689851	LESP Puebla	Instituto de Diagnostico y Referencia Epidemiologicos (INDRE)	Claudia Wong-Arámbula; Abril Rodríguez-Maldonado; Silvia Rivero-Arredondo; Ruth Madera-Sandoval; Joaquín Quiroz-Mercado; Daniel Duran-Ayala; Luz Torres Rodríguez; Claudia Rosales-Jiménez; Carlos Fuentes-Cuevas; Ernesto Ramírez-González; Fernando González-Domínguez; Lucia Hernández-Rivas, Irma López-Martínez; Mauricio Vázquez-Pichardo.
EPI_ISL_17689852, EPI_ISL_17689853	LESP State of Mexico	Instituto de Diagnostico y Referencia Epidemiologicos (INDRE)	Claudia Wong-Arámbula; Abril Rodríguez-Maldonado; Silvia Rivero-Arredondo; Ruth Madera-Sandoval; Joaquín Quiroz-Mercado; Daniel Duran-Ayala; Luz Torres Rodríguez; Claudia Rosales-Jiménez; Carlos Fuentes-Cuevas; Ernesto Ramírez-González; Fernando González-Domínguez; Lucia Hernández-Rivas, Irma López-Martínez; Mauricio Vázquez-Pichardo.
EPI_ISL_17689854, EPI_ISL_17689855, EPI_ISL_17689856	LESP Baja California	Instituto de Diagnostico y Referencia Epidemiologicos (INDRE)	Claudia Wong-Arámbula; Abril Rodríguez-Maldonado; Silvia Rivero-Arredondo; Ruth Madera-Sandoval; Joaquín Quiroz-Mercado; Daniel Duran-Ayala; Luz Torres Rodríguez; Claudia Rosales-Jiménez; Carlos Fuentes-Cuevas; Ernesto Ramírez-González; Fernando González-Domínguez; Lucia Hernández-Rivas, Irma López-Martínez; Mauricio Vázquez-Pichardo.
EPI_ISL_17689857, EPI_ISL_17689858	LESP Sinaloa	Instituto de Diagnostico y Referencia Epidemiologicos (INDRE)	Claudia Wong-Arámbula; Abril Rodríguez-Maldonado; Silvia Rivero-Arredondo; Ruth Madera-Sandoval; Joaquín Quiroz-Mercado; Daniel Duran-Ayala; Luz Torres Rodríguez; Claudia Rosales-Jiménez; Carlos Fuentes-Cuevas; Ernesto Ramírez-González; Fernando González-Domínguez; Lucia Hernández-Rivas, Irma López-Martínez; Mauricio Vázquez-Pichardo.
EPI_ISL_17689859	LESP State of Mexico	Instituto de Diagnostico y Referencia Epidemiologicos (INDRE)	Claudia Wong-Arámbula; Abril Rodríguez-Maldonado; Silvia Rivero-Arredondo; Ruth Madera-Sandoval; Joaquín Quiroz-Mercado; Daniel Duran-Ayala; Luz Torres Rodríguez; Claudia Rosales-Jiménez; Carlos Fuentes-Cuevas; Ernesto Ramírez-González; Fernando González-Domínguez; Lucia Hernández-Rivas, Irma López-Martínez; Mauricio Vázquez-Pichardo.
EPI_ISL_17689860	LESP Puebla	Instituto de Diagnostico y Referencia Epidemiologicos (INDRE)	Claudia Wong-Arámbula; Abril Rodríguez-Maldonado; Silvia Rivero-Arredondo; Ruth Madera-Sandoval; Joaquín Quiroz-Mercado; Daniel Duran-Ayala; Luz Torres Rodríguez; Claudia Rosales-Jiménez; Carlos Fuentes-Cuevas; Ernesto Ramírez-González; Fernando González-Domínguez; Lucia Hernández-Rivas, Irma López-Martínez; Mauricio Vázquez-Pichardo.
EPI_ISL_17689861, EPI_ISL_17689862	LESP Veracruz	Instituto de Diagnostico y Referencia Epidemiologicos (INDRE)	Claudia Wong-Arámbula; Abril Rodríguez-Maldonado; Silvia Rivero-Arredondo; Ruth Madera-Sandoval; Joaquín Quiroz-Mercado; Daniel Duran-Ayala; Luz Torres Rodríguez; Claudia Rosales-Jiménez; Carlos Fuentes-Cuevas; Ernesto Ramírez-González; Fernando González-Domínguez; Lucia Hernández-Rivas, Irma López-Martínez; Mauricio Vázquez-Pichardo.
EPI_ISL_17689863	LESP Sonora	Instituto de Diagnostico y Referencia Epidemiologicos (INDRE)	Claudia Wong-Arámbula; Abril Rodríguez-Maldonado; Silvia Rivero-Arredondo; Ruth Madera-Sandoval; Joaquín Quiroz-Mercado; Daniel Duran-Ayala; Luz Torres Rodríguez; Claudia Rosales-Jiménez; Carlos Fuentes-Cuevas; Ernesto Ramírez-González; Fernando González-Domínguez; Lucia Hernández-Rivas, Irma López-Martínez; Mauricio Vázquez-Pichardo.
EPI_ISL_17689864	LESP Yucatan	Instituto de Diagnostico y Referencia Epidemiologicos (INDRE)	Claudia Wong-Arámbula; Abril Rodríguez-Maldonado; Silvia Rivero-Arredondo; Ruth Madera-Sandoval; Joaquín Quiroz-Mercado; Daniel Duran-Ayala; Luz Torres Rodríguez; Claudia Rosales-Jiménez; Carlos Fuentes-Cuevas; Ernesto Ramírez-González; Fernando González-Domínguez; Lucia Hernández-Rivas, Irma López-Martínez; Mauricio Vázquez-Pichardo.
EPI_ISL_17689865	LESP Coahuila	Instituto de Diagnostico y Referencia Epidemiologicos (INDRE)	Claudia Wong-Arámbula; Abril Rodríguez-Maldonado; Silvia Rivero-Arredondo; Ruth Madera-Sandoval; Joaquín Quiroz-Mercado; Daniel Duran-Ayala; Luz Torres Rodríguez; Claudia Rosales-Jiménez; Carlos Fuentes-Cuevas; Ernesto Ramírez-González; Fernando González-Domínguez; Lucia Hernández-Rivas, Irma López-Martínez; Mauricio Vázquez-Pichardo.
EPI_ISL_17689866	LESP Veracruz	Instituto de Diagnostico y Referencia Epidemiologicos (INDRE)	Claudia Wong-Arámbula; Abril Rodríguez-Maldonado; Silvia Rivero-Arredondo; Ruth Madera-Sandoval; Joaquín Quiroz-Mercado; Daniel Duran-Ayala; Luz Torres Rodríguez; Claudia Rosales-Jiménez; Carlos Fuentes-Cuevas; Ernesto Ramírez-González; Fernando González-Domínguez; Lucia Hernández-Rivas, Irma López-Martínez; Mauricio Vázquez-Pichardo.
EPI_ISL_17689867, EPI_ISL_17689868	LESP Coahuila	Instituto de Diagnostico y Referencia Epidemiologicos (INDRE)	Claudia Wong-Arámbula; Abril Rodríguez-Maldonado; Silvia Rivero-Arredondo; Ruth Madera-Sandoval; Joaquín Quiroz-Mercado; Daniel Duran-Ayala; Luz Torres Rodríguez; Claudia Rosales-Jiménez; Carlos Fuentes-Cuevas; Ernesto Ramírez-González; Fernando González-Domínguez; Lucia Hernández-Rivas, Irma López-Martínez; Mauricio Vázquez-Pichardo.
EPI_ISL_17689869	LESP Veracruz	Instituto de Diagnostico y Referencia Epidemiologicos (INDRE)	Claudia Wong-Arámbula; Abril Rodríguez-Maldonado; Silvia Rivero-Arredondo; Ruth Madera-Sandoval; Joaquín Quiroz-Mercado; Daniel Duran-Ayala; Luz Torres Rodríguez; Claudia Rosales-Jiménez; Carlos Fuentes-Cuevas; Ernesto Ramírez-González; Fernando González-Domínguez; Lucia Hernández-Rivas, Irma López-Martínez; Mauricio Vázquez-Pichardo.
EPI_ISL_17689870	LESP Tabasco	Instituto de Diagnostico y Referencia Epidemiologicos (INDRE)	Claudia Wong-Arámbula; Abril Rodríguez-Maldonado; Silvia Rivero-Arredondo; Ruth Madera-Sandoval; Joaquín Quiroz-Mercado; Daniel Duran-Ayala; Luz Torres Rodríguez; Claudia Rosales-Jiménez; Carlos Fuentes-Cuevas; Ernesto Ramírez-González; Fernando González-Domínguez; Lucia Hernández-Rivas, Irma López-Martínez; Mauricio Vázquez-Pichardo.
EPI_ISL_17689871, EPI_ISL_17689872, EPI_ISL_17689873	LESP Quintana Roo	Instituto de Diagnostico y Referencia Epidemiologicos (INDRE)	Claudia Wong-Arámbula; Abril Rodríguez-Maldonado; Silvia Rivero-Arredondo; Ruth Madera-Sandoval; Joaquín Quiroz-Mercado; Daniel Duran-Ayala; Luz Torres Rodríguez; Claudia Rosales-Jiménez; Carlos Fuentes-Cuevas; Ernesto Ramírez-González; Fernando González-Domínguez; Lucia Hernández-Rivas, Irma López-Martínez; Mauricio Vázquez-Pichardo.
EPI_ISL_17689874, EPI_ISL_17689875, EPI_ISL_17689876	LESP Colima	Instituto de Diagnostico y Referencia Epidemiologicos (INDRE)	Claudia Wong-Arámbula; Abril Rodríguez-Maldonado; Silvia Rivero-Arredondo; Ruth Madera-Sandoval; Joaquín Quiroz-Mercado; Daniel Duran-Ayala; Luz Torres Rodríguez; Claudia Rosales-Jiménez; Carlos Fuentes-Cuevas; Ernesto Ramírez-González; Fernando González-Domínguez; Lucia Hernández-Rivas, Irma López-Martínez; Mauricio Vázquez-Pichardo.
EPI_ISL_17689877, EPI_ISL_17689878	LESP Tabasco	Instituto de Diagnostico y Referencia Epidemiologicos (INDRE)	Claudia Wong-Arámbula; Abril Rodríguez-Maldonado; Silvia Rivero-Arredondo; Ruth Madera-Sandoval; Joaquín Quiroz-Mercado; Daniel Duran-Ayala; Luz Torres Rodríguez; Claudia Rosales-Jiménez; Carlos Fuentes-Cuevas; Ernesto Ramírez-González; Fernando González-Domínguez; Lucia Hernández-Rivas, Irma López-Martínez; Mauricio Vázquez-Pichardo.
EPI_ISL_17694637, EPI_ISL_17694638	Department of Microbiology, Calcutta School of Tropical Medicine	Department of Microbiology, Calcutta School of Tropical Medicine	Tripathi,A. and Mukherjee,S.
EPI_ISL_17694639, EPI_ISL_17694640, EPI_ISL_17694641, EPI_ISL_17694642, EPI_ISL_17694643, EPI_ISL_17694644	Institute of Medical Biology, Chinese Academy of Medical Sciences, Peking Union Medical College, Tsinghua University	Institute of Medical Biology, Chinese Academy of Medical Sciences, Peking Union Medical College, Tsinghua University	Zhou,Y., Zhou,Y., Liyang,S., Wang,P., An,J. and Liu,H.
EPI_ISL_17694645, EPI_ISL_17694646, EPI_ISL_17694647, EPI_ISL_17694648, EPI_ISL_17694649	Department of Microbiology, Calcutta School of Tropical Medicine	Department of Microbiology, Calcutta School of Tropical Medicine	Tripathi,A. and Mukherjee,S.
EPI_ISL_17699727	LACEN de Sergipe	Instituto Adolfo Lutz - Strategic Laboratory	Claudio Tavares Sacchi, Karoline Rodrigues Campos, Marlon Benedito Nascimento Santos, Gabriela Vasconcelos Brito Bezerra
EPI_ISL_17699728, EPI_ISL_17699729, EPI_ISL_17699730, EPI_ISL_17699731, EPI_ISL_17699732, EPI_ISL_17699733, EPI_ISL_17699734, EPI_ISL_17699735, EPI_ISL_17699736, EPI_ISL_17699737, EPI_ISL_17699738, EPI_ISL_17699739, EPI_ISL_17699740, EPI_ISL_17699741, EPI_ISL_17699742, EPI_ISL_17699743, EPI_ISL_17699744, EPI_ISL_17699745, EPI_ISL_17699746, EPI_ISL_17699747, EPI_ISL_17699748, EPI_ISL_17699749, EPI_ISL_17699750, EPI_ISL_17699751, EPI_ISL_17699752, EPI_ISL_17699753, EPI_ISL_17699754, EPI_ISL_17699755, EPI_ISL_17699756, EPI_ISL_17699757, EPI_ISL_17699758, EPI_ISL_17699759, EPI_ISL_17699760, EPI_ISL_17699761, EPI_ISL_17699762, EPI_ISL_17699763, EPI_ISL_17699764, EPI_ISL_17699766, EPI_ISL_17699767, EPI_ISL_17699768, EPI_ISL_17699769, EPI_ISL_17699770, EPI_ISL_17699771, EPI_ISL_17699772, EPI_ISL_17699773, EPI_ISL_17699774, EPI_ISL_17699775, EPI_ISL_17699776, EPI_ISL_17699777			
see above	Instituto Adolfo Lutz - Strategic Laboratory	Instituto Adolfo Lutz - Strategic Laboratory	Claudio Tavares Sacchi, Karoline Rodrigues Campos, Marlon Benedito Nascimento Santos, Juliana Silva Nogueira
EPI_ISL_17699778, EPI_ISL_17699779, EPI_ISL_17699780, EPI_ISL_17699781, EPI_ISL_17699782, EPI_ISL_17699783, EPI_ISL_17699784, EPI_ISL_17699785	LACEN de Santa Catarina	Instituto Adolfo Lutz - Strategic Laboratory	Claudio Tavares Sacchi, Karoline Rodrigues Campos, Marlon Benedito Nascimento Santos
EPI_ISL_17699786, EPI_ISL_17699787	LACEN de Sergipe	Instituto Adolfo Lutz - Strategic Laboratory	Claudio Tavares Sacchi, Karoline Rodrigues Campos, Marlon Benedito Nascimento Santos, Gabriela Vasconcelos Brito Bezerra
EPI_ISL_17699788, EPI_ISL_17699789, EPI_ISL_17699796, EPI_ISL_17699797, EPI_ISL_17699798, EPI_ISL_17699799, EPI_ISL_17699800, EPI_ISL_17699801, EPI_ISL_17699802, EPI_ISL_17699803, EPI_ISL_17699804, EPI_ISL_17699805, EPI_ISL_17699806, EPI_ISL_17699807, EPI_ISL_17699808, EPI_ISL_17699809, EPI_ISL_17699810, EPI_ISL_17699811, EPI_ISL_17699812, EPI_ISL_17699813, EPI_ISL_17699814, EPI_ISL_17699815, EPI_ISL_17699816, EPI_ISL_17699817			
see above	LACEN do Rio de Janeiro	Instituto Adolfo Lutz - Strategic Laboratory	Claudio Tavares Sacchi, Karoline Rodrigues Campos, Marlon Benedito Nascimento Santos, Leandro Magalhaes de Souza
EPI_ISL_17733558, EPI_ISL_17733559, EPI_ISL_17733560, EPI_ISL_17733561, EPI_ISL_17733562, EPI_ISL_17733563, EPI_ISL_17733565, EPI_ISL_17733566, EPI_ISL_17733567, EPI_ISL_17733568, EPI_ISL_17733569, EPI_ISL_17733570, EPI_ISL_17733571, EPI_ISL_17733572, EPI_ISL_17733573, EPI_ISL_17733574, EPI_ISL_17733575, EPI_ISL_17733576, EPI_ISL_17733577, EPI_ISL_17733578, EPI_ISL_17733580, EPI_ISL_17733581, EPI_ISL_17733582, EPI_ISL_17733583, EPI_ISL_17733584, EPI_ISL_17733585, EPI_ISL_17733586, EPI_ISL_17733587, EPI_ISL_17733588, EPI_ISL_17733589, EPI_ISL_17733590, EPI_ISL_17733591, EPI_ISL_17733592, EPI_ISL_17733594, EPI_ISL_17733595, EPI_ISL_17733596, EPI_ISL_17733597, EPI_ISL_17733598, EPI_ISL_17733599, EPI_ISL_17733600, EPI_ISL_17733601, EPI_ISL_17733602, EPI_ISL_17733603, EPI_ISL_17733604, EPI_ISL_17733605, EPI_ISL_17733606, EPI_ISL_17733608, EPI_ISL_17733609, EPI_ISL_17733610, EPI_ISL_17733611, EPI_ISL_17733612, EPI_ISL_17733613, EPI_ISL_17733614, EPI_ISL_17733615, EPI_ISL_17733616, EPI_ISL_17733617, EPI_ISL_17733618, EPI_ISL_17733619, EPI_ISL_17733620, EPI_ISL_17733621, EPI_ISL_17733623, EPI_ISL_17733624, EPI_ISL_17733625, EPI_ISL_17733626, EPI_ISL_17733627, EPI_ISL_17733628, EPI_ISL_17733629, EPI_ISL_17733630, EPI_ISL_17733631, EPI_ISL_17733632, EPI_ISL_17733633, EPI_ISL_17733634, EPI_ISL_17733635, EPI_ISL_17733637, EPI_ISL_17733638, EPI_ISL_17733639, EPI_ISL_17733640, EPI_ISL_17733641, EPI_ISL_17733642, EPI_ISL_17733643, EPI_ISL_17733644, EPI_ISL_17733645, EPI_ISL_17733646, EPI_ISL_17733647, EPI_ISL_17733648, EPI_ISL_17733649, EPI_ISL_17733650, EPI_ISL_17733651, EPI_ISL_17733652, EPI_ISL_17733653, EPI_ISL_17733654, EPI_ISL_17733655, EPI_ISL_17733656, EPI_ISL_17733657, EPI_ISL_17733658, EPI_ISL_17733659, EPI_ISL_17733660, EPI_ISL_17733661, EPI_ISL_17733662, EPI_ISL_17733663, EPI_ISL_17733664, EPI_ISL_17733665, EPI_ISL_17733666, EPI_ISL_17733667, EPI_ISL_17733668, EPI_ISL_17733669, EPI_ISL_17733670, EPI_ISL_17733671, EPI_ISL_17733672, EPI_ISL_17733673, EPI_ISL_17733674, EPI_ISL_17733675, EPI_ISL_17733676, EPI_ISL_17733677, EPI_ISL_17733678, EPI_ISL_17733679, EPI_ISL_17733680, EPI_ISL_17733681, EPI_ISL_17733682, EPI_ISL_17733683, EPI_ISL_17733684, EPI_ISL_17733685, EPI_ISL_17733686, EPI_ISL_17733687, EPI_ISL_17733688, EPI_ISL_17733689, EPI_ISL_17733690, EPI_ISL_17733691, EPI_ISL_17733692, EPI_ISL_17733693, EPI_ISL_17733694, EPI_ISL_17733695			
see above	Central Public Health Laboratory, State Health Surveillance Center of the Rio Grande do Sul State Health Department (LACEN/CEVS/SES-RS)	Center for Scientific and Technological Development, State Center for Health Surveillance of the Secretary of Health of the State of Rio Grande do Sul (CDCT/CEVS/SES-RS)	Fernanda Godinho, Tatiana Schäffer Gregianini, Amanda Pellenz Ruivo, Júlio Augusto Schroder, Viviane Horn, Franciellen Machado dos Santos, Regina Bones Barcellos, Juliano de Oliveira Silveira, Fernanda Letícia Martiny, Érica Bortoli Möllmann, Gabriel da Luz Wallau, Richard Steiner Salvato
EPI_ISL_17733704, EPI_ISL_17733705, EPI_ISL_17733706, EPI_ISL_17733707, EPI_ISL_17733708, EPI_ISL_17733709, EPI_ISL_17733710, EPI_ISL_17733711, EPI_ISL_17733712, EPI_ISL_17733713, EPI_ISL_17733714, EPI_ISL_17733715, EPI_ISL_17733716, EPI_ISL_17733717, EPI_ISL_17733718, EPI_ISL_17733719, EPI_ISL_17733720, EPI_ISL_17733721, EPI_ISL_17733722, EPI_ISL_17733723, EPI_ISL_17733724, EPI_ISL_17733725, EPI_ISL_17733726, EPI_ISL_17733727, EPI_ISL_17733728, EPI_ISL_17733729, EPI_ISL_17733730, EPI_ISL_17733731, EPI_ISL_17733732, EPI_ISL_17733733, EPI_ISL_17733734, EPI_ISL_17733735, EPI_ISL_17733736, EPI_ISL_17733737, EPI_ISL_17733738, EPI_ISL_17733739, EPI_ISL_17733740, EPI_ISL_17733741, EPI_ISL_17733742, EPI_ISL_17733743, EPI_ISL_17733744, EPI_ISL_17733745, EPI_ISL_17733746, EPI_ISL_17733747, EPI_ISL_17733748, EPI_ISL_17733749, EPI_ISL_17733750, EPI_ISL_17733751, EPI_ISL_17733752, EPI_ISL_17733753, EPI_ISL_17733754, EPI_ISL_17733755, EPI_ISL_17733756, EPI_ISL_17733757, EPI_ISL_17733758, EPI_ISL_17733759, EPI_ISL_17733760, EPI_ISL_17733761, EPI_ISL_17733762, EPI_ISL_17733763, EPI_ISL_17733764, EPI_ISL_17733765, EPI_ISL_17733766, EPI_ISL_17733767, EPI_ISL_17733768, EPI_ISL_17733769, EPI_ISL_17733770, EPI_ISL_17733771, EPI_ISL_17733772, EPI_ISL_17733773, EPI_ISL_17733774, EPI_ISL_17733775, EPI_ISL_17733776, EPI_ISL_17733777, EPI_ISL_17733778, EPI_ISL_17733779, EPI_ISL_17733780, EPI_ISL_17733781, EPI_ISL_17733782, EPI_ISL_17733783, EPI_ISL_17733784, EPI_ISL_17733785, EPI_ISL_17733786, EPI_ISL_17733787, EPI_ISL_17733788, EPI_ISL_17733789, EPI_ISL_17733790, EPI_ISL_17733791, EPI_ISL_17733792, EPI_ISL_17733793, EPI_ISL_17733794, EPI_ISL_17733795, EPI_ISL_17733796, EPI_ISL_17733797, EPI_ISL_17733798, EPI_ISL_17733799, EPI_ISL_17733800, EPI_ISL_17733801, EPI_ISL_17733802, EPI_ISL_17733803, EPI_ISL_17733804, EPI_ISL_17733805, EPI_ISL_17733806, EPI_ISL_17733807, EPI_ISL_17733808, EPI_ISL_17733809, EPI_ISL_17733810, EPI_ISL_17733811, EPI_ISL_17733812, EPI_ISL_17733813, EPI_ISL_17733814, EPI_ISL_17733815, EPI_ISL_17733816, EPI_ISL_17733817, EPI_ISL_17733818, EPI_ISL_17733819, EPI_ISL_17733820, EPI_ISL_17733821, EPI_ISL_17733822, EPI_ISL_17733823, EPI_ISL_17733824, EPI_ISL_17733825, EPI_ISL_17733826, EPI_ISL_17733827, EPI_ISL_17733828, EPI_ISL_17733829, EPI_ISL_17733830, EPI_ISL_17733831, EPI_ISL_17733832, EPI_ISL_17733833, EPI_ISL_17733834, EPI_ISL_17733835, EPI_ISL_17733836, EPI_ISL_17733837, EPI_ISL_17733838, EPI_ISL_17733839, EPI_ISL_17733840, EPI_ISL_17733841, EPI_ISL_17733842, EPI_ISL_17733843, EPI_ISL_17733844, EPI_ISL_17733845, EPI_ISL_17733846, EPI_ISL_17733847, EPI_ISL_17733848, EPI_ISL_17733849, EPI_ISL_17733850, EPI_ISL_17733851, EPI_ISL_17733852, EPI_ISL_17733853, EPI_ISL_17733854, EPI_ISL_17733855, EPI_ISL_17733856, EPI_ISL_17733857, EPI_ISL_17733858, EPI_ISL_17733859, EPI_ISL_17733860, EPI_ISL_17733861, EPI_ISL_17733862, EPI_ISL_17733863, EPI_ISL_17733864, EPI_ISL_17733865, EPI_ISL_17733866, EPI_ISL_17733867, EPI_ISL_17733868, EPI_ISL_17733869, EPI_ISL_17733870, EPI_ISL_17733871, EPI_ISL_17733872, EPI_ISL_17733873, EPI_ISL_17733874, EPI_ISL_17733875, EPI_ISL_17733876, EPI_ISL_17733877, EPI_ISL_17733878, EPI_ISL_17733879, EPI_ISL_17733880, EPI_ISL_17733881, EPI_ISL_17733882, EPI_ISL_17733883, EPI_ISL_17733884, EPI_ISL_17733885, EPI_ISL_17733886, EPI_ISL_17733887, EPI_ISL_17733888, EPI_ISL_17733889, EPI_ISL_17733890, EPI_ISL_17733891, EPI_ISL_17733892, EPI_ISL_17733893, EPI_ISL_17733894, EPI_ISL_17733895, EPI_ISL_17733896, EPI_ISL_17733897, EPI_ISL_17733898, EPI_ISL_17733899, EPI_ISL_17733900, EPI_ISL_17733901, EPI_ISL_17733902, EPI_ISL_17733903, EPI_ISL_17733904, EPI_ISL_17733905, EPI_ISL_17733906, EPI_ISL_17733907, EPI_ISL_17733908, EPI_ISL_17733909, EPI_ISL_17733910, EPI_ISL_17733911, EPI_ISL_17733912, EPI_ISL_17733913, EPI_ISL_17733914, EPI_ISL_17733915, EPI_ISL_17733916, EPI_ISL_17733917, EPI_ISL_17733918, EPI_ISL_17733919, EPI_ISL_17733920, EPI_ISL_17733921, EPI_ISL_17733922, EPI_ISL_17733923, EPI_ISL_17733924, EPI_ISL_17733925, EPI_ISL_17733926, EPI_ISL_17733927, EPI_ISL_17733928, EPI_ISL_17733929, EPI_ISL_17733930, EPI_ISL_17733931, EPI_ISL_17733932, EPI_ISL_17733933, EPI_ISL_17733934, EPI_ISL_17733935, EPI_ISL_17733936, EPI_ISL_17733937, EPI_ISL_17733938, EPI_ISL_17733939, EPI_ISL_17733940, EPI_ISL_17733941, EPI_ISL_17733942, EPI_ISL_17733943, EPI_ISL_17733944, EPI_ISL_17733945, EPI_ISL_17733946, EPI_ISL_17733947, EPI_ISL_17733948, EPI_ISL_17733949, EPI_ISL_17733950, EPI_ISL_17733951, EPI_ISL_17733952, EPI_ISL_17733953, EPI_ISL_17733954, EPI_ISL_17733955			
see above	Guangdong Provincial Center for Disease Control and Prevention	Guangdong Provincial Center for Disease Control and Prevention	Sun,J.
EPI_ISL_17734039, EPI_ISL_17734040, EPI_ISL_17734041, EPI_ISL_17734045, EPI_ISL_17734046, EPI_ISL_17734047, EPI_ISL_17734048, EPI_ISL_17734049, EPI_ISL_17734050, EPI_ISL_17734051, EPI_ISL_17734052, EPI_ISL_17734053, EPI_ISL_17734054, EPI_ISL_17734055, EPI_ISL_17734056, EPI_ISL_17734057, EPI_ISL_17734058, EPI_ISL_17734059, EPI_ISL_17734060, EPI_ISL_17734061, EPI_ISL_17734062, EPI_ISL_17734063, EPI_ISL_17734064			
see above	Fundação Oswaldo Cruz, Instituto Carlos Chagas	Fundação Oswaldo Cruz, Instituto Carlos Chagas	Graf,T., Ferreira,C.N., Presibella,M., Fernandes,S.B. and Duarte dos Santos,C.N.
EPI_ISL_17744309	Laboratorio Central de Saude Publica "Dr. Milton Bezerra Sobral" LACEN PE	Fiocruz Pernambuco - Instituto Aggeu Magalhaes	Gustavo Barbosa de Lima, Raul Emídio de Lima, Lais Ceschini Machado, Tulio de Lima Campos, Gabriel Luz Wallau
EPI_ISL_17772334, EPI_ISL_17772335	Department of Microbiology, Calcutta School of Tropical Medicine	Department of Microbiology, Calcutta School of Tropical Medicine	Tripathi,A. and Mukherjee,S.
EPI_ISL_17783293, EPI_ISL_17783294, EPI_ISL_17783295, EPI_ISL_17783296, EPI_ISL_17783297, EPI_ISL_17783298, EPI_ISL_17783299, EPI_ISL_17783300, EPI_ISL_17783301, EPI_ISL_17783302, EPI_ISL_17783303, EPI_ISL_17783304, EPI_ISL_17783305, EPI_ISL_17783306, EPI_ISL_17783307, EPI_ISL_17783308, EPI_ISL_17783309, EPI_ISL_17783310, EPI_ISL_17783311, EPI_ISL_17783312, EPI_ISL_17783313, EPI_ISL_17783314, EPI_ISL_17783315, EPI_ISL_17783316, EPI_ISL_17783317, EPI_ISL_17783318, EPI_ISL_17783319, EPI_ISL_17783320, EPI_ISL_17783321, EPI_ISL_17783322, EPI_ISL_17783323, EPI_ISL_17783325, EPI_ISL_17783326, EPI_ISL_17783327, EPI_ISL_17783328, EPI_ISL_17783329, EPI_ISL_17783330, EPI_ISL_17783331, EPI_ISL_17783332, EPI_ISL_17783333, EPI_ISL_17783334, EPI_ISL_17783335, EPI_ISL_17783336			
see above	Grupo de Morfologia Celular, Instituto Nacional de Salud	Grupo de Morfologia Celular, Instituto Nacional de Salud	Laiton-Donato,K.D., Alvarez-Diaz,D.A., Rosales-Munar,A., Pelaez-Carvajal,D., Ajami,N.J. and Usme-Ciro,J.A.
EPI_ISL_17783337, EPI_ISL_17783338	Tokyo Metropolitan Institute of Public Health	Tokyo Metropolitan Institute of Public Health	Okada,W., Kasuya,F., Harada,S., Kumagai,R., Hasegawa,M., Miyake,H., Nagashima,M. and Sadamasu,K.

EPI_ISL_17783339, EPI_ISL_17783340, EPI_ISL_17783341, EPI_ISL_17783342, EPI_ISL_17783343, EPI_ISL_17783344, EPI_ISL_17783345, EPI_ISL_17783346, EPI_ISL_17783347, EPI_ISL_17783348, EPI_ISL_17783349, EPI_ISL_17783350, EPI_ISL_17783351, EPI_ISL_17783352, EPI_ISL_17783353			
see above	Grupo de Morfologia Celular, Instituto Nacional de Salud	Grupo de Morfologia Celular, Instituto Nacional de Salud	Laiton-Donato,K.D., Alvarez-Diaz,D.A., Rosales-Munar,A., Pelaez-Carvajal,D., Ajami,N.J. and Usme-Ciro,J.A.
EPI_ISL_17798172, EPI_ISL_17798173, EPI_ISL_17798174, EPI_ISL_17798175, EPI_ISL_17798176, EPI_ISL_17798177, EPI_ISL_17798178, EPI_ISL_17798179, EPI_ISL_17798180, EPI_ISL_17798181, EPI_ISL_17798182, EPI_ISL_17798183, EPI_ISL_17798184, EPI_ISL_17798185, EPI_ISL_17798186, EPI_ISL_17798187, EPI_ISL_17798188, EPI_ISL_17798189, EPI_ISL_17798190, EPI_ISL_17798191, EPI_ISL_17798192, EPI_ISL_17798193, EPI_ISL_17798194, EPI_ISL_17798195, EPI_ISL_17798196, EPI_ISL_17798197, EPI_ISL_17798198, EPI_ISL_17798199, EPI_ISL_17798200, EPI_ISL_17798201, EPI_ISL_17798202, EPI_ISL_17798203, EPI_ISL_17798204, EPI_ISL_17798205, EPI_ISL_17798206, EPI_ISL_17798207, EPI_ISL_17798208, EPI_ISL_17798209, EPI_ISL_17798210, EPI_ISL_17798211, EPI_ISL_17798212, EPI_ISL_17798213, EPI_ISL_17798214, EPI_ISL_17798215, EPI_ISL_17798216, EPI_ISL_17798217, EPI_ISL_17798218, EPI_ISL_17798219, EPI_ISL_17798220, EPI_ISL_17798221, EPI_ISL_17798222, EPI_ISL_17798223, EPI_ISL_17798224, EPI_ISL_17798225, EPI_ISL_17798226, EPI_ISL_17798227, EPI_ISL_17798228, EPI_ISL_17798229, EPI_ISL_17798230, EPI_ISL_17798231, EPI_ISL_17798232, EPI_ISL_17798233, EPI_ISL_17798234, EPI_ISL_17798235, EPI_ISL_17798236, EPI_ISL_17798237, EPI_ISL_17798238, EPI_ISL_17798239, EPI_ISL_17798240, EPI_ISL_17798241, EPI_ISL_17798242, EPI_ISL_17798243, EPI_ISL_17798244, EPI_ISL_17798245, EPI_ISL_17798246, EPI_ISL_17798247, EPI_ISL_17798248, EPI_ISL_17798249, EPI_ISL_17798250, EPI_ISL_17798251, EPI_ISL_17798252, EPI_ISL_17798253, EPI_ISL_17798254, EPI_ISL_17798255, EPI_ISL_17798256, EPI_ISL_17798257, EPI_ISL_17798258, EPI_ISL_17798259, EPI_ISL_17798260, EPI_ISL_17798261, EPI_ISL_17798262, EPI_ISL_17798263, EPI_ISL_17798264, EPI_ISL_17798265, EPI_ISL_17798266, EPI_ISL_17798267, EPI_ISL_17798268, EPI_ISL_17798269, EPI_ISL_17798270, EPI_ISL_17798271, EPI_ISL_17798272, EPI_ISL_17798273, EPI_ISL_17798274, EPI_ISL_17798275, EPI_ISL_17798276, EPI_ISL_17798277, EPI_ISL_17798278, EPI_ISL_17798279, EPI_ISL_17798280, EPI_ISL_17798281, EPI_ISL_17798282, EPI_ISL_17798283, EPI_ISL_17798284, EPI_ISL_17798285, EPI_ISL_17798286, EPI_ISL_17798287, EPI_ISL_17798288, EPI_ISL_17798289, EPI_ISL_17798290, EPI_ISL_17798291, EPI_ISL_17798292, EPI_ISL_17798293, EPI_ISL_17798294, EPI_ISL_17798295, EPI_ISL_17798296, EPI_ISL_17798297, EPI_ISL_17798298, EPI_ISL_17798299, EPI_ISL_17798300, EPI_ISL_17798301, EPI_ISL_17798302, EPI_ISL_17798303, EPI_ISL_17798304, EPI_ISL_17798305, EPI_ISL_17798306, EPI_ISL_17798307, EPI_ISL_17798308, EPI_ISL_17798309, EPI_ISL_17798310, EPI_ISL_17798311, EPI_ISL_17798312, EPI_ISL_17798313, EPI_ISL_17798314, EPI_ISL_17798315, EPI_ISL_17798316, EPI_ISL_17798317, EPI_ISL_17798318, EPI_ISL_17798319, EPI_ISL_17798320, EPI_ISL_17798321, EPI_ISL_17798322, EPI_ISL_17798323, EPI_ISL_17798324, EPI_ISL_17798325, EPI_ISL_17798326, EPI_ISL_17798327, EPI_ISL_17798328, EPI_ISL_17798329, EPI_ISL_17798330, EPI_ISL_17798331, EPI_ISL_17798332, EPI_ISL_17798333, EPI_ISL_17798334, EPI_ISL_17798335, EPI_ISL_17798336, EPI_ISL_17798337, EPI_ISL_17798338, EPI_ISL_17798339, EPI_ISL_17798340, EPI_ISL_17798341, EPI_ISL_17798342, EPI_ISL_17798343, EPI_ISL_17798344, EPI_ISL_17798345, EPI_ISL_17798346, EPI_ISL_17798347, EPI_ISL_17798348, EPI_ISL_17798349, EPI_ISL_17798350, EPI_ISL_17798351, EPI_ISL_17798352, EPI_ISL_17798353, EPI_ISL_17798354, EPI_ISL_17798355, EPI_ISL_17798356, EPI_ISL_17798357, EPI_ISL_17798358, EPI_ISL_17798359, EPI_ISL_17798360, EPI_ISL_17798361, EPI_ISL_17798362, EPI_ISL_17798363, EPI_ISL_17798364, EPI_ISL_17798365, EPI_ISL_17798366, EPI_ISL_17798414, EPI_ISL_17798435, EPI_ISL_17798436, EPI_ISL_17798437, EPI_ISL_17798438, EPI_ISL_17798439, EPI_ISL_17798440, EPI_ISL_17798441, EPI_ISL_17798442, EPI_ISL_17798443, EPI_ISL_17798444, EPI_ISL_17798445, EPI_ISL_17798446, EPI_ISL_17798447, EPI_ISL_17798448, EPI_ISL_17798449, EPI_ISL_17798450, EPI_ISL_17798451, EPI_ISL_17798452, EPI_ISL_17798453, EPI_ISL_17798454, EPI_ISL_17798455, EPI_ISL_17798456, EPI_ISL_17798457, EPI_ISL_17798458, EPI_ISL_17798459, EPI_ISL_17798460, EPI_ISL_17798461, EPI_ISL_17798462, EPI_ISL_17798463, EPI_ISL_17798464, EPI_ISL_17798465, EPI_ISL_17798466, EPI_ISL_17798467, EPI_ISL_17798468, EPI_ISL_17798469, EPI_ISL_17798470, EPI_ISL_17798471, EPI_ISL_17798472, EPI_ISL_17798473, EPI_ISL_17798474, EPI_ISL_17798475, EPI_ISL_17798476, EPI_ISL_17798477, EPI_ISL_17798478, EPI_ISL_17798479, EPI_ISL_17798480, EPI_ISL_17798481, EPI_ISL_17798482, EPI_ISL_17798483, EPI_ISL_17798484, EPI_ISL_17798485, EPI_ISL_17798486, EPI_ISL_17798487, EPI_ISL_17798488, EPI_ISL_17798489, EPI_ISL_17798490, EPI_ISL_17798491, EPI_ISL_17798492, EPI_ISL_17798493, EPI_ISL_17798494, EPI_ISL_17798495, EPI_ISL_17798496, EPI_ISL_17798497, EPI_ISL_17798498, EPI_ISL_17798499, EPI_ISL_17798500, EPI_ISL_17798501, EPI_ISL_17798502, EPI_ISL_17798503, EPI_ISL_17798504, EPI_ISL_17798505, EPI_ISL_17798506, EPI_ISL_17798507, EPI_ISL_17798508, EPI_ISL_17798509, EPI_ISL_17798510, EPI_ISL_17798511, EPI_ISL_17798512, EPI_ISL_17798513, EPI_ISL_17798514, EPI_ISL_17798515, EPI_ISL_17798516, EPI_ISL_17798517, EPI_ISL_17798518, EPI_ISL_17798519, EPI_ISL_17798520, EPI_ISL_17798521, EPI_ISL_17798522, EPI_ISL_17798523, EPI_ISL_17798524, EPI_ISL_17798525, EPI_ISL_17798526, EPI_ISL_17798527, EPI_ISL_17798528, EPI_ISL_17798529, EPI_ISL_17798530, EPI_ISL_17798531, EPI_ISL_17798532, EPI_ISL_17798533, EPI_ISL_17798534, EPI_ISL_17798535, EPI_ISL_17798536, EPI_ISL_17798537, EPI_ISL_17798538, EPI_ISL_17798539, EPI_ISL_17798540, EPI_ISL_17798541, EPI_ISL_17798542, EPI_ISL_17798543, EPI_ISL_17798544, EPI_ISL_17798545, EPI_ISL_17798546, EPI_ISL_17798547, EPI_ISL_17798548, EPI_ISL_17798549, EPI_ISL_17798550, EPI_ISL_17798551, EPI_ISL_17798552, EPI_ISL_17798553, EPI_ISL_17798554, EPI_ISL_17798555, EPI_ISL_17798556, EPI_ISL_17798557, EPI_ISL_17798558, EPI_ISL_17798559, EPI_ISL_17798560, EPI_ISL_17798561, EPI_ISL_17798562, EPI_ISL_17798563, EPI_ISL_17798564, EPI_ISL_17798565, EPI_ISL_17798566, EPI_ISL_17798567, EPI_ISL_17798568, EPI_ISL_17798569, EPI_ISL_17798570, EPI_ISL_17798571, EPI_ISL_17798572, EPI_ISL_17798573, EPI_ISL_17798574, EPI_ISL_17798575, EPI_ISL_17798576, EPI_ISL_17798577, EPI_ISL_17798578, EPI_ISL_17798579, EPI_ISL_17798580, EPI_ISL_17798581, EPI_ISL_17798582, EPI_ISL_17798583, EPI_ISL_17798584, EPI_ISL_17798585, EPI_ISL_17798586, EPI_ISL_17798587, EPI_ISL_17798588, EPI_ISL_17798589, EPI_ISL_17798590, EPI_ISL_17798591			
see above	Environmental Health Institute, National Environment Agency	Environmental Health Institute, National Environment Agency	Sy,A.K., Koo,C., Privaldos,K.J.R., Quinones,M.A.T., Igoy,M.A.U., Villanueva,S.Y.A.M., Hibberd,M.L., Ng,L.C. and Hapuarachchi,H.C.
EPI_ISL_17798592, EPI_ISL_17798593, EPI_ISL_17798594, EPI_ISL_17798595, EPI_ISL_17798596, EPI_ISL_17798597, EPI_ISL_17798598, EPI_ISL_17798599, EPI_ISL_17798600, EPI_ISL_17798601, EPI_ISL_17798602, EPI_ISL_17798603, EPI_ISL_17798604, EPI_ISL_17798605, EPI_ISL_17798606, EPI_ISL_17798607, EPI_ISL_17798608, EPI_ISL_17798609, EPI_ISL_17798610, EPI_ISL_17798611, EPI_ISL_17798612, EPI_ISL_17798613, EPI_ISL_17798614, EPI_ISL_17798615, EPI_ISL_17798616, EPI_ISL_17798617, EPI_ISL_17798618, EPI_ISL_17798619, EPI_ISL_17798620, EPI_ISL_17798621, EPI_ISL_17798622, EPI_ISL_17798623, EPI_ISL_17798624, EPI_ISL_17798625, EPI_ISL_17798626, EPI_ISL_17798627, EPI_ISL_17798628, EPI_ISL_17798629, EPI_ISL_17798630, EPI_ISL_17798631, EPI_ISL_17798632, EPI_ISL_17798633, EPI_ISL_17798634, EPI_ISL_17798635, EPI_ISL_17798636, EPI_ISL_17798637, EPI_ISL_17798638, EPI_ISL_17798639, EPI_ISL_17798640, EPI_ISL_17798641, EPI_ISL_17798642, EPI_ISL_17798643, EPI_ISL_17798644, EPI_ISL_17798645, EPI_ISL_17798646, EPI_ISL_17798647, EPI_ISL_17798648, EPI_ISL_17798649, EPI_ISL_17798650, EPI_ISL_17798651, EPI_ISL_17798652, EPI_ISL_17798653, EPI_ISL_17798654, EPI_ISL_17798655, EPI_ISL_17798656, EPI_ISL_17798657, EPI_ISL_17798658, EPI_ISL_17798659, EPI_ISL_17798660, EPI_ISL_17798661, EPI_ISL_17798662, EPI_ISL_17798663, EPI_ISL_17798664, EPI_ISL_17798665, EPI_ISL_17798666, EPI_ISL_17798667, EPI_ISL_17798668, EPI_ISL_17798669, EPI_ISL_17798670, EPI_ISL_17798671, EPI_ISL_17798672, EPI_ISL_17798673, EPI_ISL_17798674, EPI_ISL_17798675, EPI_ISL_17798676, EPI_ISL_17798677, EPI_ISL_17798678, EPI_ISL_17798679, EPI_ISL_17798680, EPI_ISL_17798681, EPI_ISL_17798682, EPI_ISL_17798683, EPI_ISL_17798684, EPI_ISL_17798685, EPI_ISL_17798686, EPI_ISL_17798687, EPI_ISL_17798688, EPI_ISL_17798689, EPI_ISL_17798690, EPI_ISL_17798691, EPI_ISL_17798692, EPI_ISL_17798693, EPI_ISL_17798694, EPI_ISL_17798695, EPI_ISL_17798696, EPI_ISL_17798697, EPI_ISL_17798698, EPI_ISL_17798699, EPI_ISL_17798700, EPI_ISL_17798701, EPI_ISL_17798702, EPI_ISL_17798703, EPI_ISL_17798704, EPI_ISL_17798705, EPI_ISL_17798706, EPI_ISL_17798707, EPI_ISL_17798708, EPI_ISL_17798709, EPI_ISL_17798710, EPI_ISL_17798711, EPI_ISL_17798712, EPI_ISL_17798713, EPI_ISL_17798714, EPI_ISL_17798715, EPI_ISL_17798716, EPI_ISL_17798717, EPI_ISL_17798718, EPI_ISL_17798719, EPI_ISL_17798720, EPI_ISL_17798721, EPI_ISL_17798722, EPI_ISL_17798723, EPI_ISL_17798724, EPI_ISL_17798725, EPI_ISL_17798726, EPI_ISL_17798727, EPI_ISL_17798728, EPI_ISL_17798729, EPI_ISL_17798730, EPI_ISL_17798731, EPI_ISL_17798732, EPI_ISL_17798733, EPI_ISL_17798734, EPI_ISL_17798735, EPI_ISL_17798736, EPI_ISL_17798737, EPI_ISL_17798738, EPI_ISL_17798739, EPI_ISL_17798740, EPI_ISL_17798741, EPI_ISL_17798742, EPI_ISL_17798743, EPI_ISL_17798744, EPI_ISL_17798745, EPI_ISL_17798746, EPI_ISL_17798747, EPI_ISL_17798748, EPI_ISL_17798749, EPI_ISL_17798750, EPI_ISL_17798751, EPI_ISL_17798752, EPI_ISL_17798753, EPI_ISL_17798754, EPI_ISL_17798755, EPI_ISL_17798756, EPI_ISL_17798757, EPI_ISL_17798758, EPI_ISL_17798759, EPI_ISL_17798760, EPI_ISL_17798761, EPI_ISL_17798762, EPI_ISL_17798763, EPI_ISL_17798764, EPI_ISL_17798765, EPI_ISL_17798766, EPI_ISL_17798767, EPI_ISL_17798768, EPI_ISL_17798769, EPI_ISL_17798770			
see above	Evercare Hospital Dhaka (Ex Apollo Hospitals Dhaka)	Research Institute for Microbial Diseases, Osaka University, Department of Viral Infections	Rahim,R., Hasan,A., Phadungsombat,J., Hasan,N., Ara,N., Biswas,S.M., Nakayama,E.E., Rahman,M. and Shioda,T.
EPI_ISL_17798771, EPI_ISL_17798772, EPI_ISL_17798773, EPI_ISL_17798774, EPI_ISL_17798775, EPI_ISL_17798776, EPI_ISL_17798777, EPI_ISL_17798778, EPI_ISL_17798779, EPI_ISL_17798780, EPI_ISL_17798781, EPI_ISL_17798782, EPI_ISL_17798783, EPI_ISL_17798784, EPI_ISL_17798785, EPI_ISL_17798786, EPI_ISL_17798787, EPI_ISL_17798788, EPI_ISL_17798789, EPI_ISL_17798790, EPI_ISL_17798791, EPI_ISL_17798792, EPI_ISL_17798793, EPI_ISL_17798794, EPI_ISL_17798795			
see above	Sun Yat-Sen University, Sun Yat-Sen Memorial Hospital, Guangdong Provincial Key Laboratory of Malignant Tumor Epigenetics and Gene Regulation	Sun Yat-Sen University, Sun Yat-Sen Memorial Hospital, Guangdong Provincial Key Laboratory of Malignant Tumor Epigenetics and Gene Regulation	Meng,J.X., Hu,Q.M., Zhang,L.M., Li,N., He,Y.W., Yang,Z.X., Sun,Y. and Wang,J.L.
EPI_ISL_17804503, EPI_ISL_17804504, EPI_ISL_17804505, EPI_ISL_17804506, EPI_ISL_17804507, EPI_ISL_17804508, EPI_ISL_17804509, EPI_ISL_17804510, EPI_ISL_17804511, EPI_ISL_17804512, EPI_ISL_17804513, EPI_ISL_17804514, EPI_ISL_17804515, EPI_ISL_17804516, EPI_ISL_17804517, EPI_ISL_17804518, EPI_ISL_17804519, EPI_ISL_17804520, EPI_ISL_17804521, EPI_ISL_17804522, EPI_ISL_17804523, EPI_ISL_17804524, EPI_ISL_17804525, EPI_ISL_17804526, EPI_ISL_17804527, EPI_ISL_17804528, EPI_ISL_17804529, EPI_ISL_17804530, EPI_ISL_17804531, EPI_ISL_17804532, EPI_ISL_17804533, EPI_ISL_17804534			
see above	California Academy of Sciences	California Academy of Sciences	Thongrsipong,P., Edgerton,S.V., Bos,S., Saborio,S., Kuan,G., Balmaseda,A., Harris,E. and Bennett,S.N.
EPI_ISL_17831892	State Center for Health Surveillance/Secretary of State for Health	State Center for Health Surveillance/Secretary of State for Health	Salvato,R.S., Wallau,G.L. and Graf,T.
EPI_ISL_17831894, EPI_ISL_17831895	Research Medical Center of Lambarene	Research Medical Center of Lambarene	Bikangui,R.
EPI_ISL_17831896	Blood Center of Ribeirao Preto, University of Sao Paulo	Blood Center of Ribeirao Preto, University of Sao Paulo	La-Roque,D.G.L., Santos,E.V., Oliveira,R.A.M., Slavov,S.N., Rodrigues,E.S., Fonseca,V., Martins,A.J., Giomo,D.B., Torres,P.M.A., Gentil,D.C.D., Catoia,E.A., Chiquito,N.C., Medeiros,A.P.S.S., Yamamoto,A.Y., Passos,L.M.R., Calado,R.T., Elias,M.C., Sampaio,S.C., Giovanetti,M., Alcantara,L.C., Covas,D.T. and Kashima,S.
EPI_ISL_17831897, EPI_ISL_17831898, EPI_ISL_17831899, EPI_ISL_17831900	Department of Virology and Immunity, Guangzhou Center for Disease Control and Prevention	Department of Virology and Immunity, Guangzhou Center for Disease Control and Prevention	Su,W., Lu,W. and Cao,Y.
EPI_ISL_17831901, EPI_ISL_17831902, EPI_ISL_17831903, EPI_ISL_17831904, EPI_ISL_17831905, EPI_ISL_17831906, EPI_ISL_17831907, EPI_ISL_17831908, EPI_ISL_17831909, EPI_ISL_17831910, EPI_ISL_17831911, EPI_ISL_17831912, EPI_ISL_17831913, EPI_ISL_17831914, EPI_ISL_17831915, EPI_ISL_17831916, EPI_ISL_17831917, EPI_ISL_17831918, EPI_ISL_17831919, EPI_ISL_17831920, EPI_ISL_17831921, EPI_ISL_17831922, EPI_ISL_17831923, EPI_ISL_17831924, EPI_ISL_17831925, EPI_ISL_17831926, EPI_ISL_17831927, EPI_ISL_17831928, EPI_ISL_17831929, EPI_ISL_17831930, EPI_ISL_17831931, EPI_ISL_17831932, EPI_ISL_17831933, EPI_ISL_17831934, EPI_ISL_17831935, EPI_ISL_17831936			
see above	National Hospital for Tropical Disease (NHTD)	Research Institute for Microbial Diseases, Osaka University, Department of Viral Infections	Phadungsombat,J., Vu,H.T.T., Nguyen,Q.T., Nguyen,H.T.V., Nguyen,H.T.N., Dang,B.T., Nakayama,E.E., Ishizaki,A., Ichimura,H., Shioda,T. and Pham,T.N.
EPI_ISL_17848715, EPI_ISL_17848716	Department of Microbiology, Zhongshan School of Medicine, Sun Yat-Sen University, Key Laboratory of Tropical Disease Control, Ministry of Education	Department of Microbiology, Zhongshan School of Medicine, Sun Yat-Sen University, Key Laboratory of Tropical Disease Control, Ministry of Education	Li,Y.
EPI_ISL_17848717	Department of Microbiology, Zhongshan School of Medicine, Sun Yat-Sen University, Key Laboratory of Tropical Disease Control, Ministry of Education	Department of Microbiology, Zhongshan School of Medicine, Sun Yat-Sen University, Key Laboratory of Tropical Disease Control, Ministry of Education	Li,Y.-P., Hu,M., Wu,D. and Wu,T.
EPI_ISL_17859404, EPI_ISL_17859405, EPI_ISL_17859406, EPI_ISL_17859407, EPI_ISL_17859408, EPI_ISL_17859409, EPI_ISL_17859410, EPI_ISL_17859411, EPI_ISL_17859412, EPI_ISL_17859413, EPI_ISL_17859414, EPI_ISL_17859415, EPI_ISL_17859416, EPI_ISL_17859417, EPI_ISL_17859418, EPI_ISL_17859419, EPI_ISL_17859420, EPI_ISL_17859421, EPI_ISL_17859422, EPI_ISL_17859423, EPI_ISL_17859424, EPI_ISL_17859425, EPI_ISL_17859426, EPI_ISL_17859427, EPI_ISL_17859428, EPI_ISL_17859429, EPI_ISL_17859430, EPI_ISL_17859431, EPI_ISL_17859432, EPI_ISL_17859433, EPI_ISL_17859434, EPI_ISL_17859435, EPI_ISL_17859436, EPI_ISL_17859437, EPI_ISL_17859438, EPI_ISL_17859439, EPI_ISL_17859440, EPI_ISL_17859441, EPI_ISL_17859442			
see above	Coordenacao Geral da Laboratorio em Saude Publica	Coordenacao Geral da Laboratorio em Saude Publica	Ribeiro,G., Parise,D., Paiva,A., Franz,H., Junior,W. and Arcanjo,A.R.
EPI_ISL_17859443	Bureau of Public Health Laboratories, Florida Department of Health	Bureau of Public Health Laboratories, Florida Department of Health	Schmedes,S., Tekin,O., Tarigopula,N. and Dong,Y.
EPI_ISL_17859444, EPI_ISL_17859445, EPI_ISL_17859446, EPI_ISL_17859447, EPI_ISL_17859448	Bureau of Public Health Laboratories, Florida Department of Health	Bureau of Public Health Laboratories, Florida Department of Health	Schmedes,S., Tarigopula,N., Tekin,O. and Dong,Y.
EPI_ISL_17859449	Bureau of Public Health Laboratories, Florida Department of Health	Bureau of Public Health Laboratories, Florida Department of Health	Schmedes,S., Tekin,O., Tarigopula,N. and Dong,Y.
EPI_ISL_17859450, EPI_ISL_17859451	Bureau of Public Health Laboratories, Florida Department of Health	Bureau of Public Health Laboratories, Florida Department of Health	Schmedes,S., Tarigopula,N., Tekin,O. and Dong,Y.
EPI_ISL_17859452	Bureau of Public Health Laboratories, Florida Department of Health	Bureau of Public Health Laboratories, Florida Department of Health	Schmedes,S., Tekin,O., Tarigopula,N. and Dong,Y.
EPI_ISL_17859453, EPI_ISL_17859454	Bureau of Public Health Laboratories, Florida Department of Health	Bureau of Public Health Laboratories, Florida Department of Health	Schmedes,S., Tarigopula,N., Tekin,O. and Dong,Y.
EPI_ISL_17859455, EPI_ISL_17859456	Bureau of Public Health Laboratories, Florida Department of Health	Bureau of Public Health Laboratories, Florida Department of Health	Schmedes,S., Tekin,O., Tarigopula,N. and Dong,Y.
EPI_ISL_17859457	Bureau of Public Health Laboratories, Florida Department of Health	Bureau of Public Health Laboratories, Florida Department of Health	Schmedes,S., Tarigopula,N., Tekin,O. and Dong,Y.
EPI_ISL_17859458, EPI_ISL_17859459, EPI_ISL_17859460	Bureau of Public Health Laboratories, Florida Department of Health	Bureau of Public Health Laboratories, Florida Department of Health	Schmedes,S., Tekin,O., Tarigopula,N. and Dong,Y.
EPI_ISL_17859461, EPI_ISL_17859462	Bureau of Public Health Laboratories, Florida Department of Health	Bureau of Public Health Laboratories, Florida Department of Health	Schmedes,S., Tarigopula,N., Tekin,O. and Dong,Y.
EPI_ISL_17859463	Bureau of Public Health Laboratories, Florida Department of Health	Bureau of Public Health Laboratories, Florida Department of Health	Schmedes,S., Tekin,O., Tarigopula,N. and Dong,Y.
EPI_ISL_17884732, EPI_ISL_17884733, EPI_ISL_17884734, EPI_ISL_17884735, EPI_ISL_17884736, EPI_ISL_17884737, EPI_ISL_17884738, EPI_ISL_17884739, EPI_ISL_17884740, EPI_ISL_17884741, EPI_ISL_17884742, EPI_ISL_17884743, EPI_ISL_17884744, EPI_ISL_17884745, EPI_ISL_17884746, EPI_ISL_17884747, EPI_ISL_17884748, EPI_ISL_17884749, EPI_ISL_17884750, EPI_ISL_17884751, EPI_ISL_17884752, EPI_ISL_17884753, EPI_ISL_17884754, EPI_ISL_17884755, EPI_ISL_17884756, EPI_ISL_17884757, EPI_ISL_17884758, EPI_ISL_17884759, EPI_ISL_17884760, EPI_ISL_17884761, EPI_ISL_17884762, EPI_ISL_17884763, EPI_ISL_17884764, EPI_ISL_17884765, EPI_ISL_17884766, EPI_ISL_17884767, EPI_ISL_17884768, EPI_ISL_17884769, EPI_ISL_17884770, EPI_ISL_17884771, EPI_ISL_17884772, EPI_ISL_17884773, EPI_ISL_17884774, EPI_ISL_17884775, EPI_ISL_17884776, EPI_ISL_17884777, EPI_ISL_17884778, EPI_ISL_17884779, EPI_ISL_17884780, EPI_ISL_17884781, EPI_ISL_17884782, EPI_ISL_17884783, EPI_ISL_17884784, EPI_ISL_17884785, EPI_ISL_17884786, EPI_ISL_17884787, EPI_ISL_17884788, EPI_ISL_17884789, EPI_ISL_17884790, EPI_ISL_17884791, EPI_ISL_17884792, EPI_ISL_17884793, EPI_ISL_17884794, EPI_ISL_17884795, EPI_ISL_17884796, EPI_ISL_17884797, EPI_ISL_17884798, EPI_ISL_17884799, EPI_ISL_17884800, EPI_ISL_17884801, EPI_ISL_17884802, EPI_ISL_17884803, EPI_ISL_17884804, EPI_ISL_17884805, EPI_ISL_17884806, EPI_ISL_17884807, EPI_ISL_17884808, EPI_ISL_17884809, EPI_ISL_17884810, EPI_ISL_17884811, EPI_ISL_17884812, EPI_ISL_17884813, EPI_ISL_17884814, EPI_ISL_17884815, EPI_ISL_17884816, EPI_ISL_17884817, EPI_ISL_17884818, EPI_ISL_17884819, EPI_ISL_17884820, EPI_ISL_17884821, EPI_ISL_17884822, EPI_ISL_17884823, EPI_ISL_17884824, EPI_ISL_17884825, EPI_ISL_17884826, EPI_ISL_17884827, EPI_ISL_17884828, EPI_ISL_17884829, EPI_ISL_17884830, EPI_ISL_17884831, EPI_ISL_17884832, EPI_ISL_17884833, EPI_ISL_17884834, EPI_ISL_17884835, EPI_ISL_17884836, EPI_ISL_17884837, EPI_ISL_17884838, EPI_ISL_17884839, EPI_ISL_17884840, EPI_ISL_17884841, EPI_ISL_17884842, EPI_ISL_17884843, EPI_ISL_17884844, EPI_ISL_17884845, EPI_ISL_17884846, EPI_ISL_17884847, EPI_ISL_17884848, EPI_ISL_17884849			
see above	Central Public Health Laboratory Dr. Giovanni	Central Public Health Laboratory Dr. Giovanni	Mendonca,A.F., Andrade,C.F., Sobreiro,M.B., Almeida,R.M., Silva,V.L. and Pereira,L.A.

	Cysneiros (LACEN-GO)	Cysneiros (LACEN-GO)	
EPI_ISL_17884850, EPI_ISL_17884851, EPI_ISL_17884852, EPI_ISL_17884853, EPI_ISL_17884854, EPI_ISL_17884855, EPI_ISL_17884856, EPI_ISL_17884857, EPI_ISL_17884858, EPI_ISL_17884859, EPI_ISL_17884860, EPI_ISL_17884861, EPI_ISL_17884862, EPI_ISL_17884863, EPI_ISL_17884864, EPI_ISL_17884865, EPI_ISL_17884866, EPI_ISL_17884867, EPI_ISL_17884868, EPI_ISL_17884869, EPI_ISL_17884870, EPI_ISL_17884871			
see above	Coordenacao Geral da Laboratorio em Saude Publica	Coordenacao Geral da Laboratorio em Saude Publica	Ribeiro,G., Parise,D., Paiva,A., Franz,H., Junior,W. and Arcanjo,A.R.
EPI_ISL_17884872	Virus Laboratory, Indian Council of Medical Research, National Institute of Cholera and Enteric Diseases	Virus Laboratory, Indian Council of Medical Research, National Institute of Cholera and Enteric Diseases	Baskey,U., Verma,P., Ghosh,A. and Sadhukhan,P.C.
EPI_ISL_17959701, EPI_ISL_17959702, EPI_ISL_17959703, EPI_ISL_17959704, EPI_ISL_17959705, EPI_ISL_17959706, EPI_ISL_17959707, EPI_ISL_17959708, EPI_ISL_17959709, EPI_ISL_17959710, EPI_ISL_17959711, EPI_ISL_17959712, EPI_ISL_17959713, EPI_ISL_17959714, EPI_ISL_17959715, EPI_ISL_17959716, EPI_ISL_17959718, EPI_ISL_17959719, EPI_ISL_17959720, EPI_ISL_17959721, EPI_ISL_17959722, EPI_ISL_17959723, EPI_ISL_17959724, EPI_ISL_17959725, EPI_ISL_17959726			
see above	Institute of Microbiology, Universidad San Francisco de Quito	Institute of Microbiology, Universidad San Francisco de Quito	Sully Márquez, Gwenyth Lee, Bernardo Gutiérrez, Joseph Eisenberg, Gabriel Trueba, Josefina Coloma
EPI_ISL_17975657, EPI_ISL_17975658, EPI_ISL_17975659, EPI_ISL_17975660, EPI_ISL_17975661, EPI_ISL_17975662, EPI_ISL_17975663, EPI_ISL_17975664, EPI_ISL_17975665, EPI_ISL_17975666, EPI_ISL_17975667, EPI_ISL_17975668, EPI_ISL_17975669, EPI_ISL_17975670, EPI_ISL_17975671, EPI_ISL_17975672, EPI_ISL_17975673, EPI_ISL_17975674, EPI_ISL_17975675, EPI_ISL_17975676, EPI_ISL_17975677, EPI_ISL_17975678, EPI_ISL_17975679, EPI_ISL_17975680, EPI_ISL_17975681			
see above	Gandhi Medical College	Gandhi Medical College	Agarwal,A., Ganvir,R., Kale,D., Chaurasia,D. and Kapoor,G.
EPI_ISL_17975682, EPI_ISL_17975683, EPI_ISL_17975684, EPI_ISL_17975685, EPI_ISL_17975686, EPI_ISL_17975687, EPI_ISL_17975688, EPI_ISL_17975689, EPI_ISL_17975690, EPI_ISL_17975691, EPI_ISL_17975692, EPI_ISL_17975693, EPI_ISL_17975694, EPI_ISL_17975695, EPI_ISL_17975696, EPI_ISL_17975697, EPI_ISL_17975698, EPI_ISL_17975699			
see above	Armauer Hansen Research Institute	Armauer Hansen Research Institute	Mengesha,M., Tayachew,A., Tsega,D., Hailu,D., Abeje,D., Seyoum,T., Adnew,B., Konde,A., Biru,M., Agunie,A., Asrat,Y., Wossen,M., Gelanew,T., Piantadosi,A., Mihret,A. and Mulu,A.
EPI_ISL_17976304, EPI_ISL_17977444	Instituto de Investigaciones Biologicas del Tropico, Universidad de Córdoba	Instituto de Investigaciones Biologicas del Tropico, Universidad de Córdoba	Ricardo Rivero, Daniela Paternina, Vaneza Tique, Salim Mattar
EPI_ISL_17980694, EPI_ISL_17980695, EPI_ISL_17980696, EPI_ISL_17980697, EPI_ISL_17980698, EPI_ISL_17980699, EPI_ISL_17980700, EPI_ISL_17980701, EPI_ISL_17980702, EPI_ISL_17980703, EPI_ISL_17980704, EPI_ISL_17980705			
see above	Department of Microbiology	Department of Microbiology	Okada,W., Kasuya,F., Harada,S., Kumagai,R., Amano,A., Hasegawa,M., Miyake,H., Nagashima,M. and Sadamasu,K.
EPI_ISL_17980809, EPI_ISL_17980810	Hospital Maternidade Sao Jose (Guaraciaba Do Norte)	Laboratório Central de Saúde Pública do Ceará	Tayna da Silva Fiuza, Vânia Angélica Feitosa Viana, Shirlene Telmos Silva de Lima
EPI_ISL_17980811	Laboratorio De Patologia Clinica De Morada Nova	Laboratório Central de Saúde Pública do Ceará	Tayna da Silva Fiuza, Vânia Angélica Feitosa Viana, Shirlene Telmos Silva de Lima
EPI_ISL_17980812	Hospital Municipal Jaime Osterno (Marco)	Laboratório Central de Saúde Pública do Ceará	Tayna da Silva Fiuza, Vânia Angélica Feitosa Viana, Shirlene Telmos Silva de Lima
EPI_ISL_17980813	Centro De Saude De Bela Cruzfns(Bela Cruz)	Laboratório Central de Saúde Pública do Ceará	Tayna da Silva Fiuza, Vânia Angélica Feitosa Viana, Shirlene Telmos Silva de Lima
EPI_ISL_17980814	Hospital Municipal De Jaguaribe	Laboratório Central de Saúde Pública do Ceará	Tayna da Silva Fiuza, Vânia Angélica Feitosa Viana, Shirlene Telmos Silva de Lima
EPI_ISL_17980815, EPI_ISL_17980816, EPI_ISL_17980817, EPI_ISL_17980818, EPI_ISL_17980819, EPI_ISL_17980820, EPI_ISL_17980821, EPI_ISL_17980822, EPI_ISL_17980823, EPI_ISL_17980824, EPI_ISL_17980825, EPI_ISL_17980826, EPI_ISL_17980827			
see above	Centro De Saude De Bela Cruzfns(Bela Cruz)	Laboratório Central de Saúde Pública do Ceará	Tayna da Silva Fiuza, Vânia Angélica Feitosa Viana, Shirlene Telmos Silva de Lima
EPI_ISL_17980828	Lab De Analises Clinicas De Cruz	Laboratório Central de Saúde Pública do Ceará	Tayna da Silva Fiuza, Vânia Angélica Feitosa Viana, Shirlene Telmos Silva de Lima
EPI_ISL_17980829	Hosp Munic Dr Deoclecio Lima Verde (Limoeiro)	Laboratório Central de Saúde Pública do Ceará	Tayna da Silva Fiuza, Vânia Angélica Feitosa Viana, Shirlene Telmos Silva de Lima
EPI_ISL_17980830	Centro De Saude De Bela Cruzfns(Bela Cruz)	Laboratório Central de Saúde Pública do Ceará	Tayna da Silva Fiuza, Vânia Angélica Feitosa Viana, Shirlene Telmos Silva de Lima
EPI_ISL_17980831, EPI_ISL_17980832	Hospital Municipal De Jaguaribe	Laboratório Central de Saúde Pública do Ceará	Tayna da Silva Fiuza, Vânia Angélica Feitosa Viana, Shirlene Telmos Silva de Lima
EPI_ISL_17980833	Centro De Saude De Bela Cruzfns(Bela Cruz)	Laboratório Central de Saúde Pública do Ceará	Tayna da Silva Fiuza, Vânia Angélica Feitosa Viana, Shirlene Telmos Silva de Lima
EPI_ISL_17980834, EPI_ISL_17980835, EPI_ISL_17980836, EPI_ISL_17980837	Hospital Municipal De Jaguaribe	Laboratório Central de Saúde Pública do Ceará	Tayna da Silva Fiuza, Vânia Angélica Feitosa Viana, Shirlene Telmos Silva de Lima
EPI_ISL_17980838, EPI_ISL_17980839, EPI_ISL_17980840, EPI_ISL_17980841, EPI_ISL_17980842, EPI_ISL_17980843, EPI_ISL_17980844, EPI_ISL_17980845, EPI_ISL_17980846, EPI_ISL_17980847, EPI_ISL_17980848			
see above	Centro De Saude De Bela Cruzfns(Bela Cruz)	Laboratório Central de Saúde Pública do Ceará	Tayna da Silva Fiuza, Vânia Angélica Feitosa Viana, Shirlene Telmos Silva de Lima
EPI_ISL_17980849	Hospital Infantil Albert Sabin (Fortaleza)	Laboratório Central de Saúde Pública do Ceará	Tayna da Silva Fiuza, Vânia Angélica Feitosa Viana, Shirlene Telmos Silva de Lima
EPI_ISL_17983067	LACEN de Tocantins	Instituto Adolfo Lutz - Strategic Laboratory	Claudio Tavares Sacchi, Karoline Rodrigues Campos, Marlon Benedito Nascimento Santos, Franciano Dias Pereira Cardoso
EPI_ISL_17983068, EPI_ISL_17983069, EPI_ISL_17983070, EPI_ISL_17983071	LACEN de Sergipe	Instituto Adolfo Lutz - Strategic Laboratory	Claudio Tavares Sacchi, Karoline Rodrigues Campos, Marlon Benedito Nascimento Santos, Gabriela Vasconcelos Brito Bezerra
EPI_ISL_17983072, EPI_ISL_17983073, EPI_ISL_17983074, EPI_ISL_17983075, EPI_ISL_17983076, EPI_ISL_17983077, EPI_ISL_17983078, EPI_ISL_17983079, EPI_ISL_17983080, EPI_ISL_17983081, EPI_ISL_17983082, EPI_ISL_17983083, EPI_ISL_17983084, EPI_ISL_17983085, EPI_ISL_17983086, EPI_ISL_17983087			
see above	Instituto Adolfo Lutz	Instituto Adolfo Lutz - Strategic Laboratory	Claudio Tavares Sacchi, Karoline Rodrigues Campos, Marlon Benedito Nascimento Santos, Juliana Silva Nogueira
EPI_ISL_17983088, EPI_ISL_17983089, EPI_ISL_17983090, EPI_ISL_17983091	LACEN de Sergipe	Instituto Adolfo Lutz - Strategic Laboratory	Claudio Tavares Sacchi, Karoline Rodrigues Campos, Marlon Benedito Nascimento Santos, Gabriela Vasconcelos Brito Bezerra
EPI_ISL_17983092, EPI_ISL_17983093, EPI_ISL_17983094	LACEN de Tocantins	Instituto Adolfo Lutz - Strategic Laboratory	Claudio Tavares Sacchi, Karoline Rodrigues Campos, Marlon Benedito Nascimento Santos, Franciano Dias Pereira Cardoso
EPI_ISL_17983095	LACEN de Tocantins	Instituto Adolfo Lutz - Strategic Laboratory	Claudio Tavares Sacchi, Karoline Rodrigues Campos, Marlon Benedito Nascimento Santos, Gabriela Vasconcelos Brito Bezerra
EPI_ISL_18001716	Armauer Hansen Research Institute	Armauer Hansen Research Institute	Mengesha,M., Tayachew,A., Tsega,D., Hailu,D., Abeje,D., Seyoum,T., Adnew,B., Konde,A., Biru,M., Agunie,A., Asrat,Y., Wossen,M., Gelanew,T., Piantadosi,A., Mihret,A. and Mulu,A.
EPI_ISL_18001717, EPI_ISL_18001718, EPI_ISL_18001719, EPI_ISL_18001720, EPI_ISL_18001721, EPI_ISL_18001722, EPI_ISL_18001723, EPI_ISL_18001724, EPI_ISL_18001725, EPI_ISL_18001726, EPI_ISL_18001727, EPI_ISL_18001728			
see above	Department of Microbiology	Department of Microbiology	Okada,W., Kasuya,F., Harada,S., Kumagai,R., Amano,A., Hasegawa,M., Miyake,H., Nagashima,M. and Sadamasu,K.
EPI_ISL_18001729, EPI_ISL_18001730, EPI_ISL_18001731, EPI_ISL_18001732, EPI_ISL_18001733, EPI_ISL_18001734, EPI_ISL_18001735, EPI_ISL_18001736, EPI_ISL_18001737, EPI_ISL_18001738, EPI_ISL_18001739, EPI_ISL_18001740, EPI_ISL_18001741, EPI_ISL_18001742, EPI_ISL_18001743, EPI_ISL_18001744, EPI_ISL_18001745			
see above	Gandhi Medical College	Gandhi Medical College	Agarwal,A., Ganvir,R., Kale,D., Chaurasia,D. and Kapoor,G.
EPI_ISL_18007204, EPI_ISL_18007205, EPI_ISL_18007206, EPI_ISL_18007207, EPI_ISL_18007208, EPI_ISL_18007209, EPI_ISL_18007210, EPI_ISL_18007211, EPI_ISL_18007212	Centro Nacional de Enfermedades Tropicales	Centro Nacional de Enfermedades Tropicales	Roca,Y., Baronti,C., Revollo,R.J., Cook,S., Loayza,R., Ninove,L., Fernandez,R.T., Flores,J.V., Herve,J.P. and de Lamballerie,X.
EPI_ISL_18007213	Department of Virology and Immunity, Guangzhou Center for Disease Control and Prevention	Department of Virology and Immunity, Guangzhou Center for Disease Control and Prevention	Su,W., Lu,W. and Cao,Y.
EPI_ISL_18009962, EPI_ISL_18009963	LACEN-AL	Laboratório Central de Saúde Pública do Ceará	Tayna da Silva Fiuza, Vânia Angélica Feitosa Viana, Shirlene Telmos Silva de Lima
EPI_ISL_18009964	LACEN-MA	Laboratório Central de Saúde Pública do Ceará	Tayna da Silva Fiuza, Vânia Angélica Feitosa Viana, Shirlene Telmos Silva de Lima on behalf of LACEN-MA
EPI_ISL_18009965	Unidade Mista Dr Edmar Da Costa Barroso (Pacatuba)	Laboratório Central de Saúde Pública do Ceará	Tayna da Silva Fiuza, Vânia Angélica Feitosa Viana, Shirlene Telmos Silva de Lima
EPI_ISL_18009966, EPI_ISL_18009967, EPI_ISL_18009968, EPI_ISL_18009969, EPI_ISL_18009970, EPI_ISL_18009971, EPI_ISL_18009972	Hospital Municipal De Jaguaribe	Laboratório Central de Saúde Pública do Ceará	Tayna da Silva Fiuza, Vânia Angélica Feitosa Viana, Shirlene Telmos Silva de Lima
EPI_ISL_18009973	Lab De Analises Clinicas De Cruz	Laboratório Central de Saúde Pública do Ceará	Tayna da Silva Fiuza, Vânia Angélica Feitosa Viana, Shirlene Telmos Silva de Lima
EPI_ISL_18009974	Hospital Municipal De Jaguaribe	Laboratório Central de Saúde Pública do Ceará	Tayna da Silva Fiuza, Vânia Angélica Feitosa Viana, Shirlene Telmos Silva de Lima
EPI_ISL_18009975	Hospital Municipal Jaime Osterno (Marco)	Laboratório Central de Saúde Pública do Ceará	Tayna da Silva Fiuza, Vânia Angélica Feitosa Viana, Shirlene Telmos Silva de Lima
EPI_ISL_18009976	Lab De Analises Clinicas De Cruz	Laboratório Central de Saúde Pública do Ceará	Tayna da Silva Fiuza, Vânia Angélica Feitosa Viana, Shirlene Telmos Silva de Lima
EPI_ISL_18009977, EPI_ISL_18009978	Hospital Municipal De Jaguaribe	Laboratório Central de Saúde Pública do Ceará	Tayna da Silva Fiuza, Vânia Angélica Feitosa Viana, Shirlene Telmos Silva de Lima
EPI_ISL_18009979	Hospital Da Mulher De Fortaleza	Laboratório Central de Saúde Pública do Ceará	Tayna da Silva Fiuza, Vânia Angélica Feitosa Viana, Shirlene Telmos Silva de Lima
EPI_ISL_18009980	Hospital Municipal Jaime Osterno (Marco)	Laboratório Central de Saúde Pública do Ceará	Tayna da Silva Fiuza, Vânia Angélica Feitosa Viana, Shirlene Telmos Silva de Lima
EPI_ISL_18009981, EPI_ISL_18009982, EPI_ISL_18009983	Hospital Municipal De Jaguaribe	Laboratório Central de Saúde Pública do Ceará	Tayna da Silva Fiuza, Vânia Angélica Feitosa Viana, Shirlene Telmos Silva de Lima
EPI_ISL_18009984	Lab De Analises Clinicas De Cruz	Laboratório Central de Saúde Pública do Ceará	Tayna da Silva Fiuza, Vânia Angélica Feitosa Viana, Shirlene Telmos Silva de Lima
EPI_ISL_18009985, EPI_ISL_18009986	Hospital Municipal De Jaguaribe	Laboratório Central de Saúde Pública do Ceará	Tayna da Silva Fiuza, Vânia Angélica Feitosa Viana, Shirlene Telmos Silva de Lima
EPI_ISL_18009987	Hospital Sao Jose (Fortaleza)	Laboratório Central de Saúde Pública do Ceará	Tayna da Silva Fiuza, Vânia Angélica Feitosa Viana, Shirlene Telmos Silva de Lima
EPI_ISL_18009988, EPI_ISL_18009989, EPI_ISL_18009990, EPI_ISL_18009991	Lab De Analises Clinicas De Cruz	Laboratório Central de Saúde Pública do Ceará	Tayna da Silva Fiuza, Vânia Angélica Feitosa Viana, Shirlene Telmos Silva de Lima
EPI_ISL_18009992, EPI_ISL_18009993, EPI_ISL_18009994, EPI_ISL_18009995	Hospital Municipal De Jaguaribe	Laboratório Central de Saúde Pública do Ceará	Tayna da Silva Fiuza, Vânia Angélica Feitosa Viana, Shirlene Telmos Silva de Lima
EPI_ISL_18009996	Casa De Saude Adilia Maria (Boa Viagem)	Laboratório Central de Saúde Pública do Ceará	Tayna da Silva Fiuza, Vânia Angélica Feitosa Viana, Shirlene Telmos Silva de Lima

EPI_ISL_18009997	Lab De Analises Clinicas De Cruz	Laboratório Central de Saúde Pública do Ceará	Tayna da Silva Fiuza, Vânia Angélica Feitosa Viana, Shirlene Telmos Silva de Lima
EPI_ISL_18009998	Hospital Municipal De Jaguaribe	Laboratório Central de Saúde Pública do Ceará	Tayna da Silva Fiuza, Vânia Angélica Feitosa Viana, Shirlene Telmos Silva de Lima
EPI_ISL_18009999	Centro De Saude Dr Nestor De Paula Pessoa (Acarau)	Laboratório Central de Saúde Pública do Ceará	Tayna da Silva Fiuza, Vânia Angélica Feitosa Viana, Shirlene Telmos Silva de Lima
EPI_ISL_18010000, EPI_ISL_18010001, EPI_ISL_18010002	Lab De Analises Clinicas De Cruz	Laboratório Central de Saúde Pública do Ceará	Tayna da Silva Fiuza, Vânia Angélica Feitosa Viana, Shirlene Telmos Silva de Lima
EPI_ISL_18010003, EPI_ISL_18010004, EPI_ISL_18010005, EPI_ISL_18010006	Hospital Municipal De Jaguaribe	Laboratório Central de Saúde Pública do Ceará	Tayna da Silva Fiuza, Vânia Angélica Feitosa Viana, Shirlene Telmos Silva de Lima
EPI_ISL_18010007, EPI_ISL_18010008, EPI_ISL_18010009	Lab De Analises Clinicas De Cruz	Laboratório Central de Saúde Pública do Ceará	Tayna da Silva Fiuza, Vânia Angélica Feitosa Viana, Shirlene Telmos Silva de Lima
EPI_ISL_18010010, EPI_ISL_18010011	Hospital Municipal De Jaguaribe	Laboratório Central de Saúde Pública do Ceará	Tayna da Silva Fiuza, Vânia Angélica Feitosa Viana, Shirlene Telmos Silva de Lima
EPI_ISL_18010012	Lamac Laboratorio Municipal De Analises Clinicas (Camocim)	Laboratório Central de Saúde Pública do Ceará	Tayna da Silva Fiuza, Vânia Angélica Feitosa Viana, Shirlene Telmos Silva de Lima
EPI_ISL_18010013	Hospital Municipal De Jaguaribe	Laboratório Central de Saúde Pública do Ceará	Tayna da Silva Fiuza, Vânia Angélica Feitosa Viana, Shirlene Telmos Silva de Lima
EPI_ISL_18010014	Hospital Geral Dr Waldemar Alcantara	Laboratório Central de Saúde Pública do Ceará	Tayna da Silva Fiuza, Vânia Angélica Feitosa Viana, Shirlene Telmos Silva de Lima
EPI_ISL_18010015, EPI_ISL_18010016	Hospital Municipal De Jaguaribe	Laboratório Central de Saúde Pública do Ceará	Tayna da Silva Fiuza, Vânia Angélica Feitosa Viana, Shirlene Telmos Silva de Lima
EPI_ISL_18010017, EPI_ISL_18010018	Centro De Saude De Groairas	Laboratório Central de Saúde Pública do Ceará	Tayna da Silva Fiuza, Vânia Angélica Feitosa Viana, Shirlene Telmos Silva de Lima
EPI_ISL_18010019, EPI_ISL_18010020, EPI_ISL_18010021, EPI_ISL_18010022, EPI_ISL_18010023, EPI_ISL_18010024	Centro De Saude De Bela Cruzfns(Bela Cruz)	Laboratório Central de Saúde Pública do Ceará	Tayna da Silva Fiuza, Vânia Angélica Feitosa Viana, Shirlene Telmos Silva de Lima
EPI_ISL_18010025	Hospital Municipal De Jaguaribe	Laboratório Central de Saúde Pública do Ceará	Tayna da Silva Fiuza, Vânia Angélica Feitosa Viana, Shirlene Telmos Silva de Lima
EPI_ISL_18010026	Centro De Saude De Bela Cruzfns(Bela Cruz)	Laboratório Central de Saúde Pública do Ceará	Tayna da Silva Fiuza, Vânia Angélica Feitosa Viana, Shirlene Telmos Silva de Lima
EPI_ISL_18010027, EPI_ISL_18010028	Hospital Municipal De Jaguaribe	Laboratório Central de Saúde Pública do Ceará	Tayna da Silva Fiuza, Vânia Angélica Feitosa Viana, Shirlene Telmos Silva de Lima
EPI_ISL_18010029, EPI_ISL_18010030	Centro De Saude De Bela Cruzfns(Bela Cruz)	Laboratório Central de Saúde Pública do Ceará	Tayna da Silva Fiuza, Vânia Angélica Feitosa Viana, Shirlene Telmos Silva de Lima
EPI_ISL_18029956, EPI_ISL_18029957, EPI_ISL_18029958, EPI_ISL_18029959, EPI_ISL_18029960, EPI_ISL_18029961, EPI_ISL_18029962, EPI_ISL_18029963, EPI_ISL_18029964, EPI_ISL_18029965, EPI_ISL_18029966, EPI_ISL_18029967, EPI_ISL_18029968, EPI_ISL_18029969, EPI_ISL_18029970, EPI_ISL_18029971, EPI_ISL_18029972, EPI_ISL_18029973			
see above	Department of Microbiology	Department of Microbiology	Okada,W., Kasuya,F., Harada,S., Kumagai,R., Amano,A., Hasegawa,M., Miyake,H., Nagashima,M. and Sadamasu,K.
EPI_ISL_18059883, EPI_ISL_18059884, EPI_ISL_18059885	Ministry of Health, Central Virology Laboratory	Ministry of Health, Central Virology Laboratory	Zuckerman,N.S., Schwartz,E., Pandey,P., Halpern,O., Bucris,E., Morad,H., Geva,M., Wax,M. and Lustig,Y.
EPI_ISL_18103416, EPI_ISL_18103417, EPI_ISL_18103418	Undisclosed Laboratory in Brazil for the Health Emergency Information and Risk Assessment (HIM) Unit of PAHO	Undisclosed Laboratory in Brazil for the Health Emergency Information and Risk Assessment (HIM) Unit of PAHO	Vazquez,C., Luiz Carlos Junior Alcantara, Vagner Fonseca, Mauricio Lima, Joilson Xavier, Adelino,T., Hegger Fritsch, Castro,E., de Oliveira,C., Schuab,G., Lima,A.R.J., Villalba,S., Gomez de la Fuente,A., Rojas,A., Cantero,C., Fleitas,F., Aquino,C., Ojeda,A., Sequera,G., Torales,J., Barrios,J., Elias,M.C., Iani,F.C.M., Ortega,M.J., Gamarra,M.L., Montoya,R., Rodrigues,E.S., Simone Kashima, Sampaio,S.C., Coluchi,N., Leite,J., Gresh,L., Franco,L., Lourenco,J., Rico,J.M., Bispo de Filippis,A.M. and Marta Giovanetti
EPI_ISL_18103419	Undisclosed Laboratory in Brazil for the Health Emergency Information and Risk Assessment (HIM) Unit of PAHO	Undisclosed Laboratory in Brazil for the Health Emergency Information and Risk Assessment (HIM) Unit of PAHO	Hegger Fritsch, Moreno,K., Lima,I.A.B., Santos,C.S., Costa,B.G.G., de Almeida,B.L., Dos Santos,R.A., Francisco,M.V.L.O., Sampaio,M.P.S., de Mauricio LimaM., Pereira,F.M., Vagner Fonseca, Tosta,S., Joilson Xavier, de Oliveira,C., Adelino,T., de Mello,A.L.E.S., Graf,T., Luiz Carlos Junior Alcantara, Marta Giovanetti and de Siqueira,I.C.
EPI_ISL_18103420, EPI_ISL_18103421, EPI_ISL_18103422, EPI_ISL_18103423, EPI_ISL_18103424, EPI_ISL_18103425, EPI_ISL_18103426, EPI_ISL_18103427, EPI_ISL_18103428, EPI_ISL_18103429, EPI_ISL_18103430, EPI_ISL_18103431, EPI_ISL_18103432			
see above	Undisclosed Laboratory in Brazil for the Health Emergency Information and Risk Assessment (HIM) Unit of PAHO	Undisclosed Laboratory in Brazil for the Health Emergency Information and Risk Assessment (HIM) Unit of PAHO	Vazquez,C., Luiz Carlos Junior Alcantara, Vagner Fonseca, Mauricio Lima, Joilson Xavier, Adelino,T., Hegger Fritsch, Castro,E., de Oliveira,C., Schuab,G., Lima,A.R.J., Villalba,S., Gomez de la Fuente,A., Rojas,A., Cantero,C., Fleitas,F., Aquino,C., Ojeda,A., Sequera,G., Torales,J., Barrios,J., Elias,M.C., Iani,F.C.M., Ortega,M.J., Gamarra,M.L., Montoya,R., Rodrigues,E.S., Simone Kashima, Sampaio,S.C., Coluchi,N., Leite,J., Gresh,L., Franco,L., Lourenco,J., Rico,J.M., Bispo de Filippis,A.M. and Marta Giovanetti
EPI_ISL_18103433	Undisclosed Laboratory in Brazil for the Health Emergency Information and Risk Assessment (HIM) Unit of PAHO	Undisclosed Laboratory in Brazil for the Health Emergency Information and Risk Assessment (HIM) Unit of PAHO	Hegger Fritsch, Moreno,K., Lima,I.A.B., Santos,C.S., Costa,B.G.G., de Almeida,B.L., Dos Santos,R.A., Francisco,M.V.L.O., Sampaio,M.P.S., de Mauricio LimaM., Pereira,F.M., Vagner Fonseca, Tosta,S., Joilson Xavier, de Oliveira,C., Adelino,T., de Mello,A.L.E.S., Graf,T., Luiz Carlos Junior Alcantara, Marta Giovanetti and de Siqueira,I.C.
EPI_ISL_18103434, EPI_ISL_18103435, EPI_ISL_18103436, EPI_ISL_18103437, EPI_ISL_18103438, EPI_ISL_18103439, EPI_ISL_18103440	Undisclosed Laboratory in Brazil for the Health Emergency Information and Risk Assessment (HIM) Unit of PAHO	Undisclosed Laboratory in Brazil for the Health Emergency Information and Risk Assessment (HIM) Unit of PAHO	Vazquez,C., Luiz Carlos Junior Alcantara, Vagner Fonseca, Mauricio Lima, Joilson Xavier, Adelino,T., Hegger Fritsch, Castro,E., de Oliveira,C., Schuab,G., Lima,A.R.J., Villalba,S., Gomez de la Fuente,A., Rojas,A., Cantero,C., Fleitas,F., Aquino,C., Ojeda,A., Sequera,G., Torales,J., Barrios,J., Elias,M.C., Iani,F.C.M., Ortega,M.J., Gamarra,M.L., Montoya,R., Rodrigues,E.S., Simone Kashima, Sampaio,S.C., Coluchi,N., Leite,J., Gresh,L., Franco,L., Lourenco,J., Rico,J.M., Bispo de Filippis,A.M. and Marta Giovanetti
EPI_ISL_18103441	Undisclosed Laboratory in Brazil for the Health Emergency Information and Risk Assessment (HIM) Unit of PAHO	Undisclosed Laboratory in Brazil for the Health Emergency Information and Risk Assessment (HIM) Unit of PAHO	Hegger Fritsch, Moreno,K., Lima,I.A.B., Santos,C.S., Costa,B.G.G., de Almeida,B.L., Dos Santos,R.A., Francisco,M.V.L.O., Sampaio,M.P.S., de Mauricio LimaM., Pereira,F.M., Vagner Fonseca, Tosta,S., Joilson Xavier, de Oliveira,C., Adelino,T., de Mello,A.L.E.S., Graf,T., Luiz Carlos Junior Alcantara, Marta Giovanetti and de Siqueira,I.C.
EPI_ISL_18103442, EPI_ISL_18103443, EPI_ISL_18103444	Undisclosed Laboratory in Brazil for the Health Emergency Information and Risk Assessment (HIM) Unit of PAHO	Undisclosed Laboratory in Brazil for the Health Emergency Information and Risk Assessment (HIM) Unit of PAHO	Vazquez,C., Luiz Carlos Junior Alcantara, Vagner Fonseca, Mauricio Lima, Joilson Xavier, Adelino,T., Hegger Fritsch, Castro,E., de Oliveira,C., Schuab,G., Lima,A.R.J., Villalba,S., Gomez de la Fuente,A., Rojas,A., Cantero,C., Fleitas,F., Aquino,C., Ojeda,A., Sequera,G., Torales,J., Barrios,J., Elias,M.C., Iani,F.C.M., Ortega,M.J., Gamarra,M.L., Montoya,R., Rodrigues,E.S., Simone Kashima, Sampaio,S.C., Coluchi,N., Leite,J., Gresh,L., Franco,L., Lourenco,J., Rico,J.M., Bispo de Filippis,A.M. and Marta Giovanetti
EPI_ISL_18103445	Undisclosed Laboratory in Brazil for the Health Emergency Information and Risk Assessment (HIM) Unit of PAHO	Undisclosed Laboratory in Brazil for the Health Emergency Information and Risk Assessment (HIM) Unit of PAHO	Hegger Fritsch, Moreno,K., Lima,I.A.B., Santos,C.S., Costa,B.G.G., de Almeida,B.L., Dos Santos,R.A., Francisco,M.V.L.O., Sampaio,M.P.S., de Mauricio LimaM., Pereira,F.M., Vagner Fonseca, Tosta,S., Joilson Xavier, de Oliveira,C., Adelino,T., de Mello,A.L.E.S., Graf,T., Luiz Carlos Junior Alcantara, Marta Giovanetti and de Siqueira,I.C.
EPI_ISL_18103446, EPI_ISL_18103447, EPI_ISL_18103448	Undisclosed Laboratory in Brazil for the Health Emergency Information and Risk Assessment (HIM) Unit of PAHO	Undisclosed Laboratory in Brazil for the Health Emergency Information and Risk Assessment (HIM) Unit of PAHO	Vazquez,C., Luiz Carlos Junior Alcantara, Vagner Fonseca, Mauricio Lima, Joilson Xavier, Adelino,T., Hegger Fritsch, Castro,E., de Oliveira,C., Schuab,G., Lima,A.R.J., Villalba,S., Gomez de la Fuente,A., Rojas,A., Cantero,C., Fleitas,F., Aquino,C., Ojeda,A., Sequera,G., Torales,J., Barrios,J., Elias,M.C., Iani,F.C.M., Ortega,M.J., Gamarra,M.L., Montoya,R., Rodrigues,E.S., Simone Kashima, Sampaio,S.C., Coluchi,N., Leite,J., Gresh,L., Franco,L., Lourenco,J., Rico,J.M., Bispo de Filippis,A.M. and Marta Giovanetti
EPI_ISL_18103449, EPI_ISL_18103450, EPI_ISL_18103451	Undisclosed Laboratory in Brazil for the Health Emergency Information and Risk Assessment (HIM) Unit of PAHO	Undisclosed Laboratory in Brazil for the Health Emergency Information and Risk Assessment (HIM) Unit of PAHO	Hegger Fritsch, Moreno,K., Lima,I.A.B., Santos,C.S., Costa,B.G.G., de Almeida,B.L., Dos Santos,R.A., Francisco,M.V.L.O., Sampaio,M.P.S., de Mauricio LimaM., Pereira,F.M., Vagner Fonseca, Tosta,S., Joilson Xavier, de Oliveira,C., Adelino,T., de Mello,A.L.E.S., Graf,T., Luiz Carlos Junior Alcantara, Marta Giovanetti and de Siqueira,I.C.
EPI_ISL_18103452, EPI_ISL_18103453, EPI_ISL_18103454, EPI_ISL_18103455, EPI_ISL_18103456, EPI_ISL_18103457, EPI_ISL_18103458, EPI_ISL_18103459	Undisclosed Laboratory in Brazil for the Health Emergency Information and Risk Assessment (HIM) Unit of PAHO	Undisclosed Laboratory in Brazil for the Health Emergency Information and Risk Assessment (HIM) Unit of PAHO	Vazquez,C., Luiz Carlos Junior Alcantara, Vagner Fonseca, Mauricio Lima, Joilson Xavier, Adelino,T., Hegger Fritsch, Castro,E., de Oliveira,C., Schuab,G., Lima,A.R.J., Villalba,S., Gomez de la Fuente,A., Rojas,A., Cantero,C., Fleitas,F., Aquino,C., Ojeda,A., Sequera,G., Torales,J., Barrios,J., Elias,M.C., Iani,F.C.M., Ortega,M.J., Gamarra,M.L., Montoya,R., Rodrigues,E.S., Simone Kashima, Sampaio,S.C., Coluchi,N., Leite,J., Gresh,L., Franco,L., Lourenco,J., Rico,J.M., Bispo de Filippis,A.M. and Marta Giovanetti
EPI_ISL_18103460	Undisclosed Laboratory in Brazil for the Health Emergency Information and Risk Assessment (HIM) Unit of PAHO	Undisclosed Laboratory in Brazil for the Health Emergency Information and Risk Assessment (HIM) Unit of PAHO	Hegger Fritsch, Moreno,K., Lima,I.A.B., Santos,C.S., Costa,B.G.G., de Almeida,B.L., Dos Santos,R.A., Francisco,M.V.L.O., Sampaio,M.P.S., de Mauricio LimaM., Pereira,F.M., Vagner Fonseca, Tosta,S., Joilson Xavier, de Oliveira,C., Adelino,T., de Mello,A.L.E.S., Graf,T., Luiz Carlos Junior Alcantara, Marta Giovanetti and de Siqueira,I.C.
EPI_ISL_18103461, EPI_ISL_18103462, EPI_ISL_18103463	Undisclosed Laboratory in Brazil for the Health Emergency Information and Risk Assessment (HIM) Unit of PAHO	Undisclosed Laboratory in Brazil for the Health Emergency Information and Risk Assessment (HIM) Unit of PAHO	Vazquez,C., Luiz Carlos Junior Alcantara, Vagner Fonseca, Mauricio Lima, Joilson Xavier, Adelino,T., Hegger Fritsch, Castro,E., de Oliveira,C., Schuab,G., Lima,A.R.J., Villalba,S., Gomez de la Fuente,A., Rojas,A., Cantero,C., Fleitas,F., Aquino,C., Ojeda,A., Sequera,G., Torales,J., Barrios,J., Elias,M.C., Iani,F.C.M., Ortega,M.J., Gamarra,M.L., Montoya,R., Rodrigues,E.S., Simone Kashima, Sampaio,S.C., Coluchi,N., Leite,J., Gresh,L., Franco,L., Lourenco,J., Rico,J.M., Bispo de Filippis,A.M. and Marta Giovanetti
EPI_ISL_18103464	Undisclosed Laboratory in Brazil for the Health Emergency Information and Risk Assessment (HIM) Unit of PAHO	Undisclosed Laboratory in Brazil for the Health Emergency Information and Risk Assessment (HIM) Unit of PAHO	Hegger Fritsch, Moreno,K., Lima,I.A.B., Santos,C.S., Costa,B.G.G., de Almeida,B.L., Dos Santos,R.A., Francisco,M.V.L.O., Sampaio,M.P.S., de Mauricio LimaM., Pereira,F.M., Vagner Fonseca, Tosta,S., Joilson Xavier, de Oliveira,C., Adelino,T., de Mello,A.L.E.S., Graf,T., Luiz Carlos Junior Alcantara, Marta Giovanetti and de Siqueira,I.C.
EPI_ISL_18103465	Undisclosed Laboratory in Brazil for the Health Emergency Information and Risk Assessment (HIM) Unit of PAHO	Undisclosed Laboratory in Brazil for the Health Emergency Information and Risk Assessment (HIM) Unit of PAHO	Vazquez,C., Luiz Carlos Junior Alcantara, Vagner Fonseca, Mauricio Lima, Joilson Xavier, Adelino,T., Hegger Fritsch, Castro,E., de Oliveira,C., Schuab,G., Lima,A.R.J., Villalba,S., Gomez de la Fuente,A., Rojas,A., Cantero,C., Fleitas,F., Aquino,C., Ojeda,A., Sequera,G., Torales,J., Barrios,J., Elias,M.C., Iani,F.C.M., Ortega,M.J., Gamarra,M.L., Montoya,R., Rodrigues,E.S., Simone Kashima, Sampaio,S.C., Coluchi,N., Leite,J., Gresh,L., Franco,L., Lourenco,J., Rico,J.M., Bispo de Filippis,A.M. and Marta Giovanetti
EPI_ISL_18103466, EPI_ISL_18103467	Undisclosed Laboratory in Brazil for the Health Emergency Information and Risk Assessment (HIM) Unit of PAHO	Undisclosed Laboratory in Brazil for the Health Emergency Information and Risk Assessment (HIM) Unit of PAHO	Hegger Fritsch, Moreno,K., Lima,I.A.B., Santos,C.S., Costa,B.G.G., de Almeida,B.L., Dos Santos,R.A., Francisco,M.V.L.O., Sampaio,M.P.S., de Mauricio LimaM., Pereira,F.M., Vagner Fonseca, Tosta,S., Joilson Xavier, de Oliveira,C., Adelino,T., de Mello,A.L.E.S., Graf,T., Luiz Carlos Junior Alcantara, Marta Giovanetti and de Siqueira,I.C.
EPI_ISL_18103468	Undisclosed Laboratory in Brazil for the Health Emergency Information and Risk Assessment (HIM) Unit of PAHO	Undisclosed Laboratory in Brazil for the Health Emergency Information and Risk Assessment (HIM) Unit of PAHO	Vazquez,C., Luiz Carlos Junior Alcantara, Vagner Fonseca, Mauricio Lima, Joilson Xavier, Adelino,T., Hegger Fritsch, Castro,E., de Oliveira,C., Schuab,G., Lima,A.R.J., Villalba,S., Gomez de la Fuente,A., Rojas,A., Cantero,C., Fleitas,F., Aquino,C., Ojeda,A., Sequera,G., Torales,J., Barrios,J., Elias,M.C., Iani,F.C.M., Ortega,M.J., Gamarra,M.L., Montoya,R., Rodrigues,E.S., Simone Kashima, Sampaio,S.C., Coluchi,N., Leite,J., Gresh,L., Franco,L., Lourenco,J., Rico,J.M., Bispo de Filippis,A.M. and Marta Giovanetti
EPI_ISL_18103469	Undisclosed Laboratory in Brazil for the Health Emergency Information and Risk Assessment (HIM) Unit of PAHO	Undisclosed Laboratory in Brazil for the Health Emergency Information and Risk Assessment (HIM) Unit of PAHO	Hegger Fritsch, Moreno,K., Lima,I.A.B., Santos,C.S., Costa,B.G.G., de Almeida,B.L., Dos Santos,R.A., Francisco,M.V.L.O., Sampaio,M.P.S., de Mauricio LimaM., Pereira,F.M., Vagner Fonseca, Tosta,S., Joilson Xavier, de Oliveira,C., Adelino,T., de Mello,A.L.E.S., Graf,T., Luiz Carlos Junior Alcantara, Marta Giovanetti and de Siqueira,I.C.
EPI_ISL_18103470, EPI_ISL_18103471, EPI_ISL_18103472	Undisclosed Laboratory in Brazil for the Health Emergency Information and Risk Assessment (HIM) Unit of PAHO	Undisclosed Laboratory in Brazil for the Health Emergency Information and Risk Assessment (HIM) Unit of PAHO	Vazquez,C., Luiz Carlos Junior Alcantara, Vagner Fonseca, Mauricio Lima, Joilson Xavier, Adelino,T., Hegger Fritsch, Castro,E., de Oliveira,C., Schuab,G., Lima,A.R.J., Villalba,S., Gomez de la Fuente,A., Rojas,A., Cantero,C., Fleitas,F., Aquino,C., Ojeda,A., Sequera,G., Torales,J., Barrios,J., Elias,M.C., Iani,F.C.M., Ortega,M.J., Gamarra,M.L., Montoya,R., Rodrigues,E.S., Simone Kashima, Sampaio,S.C., Coluchi,N., Leite,J., Gresh,L., Franco,L., Lourenco,J., Rico,J.M., Bispo de Filippis,A.M. and Marta Giovanetti
EPI_ISL_18103473	Undisclosed Laboratory in Brazil for the Health Emergency Information and Risk Assessment (HIM) Unit of PAHO	Undisclosed Laboratory in Brazil for the Health Emergency Information and Risk Assessment (HIM) Unit of PAHO	Hegger Fritsch, Moreno,K., Lima,I.A.B., Santos,C.S., Costa,B.G.G., de Almeida,B.L., Dos Santos,R.A., Francisco,M.V.L.O., Sampaio,M.P.S., de Mauricio LimaM., Pereira,F.M., Vagner Fonseca, Tosta,S., Joilson Xavier, de Oliveira,C., Adelino,T., de Mello,A.L.E.S., Graf,T., Luiz Carlos Junior Alcantara, Marta Giovanetti and de Siqueira,I.C.
EPI_ISL_18103474, EPI_ISL_18103475	Undisclosed Laboratory in Brazil for the Health Emergency Information and Risk Assessment (HIM) Unit of PAHO	Undisclosed Laboratory in Brazil for the Health Emergency Information and Risk Assessment (HIM) Unit of PAHO	Vazquez,C., Luiz Carlos Junior Alcantara, Vagner Fonseca, Mauricio Lima, Joilson Xavier, Adelino,T., Hegger Fritsch, Castro,E., de Oliveira,C., Schuab,G., Lima,A.R.J., Villalba,S., Gomez de la Fuente,A., Rojas,A., Cantero,C., Fleitas,F., Aquino,C., Ojeda,A., Sequera,G., Torales,J., Barrios,J., Elias,M.C., Iani,F.C.M., Ortega,M.J., Gamarra,M.L., Montoya,R., Rodrigues,E.S., Simone Kashima, Sampaio,S.C., Coluchi,N., Leite,J., Gresh,L., Franco,L., Lourenco,J., Rico,J.M., Bispo de Filippis,A.M. and Marta Giovanetti
EPI_ISL_18103476	Undisclosed Laboratory in Brazil for the Health Emergency Information and Risk Assessment (HIM) Unit of PAHO	Undisclosed Laboratory in Brazil for the Health Emergency Information and Risk Assessment (HIM) Unit of PAHO	Hegger Fritsch, Moreno,K., Lima,I.A.B., Santos,C.S., Costa,B.G.G., de Almeida,B.L., Dos Santos,R.A., Francisco,M.V.L.O., Sampaio,M.P.S., de Mauricio LimaM., Pereira,F.M., Vagner Fonseca, Tosta,S., Joilson Xavier, de Oliveira,C., Adelino,T., de Mello,A.L.E.S., Graf,T., Luiz Carlos Junior Alcantara, Marta Giovanetti and de Siqueira,I.C.

EPI_ISL_18103477, EPI_ISL_18103478, EPI_ISL_18103479, EPI_ISL_18103480, EPI_ISL_18103481, EPI_ISL_18103482, EPI_ISL_18103483, EPI_ISL_18103484, EPI_ISL_18103485, EPI_ISL_18103486, EPI_ISL_18103487, EPI_ISL_18103488, EPI_ISL_18103489			
see above	Undisclosed Laboratory in Brazil for the Health Emergency Information and Risk Assessment (HIM) Unit of PAHO	Undisclosed Laboratory in Brazil for the Health Emergency Information and Risk Assessment (HIM) Unit of PAHO	Vazquez,C., Luiz Carlos Junior Alcantara, Vagner Fonseca, Mauricio Lima, Joilson Xavier, Adelino,T., Hegger Fritsch, Castro,E., de Oliveira,C., Schuab,G., Lima,A.R.J., Villalba,S., Gomez de la Fuente,A., Rojas,A., Cantero,C., Fleitas,F., Aquino,C., Ojeda,A., Sequera,G., Torales,J., Barrios,J., Elias,M.C., Iani,F.C.M., Ortega,M.J., Gamarra,M.L., Montoya,R., Rodrigues,E.S., Simone Kashima, Sampaio,S.C., Coluchi,N., Leite,J., Gresh,L., Franco,L., Lourenco,J., Rico,J.M., Bispo de Filippis,A.M. and Marta Giovanetti
EPI_ISL_18103490	Undisclosed Laboratory in Brazil for the Health Emergency Information and Risk Assessment (HIM) Unit of PAHO	Undisclosed Laboratory in Brazil for the Health Emergency Information and Risk Assessment (HIM) Unit of PAHO	Hegger Fritsch, Moreno,K., Lima,I.A.B., Santos,C.S., Costa,B.G.G., de Almeida,B.L., Dos Santos,R.A., Francisco,M.V.L.O., Sampaio,M.P.S., de Mauricio LimaM., Pereira,F.M., Vagner Fonseca, Tosta,S., Joilson Xavier, de Oliveira,C., Adelino,T., de Mello,A.L.E.S., Graf,T., Luiz Carlos Junior Alcantara, Marta Giovanetti and de Siqueira,I.C.
EPI_ISL_18103491, EPI_ISL_18103492, EPI_ISL_18103493, EPI_ISL_18103494, EPI_ISL_18103495, EPI_ISL_18103496, EPI_ISL_18103497, EPI_ISL_18103498, EPI_ISL_18103499, EPI_ISL_18103500, EPI_ISL_18103501, EPI_ISL_18103502, EPI_ISL_18103503, EPI_ISL_18103504			
see above	Undisclosed Laboratory in Brazil for the Health Emergency Information and Risk Assessment (HIM) Unit of PAHO	Undisclosed Laboratory in Brazil for the Health Emergency Information and Risk Assessment (HIM) Unit of PAHO	Vazquez,C., Luiz Carlos Junior Alcantara, Vagner Fonseca, Mauricio Lima, Joilson Xavier, Adelino,T., Hegger Fritsch, Castro,E., de Oliveira,C., Schuab,G., Lima,A.R.J., Villalba,S., Gomez de la Fuente,A., Rojas,A., Cantero,C., Fleitas,F., Aquino,C., Ojeda,A., Sequera,G., Torales,J., Barrios,J., Elias,M.C., Iani,F.C.M., Ortega,M.J., Gamarra,M.L., Montoya,R., Rodrigues,E.S., Simone Kashima, Sampaio,S.C., Coluchi,N., Leite,J., Gresh,L., Franco,L., Lourenco,J., Rico,J.M., Bispo de Filippis,A.M. and Marta Giovanetti
EPI_ISL_18103505	Undisclosed Laboratory in Brazil for the Health Emergency Information and Risk Assessment (HIM) Unit of PAHO	Undisclosed Laboratory in Brazil for the Health Emergency Information and Risk Assessment (HIM) Unit of PAHO	Hegger Fritsch, Moreno,K., Lima,I.A.B., Santos,C.S., Costa,B.G.G., de Almeida,B.L., Dos Santos,R.A., Francisco,M.V.L.O., Sampaio,M.P.S., de Mauricio LimaM., Pereira,F.M., Vagner Fonseca, Tosta,S., Joilson Xavier, de Oliveira,C., Adelino,T., de Mello,A.L.E.S., Graf,T., Luiz Carlos Junior Alcantara, Marta Giovanetti and de Siqueira,I.C.
EPI_ISL_18103506	Undisclosed Laboratory in Brazil for the Health Emergency Information and Risk Assessment (HIM) Unit of PAHO	Undisclosed Laboratory in Brazil for the Health Emergency Information and Risk Assessment (HIM) Unit of PAHO	Vazquez,C., Luiz Carlos Junior Alcantara, Vagner Fonseca, Mauricio Lima, Joilson Xavier, Adelino,T., Hegger Fritsch, Castro,E., de Oliveira,C., Schuab,G., Lima,A.R.J., Villalba,S., Gomez de la Fuente,A., Rojas,A., Cantero,C., Fleitas,F., Aquino,C., Ojeda,A., Sequera,G., Torales,J., Barrios,J., Elias,M.C., Iani,F.C.M., Ortega,M.J., Gamarra,M.L., Montoya,R., Rodrigues,E.S., Simone Kashima, Sampaio,S.C., Coluchi,N., Leite,J., Gresh,L., Franco,L., Lourenco,J., Rico,J.M., Bispo de Filippis,A.M. and Marta Giovanetti
EPI_ISL_18103507	Undisclosed Laboratory in Brazil for the Health Emergency Information and Risk Assessment (HIM) Unit of PAHO	Undisclosed Laboratory in Brazil for the Health Emergency Information and Risk Assessment (HIM) Unit of PAHO	Hegger Fritsch, Moreno,K., Lima,I.A.B., Santos,C.S., Costa,B.G.G., de Almeida,B.L., Dos Santos,R.A., Francisco,M.V.L.O., Sampaio,M.P.S., de Mauricio LimaM., Pereira,F.M., Vagner Fonseca, Tosta,S., Joilson Xavier, de Oliveira,C., Adelino,T., de Mello,A.L.E.S., Graf,T., Luiz Carlos Junior Alcantara, Marta Giovanetti and de Siqueira,I.C.
EPI_ISL_18103508, EPI_ISL_18103509	Undisclosed Laboratory in Brazil for the Health Emergency Information and Risk Assessment (HIM) Unit of PAHO	Undisclosed Laboratory in Brazil for the Health Emergency Information and Risk Assessment (HIM) Unit of PAHO	Vazquez,C., Luiz Carlos Junior Alcantara, Vagner Fonseca, Mauricio Lima, Joilson Xavier, Adelino,T., Hegger Fritsch, Castro,E., de Oliveira,C., Schuab,G., Lima,A.R.J., Villalba,S., Gomez de la Fuente,A., Rojas,A., Cantero,C., Fleitas,F., Aquino,C., Ojeda,A., Sequera,G., Torales,J., Barrios,J., Elias,M.C., Iani,F.C.M., Ortega,M.J., Gamarra,M.L., Montoya,R., Rodrigues,E.S., Simone Kashima, Sampaio,S.C., Coluchi,N., Leite,J., Gresh,L., Franco,L., Lourenco,J., Rico,J.M., Bispo de Filippis,A.M. and Marta Giovanetti
EPI_ISL_18103510	Undisclosed Laboratory in Brazil for the Health Emergency Information and Risk Assessment (HIM) Unit of PAHO	Undisclosed Laboratory in Brazil for the Health Emergency Information and Risk Assessment (HIM) Unit of PAHO	Hegger Fritsch, Moreno,K., Lima,I.A.B., Santos,C.S., Costa,B.G.G., de Almeida,B.L., Dos Santos,R.A., Francisco,M.V.L.O., Sampaio,M.P.S., de Mauricio LimaM., Pereira,F.M., Vagner Fonseca, Tosta,S., Joilson Xavier, de Oliveira,C., Adelino,T., de Mello,A.L.E.S., Graf,T., Luiz Carlos Junior Alcantara, Marta Giovanetti and de Siqueira,I.C.
EPI_ISL_18103511, EPI_ISL_18103512, EPI_ISL_18103513, EPI_ISL_18103514, EPI_ISL_18103515, EPI_ISL_18103516, EPI_ISL_18103517, EPI_ISL_18103518, EPI_ISL_18103519, EPI_ISL_18103520, EPI_ISL_18103521, EPI_ISL_18103522			
see above	Undisclosed Laboratory in Brazil for the Health Emergency Information and Risk Assessment (HIM) Unit of PAHO	Undisclosed Laboratory in Brazil for the Health Emergency Information and Risk Assessment (HIM) Unit of PAHO	Vazquez,C., Luiz Carlos Junior Alcantara, Vagner Fonseca, Mauricio Lima, Joilson Xavier, Adelino,T., Hegger Fritsch, Castro,E., de Oliveira,C., Schuab,G., Lima,A.R.J., Villalba,S., Gomez de la Fuente,A., Rojas,A., Cantero,C., Fleitas,F., Aquino,C., Ojeda,A., Sequera,G., Torales,J., Barrios,J., Elias,M.C., Iani,F.C.M., Ortega,M.J., Gamarra,M.L., Montoya,R., Rodrigues,E.S., Simone Kashima, Sampaio,S.C., Coluchi,N., Leite,J., Gresh,L., Franco,L., Lourenco,J., Rico,J.M., Bispo de Filippis,A.M. and Marta Giovanetti
EPI_ISL_18103523	Undisclosed Laboratory in Brazil for the Health Emergency Information and Risk Assessment (HIM) Unit of PAHO	Undisclosed Laboratory in Brazil for the Health Emergency Information and Risk Assessment (HIM) Unit of PAHO	Hegger Fritsch, Moreno,K., Lima,I.A.B., Santos,C.S., Costa,B.G.G., de Almeida,B.L., Dos Santos,R.A., Francisco,M.V.L.O., Sampaio,M.P.S., de Mauricio LimaM., Pereira,F.M., Vagner Fonseca, Tosta,S., Joilson Xavier, de Oliveira,C., Adelino,T., de Mello,A.L.E.S., Graf,T., Luiz Carlos Junior Alcantara, Marta Giovanetti and de Siqueira,I.C.
EPI_ISL_18103524, EPI_ISL_18103525, EPI_ISL_18103526, EPI_ISL_18103527, EPI_ISL_18103528, EPI_ISL_18103529, EPI_ISL_18103530, EPI_ISL_18103531, EPI_ISL_18103532, EPI_ISL_18103533	Undisclosed Laboratory in Brazil for the Health Emergency Information and Risk Assessment (HIM) Unit of PAHO	Undisclosed Laboratory in Brazil for the Health Emergency Information and Risk Assessment (HIM) Unit of PAHO	Vazquez,C., Luiz Carlos Junior Alcantara, Vagner Fonseca, Mauricio Lima, Joilson Xavier, Adelino,T., Hegger Fritsch, Castro,E., de Oliveira,C., Schuab,G., Lima,A.R.J., Villalba,S., Gomez de la Fuente,A., Rojas,A., Cantero,C., Fleitas,F., Aquino,C., Ojeda,A., Sequera,G., Torales,J., Barrios,J., Elias,M.C., Iani,F.C.M., Ortega,M.J., Gamarra,M.L., Montoya,R., Rodrigues,E.S., Simone Kashima, Sampaio,S.C., Coluchi,N., Leite,J., Gresh,L., Franco,L., Lourenco,J., Rico,J.M., Bispo de Filippis,A.M. and Marta Giovanetti
EPI_ISL_18358948, EPI_ISL_18358949, EPI_ISL_18358950, EPI_ISL_18358951, EPI_ISL_18358952, EPI_ISL_18358953, EPI_ISL_18358954, EPI_ISL_18358955, EPI_ISL_18358956, EPI_ISL_18358957, EPI_ISL_18358958, EPI_ISL_18358959, EPI_ISL_18358960, EPI_ISL_18358961, EPI_ISL_18358962, EPI_ISL_18358963, EPI_ISL_18358964, EPI_ISL_18358965, EPI_ISL_18358966, EPI_ISL_18358967, EPI_ISL_18358968, EPI_ISL_18358969, EPI_ISL_18358970, EPI_ISL_18358971, EPI_ISL_18358972, EPI_ISL_18358973, EPI_ISL_18358974, EPI_ISL_18358975, EPI_ISL_18358976, EPI_ISL_18358977, EPI_ISL_18358978, EPI_ISL_18358979			
see above	Institut Pasteur de la Guadeloupe	Institut Pasteur de la Guadeloupe	Garcia-Van Smevoorde,M., Piorkowski,G., Emboule,L., Dos Santos,G., Loraux,C., Guyomard-Rabenirina,S., Joannes,M.O., Fagour,L., Najioullah,F., Cabie,A., de Lamballerie,X., Vega-Rua,A., Cesaire,R. and Calvez,E.
EPI_ISL_18360767, EPI_ISL_18360768, EPI_ISL_18360769, EPI_ISL_18360770, EPI_ISL_18360771, EPI_ISL_18360772, EPI_ISL_18360773, EPI_ISL_18360774, EPI_ISL_18360775, EPI_ISL_18360776, EPI_ISL_18360777, EPI_ISL_18360778, EPI_ISL_18360779, EPI_ISL_18360780, EPI_ISL_18360781, EPI_ISL_18360782, EPI_ISL_18360783, EPI_ISL_18360784, EPI_ISL_18360785, EPI_ISL_18360786, EPI_ISL_18360787, EPI_ISL_18360788, EPI_ISL_18360789, EPI_ISL_18360790, EPI_ISL_18360791, EPI_ISL_18360792, EPI_ISL_18360793, EPI_ISL_18360794, EPI_ISL_18360795, EPI_ISL_18360796, EPI_ISL_18360797, EPI_ISL_18360798, EPI_ISL_18360799, EPI_ISL_18360800, EPI_ISL_18360801, EPI_ISL_18360802, EPI_ISL_18360803, EPI_ISL_18360804, EPI_ISL_18360805, EPI_ISL_18360806, EPI_ISL_18360807, EPI_ISL_18360808, EPI_ISL_18360809, EPI_ISL_18360810, EPI_ISL_18360811, EPI_ISL_18360812, EPI_ISL_18360813, EPI_ISL_18360814, EPI_ISL_18360815, EPI_ISL_18360816, EPI_ISL_18360817, EPI_ISL_18360818, EPI_ISL_18360819, EPI_ISL_18360820, EPI_ISL_18360821, EPI_ISL_18360822, EPI_ISL_18360823, EPI_ISL_18360824, EPI_ISL_18360825, EPI_ISL_18360826, EPI_ISL_18360827, EPI_ISL_18360828, EPI_ISL_18360829, EPI_ISL_18360830, EPI_ISL_18360831, EPI_ISL_18360832, EPI_ISL_18360833, EPI_ISL_18360834, EPI_ISL_18360835, EPI_ISL_18360836, EPI_ISL_18360837, EPI_ISL_18360838, EPI_ISL_18360839, EPI_ISL_18360840, EPI_ISL_18360841, EPI_ISL_18360842, EPI_ISL_18360843, EPI_ISL_18360844, EPI_ISL_18360845, EPI_ISL_18360846, EPI_ISL_18360847, EPI_ISL_18360848, EPI_ISL_18360849, EPI_ISL_18360850, EPI_ISL_18360851, EPI_ISL_18360852, EPI_ISL_18360853, EPI_ISL_18360854, EPI_ISL_18360855, EPI_ISL_18360856, EPI_ISL_18360857, EPI_ISL_18360858, EPI_ISL_18360859, EPI_ISL_18360860, EPI_ISL_18360861, EPI_ISL_18360862, EPI_ISL_18360863, EPI_ISL_18360864, EPI_ISL_18360865, EPI_ISL_18360866, EPI_ISL_18360867, EPI_ISL_18360868, EPI_ISL_18360869, EPI_ISL_18360870, EPI_ISL_18360871, EPI_ISL_18360872, EPI_ISL_18360873, EPI_ISL_18360874, EPI_ISL_18360875, EPI_ISL_18360876, EPI_ISL_18360877, EPI_ISL_18360878, EPI_ISL_18360879, EPI_ISL_18360880, EPI_ISL_18360881, EPI_ISL_18360882, EPI_ISL_18360883, EPI_ISL_18360884, EPI_ISL_18360885, EPI_ISL_18360886, EPI_ISL_18360887, EPI_ISL_18360888, EPI_ISL_18360889, EPI_ISL_18360890, EPI_ISL_18360891, EPI_ISL_18360892, EPI_ISL_18360893, EPI_ISL_18360894, EPI_ISL_18360895, EPI_ISL_18360896, EPI_ISL_18360897, EPI_ISL_18360898, EPI_ISL_18360899, EPI_ISL_18360900, EPI_ISL_18360901, EPI_ISL_18360902, EPI_ISL_18360903, EPI_ISL_18360904, EPI_ISL_18360905, EPI_ISL_18360906, EPI_ISL_18360907, EPI_ISL_18360908, EPI_ISL_18360909, EPI_ISL_18360910, EPI_ISL_18360911, EPI_ISL_18360912, EPI_ISL_18360913, EPI_ISL_18360914, EPI_ISL_18360915, EPI_ISL_18360916, EPI_ISL_18360917, EPI_ISL_18360918, EPI_ISL_18360919, EPI_ISL_18360920, EPI_ISL_18360921, EPI_ISL_18360922, EPI_ISL_18360923, EPI_ISL_18360924, EPI_ISL_18360925, EPI_ISL_18360926, EPI_ISL_18360927, EPI_ISL_18360928, EPI_ISL_18360929, EPI_ISL_18360930, EPI_ISL_18360931, EPI_ISL_18360932, EPI_ISL_18360933, EPI_ISL_18360934, EPI_ISL_18360935, EPI_ISL_18360936, EPI_ISL_18360937, EPI_ISL_18360938, EPI_ISL_18360939, EPI_ISL_18360940, EPI_ISL_18360941, EPI_ISL_18360942, EPI_ISL_18360943			
see above	Laboratório Central de Saúde Pública do Estado de Mato Grosso do Sul	Laboratório Central de Saúde Pública do Estado de Mato Grosso do Sul	Larissa Domingues Castilho de Arruda, Marta Giovanetti, Vagner Fonseca, Marina Castilhos Souza Umaki Zardin, Gislene Garcia de Castro Lichs, Silvia Asato, Ana Olivia Pascoto Esposito, Miriam Tokeshi Müller, Joilson Xavier, Hegger Fritsch, Mauricio Lima, Carla de Oliveira, Elaine Vieira Santos, Livia de Mello Almeida Maziero, Danila Fernanda Rodrigues Frias, Danielle Ahad das Neves, Liliane Ferreira da Silva, Ellen Caroline Rodrigues Barretos, Paulo Eduardo Tsuha Oshiro, Bianca Modafari Goday, Jessica Klener Lemos dos Santos, Simone Kashima, Carlos F. C. de Albuquerque, Rodrigo Fabiano do Carmo Said, Alexander Rosewell, Luiz Henrique Ferraz Demarchi, Julio Croda, Luiz Carlos Junior Alcantara and Crhistinne Cavalheiro Maymone Gonçalves
EPI_ISL_18414933	National University of Singapore	National University of Singapore	Chin,W.X., Teo,Z.Y. and Chu,J.J.H.
EPI_ISL_18474688, EPI_ISL_18474689	Universidad de Antioquia	Universidad de Antioquia	Ciuoderis,K.A., Usuga,J., Moreno-Lopez,I., Perez-Restrepo,L.S., Florez,D.Y., Hernandez-Ortiz,J.P., Cloherty,G.A. and Osorio,J.E.
EPI_ISL_18474690	Universidad de Antioquia	Universidad de Antioquia	Ciuoderis,K.A., Moreno-Lopez,I., Usuga-Restrepo,J.A., Perez-Restrepo,L.S., Florez,D.Y., Hernandez-Ortiz,J.P., Cloherty,G.A. and Osorio,J.E.
EPI_ISL_18474691	Universidad de Antioquia	Universidad de Antioquia	Ciuoderis,K.A., Usuga,J., Moreno-Lopez,I., Perez-Restrepo,L.S., Florez,D.Y., Hernandez-Ortiz,J.P., Cloherty,G.A. and Osorio,J.E.
EPI_ISL_18474692	Universidad de Antioquia	Universidad de Antioquia	Ciuoderis,K., Usuga,J., Moreno-Lopez,I., Perez-Restrepo,L., Florez,D., Hernandez-Ortiz,J., Cloherty,G. and Osorio,J.
EPI_ISL_18474693, EPI_ISL_18474694	Universidad de Antioquia	Universidad de Antioquia	Ciuoderis,K.A., Moreno-Lopez,I., Usuga-Restrepo,J.A., Perez-Restrepo,L.S., Florez,D.Y., Hernandez-Ortiz,J.P., Cloherty,G.A. and Osorio,J.E.
EPI_ISL_18474695, EPI_ISL_18474696, EPI_ISL_18474697, EPI_ISL_18474698, EPI_ISL_18474699, EPI_ISL_18474700, EPI_ISL_18474701	Universidad de Antioquia	Universidad de Antioquia	Ciuoderis,K.A., Usuga,J., Moreno-Lopez,I., Perez-Restrepo,L.S., Florez,D.Y., Hernandez-Ortiz,J.P., Cloherty,G.A. and Osorio,J.E.
EPI_ISL_18474702	Universidad de Antioquia	Universidad de Antioquia	Ciuoderis,K.A., Moreno-Lopez,I., Usuga-Restrepo,J.A., Perez-Restrepo,L.S., Florez,D.Y., Hernandez-Ortiz,J.P., Cloherty,G.A. and Osorio,J.E.
EPI_ISL_18474703, EPI_ISL_18474704, EPI_ISL_18474705, EPI_ISL_18474706, EPI_ISL_18474707, EPI_ISL_18474708	Universidad de Antioquia	Universidad de Antioquia	Ciuoderis,K.A., Usuga,J., Moreno-Lopez,I., Perez-Restrepo,L.S., Florez,D.Y., Hernandez-Ortiz,J.P., Cloherty,G.A. and Osorio,J.E.
EPI_ISL_18474709	Universidad de Antioquia	Universidad de Antioquia	Ciuoderis,K.A., Moreno-Lopez,I., Usuga-Restrepo,J.A., Perez-Restrepo,L.S., Florez,D.Y., Hernandez-Ortiz,J.P., Cloherty,G.A. and Osorio,J.E.
EPI_ISL_18474710	Universidad de Antioquia	Universidad de Antioquia	Ciuoderis,K.A., Usuga,J., Moreno-Lopez,I., Perez-Restrepo,L.S., Florez,D.Y., Hernandez-Ortiz,J.P., Cloherty,G.A. and Osorio,J.E.
EPI_ISL_18474711, EPI_ISL_18474712, EPI_ISL_18474713, EPI_ISL_18474714, EPI_ISL_18474715	Universidad de Antioquia	Universidad de Antioquia	Ciuoderis,K.A., Moreno-Lopez,I., Usuga-Restrepo,J.A., Perez-Restrepo,L.S., Florez,D.Y., Hernandez-Ortiz,J.P., Cloherty,G.A. and Osorio,J.E.
EPI_ISL_18474716, EPI_ISL_18474717, EPI_ISL_18474718	Universidad de Antioquia	Universidad de Antioquia	Ciuoderis,K.A., Usuga,J., Moreno-Lopez,I., Perez-Restrepo,L.S., Florez,D.Y., Hernandez-Ortiz,J.P., Cloherty,G.A. and Osorio,J.E.
EPI_ISL_18474719	Universidad de Antioquia	Universidad de Antioquia	Ciuoderis,K.A., Moreno-Lopez,I., Usuga-Restrepo,J.A., Perez-Restrepo,L.S., Florez,D.Y., Hernandez-Ortiz,J.P., Cloherty,G.A. and Osorio,J.E.
EPI_ISL_18474720, EPI_ISL_18474721, EPI_ISL_18474722, EPI_ISL_18474723, EPI_ISL_18474724	Universidad de Antioquia	Universidad de Antioquia	Ciuoderis,K.A., Usuga,J., Moreno-Lopez,I., Perez-Restrepo,L.S., Florez,D.Y., Hernandez-Ortiz,J.P., Cloherty,G.A. and Osorio,J.E.
EPI_ISL_18474725	Universidad de Antioquia	Universidad de Antioquia	Ciuoderis,K.A., Moreno-Lopez,I., Usuga-Restrepo,J.A., Perez-Restrepo,L.S., Florez,D.Y., Hernandez-Ortiz,J.P., Cloherty,G.A. and Osorio,J.E.
EPI_ISL_18474726, EPI_ISL_18474727, EPI_ISL_18474728, EPI_ISL_18474729	Universidad de Antioquia	Universidad de Antioquia	Ciuoderis,K.A., Usuga,J., Moreno-Lopez,I., Perez-Restrepo,L.S., Florez,D.Y., Hernandez-Ortiz,J.P., Cloherty,G.A. and Osorio,J.E.
EPI_ISL_18474730, EPI_ISL_18474736, EPI_ISL_18474752, EPI_ISL_18474770, EPI_ISL_18474787, EPI_ISL_18474814, EPI_ISL_18474819, EPI_ISL_18474834	unknown	unknown	Fonseca,V., Giovanetti,M., Xavier,J., Oliveira,C., Lima,M., Barbosa,E., Guimaraes,N., Adelino,T., Fritsch,H., Mendonca,M., Bispo Filippis,A. and Alcantara,L.
EPI_ISL_18560746, EPI_ISL_18560747, EPI_ISL_18560748	NC - Biological Sciences,NC - Redeemer's University	NC - Biological Sciences,NC - Redeemer's University	Onoja,B.A., Oguzie,J.U., George,U.E., Asoh,K.E., Ajayi,P., Omofaye,T.F., Igeleke,I.O., Eromon,P., Harouna,S., Parker,E., Adeniji,A.J. and Happi,C.T.
EPI_ISL_18579214, EPI_ISL_18579215, EPI_ISL_18579216, EPI_ISL_18579217, EPI_ISL_18579218, EPI_ISL_18579219, EPI_ISL_18579220, EPI_ISL_18579221, EPI_ISL_18579222, EPI_ISL_18579223, EPI_ISL_18579224, EPI_ISL_18579225, EPI_ISL_18579226, EPI_ISL_18579227, EPI_ISL_18579228, EPI_ISL_18579229, EPI_ISL_18579230, EPI_ISL_18579231, EPI_ISL_18579232, EPI_ISL_18579233, EPI_ISL_18579234, EPI_ISL_18579235, EPI_ISL_18579236, EPI_ISL_18579237, EPI_ISL_18579238, EPI_ISL_18579239, EPI_ISL_18579240, EPI_ISL_18579241, EPI_ISL_18579242, EPI_ISL_18579243, EPI_ISL_18579244, EPI_ISL_18579245, EPI_ISL_18579246, EPI_ISL_18579247, EPI_ISL_18579248, EPI_ISL_18579249, EPI_ISL_18579250, EPI_ISL_18579251, EPI_ISL_18579252, EPI_ISL_18579253, EPI_ISL_18579254, EPI_ISL_18579255, EPI_ISL_18579256, EPI_ISL_18579257, EPI_ISL_18579258, EPI_ISL_18579259, EPI_ISL_18579260, EPI_ISL_18579261, EPI_ISL_18579262, EPI_ISL_18579263, EPI_ISL_18579264, EPI_ISL_18579265, EPI_ISL_18579266, EPI_ISL_18579267, EPI_ISL_18579268, EPI_ISL_18579269, EPI_ISL_18579270, EPI_ISL_18579271, EPI_ISL_18579272, EPI_ISL_18579273, EPI_ISL_18579274, EPI_ISL_18579275, EPI_ISL_18579276, EPI_ISL_18579277, EPI_ISL_18579278, EPI_ISL_18579279, EPI_ISL_18579280			
see above	University of Western Australia	University of Western Australia	Harapan,H., Ernst,T., Panta,K., McCarthy,S., Michie,A., Sasmono,R.T., Smith,D.W., Effler,P.V. and Imrie,A.
EPI_ISL_18607159, EPI_ISL_18607160, EPI_ISL_18607161, EPI_ISL_18607162, EPI_ISL_18607163, EPI_ISL_18607164, EPI_ISL_18607165, EPI_ISL_18607166, EPI_ISL_18607167	Oswaldo Cruz Foundation, Instituto Carlos Chagas	Oswaldo Cruz Foundation, Instituto Carlos Chagas	de Arruda,T.B., Bavia,L., Mosimann,A.L.P., Aoki,M.N., Sarzi,M.L., Conchon-Costa,I., Wowk,P.F., Duarte Dos Santos,C.N., Pavanelli,W.R., Silveira,G.F. and Bordignon,J.
EPI_ISL_18689287, EPI_ISL_18689288, EPI_ISL_18689289, EPI_ISL_18689290, EPI_ISL_18689291, EPI_ISL_18689292, EPI_ISL_18689293, EPI_ISL_18689294, EPI_ISL_18689295, EPI_ISL_18689296, EPI_ISL_18689297, EPI_ISL_18689298, EPI_ISL_18689299, EPI_ISL_18689300, EPI_ISL_18689301, EPI_ISL_18689302, EPI_ISL_18689303, EPI_ISL_18689304, EPI_ISL_18689305, EPI_ISL_18689306, EPI_ISL_18689307, EPI_ISL_18689308, EPI_ISL_18689309, EPI_ISL_18689310, EPI_ISL_18689311, EPI_ISL_18689312, EPI_ISL_18689313, EPI_ISL_18689314, EPI_ISL_18689315, EPI_ISL_18689316, EPI_ISL_18689317, EPI_ISL_18689318, EPI_ISL_18689319, EPI_ISL_18689320, EPI_ISL_18689321, EPI_ISL_18689322, EPI_ISL_18689323, EPI_ISL_18689324, EPI_ISL_18689325, EPI_ISL_18689326, EPI_ISL_18689327, EPI_ISL_18689328, EPI_ISL_18689329, EPI_ISL_18689330, EPI_ISL_18689331, EPI_ISL_18689332, EPI_ISL_18689333, EPI_ISL_18689334, EPI_ISL_18689335, EPI_ISL_18689336, EPI_ISL_18689337, EPI_ISL_18689338, EPI_ISL_18689339, EPI_ISL_18689340, EPI_ISL_18689341, EPI_ISL_18689342, EPI_ISL_18689343, EPI_ISL_18689344, EPI_ISL_18689345, EPI_ISL_18689346, EPI_ISL_18689347, EPI_ISL_18689348			

see above	Instituto de Investigaciones en Ciencias de la Salud, Universidad Nacional de Asuncion, Production Department	Instituto de Investigaciones en Ciencias de la Salud, Universidad Nacional de Asuncion, Production Department	Rojas,A., Shen,J., Cardozo,F., Bernal,C., Caballero,O., Ping,S., Key,A., Haider,A., Stittleburg,V., de Guillen,Y., Langjahr,P., Acosta,M.E., Aria,L., Mendoza,L., Paez,M., Von-Horoch,M., Luraschi,P., Cabral,S., Sanchez,M.C., Torres,A., Pinsky,B.A., Piantadosi,A. and Waggoner,J.
EPI_ISL_18689389, EPI_ISL_18689392, EPI_ISL_18689398, EPI_ISL_18689404, EPI_ISL_18689409, EPI_ISL_18689417, EPI_ISL_18689422, EPI_ISL_18689424	University of Sao Paulo	University of Sao Paulo	Souza,C. and Romano,C.
EPI_ISL_18708170, EPI_ISL_18708171, EPI_ISL_18708172, EPI_ISL_18708173, EPI_ISL_18708174, EPI_ISL_18708175, EPI_ISL_18708176, EPI_ISL_18708177, EPI_ISL_18708178, EPI_ISL_18708179, EPI_ISL_18708180, EPI_ISL_18708181, EPI_ISL_18708182			
see above	University of Bengkulu, Microbiology	University of Bengkulu, Microbiology	Nugraheni,E., Syahrurachman,A., Nainggolan,L., Sipriyadi,S., Sariyanti,M., Nugroho,M.A., Fithriyah,F. and Dewi,B.E.
EPI_ISL_18708233, EPI_ISL_18708234, EPI_ISL_18708235, EPI_ISL_18708236, EPI_ISL_18708237	Viral Immunology Laboratory, Oswaldo Cruz Institute	Viral Immunology Laboratory, Oswaldo Cruz Institute	de Mendonca,M.C.L., Oliveira,C., Rodrigues,C.D.S., Santos,C.S., Sampaio,S.A., Fabri,A., de Filippis,A.M.B. and de Bruycker-Nogueira,F.
EPI_ISL_18713032, EPI_ISL_18713033, EPI_ISL_18713034, EPI_ISL_18713035	Evandro Chagas Institute, Department of Arbovirology and Hemorrhagic Fevers	Evandro Chagas Institute, Department of Arbovirology and Hemorrhagic Fevers	Amorim,M.T., Hernandez,L.H.A., Naveca,F.G., Essashika Prazeres,I.T., Wanzeller,A.L.M., Silva,E.V.P.D., Casseb,L.M.N., Silva,F.S.D., da Silva,S.P., Nunes,B.T.D. and Cruz,A.C.R.
EPI_ISL_18714550, EPI_ISL_18714551, EPI_ISL_18714552, EPI_ISL_18714553, EPI_ISL_18714554, EPI_ISL_18714555, EPI_ISL_18714556, EPI_ISL_18714557, EPI_ISL_18714558, EPI_ISL_18714559, EPI_ISL_18714560, EPI_ISL_18714561, EPI_ISL_18714562, EPI_ISL_18714563, EPI_ISL_18714564, EPI_ISL_18714565, EPI_ISL_18714566, EPI_ISL_18714567, EPI_ISL_18714568, EPI_ISL_18714569, EPI_ISL_18714570, EPI_ISL_18714571, EPI_ISL_18714572, EPI_ISL_18714573, EPI_ISL_18714574, EPI_ISL_18714575, EPI_ISL_18714576, EPI_ISL_18714577, EPI_ISL_18714578, EPI_ISL_18714579, EPI_ISL_18714580, EPI_ISL_18714581, EPI_ISL_18714582, EPI_ISL_18714583, EPI_ISL_18714584, EPI_ISL_18714585, EPI_ISL_18714586, EPI_ISL_18714587, EPI_ISL_18714588, EPI_ISL_18714589, EPI_ISL_18714590, EPI_ISL_18714591, EPI_ISL_18714592, EPI_ISL_18714593, EPI_ISL_18714594, EPI_ISL_18714595, EPI_ISL_18714596, EPI_ISL_18714597, EPI_ISL_18714598, EPI_ISL_18714599, EPI_ISL_18714600, EPI_ISL_18714601, EPI_ISL_18714602, EPI_ISL_18714603, EPI_ISL_18714604, EPI_ISL_18714605, EPI_ISL_18714606, EPI_ISL_18714607, EPI_ISL_18714608, EPI_ISL_18714609, EPI_ISL_18714610, EPI_ISL_18714611, EPI_ISL_18714612, EPI_ISL_18714613, EPI_ISL_18714614, EPI_ISL_18714615, EPI_ISL_18714616, EPI_ISL_18714617, EPI_ISL_18714618, EPI_ISL_18714619, EPI_ISL_18714620, EPI_ISL_18714621, EPI_ISL_18714622, EPI_ISL_18714623, EPI_ISL_18714624, EPI_ISL_18714625, EPI_ISL_18714626, EPI_ISL_18714627, EPI_ISL_18714628, EPI_ISL_18714629, EPI_ISL_18714630, EPI_ISL_18714631, EPI_ISL_18714632, EPI_ISL_18714633, EPI_ISL_18714634, EPI_ISL_18714635, EPI_ISL_18714636, EPI_ISL_18714637, EPI_ISL_18714638, EPI_ISL_18714639, EPI_ISL_18714640, EPI_ISL_18714641, EPI_ISL_18714642, EPI_ISL_18714643, EPI_ISL_18714644, EPI_ISL_18714645, EPI_ISL_18714646, EPI_ISL_18714647, EPI_ISL_18714648, EPI_ISL_18714649, EPI_ISL_18714650, EPI_ISL_18714651, EPI_ISL_18714652			
see above	Undisclosed Laboratory in Brazil for the Health Emergency Information and Risk Assessment (HIM) Unit of PAHO	Undisclosed Laboratory in Brazil for the Health Emergency Information and Risk Assessment (HIM) Unit of PAHO	Fonseca,V., Giovanetti,M., Xavier,J., Oliveira,C., Lima,M., Barbosa,E., Guimaraes,N., Adelino,T., Fritsch,H., Mendonca,M., Bispo Filippis,A. and Alcantara,L.
EPI_ISL_18737456, EPI_ISL_18737457, EPI_ISL_18737458, EPI_ISL_18737459, EPI_ISL_18737460, EPI_ISL_18737461, EPI_ISL_18737462, EPI_ISL_18737464, EPI_ISL_18737465, EPI_ISL_18737466, EPI_ISL_18737467, EPI_ISL_18737468, EPI_ISL_18737469, EPI_ISL_18737470, EPI_ISL_18737471, EPI_ISL_18737473, EPI_ISL_18737474, EPI_ISL_18737475, EPI_ISL_18737476, EPI_ISL_18737477, EPI_ISL_18737478, EPI_ISL_18737479, EPI_ISL_18737480, EPI_ISL_18737482, EPI_ISL_18737483, EPI_ISL_18737484, EPI_ISL_18737485, EPI_ISL_18737486, EPI_ISL_18737487, EPI_ISL_18737488, EPI_ISL_18737489, EPI_ISL_18737491, EPI_ISL_18737492, EPI_ISL_18737493, EPI_ISL_18737494, EPI_ISL_18737495, EPI_ISL_18737496, EPI_ISL_18737497, EPI_ISL_18737499, EPI_ISL_18737500, EPI_ISL_18737501, EPI_ISL_18737502, EPI_ISL_18737503, EPI_ISL_18737504, EPI_ISL_18737505, EPI_ISL_18737506, EPI_ISL_18737508, EPI_ISL_18737509, EPI_ISL_18737510, EPI_ISL_18737511, EPI_ISL_18737512, EPI_ISL_18737513, EPI_ISL_18737514, EPI_ISL_18737515, EPI_ISL_18737517, EPI_ISL_18737518			
see above	Center for Vectors and Infectious Diseases Research (CEVDI), National Health Institute Doutor Ricardo Jorge, IP (INSA)	Center for Vectors and Infectious Diseases Research (CEVDI), National Health Institute Doutor Ricardo Jorge, IP (INSA)	Ze-Ze,L. and Alves,M.J.
EPI_ISL_18742163, EPI_ISL_18742164, EPI_ISL_18742165	Ministry of Health, Central Virology Laboratory	Ministry of Health, Central Virology Laboratory	Zuckerman,N.S., Schwartz,E., Pandey,P., Erster,O., Halpern,O., Bucris,E., Morad-Eliyahu,H., Wax,M. and Lustig,Y.
EPI_ISL_18951576, EPI_ISL_18951577, EPI_ISL_18951578, EPI_ISL_18951579, EPI_ISL_18951580, EPI_ISL_18951581, EPI_ISL_18951582, EPI_ISL_18951583, EPI_ISL_18951584, EPI_ISL_18951585, EPI_ISL_18951586, EPI_ISL_18951587, EPI_ISL_18951588, EPI_ISL_18951589, EPI_ISL_18951590, EPI_ISL_18951591, EPI_ISL_18951592, EPI_ISL_18951593, EPI_ISL_18951594, EPI_ISL_18951595, EPI_ISL_18951596, EPI_ISL_18951597, EPI_ISL_18951598, EPI_ISL_18951599, EPI_ISL_18951600, EPI_ISL_18951601, EPI_ISL_18951602, EPI_ISL_18951603			
see above	Institute of Tropical Medicine, Nagasaki University	Institute of Tropical Medicine, Nagasaki University	Nabeshima,T., Thi Thanh,N.N., Matsuzaki,A., Xayavong,D., Fraenkel,S., Qiang,X., Nwe,K.M., Palma,J.C.B., Tun,M.M.N., Thuy Duong,N.T., Mai,L.T.Q., Morita,K., Hasebe,F. and Takamatsu,Y.
EPI_ISL_18951604	Institute of Tropical Medicine, Nagasaki University		Nabeshima,T., Thi Thanh,N.N., Matsuzaki,A., Xayavong,D., Fraenkel,S., Qiang,X., Nwe,K.M., Palma,J.C.B., Tun,M.M.N., Thuy Duong,N.T., Mai,L.T.Q., Morita,K., Hasebe,F. and Takamatsu,Y.
EPI_ISL_18951605, EPI_ISL_18951606, EPI_ISL_18951607, EPI_ISL_18951608, EPI_ISL_18951609, EPI_ISL_18951610, EPI_ISL_18951611, EPI_ISL_18951612, EPI_ISL_18951613, EPI_ISL_18951614, EPI_ISL_18951615, EPI_ISL_18951616, EPI_ISL_18951617, EPI_ISL_18951618, EPI_ISL_18951619, EPI_ISL_18951620, EPI_ISL_18951621, EPI_ISL_18951622, EPI_ISL_18951623, EPI_ISL_18951624, EPI_ISL_18951625, EPI_ISL_18951626, EPI_ISL_18951627, EPI_ISL_18951628, EPI_ISL_18951629, EPI_ISL_18951630, EPI_ISL_18951631, EPI_ISL_18951632, EPI_ISL_18951633, EPI_ISL_18951634, EPI_ISL_18951635, EPI_ISL_18951636, EPI_ISL_18951637, EPI_ISL_18951638, EPI_ISL_18951639, EPI_ISL_18951640, EPI_ISL_18951641, EPI_ISL_18951642, EPI_ISL_18951643, EPI_ISL_18951644, EPI_ISL_18951645, EPI_ISL_18951646, EPI_ISL_18951647, EPI_ISL_18951648, EPI_ISL_18951649, EPI_ISL_18951650, EPI_ISL_18951651, EPI_ISL_18951652, EPI_ISL_18951653			
see above	Institute of Tropical Medicine, Nagasaki University	Institute of Tropical Medicine, Nagasaki University	Nabeshima,T., Thi Thanh,N.N., Matsuzaki,A., Xayavong,D., Fraenkel,S., Qiang,X., Nwe,K.M., Palma,J.C.B., Tun,M.M.N., Thuy Duong,N.T., Mai,L.T.Q., Morita,K., Hasebe,F. and Takamatsu,Y.
EPI_ISL_18956187	Evandro Chagas Institute, Department of Arbovirology and Hemorrhagic Fevers	Evandro Chagas Institute, Department of Arbovirology and Hemorrhagic Fevers	Amorim,M.T., Silva,S.P., Hernandez,L.H. and Cruz,A.C.R.
EPI_ISL_19007009, EPI_ISL_19007010, EPI_ISL_19007011, EPI_ISL_19007012, EPI_ISL_19007013, EPI_ISL_19007014, EPI_ISL_19007015, EPI_ISL_19007016, EPI_ISL_19007017, EPI_ISL_19007018, EPI_ISL_19007019, EPI_ISL_19007020, EPI_ISL_19007021, EPI_ISL_19007022, EPI_ISL_19007023, EPI_ISL_19007024, EPI_ISL_19007025, EPI_ISL_19007026, EPI_ISL_19007027, EPI_ISL_19007028, EPI_ISL_19007029, EPI_ISL_19007030, EPI_ISL_19007031, EPI_ISL_19007032, EPI_ISL_19007033, EPI_ISL_19007034, EPI_ISL_19007035, EPI_ISL_19007036, EPI_ISL_19007037, EPI_ISL_19007038, EPI_ISL_19007039, EPI_ISL_19007040, EPI_ISL_19007041, EPI_ISL_19007042, EPI_ISL_19007043, EPI_ISL_19007044, EPI_ISL_19007045, EPI_ISL_19007046, EPI_ISL_19007047, EPI_ISL_19007048, EPI_ISL_19007049, EPI_ISL_19007050, EPI_ISL_19007051, EPI_ISL_19007052, EPI_ISL_19007053, EPI_ISL_19007054, EPI_ISL_19007055, EPI_ISL_19007056, EPI_ISL_19007057, EPI_ISL_19007058, EPI_ISL_19007059, EPI_ISL_19007060, EPI_ISL_19007061, EPI_ISL_19007062, EPI_ISL_19007063, EPI_ISL_19007064, EPI_ISL_19007065, EPI_ISL_19007066, EPI_ISL_19007067, EPI_ISL_19007068, EPI_ISL_19007069, EPI_ISL_19007070, EPI_ISL_19007071, EPI_ISL_19007072, EPI_ISL_19007073, EPI_ISL_19007074, EPI_ISL_19007075, EPI_ISL_19007076, EPI_ISL_19007077, EPI_ISL_19007078, EPI_ISL_19007079, EPI_ISL_19007080, EPI_ISL_19007081, EPI_ISL_19007082, EPI_ISL_19007083, EPI_ISL_19007084, EPI_ISL_19007085, EPI_ISL_19007086, EPI_ISL_19007087, EPI_ISL_19007088, EPI_ISL_19007089, EPI_ISL_19007090, EPI_ISL_19007091, EPI_ISL_19007092, EPI_ISL_19007093, EPI_ISL_19007094, EPI_ISL_19007095, EPI_ISL_19007096, EPI_ISL_19007097, EPI_ISL_19007098, EPI_ISL_19007099, EPI_ISL_19007100, EPI_ISL_19007101, EPI_ISL_19007102, EPI_ISL_19007103, EPI_ISL_19007104, EPI_ISL_19007105, EPI_ISL_19007106, EPI_ISL_19007107, EPI_ISL_19007108, EPI_ISL_19007109, EPI_ISL_19007110, EPI_ISL_19007111, EPI_ISL_19007112, EPI_ISL_19007113, EPI_ISL_19007114, EPI_ISL_19007115, EPI_ISL_19007116, EPI_ISL_19007117, EPI_ISL_19007118, EPI_ISL_19007119, EPI_ISL_19007120, EPI_ISL_19007121, EPI_ISL_19007122, EPI_ISL_19007123, EPI_ISL_19007124, EPI_ISL_19007125, EPI_ISL_19007126, EPI_ISL_19007127, EPI_ISL_19007128, EPI_ISL_19007129, EPI_ISL_19007130, EPI_ISL_19007131, EPI_ISL_19007132, EPI_ISL_19007133, EPI_ISL_19007134, EPI_ISL_19007135, EPI_ISL_19007136, EPI_ISL_19007137, EPI_ISL_19007138, EPI_ISL_19007139, EPI_ISL_19007140, EPI_ISL_19007141, EPI_ISL_19007142, EPI_ISL_19007143, EPI_ISL_19007144, EPI_ISL_19007145, EPI_ISL_19007146, EPI_ISL_19007147, EPI_ISL_19007148, EPI_ISL_19007149, EPI_ISL_19007150, EPI_ISL_19007151, EPI_ISL_19007152, EPI_ISL_19007153, EPI_ISL_19007154, EPI_ISL_19007155, EPI_ISL_19007156, EPI_ISL_19007157, EPI_ISL_19007158, EPI_ISL_19007159, EPI_ISL_19007160, EPI_ISL_19007161, EPI_ISL_19007162, EPI_ISL_19007163, EPI_ISL_19007164, EPI_ISL_19007165, EPI_ISL_19007166, EPI_ISL_19007167, EPI_ISL_19007168, EPI_ISL_19007169, EPI_ISL_19007170, EPI_ISL_19007171, EPI_ISL_19007172, EPI_ISL_19007173, EPI_ISL_19007174, EPI_ISL_19007175, EPI_ISL_19007176, EPI_ISL_19007177, EPI_ISL_19007178, EPI_ISL_19007179, EPI_ISL_19007180, EPI_ISL_19007181, EPI_ISL_19007182, EPI_ISL_19007183, EPI_ISL_19007184, EPI_ISL_19007185, EPI_ISL_19007186, EPI_ISL_19007187, EPI_ISL_19007188, EPI_ISL_19007189, EPI_ISL_19007190, EPI_ISL_19007191, EPI_ISL_19007192, EPI_ISL_19007193, EPI_ISL_19007194, EPI_ISL_19007195, EPI_ISL_19007196, EPI_ISL_19007197, EPI_ISL_19007198, EPI_ISL_19007199, EPI_ISL_19007200, EPI_ISL_19007201, EPI_ISL_19007202, EPI_ISL_19007203, EPI_ISL_19007204, EPI_ISL_19007205, EPI_ISL_19007206, EPI_ISL_19007207, EPI_ISL_19007208, EPI_ISL_19007209, EPI_ISL_19007210, EPI_ISL_19007211, EPI_ISL_19007212, EPI_ISL_19007213, EPI_ISL_19007214, EPI_ISL_19007215, EPI_ISL_19007216, EPI_ISL_19007217, EPI_ISL_19007218, EPI_ISL_19007219, EPI_ISL_19007220, EPI_ISL_19007221, EPI_ISL_19007222, EPI_ISL_19007223, EPI_ISL_19007224, EPI_ISL_19007225, EPI_ISL_19007226, EPI_ISL_19007227, EPI_ISL_19007228, EPI_ISL_19007229, EPI_ISL_19007230, EPI_ISL_19007231, EPI_ISL_19007232, EPI_ISL_19007233, EPI_ISL_19007234, EPI_ISL_19007235, EPI_ISL_19007236, EPI_ISL_19007237, EPI_ISL_19007238, EPI_ISL_19007239, EPI_ISL_19007240, EPI_ISL_19007241, EPI_ISL_19007242, EPI_ISL_19007243, EPI_ISL_19007244, EPI_ISL_19007245, EPI_ISL_19007246, EPI_ISL_19007247, EPI_ISL_19007248, EPI_ISL_19007249, EPI_ISL_19007250, EPI_ISL_19007251, EPI_ISL_19007252, EPI_ISL_19007253, EPI_ISL_19007254, EPI_ISL_19007255, EPI_ISL_19007256, EPI_ISL_19007257, EPI_ISL_19007258, EPI_ISL_19007259, EPI_ISL_19007260, EPI_ISL_19007261, EPI_ISL_19007262, EPI_ISL_19007263, EPI_ISL_19007264, EPI_ISL_19007265, EPI_ISL_19007266, EPI_ISL_19007267, EPI_ISL_19007268, EPI_ISL_19007269, EPI_ISL_19007270, EPI_ISL_19007271, EPI_ISL_19007272, EPI_ISL_19007273, EPI_ISL_19007274, EPI_ISL_19007275, EPI_ISL_19007276, EPI_ISL_19007277, EPI_ISL_19007278, EPI_ISL_19007279, EPI_ISL_19007280, EPI_ISL_19007281, EPI_ISL_19007282, EPI_ISL_19007283, EPI_ISL_19007284, EPI_ISL_19007285, EPI_ISL_19007286, EPI_ISL_19007287, EPI_ISL_19007288, EPI_ISL_19007289, EPI_ISL_19007290, EPI_ISL_19007291, EPI_ISL_19007292, EPI_ISL_19007293, EPI_ISL_19007294, EPI_ISL_19007295, EPI_ISL_19007296, EPI_ISL_19007297, EPI_ISL_19007298, EPI_ISL_19007299, EPI_ISL_19007300, EPI_ISL_19007301, EPI_ISL_19007302, EPI_ISL_19007303, EPI_ISL_19007304, EPI_ISL_19007305, EPI_ISL_19007306, EPI_ISL_19007307, EPI_ISL_19007308, EPI_ISL_19007309, EPI_ISL_19007310, EPI_ISL_19007311, EPI_ISL_19007312, EPI_ISL_19007313, EPI_ISL_19007314, EPI_ISL_19007315, EPI_ISL_19007316, EPI_ISL_19007317, EPI_ISL_19007318, EPI_ISL_19007319, EPI_ISL_19007320, EPI_ISL_19007321, EPI_ISL_19007322, EPI_ISL_19007323, EPI_ISL_19007324, EPI_ISL_19007325, EPI_ISL_19007326, EPI_ISL_19007327, EPI_ISL_19007328, EPI_ISL_19007329, EPI_ISL_19007330, EPI_ISL_19007331, EPI_ISL_19007332, EPI_ISL_19007333, EPI_ISL_19007334, EPI_ISL_19007335, EPI_ISL_19007336, EPI_ISL_19007337, EPI_ISL_19007338, EPI_ISL_19007339, EPI_ISL_19007340, EPI_ISL_19007341, EPI_ISL_19007342, EPI_ISL_19007343, EPI_ISL_19007344, EPI_ISL_19007345, EPI_ISL_19007346, EPI_ISL_19007347, EPI_ISL_19007348, EPI_ISL_19007349, EPI_ISL_19007350, EPI_ISL_19007351, EPI_ISL_19007352, EPI_ISL_19007353, EPI_ISL_19007354, EPI_ISL_19007355, EPI_ISL_19007356, EPI_ISL_19007357, EPI_ISL_19007358, EPI_ISL_19007359, EPI_ISL_19007360, EPI_ISL_19007361, EPI_ISL_19007362, EPI_ISL_19007363, EPI_ISL_19007364, EPI_ISL_19007365, EPI_ISL_19007366, EPI_ISL_19007367, EPI_ISL_19007368, EPI_ISL_19007369, EPI_ISL_19007370, EPI_ISL_19007371, EPI_ISL_19007372, EPI_ISL_19007373, EPI_ISL_19007374, EPI_ISL_19007375, EPI_ISL_19007376, EPI_ISL_19007377, EPI_ISL_19007378, EPI_ISL_19007379, EPI_ISL_19007380, EPI_ISL_19007381, EPI_ISL_19007382, EPI_ISL_19007383, EPI_ISL_19007384, EPI_ISL_19007385, EPI_ISL_19007386, EPI_ISL_19007387, EPI_ISL_19007388, EPI_ISL_19007389, EPI_ISL_19007390, EPI_ISL_19007391, EPI_ISL_19007392, EPI_ISL_19007393, EPI_ISL_19007394, EPI_ISL_19007395, EPI_ISL_19007396, EPI_ISL_19007397, EPI_ISL_19007398, EPI_ISL_19007399, EPI_ISL_19007400, EPI_ISL_19007401, EPI_ISL_19007402, EPI_ISL_19007403, EPI_ISL_19007404, EPI_ISL_19007405, EPI_ISL_19007406, EPI_ISL_19007407, EPI_ISL_19007408, EPI_ISL_19007409, EPI_ISL_19007410, EPI_ISL_19007411, EPI_ISL_19007412, EPI_ISL_19007413, EPI_ISL_19007414, EPI_ISL_19007415, EPI_ISL_19007416, EPI_ISL_19007417, EPI_ISL_19007418, EPI_ISL_19007419, EPI_ISL_19007420, EPI_ISL_19007421, EPI_ISL_19007422, EPI_ISL_19007423, EPI_ISL_19007424, EPI_ISL_19007425, EPI_ISL_19007426, EPI_ISL_19007427, EPI_ISL_19007428, EPI_ISL_19007429, EPI_ISL_19007430, EPI_ISL_19007431, EPI_ISL_19007432, EPI_ISL_19007433, EPI_ISL_19007434, EPI_ISL_19007435, EPI_ISL_19007436, EPI_ISL_19007437, EPI_ISL_19007438, EPI_ISL_19007439, EPI_ISL_19007440, EPI_ISL_19007441, EPI_ISL_19007442, EPI_ISL_19007443, EPI_ISL_19007444, EPI_ISL_19007445, EPI_ISL_19007446, EPI_ISL_19007447, EPI_ISL_19007448, EPI_ISL_19007449, EPI_ISL_19007450, EPI_ISL_19007451, EPI_ISL_19007452, EPI_ISL_19007453, EPI_ISL_19007454, EPI_ISL_19007455, EPI_ISL_19007456, EPI_ISL_19007457, EPI_ISL_19007458, EPI_ISL_19007459, EPI_ISL_19007460, EPI_ISL_19007461, EPI_ISL_19007462, EPI_ISL_19007463, EPI_ISL_19007464, EPI_ISL_19007465, EPI_ISL_19007466, EPI_ISL_19007467, EPI_ISL_19007468, EPI_ISL_19007469, EPI_ISL_19007470, EPI_ISL_19007471, EPI_ISL_19007472, EPI_ISL_19007473, EPI_ISL_19007474, EPI_ISL_19007475, EPI_ISL_19007476, EPI_ISL_19007477, EPI_ISL_19007478, EPI_ISL_19007479, EPI_ISL_19007480, EPI_ISL_19007481, EPI_ISL_19007482, EPI_ISL_19007483, EPI_ISL_19007484, EPI_ISL_19007485, EPI_ISL_19007486, EPI_ISL_19007487, EPI_ISL_19007488, EPI_ISL_19007489, EPI_ISL_19007490, EPI_ISL_19007491, EPI_ISL_19007492, EPI_ISL_19007493, EPI_ISL_19007494, EPI_ISL_19007495, EPI_ISL_19007496, EPI_ISL_19007497, EPI_ISL_19007498, EPI_ISL_19007499, EPI_ISL_19007500, EPI_ISL_19007501, EPI_ISL_19007502, EPI_ISL_19007503, EPI_ISL_19007504, EPI_ISL_19007505, EPI_ISL_19007506, EPI_ISL_19007507, EPI_ISL_19007508, EPI_ISL_19007509, EPI_ISL_19007510, EPI_ISL_19007511, EPI_ISL_19007512, EPI_ISL_19007513, EPI_ISL_19007514, EPI_ISL_19007515, EPI_ISL_19007516, EPI_ISL_19007517, EPI_ISL_19007518, EPI_ISL_19007519, EPI_ISL_19007520, EPI_ISL_19007521, EPI_ISL_19007522, EPI_ISL_19007523, EPI_ISL_19007524, EPI_ISL_19007525, EPI_ISL_19007526, EPI_ISL_19007527, EPI_ISL_19007528, EPI_ISL_19007529, EPI_ISL_19007530, EPI_ISL_19007531, EPI_ISL_19007532, EPI_ISL_19007533, EPI_ISL_19007534, EPI_ISL_19007535, EPI_ISL_19007536, EPI_ISL_19007537, EPI_ISL_19007538, EPI_ISL_19007539, EPI_ISL_19007540, EPI_ISL_19007541, EPI_ISL_19007542, EPI_ISL_19007543, EPI_ISL_19007544, EPI_ISL_19007545, EPI_ISL_19007546, EPI_ISL_19007547, EPI_ISL_19007548, EPI_ISL_19007549, EPI_ISL_19007550, EPI_ISL_19007551, EPI_ISL_19007552, EPI_ISL_19007553, EPI_ISL_19007554, EPI_ISL_19007555, EPI_ISL_19007556, EPI_ISL_19007557, EPI_ISL_19007558, EPI_ISL_19007559, EPI_ISL_19007560, EPI_ISL_19007561, EPI_ISL_19007562, EPI_ISL_19007563, EPI_ISL_19007564, EPI_ISL_19007565, EPI_ISL_19007566, EPI_ISL_19007567, EPI_ISL_19007568, EPI_ISL_19007569, EPI_ISL_19007570, EPI_ISL_19007571, EPI_ISL_19007572, EPI_ISL_19007573, EPI_ISL_19007574, EPI_ISL_19007575, EPI_ISL_19007576, EPI_ISL_19007577, EPI_ISL_19007578, EPI_ISL_19007579, EPI_ISL_19007580, EPI_ISL_19007581, EPI_ISL_19007582, EPI_ISL_19007583, EPI_ISL_19007584, EPI_ISL_19007585, EPI_ISL_19007586, EPI_ISL_19007587, EPI_ISL_19007588, EPI_ISL_19007589, EPI_ISL_19007590, EPI_ISL_19007591, EPI_ISL_19007592, EPI_ISL_19007593, EPI_ISL_19007594, EPI_ISL_19007595, EPI_ISL_19007596, EPI_ISL_19007597, EPI_ISL_19007598, EPI_ISL_19007599, EPI_ISL_19007600, EPI_ISL_19007601, EPI_ISL_19007602, EPI_ISL_19007603, EPI_ISL_19007604, EPI_ISL_19007605, EPI_ISL_19007606, EPI_ISL_19007607, EPI_ISL_19007608, EPI_ISL_19007609, EPI_ISL_19007610, EPI_ISL_19007611, EPI_ISL_19007612, EPI_ISL_19007613, EPI_ISL_19007614, EPI_ISL_19007615, EPI_ISL_19007616, EPI_ISL_19007617, EPI_ISL_19007618, EPI_ISL_19007619, EPI_ISL_19007620, EPI_ISL_19007621, EPI_ISL_19007622, EPI_ISL_19007623, EPI_ISL_19007624, EPI_ISL_19007625, EPI_ISL_19007626, EPI_ISL_19007627, EPI_ISL_19007628, EPI_ISL_19007629, EPI_ISL_19007630, EPI_ISL_19007631, EPI_ISL_19007632, EPI_ISL_19007633, EPI_ISL_19007634, EPI_ISL_19007635, EPI_ISL_19007636, EPI_ISL_19007637, EPI_ISL_19007638, EPI_ISL_19007639, EPI_ISL_19007640, EPI_ISL_19007641, EPI_ISL_19007642, EPI_ISL_19007643, EPI_ISL_19007644, EPI_ISL_19007645, EPI_ISL_19007646, EPI_ISL_19007647, EPI_ISL_19007648, EPI_ISL_19007649, EPI_ISL_19007650, EPI_ISL_19007651, EPI_ISL_19007652, EPI_ISL_19007653, EPI_ISL_19007654, EPI_ISL_19007655, EPI_ISL_19007656, EPI_ISL_19007657, EPI_ISL_19007658, EPI_ISL_19007659, EPI_ISL_19007660, EPI_ISL_19007661, EPI_ISL_19007662, EPI_ISL_19007663, EPI_ISL_19007664, EPI_ISL_19007665, EPI_ISL_19007666, EPI_ISL_19007667, EPI_ISL_19007668, EPI_ISL_19007669, EPI_ISL_19007670, EPI_ISL_19007671, EPI_ISL_19007672, EPI_ISL_19007673, EPI_ISL_19007674, EPI_ISL_19007675, EPI_ISL_19007676, EPI_ISL_19007677, EPI_ISL_19007678, EPI_ISL_19007679, EPI_ISL_19007680, EPI_ISL_19007681, EPI_ISL_19007682, EPI_ISL_19007683, EPI_ISL_19007684, EPI_ISL_19007685, EPI_ISL_19007686, EPI_ISL_19007687, EPI_ISL_19007688, EPI_ISL_19007689, EPI_ISL_19007690, EPI_ISL_19007691, EPI_ISL_19007692, EPI_ISL_19007693, EPI_ISL_19007694, EPI_ISL_19007695, EPI_ISL_19007696, EPI_ISL_19007697, EPI_ISL_19007698, EPI_ISL_19007699, EPI_ISL_19007700, EPI_ISL_19007701, EPI_ISL_19007702, EPI_ISL_19007703, EPI_ISL_19007704, EPI_ISL_19007705, EPI_ISL_19007706, EPI_ISL_19007707, EPI_ISL_19007708, EPI_ISL_19007709, EPI_ISL_19007710, EPI_ISL_19007711, EPI_ISL_19007712, EPI_ISL_19007713, EPI_ISL_19007714, EPI_ISL_19007715, EPI_ISL_19007716, EPI_ISL_19007717, EPI_ISL_19007718, EPI_ISL_19007719, EPI_ISL_19007720, EPI_ISL_19007721, EPI_ISL_19007722, EPI_ISL_19007723, EPI_ISL_19007724, EPI_ISL_19007725, EPI_ISL_19007726, EPI_ISL_19007727, EPI_ISL_19007728, EPI_ISL_19007729, EPI_ISL_19007730, EPI_ISL_19007731, EPI_ISL_19007732, EPI_ISL_19007733, EPI_ISL_19007734, EPI_ISL_19007735, EPI_ISL_19007736, EPI_ISL_19007737, EPI_ISL_19007738, EPI_ISL_19007739, EPI_ISL_19007740, EPI_ISL_19007741, EPI_ISL_19007742, EPI_ISL_19007743, EPI_ISL_19007744, EPI_ISL_19007745, EPI_ISL_19007746, EPI_ISL_19007747, EPI_ISL_19007748, EPI_ISL_19007749, EPI_ISL_19007750, EPI_ISL_19007751, EPI_ISL_19007752, EPI_ISL_19007753, EPI_ISL_19007754, EPI_ISL_19007755, EPI_ISL_19007756, EPI_ISL_19007757, EPI_ISL_19007758, EPI_ISL_19007759, EPI_ISL_19007760, EPI_ISL_19007761, EPI_ISL_19007762, EPI_ISL_19007763, EPI_ISL_19007764, EPI_ISL_19007765, EPI_ISL_19007766, EPI_ISL_19007767, EPI_ISL_19007768, EPI_ISL_19007769, EPI_ISL_19007770, EPI_ISL_19007771, EPI_ISL_19007772, EPI_ISL_19007773, EPI_ISL_19007774, EPI_ISL_19007775, EPI_ISL_19007776, EPI_ISL_19007777, EPI_ISL_19007778, EPI_ISL_19007779, EPI_ISL_19007780, EPI_ISL_19007781, EPI_ISL_19007782, EPI_ISL_19007783, EPI_ISL_19007784, EPI_ISL_19007785, EPI_ISL_19007786, EPI_ISL_19007787, EPI_ISL_19007788, EPI_ISL_19007789, EPI_ISL_19007790, EPI_ISL_19007791, EPI_ISL_19007792, EPI_ISL_19007793, EPI_ISL_19007794, EPI_ISL_19007795, EPI_ISL_19007796, EPI_ISL_19007797, EPI_ISL_19007798, EPI_ISL_19007799, EPI_ISL_19007800, EPI_ISL_19007801, EPI_ISL_19007802, EPI_ISL_19007803, EPI_ISL_19007804, EPI_ISL_19007805, EPI_ISL_19007806, EPI_ISL_19007807, EPI_ISL_19007808, EPI_ISL_19007809, EPI_ISL_19007810, EPI_ISL_19007811, EPI_ISL_19007812, EPI_ISL_19007813, EPI_ISL_19007814, EPI_ISL_19007815, EPI_ISL_19007816, EPI_ISL_19007817, EPI_ISL_19007818, EPI_ISL_19007819, EPI_ISL_19007820, EPI_ISL_19007821, EPI_ISL_19007822, EPI_ISL_19007823, EPI_ISL_19007824, EPI_ISL_19007825, EPI_ISL_19007826, EPI_ISL_19007827, EPI_ISL_19007828, EPI_ISL_19007829, EPI_ISL_19007830, EPI_ISL_19007831, EPI_ISL_19007832, EPI_ISL_19007833, EPI_ISL_19007834, EPI_ISL_19007835, EPI_ISL_19007836, EPI_ISL_19007837, EPI_ISL_19007838, EPI_ISL_19007839, EPI_ISL_19007840, EPI_ISL_19007841, EPI_ISL_19007842, EPI_ISL_19007843, EPI_ISL_19007844, EPI_ISL_19007845, EPI_ISL_19007846, EPI_ISL_19007847, EPI_ISL_19007848, EPI_ISL_19007849, EPI_ISL_19007850, EPI_ISL_19007851, EPI_ISL_19007852, EPI_ISL_19007853, EPI_ISL_19007854, EPI_ISL_19007855, EPI_ISL_19007856, EPI_ISL_19007857, EPI_ISL_19007858, EPI_ISL_19007859, EPI_ISL_19007860, EPI_ISL_19007861, EPI_ISL_19007862, EPI_ISL_19007863, EPI_ISL_19007864, EPI_ISL_19007865, EPI_ISL_19007866, EPI_ISL_19007867, EPI_ISL_19007868, EPI_ISL_19007869, EPI_ISL_19007870, EPI_ISL_19007871, EPI_ISL_19007872, EPI_ISL_19007873, EPI_ISL_19007874, EPI_ISL_19007875, EPI_ISL_19007876, EPI_ISL_19007877, EPI_ISL_19007878, EPI_ISL_19007879, EPI_ISL_19007880, EPI_ISL_19007881, EPI_ISL_19007882, EPI_ISL_19007883, EPI_ISL_19007884, EPI_ISL_19007885, EPI_ISL_19007886, EPI_ISL_19007887, EPI_ISL_19007888, EPI_ISL_19007889, EPI_ISL_19007890, EPI_ISL_19007891, EPI_ISL_19007892, EPI_ISL_19007893, EPI_ISL_19007894, EPI_ISL_19007895, EPI_ISL_19007896, EPI_ISL_19007897, EPI_ISL_19007898, EPI_ISL_19007899, EPI_ISL_19007900, EPI_ISL_19007901, EPI_ISL_19007902, EPI_ISL_19007903, EPI_ISL_19007904, EPI_ISL_19007905, EPI_ISL_19007906, EPI_ISL_19007907, EPI_ISL_19007908, EPI_ISL_19007909, EPI_ISL_19007910, EPI_ISL_19007911, EPI_ISL_19007912, EPI_ISL_19007913, EPI_ISL_19007914, EPI_ISL_19007915, EPI_ISL_19007916			
see above	Laboratoire de virologie, Centre National de Référence arbovirus Associe (CNR), Centre Hospitalier Universitaire de la Reunion (CHU)	Laboratoire de virologie, Centre National de Référence arbovirus Associe (CNR), Centre Hospitalier Universitaire de la Reunion (CHU)	Frumence,E., Traversier,N. and Jaffar Bandjee,M.-C.
EPI_ISL_19026416, EPI_ISL_19026417, EPI_ISL_19026418, EPI_ISL_19026419, EPI_ISL_19026420, EPI_ISL_19026421, EPI_ISL_19026422, EPI_ISL_19026423, EPI_ISL_19026424, EPI_ISL_19026425, EPI_ISL_19026426, EPI_ISL_19026427, EPI_ISL_19026428, EPI_ISL_19026429			
see above	Nagasaki University, Department of Virology	Nagasaki University, Department of Virology	Ngwe Tun,M.M., Kyaw,A.K., Nabeshima,T., Dumre,S.P., Soe,A.M., Nwe,K.M., Myaing,S.S., Lwin,E.P., Win,Y.T., Inoue,S., Takamatsu,Y., Urano,T., Thu,H.M., Thant,K.Z., Htun,Z.T. and Morita,K.
EPI_ISL_19091309, EPI_ISL_19091310, EPI_ISL_19091311, EPI_ISL_19091312, EPI_ISL_19091313, EPI_ISL_19091314, EPI_ISL_19091315, EPI_ISL_19091316, EPI_ISL_19091317, EPI_ISL_19091318, EPI_ISL_19091319, EPI_ISL_19091320, EPI_ISL_19091321, EPI_ISL_19091322			
see above	Virology, US Army Medical Directorate of the Armed Forces Research Institute of Medical Sciences (USAMD-AFRIMS)	Virology, US Army Medical Directorate of the Armed Forces Research Institute of Medical Sciences (USAMD-AFRIMS)	Gyeltshen,S., Dorji,K., Zangmo,S., Adhikari,L.M., Dorji,T., Wangchuk,S., Rodpradit,P., Phonpakobsin,T., Poolpanichupatam,Y., Chinnawirotpisan,P., Manasatienkij,W., Cotrone,T.S., Fernandez,S. and Klungthong,C.
EPI_ISL_8636670, EPI_ISL_8636671, EPI_ISL_8636672, EPI_ISL_8636673, EPI_ISL_8636674, EPI_ISL_8636675, EPI_ISL_8636676	Guangxi Zhuang Autonomous Region Center for Disease Prevention and Control	Guangxi Zhuang Autonomous Region Center for Disease Prevention and Control	Wang,J.

EPI_ISL_8766816, EPI_ISL_8766817, EPI_ISL_8766818, EPI_ISL_8766819, EPI_ISL_8766820, EPI_ISL_8766821, EPI_ISL_8766822, EPI_ISL_8766823, EPI_ISL_8766824, EPI_ISL_8766825, EPI_ISL_8766826, EPI_ISL_8766827, EPI_ISL_8766828, EPI_ISL_8766876, EPI_ISL_8767061, EPI_ISL_8767062, EPI_ISL_8767063, EPI_ISL_8767064, EPI_ISL_8767626, EPI_ISL_8768614, EPI_ISL_8768615, EPI_ISL_8768616, EPI_ISL_8768617, EPI_ISL_8768889, EPI_ISL_8768890, EPI_ISL_8768891, EPI_ISL_8768892, EPI_ISL_8768893, EPI_ISL_8768894, EPI_ISL_8768895, EPI_ISL_8768896			
see above	Shanghai Municipal Center for Disease Control and Prevention	Shanghai Municipal Center for Disease Control and Prevention	Wang,W.
EPI_ISL_8824344, EPI_ISL_8824350	Emory University School of Medicine	Emory University School of Medicine	Key,A., Waggoner,J., Ali,I.M., Piantadosi,A.L. and Collins,M.
EPI_ISL_9004460, EPI_ISL_9004461, EPI_ISL_9004462, EPI_ISL_9004463, EPI_ISL_9004464	UNIVERSITY OF HYDERABAD	UNIVERSITY OF HYDERABAD	Maisnam,D. and Musturi,V.
EPI_ISL_9004465, EPI_ISL_9004466, EPI_ISL_9004467, EPI_ISL_9004468, EPI_ISL_9004469	Guangzhou Center for Disease Control and Prevention	Guangzhou Center for Disease Control and Prevention	Su,W., Cao,Y. and Lu,W.
EPI_ISL_9004470	Guangzhou Center for Disease Control and Prevention	Guangzhou Center for Disease Control and Prevention	Su,W., Jiang,L. and Lu,W.
EPI_ISL_9103208, EPI_ISL_9103209, EPI_ISL_9103210, EPI_ISL_9103211	Chinese Academy of Sciences	Chinese Academy of Sciences	lihong,H.
EPI_ISL_9152287, EPI_ISL_9152288, EPI_ISL_9152289, EPI_ISL_9152290, EPI_ISL_9152291, EPI_ISL_9152292, EPI_ISL_9437081, EPI_ISL_9437082	Secretaria de Salud	Secretaria de Salud	Wong-Arambula,C.E., Rodriguez-Maldonado,A.P., Fuentes-Cuevas,C.H., Duran-Ayala,D., Torres-Rodriguez,Mdl.L., Rivero-Arredondo,V., Serrano-Gutierrez,S., Aparicio-Antonio,M., Rosales-Jimenez,C., del Mazo-Lopez,J.C., Hernandez-Rivas,L., Lopez-Martinez,I., Ramirez-Gonzalez,J.E. and Vazquez-Pichardo,M.
EPI_ISL_9636328, EPI_ISL_9636329, EPI_ISL_9636330, EPI_ISL_9636331, EPI_ISL_9636332	University Of Hyderabad	UNIVERSITY OF HYDERABAD	Maisnam,D. and Musturi,V.
EPI_ISL_9711778, EPI_ISL_9711914, EPI_ISL_9711959, EPI_ISL_9712018, EPI_ISL_9712019, EPI_ISL_9712020, EPI_ISL_9712021, EPI_ISL_9712022, EPI_ISL_9712023, EPI_ISL_9712024, EPI_ISL_9713936, EPI_ISL_9713937, EPI_ISL_9713980, EPI_ISL_9713981, EPI_ISL_9713982, EPI_ISL_9713983, EPI_ISL_9713984, EPI_ISL_9713985, EPI_ISL_9713986, EPI_ISL_9713987, EPI_ISL_9713988, EPI_ISL_9713989, EPI_ISL_9713990, EPI_ISL_9713991			
see above	National Institute of Virology	National Institute of Virology	Yadav,P.D.
EPI_ISL_9713992	Adolfo Lutiz Institute	Adolfo Lutiz Institute	Cunha,M.S., de Moura Coletti,T., Guerra,J.M., Ponce,C.C., Fernandes,N.C.C.A., Resio,R.A., Claro,I.M., Salles,F., Lima Neto,D.F. and Sabino,E.
EPI_ISL_9768996, EPI_ISL_9769066	Fundacion Universitaria Autonoma de las Americas	Fundacion Universitaria Autonoma de las Americas	Rojas-Gallardo,D.M., Restrepo-Chica,J., Key,A., Acosta,A., Castrillon-Spitia,J.D., Tabares-Villa,F.A., Trujillo,A.M., JJimenez-Posada,E.V., Martinez-Gutierrez,M., Ruiz-Saenz,J., Waggoner,J., Collins,M.H., Piantadosi,A. and Cardona-Ospina,J.A.
EPI_ISL_9845835, EPI_ISL_9845836, EPI_ISL_9882074, EPI_ISL_9882077, EPI_ISL_9935327, EPI_ISL_9935714, EPI_ISL_9936221, EPI_ISL_9988597, EPI_ISL_9988598	National Institute of Virology	National Institute of Virology	Yadav,P.D.
