# Supplementary figures and images for: A new lineage nomenclature to aid genomic surveillance of dengue virus
Source: PLoS Biol. 2024 Sep 16;22(9):e3002834. doi: 10.1371/journal.pbio.3002834 (PMC11426435; doi:10.1371/journal.pbio.3002834)

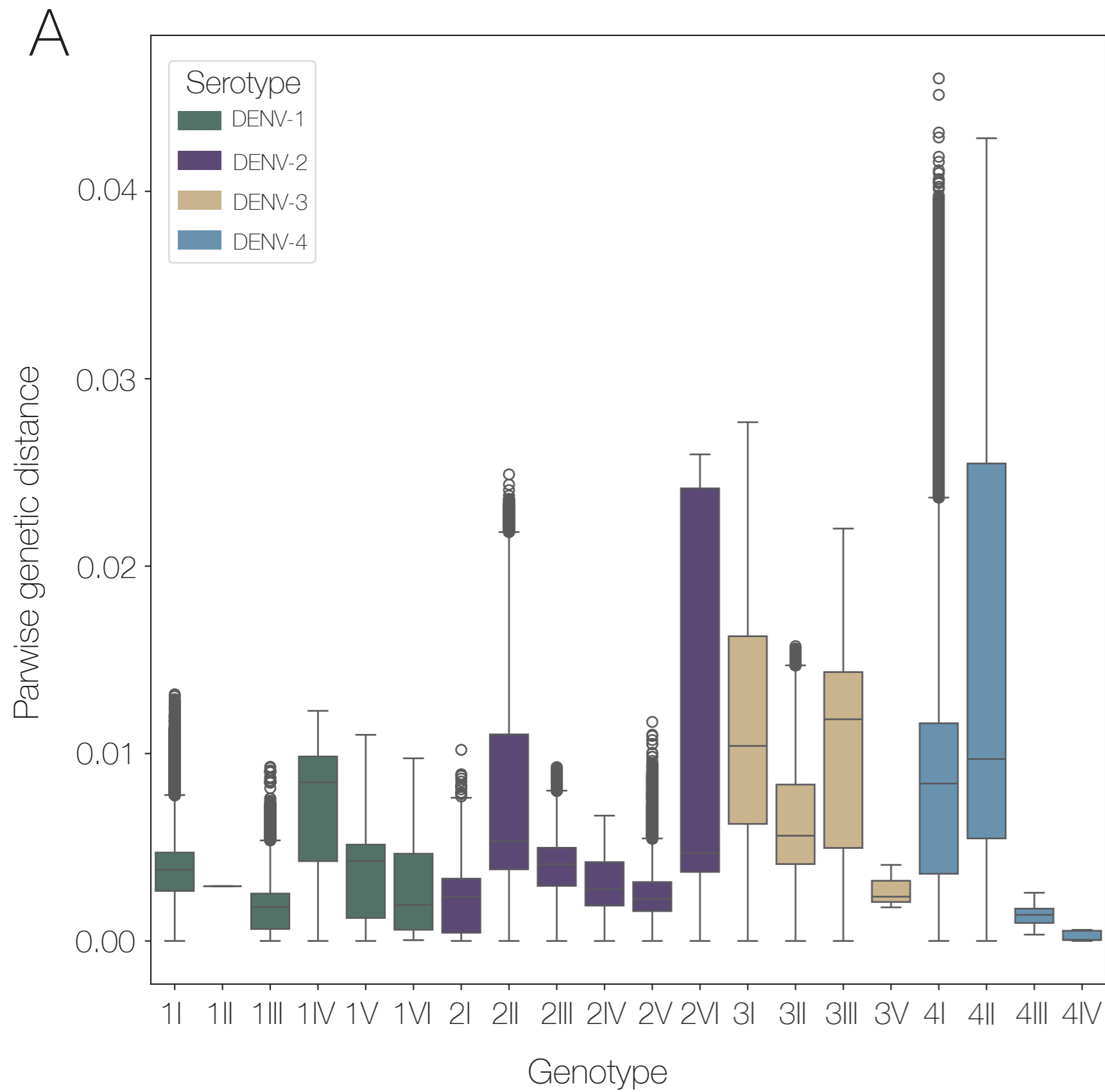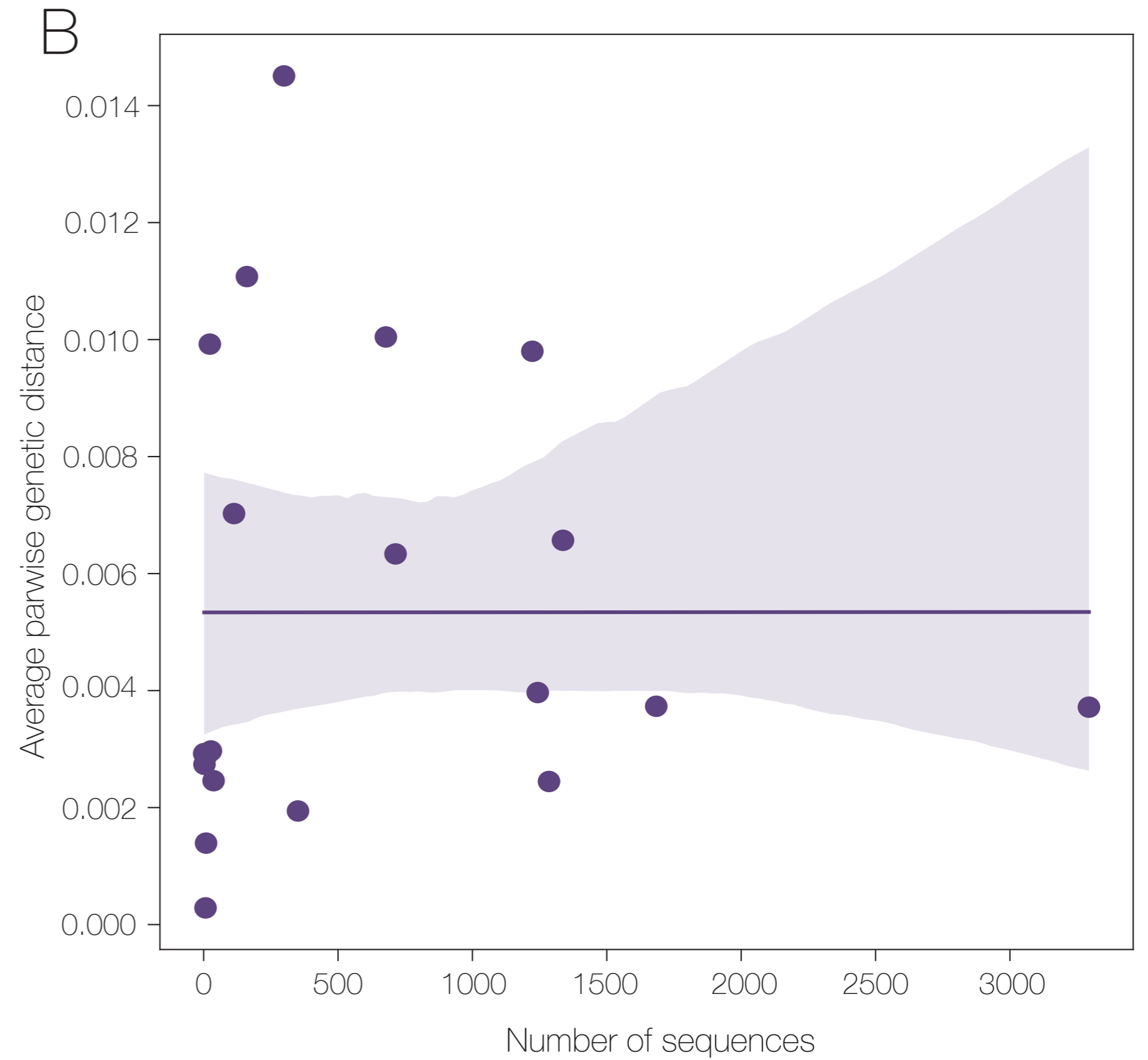

Supplement: S1 Fig — (A) Distribution of pairwise genetic distance within each genotype, colored by serotype. (B) Regression of the number of sequences in each genotype compared to the average pairwise genetic distance. (PDF) [file pbio.3002834.s005.pdf]

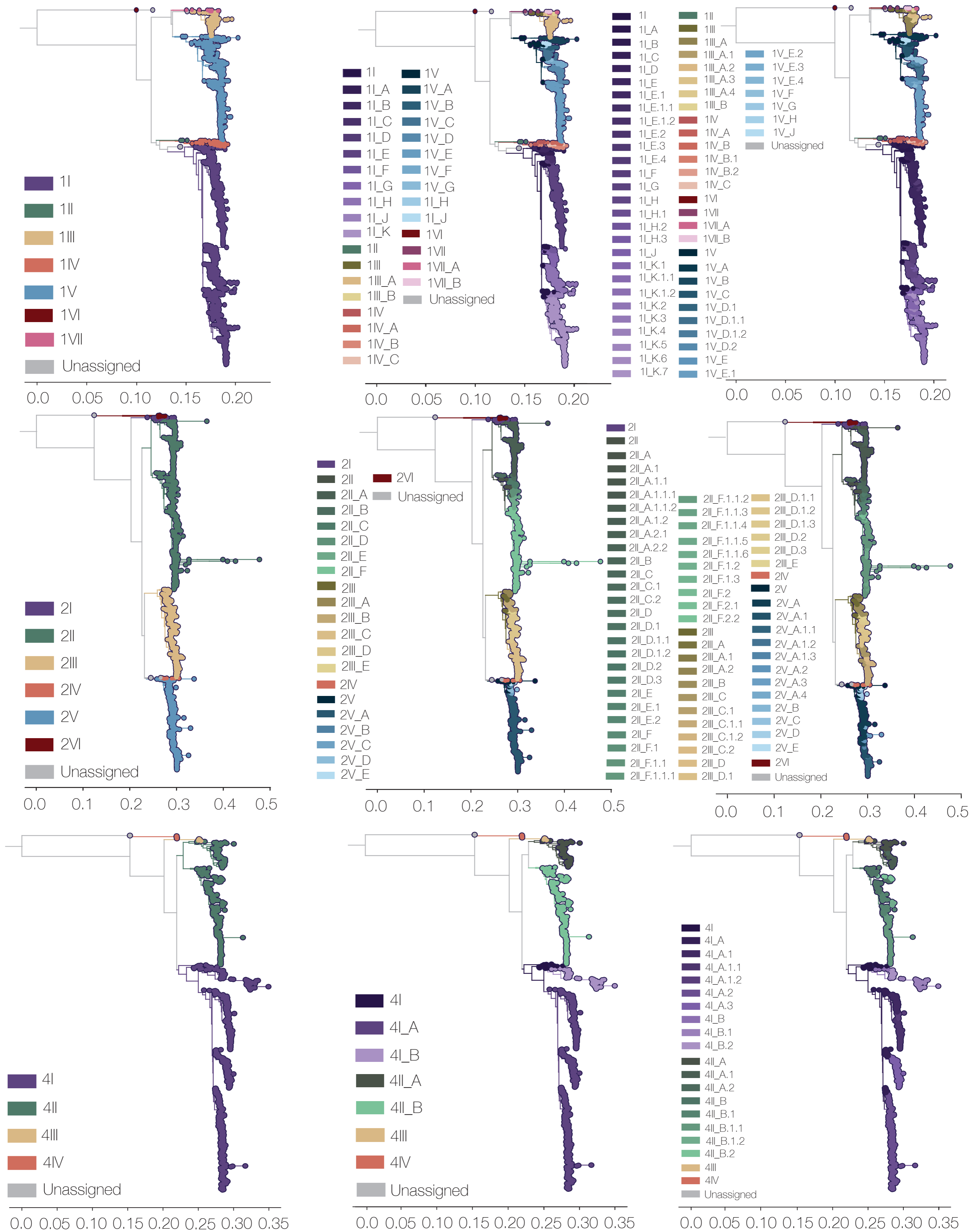

Supplement: S2 Fig — Each row is a serotype and each column, respectively, is new genotype, major lineage and minor lineage. Note that there are many more minor lineages for serotypes 1 and 2, as they have much larger data sets currently compared to serotype 4. Serotype 3 shown in Fig 2. (PDF) [file pbio.3002834.s006.pdf]

A

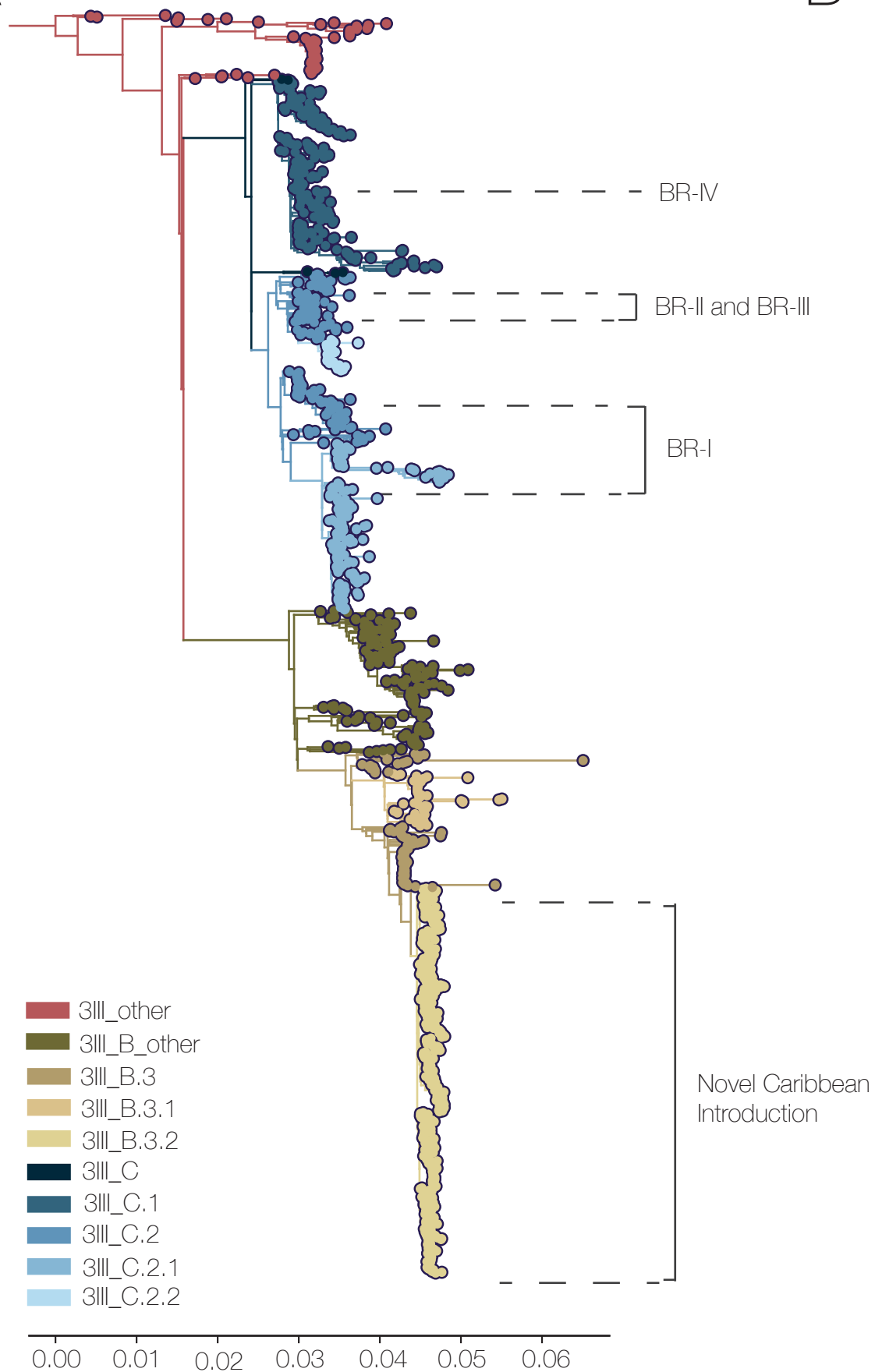

B

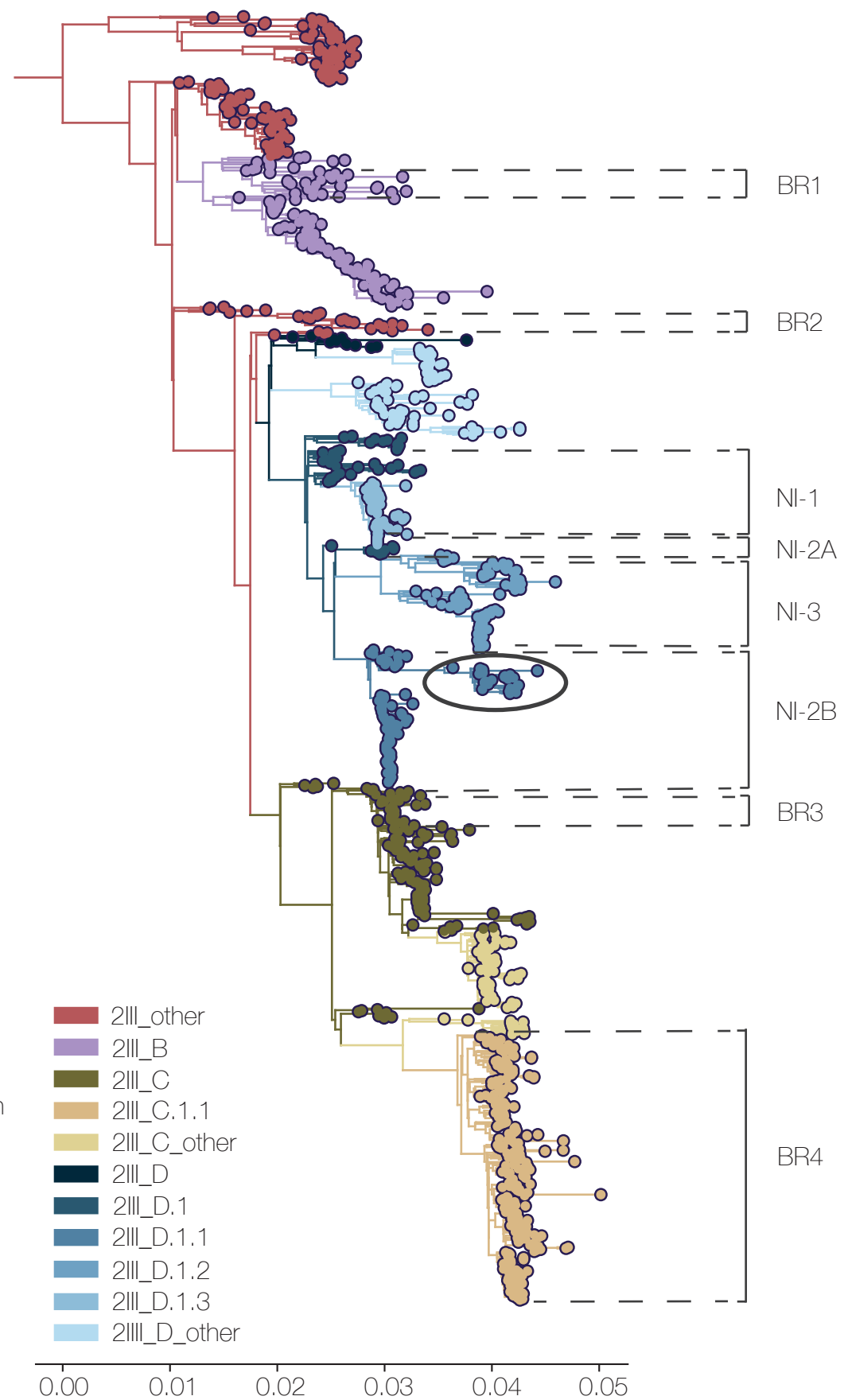

Supplement: S4 Fig — (A) Maximum likelihood tree of DENV-3 genotype III, colored by new lineage designation, with lineages BRI-IV and novel Caribbean introduction indicated. (B) Maximum likelihood tree of DENV-2 genotype III, colored by new lineage designation, with lineages BR1-4 and NI-1 to NI-3 indicated. The circled clade indicates recent circulation of NI-2B/2III_D.1.1, which is suggested to have a transmission advantage [9], in Cuba and Puerto Rico. (PDF) [file pbio.3002834.s008.pdf]

A

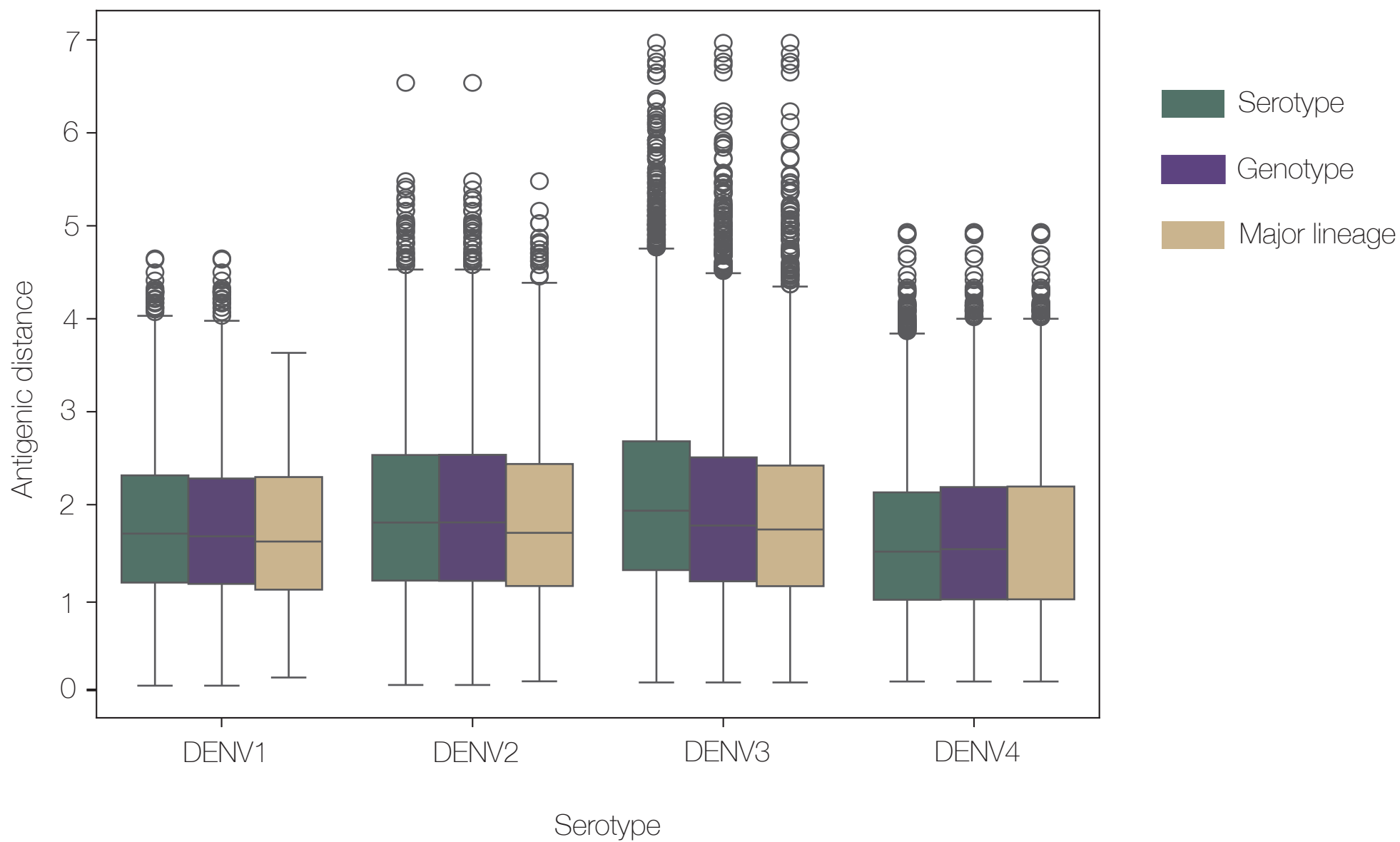

B

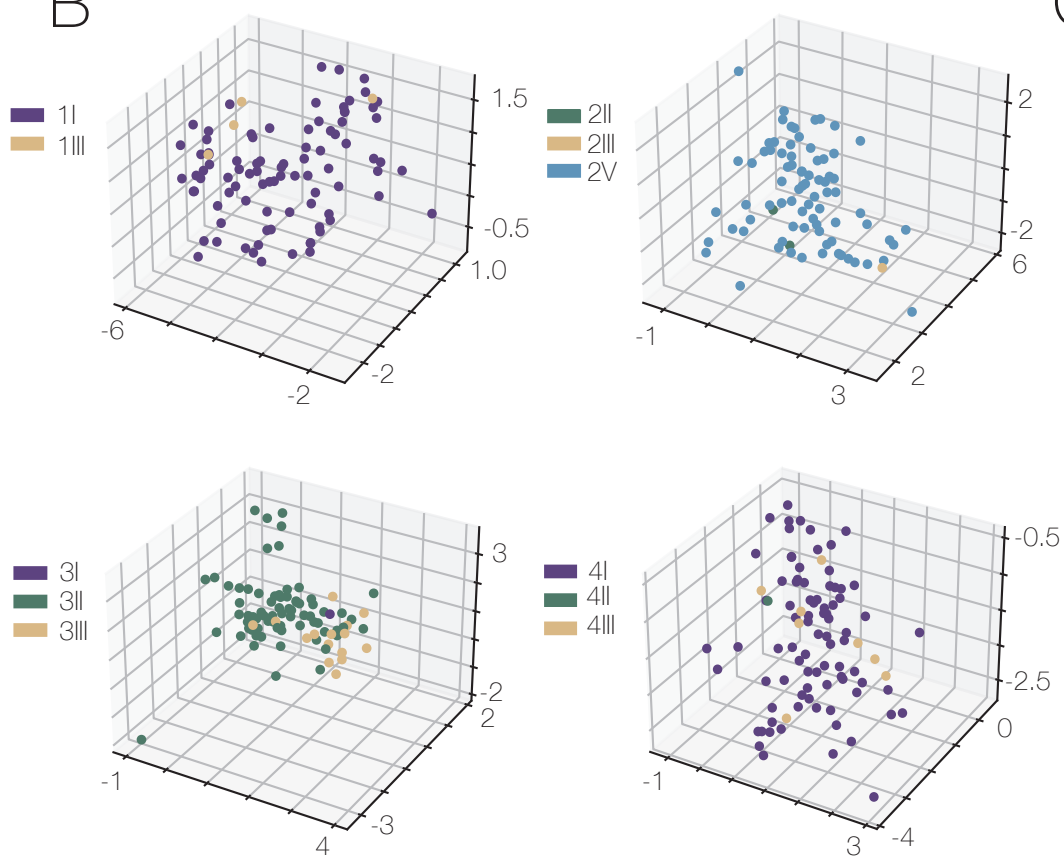

C

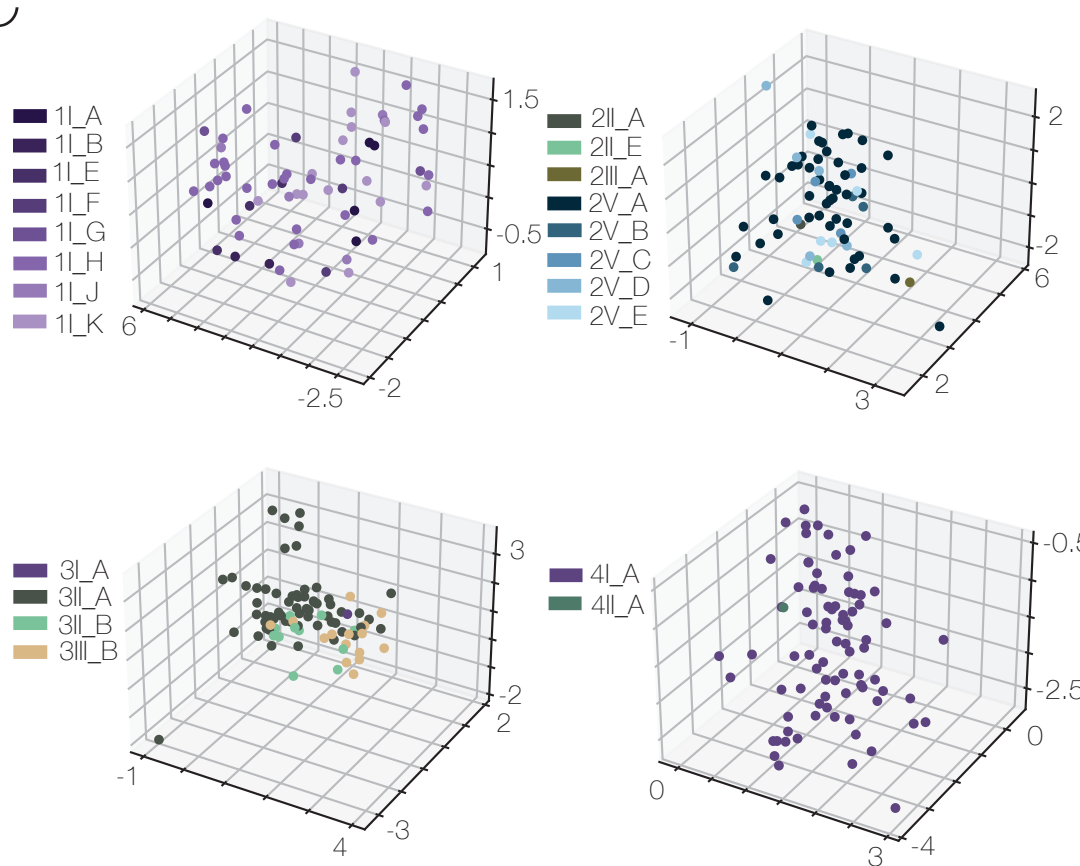

Supplement: S5 Fig — (A) Distribution of antigenic distance in each level of classification, with serotype in green, genotype in purple and major lineage in yellow. Minor lineage is excluded due to a lack of antigenic data across minor lineages. (B) 3D map of sequences in antigenic space by serotype, colored by genotype. (C) 3D map of sequences in antigenic space by serotype, colored by major lineage (PDF) [file pbio.3002834.s009.pdf]

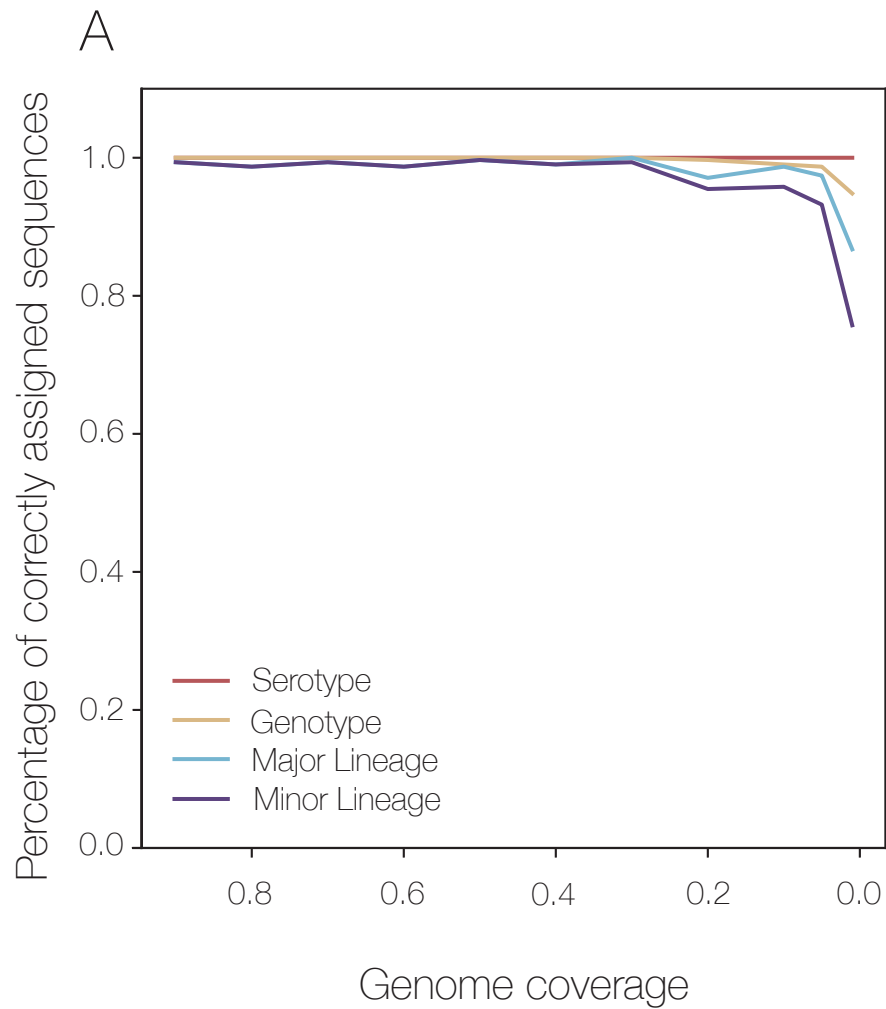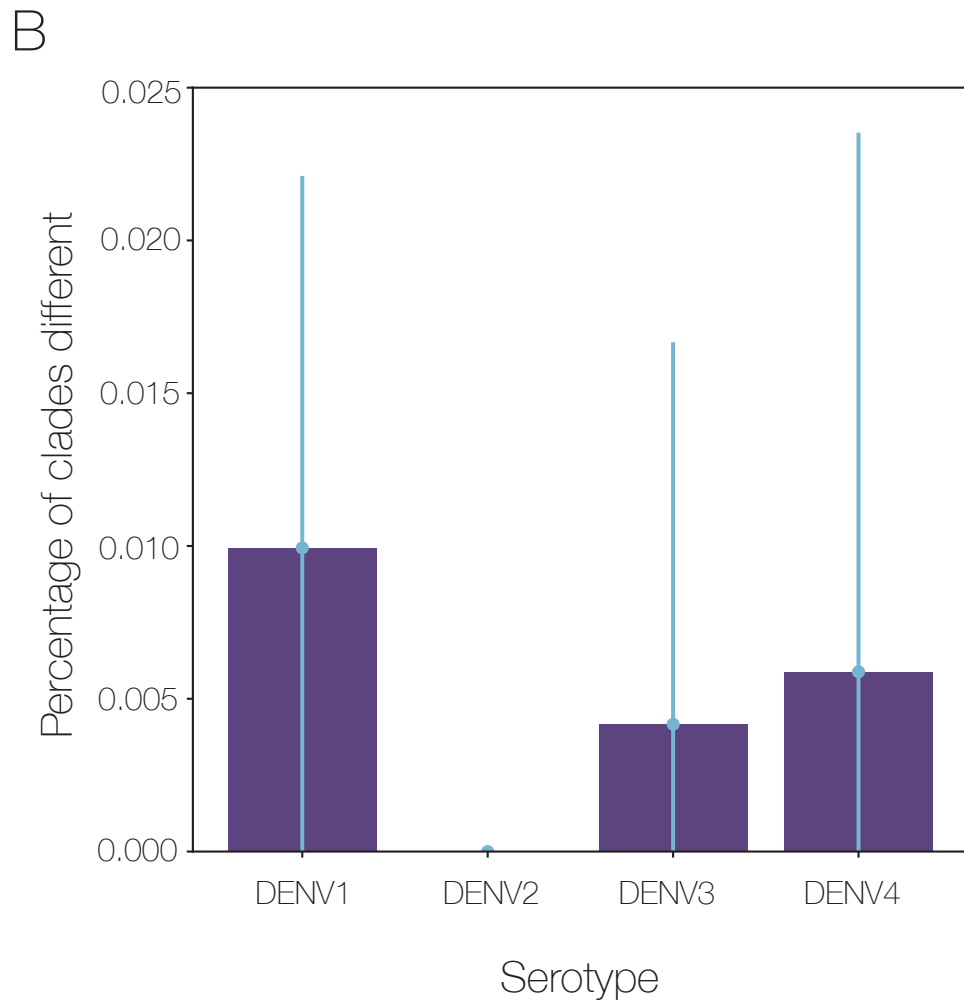

Supplement: S6 Fig — (A) Average correctness of genome detective assignments at different classification levels using artificially downsampled sequences across 5 replicates. Each line corresponds to a different classification level. Error bar is not visible. (B) Assessment of clade stability compared to different subsamples of the sequence data set. (PDF) [file pbio.3002834.s010.pdf]

A

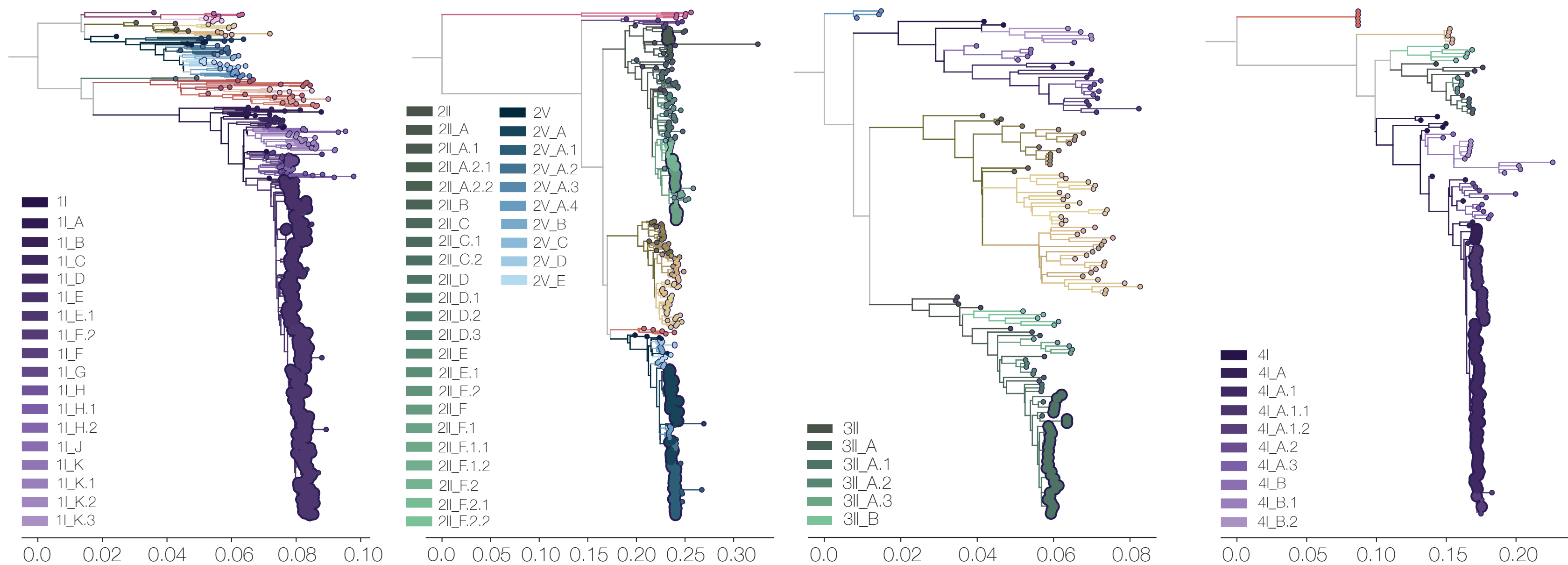

B

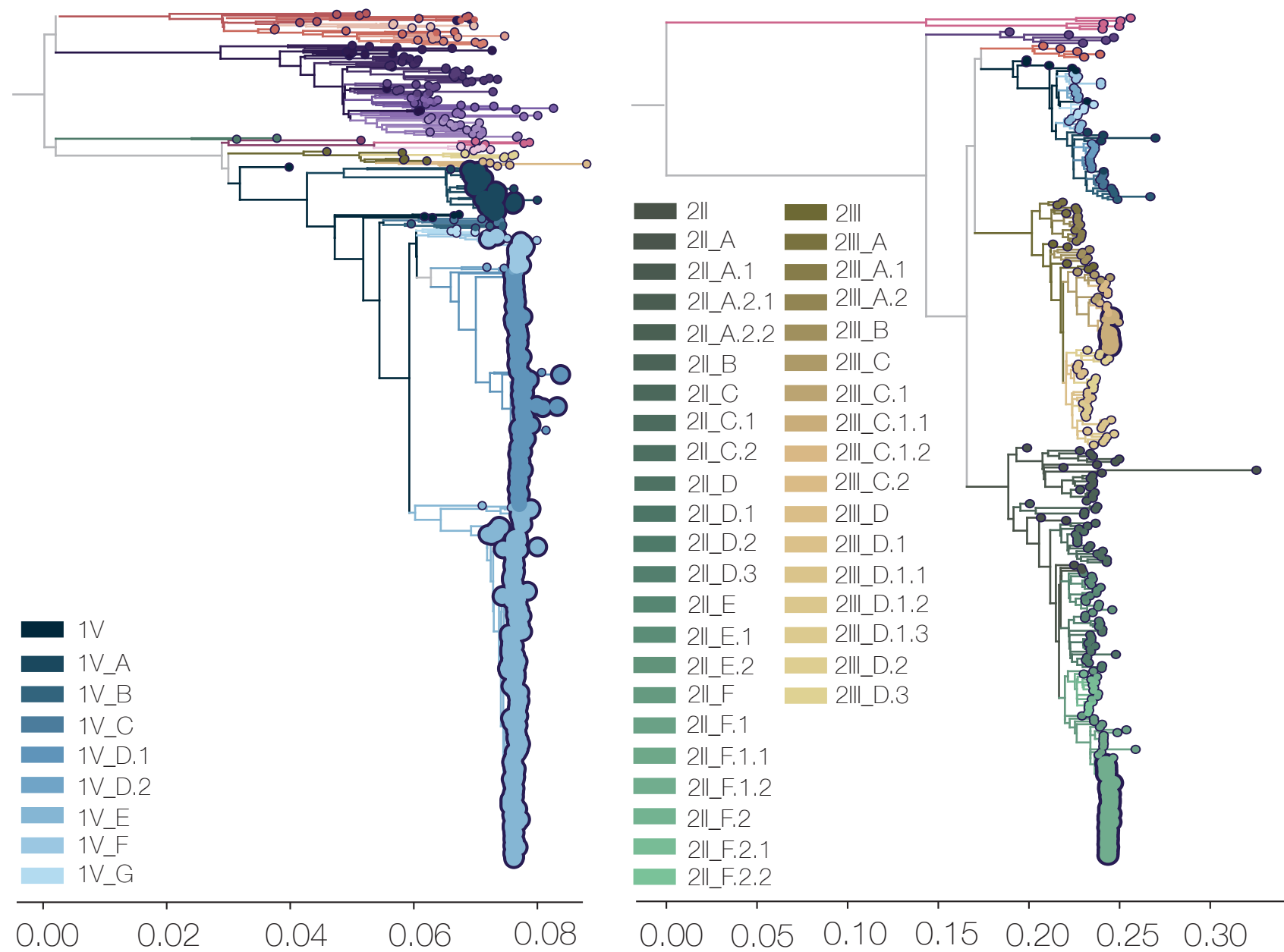

C

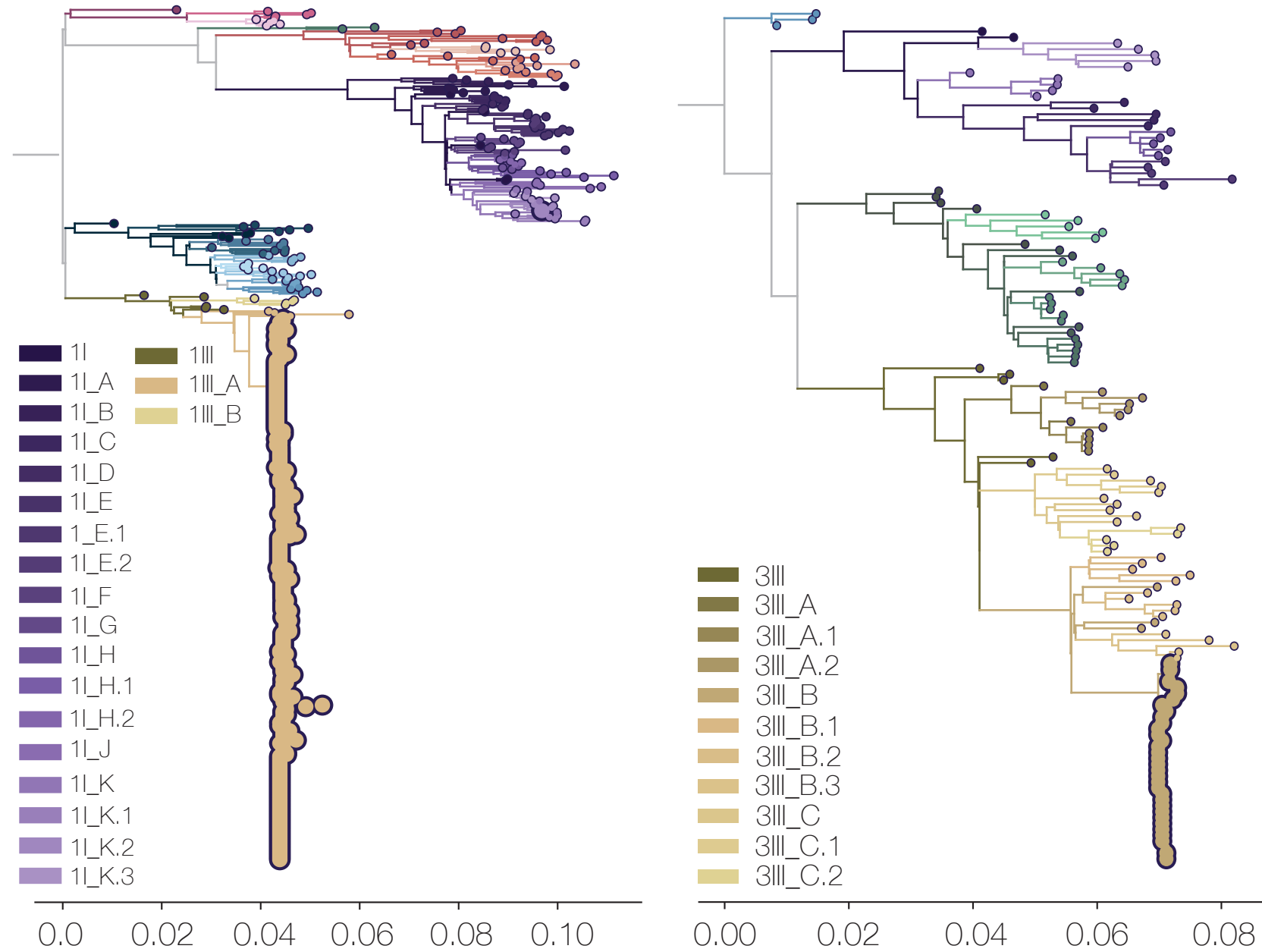

Supplement: S7 Fig — (A) DENV-1-4 whole genome sequences from Vietnam, time series 2010–2023. (B) DENV-1 and DENV-2 whole genome sequences from Brazil, time series from 2015–2023. (C) DENV-1 and DENV-3 E sequences from Tanzania. (PDF) [file pbio.3002834.s011.pdf]

A

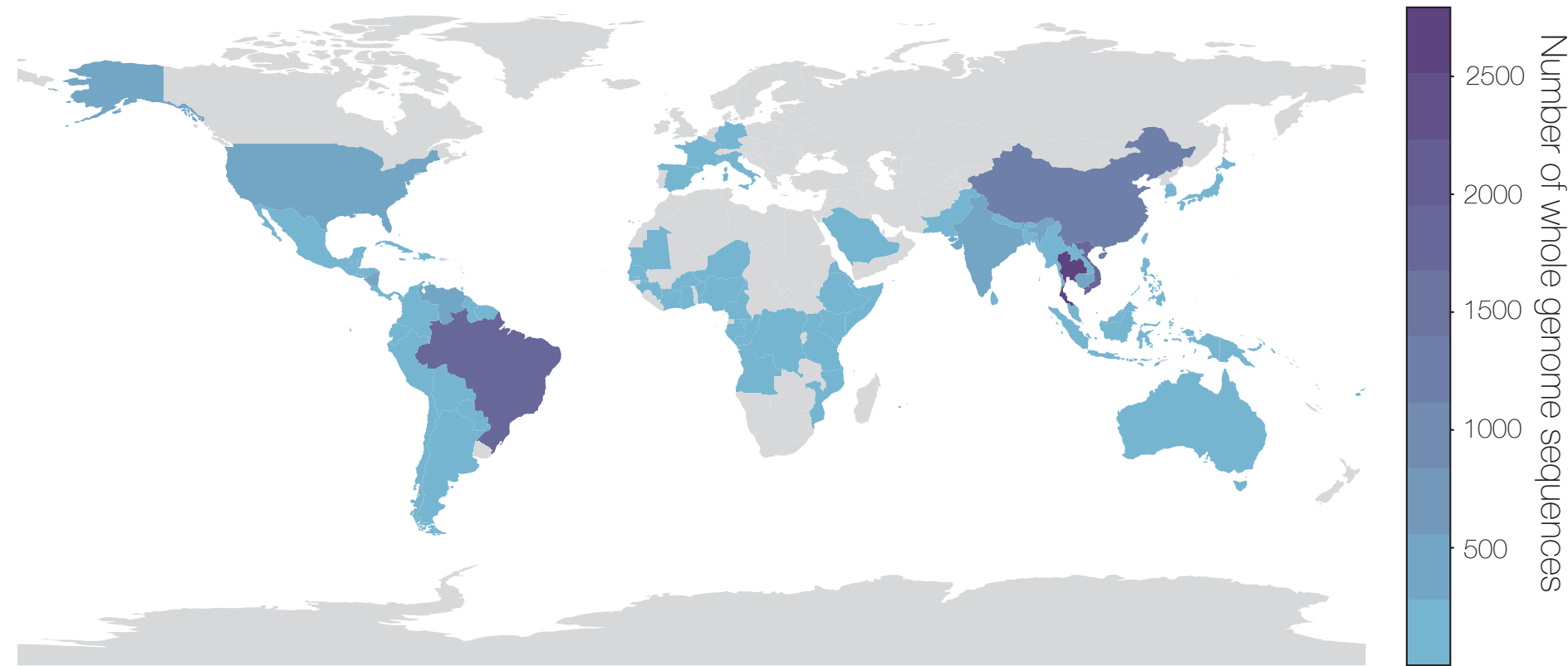

B

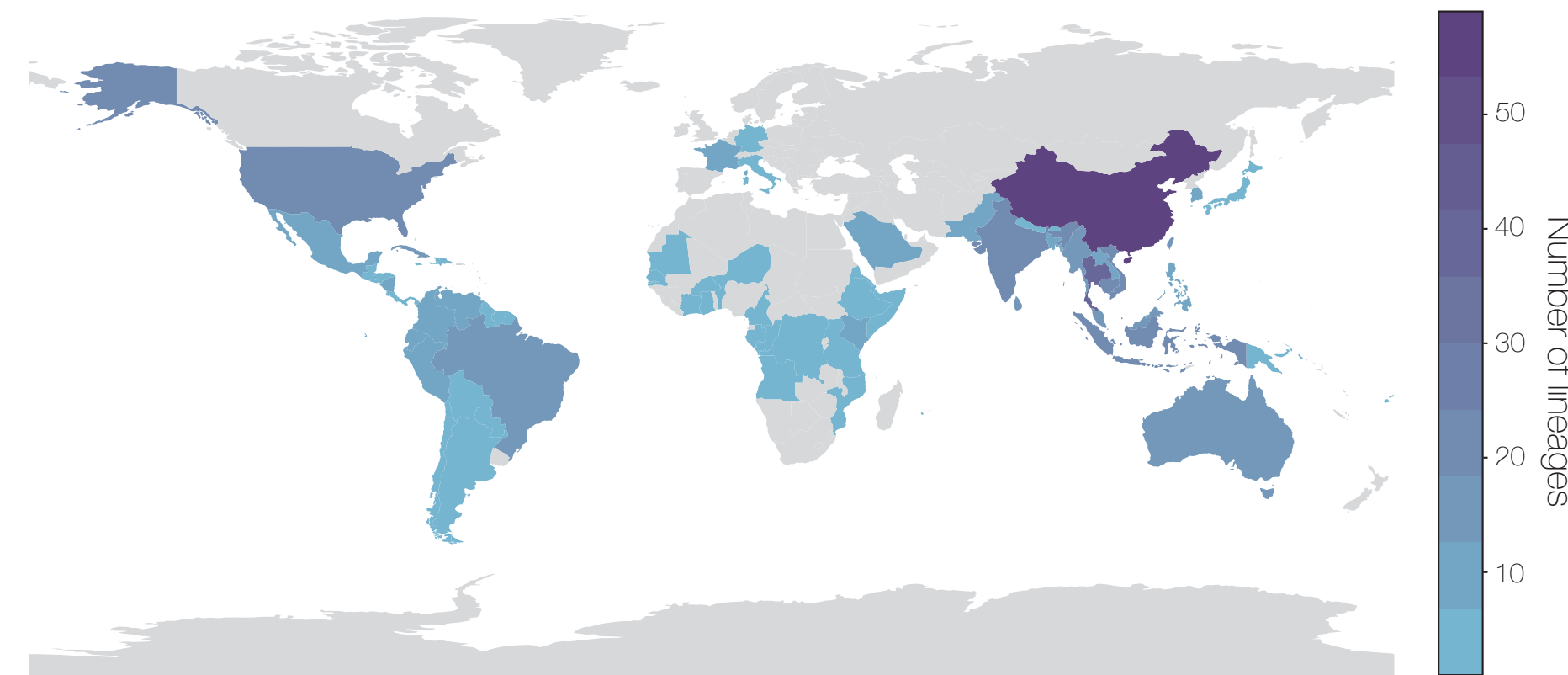

C

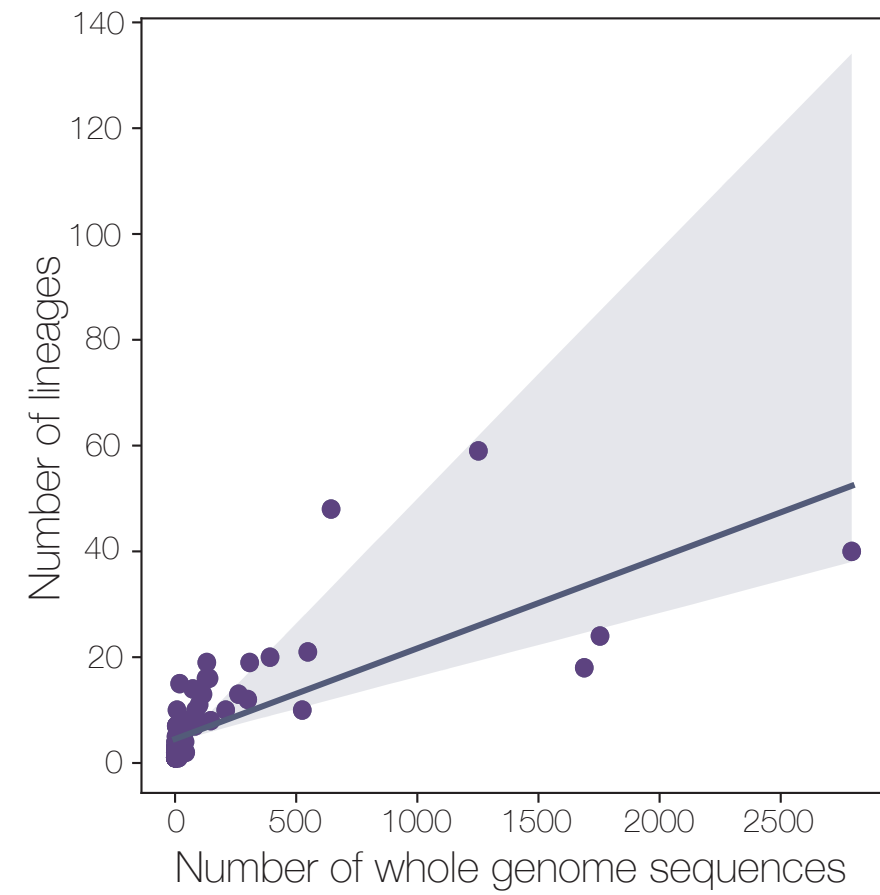

Supplement: S11 Fig — (A) Sampling location of whole genome sequences by country. (B) Number of lineages sampled in each country. (C) Linear regression of the number of whole genome sequences against the number of lineages in each country (p < 0.001). Base map layer downloaded from the Global Administrative Database (https://gadm.org/download_world.html). (PDF) [file pbio.3002834.s015.pdf]

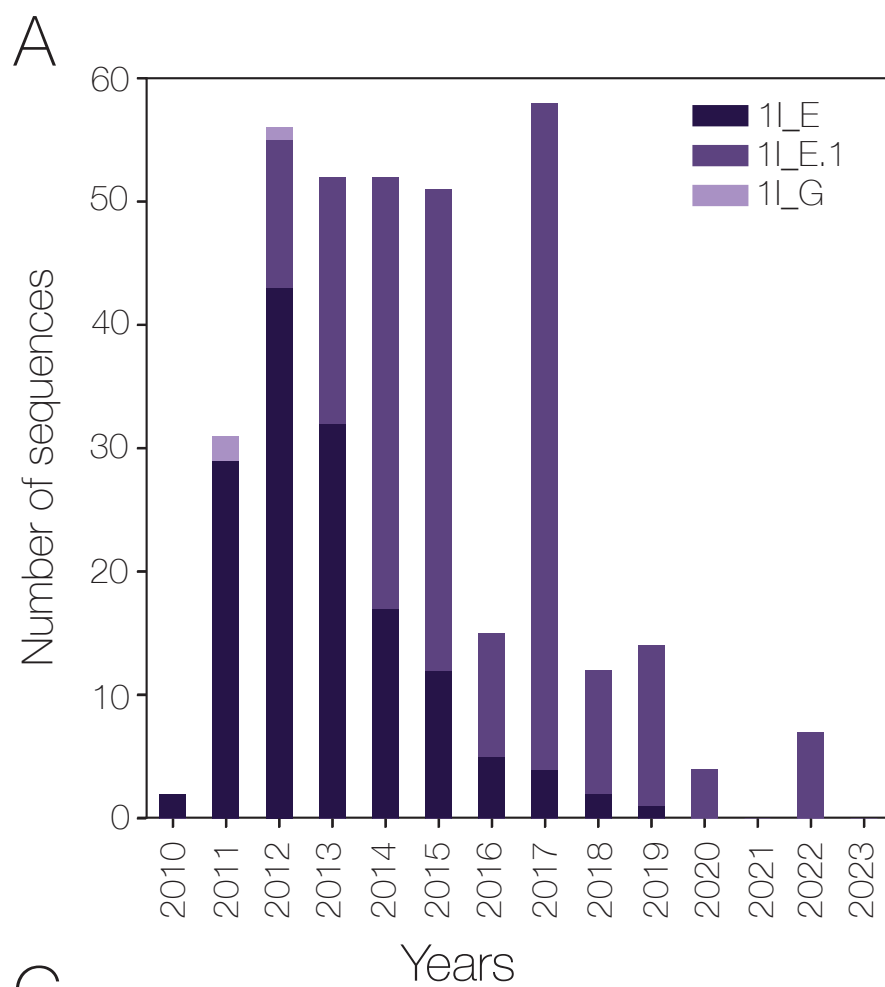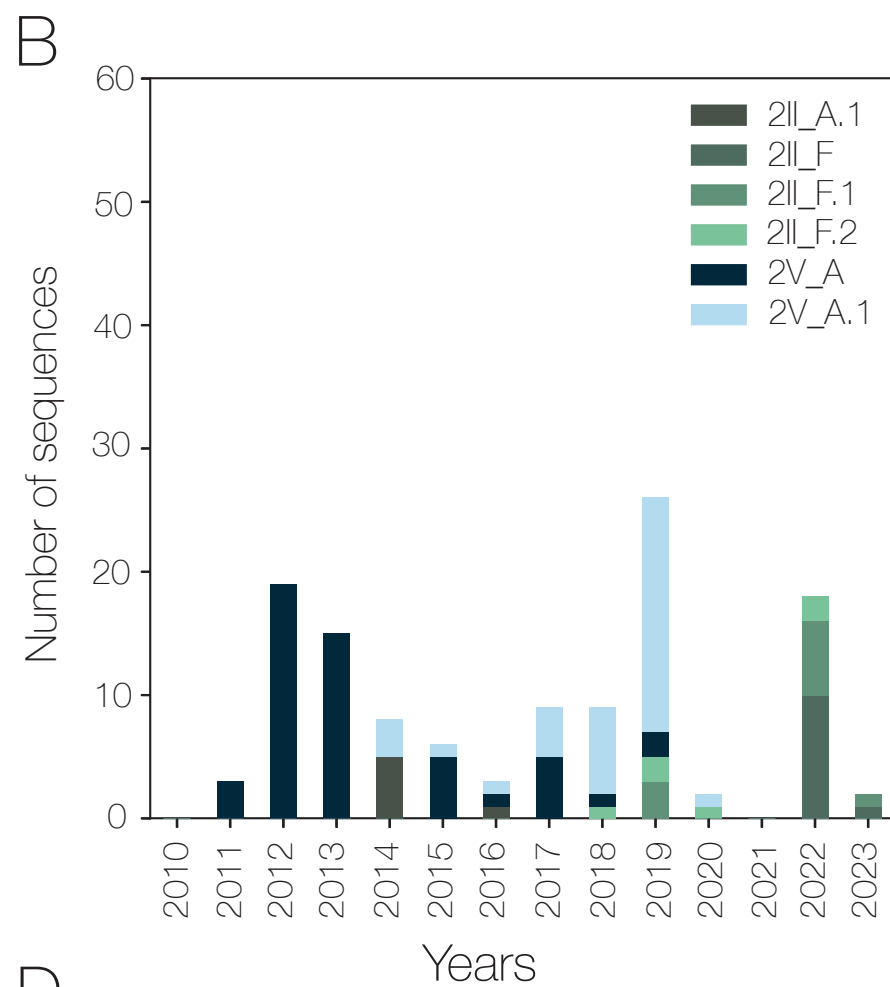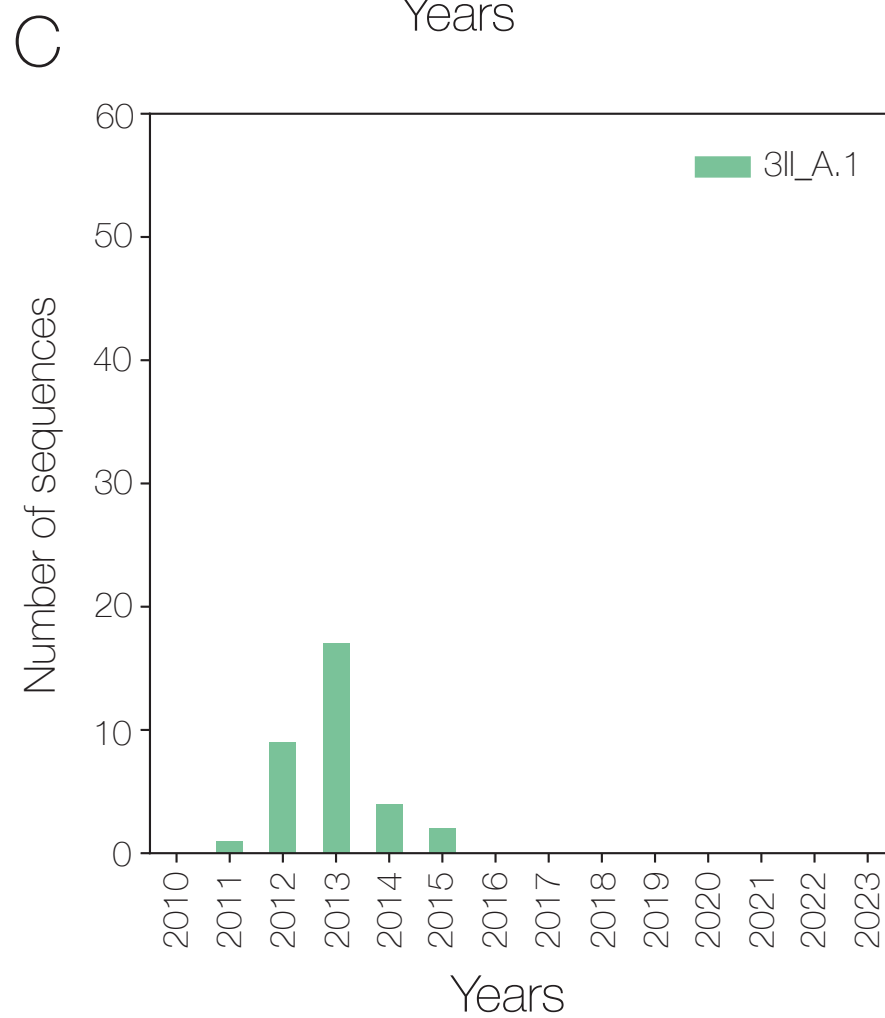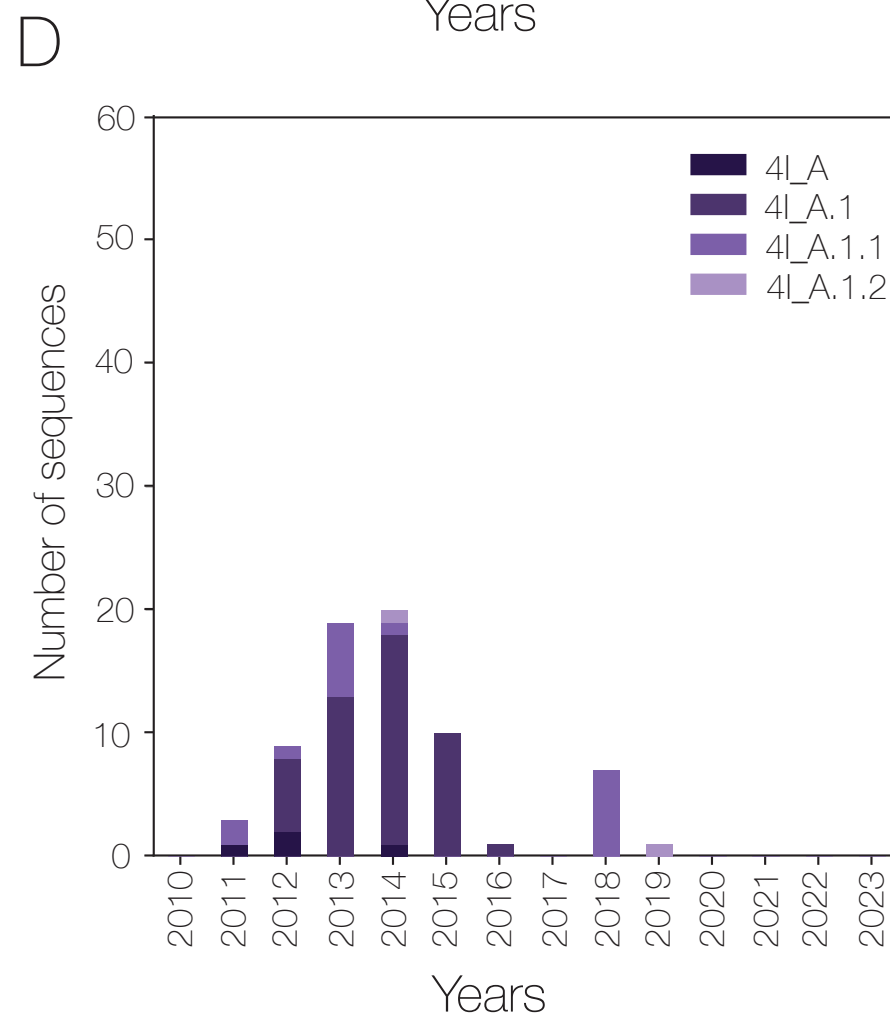

Supplement: S12 Fig — Number of dengue virus whole genome sequences mostly from Ho Chi Minh City, Vietnam assigned to each lineage over time for (A) DENV-1, (B) DENV-2, (C) DENV-3, and (D) DENV-4. (PDF) [file pbio.3002834.s016.pdf]

A

Rio Grande Do Sul

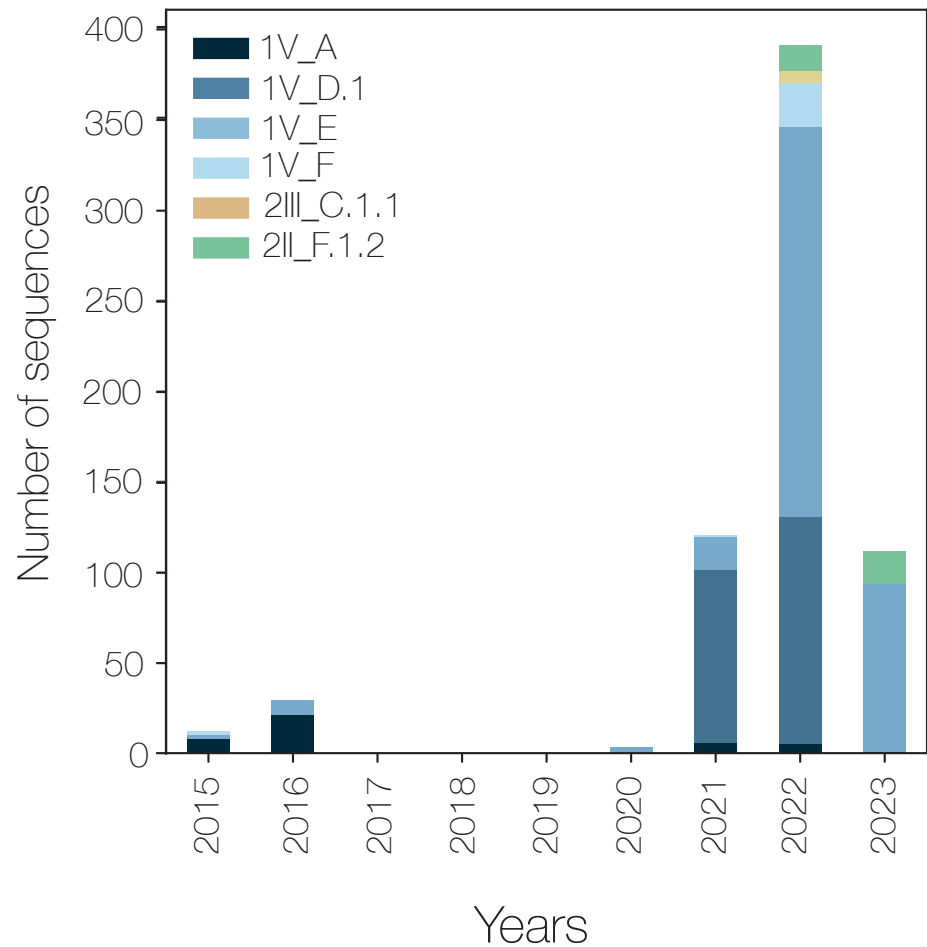

B

Rest of Brazil

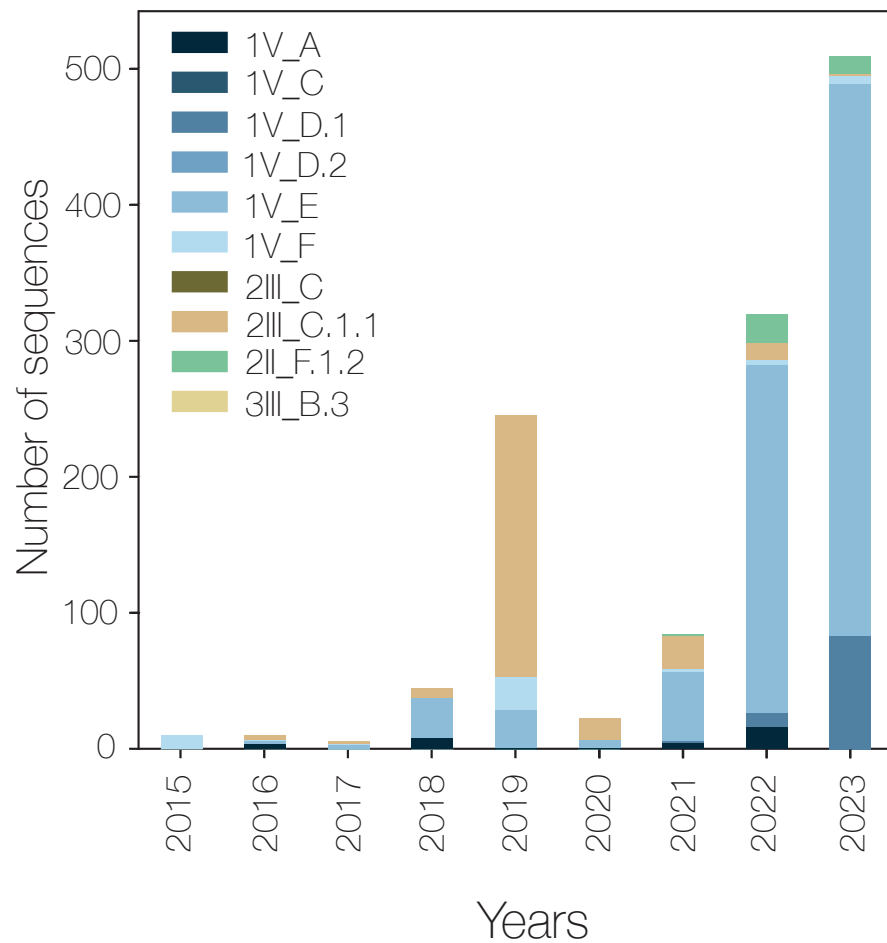

Supplement: S13 Fig — (A) Time series of whole genome sequences from Rio Grande do Sul, Brazil by year. (B) Lineage assignments of whole genome sequences from the rest of Brazil in this dataset (non-case study sequences from Rio Grande Do Sul have been removed). (PDF) [file pbio.3002834.s017.pdf]

A

1III\_A

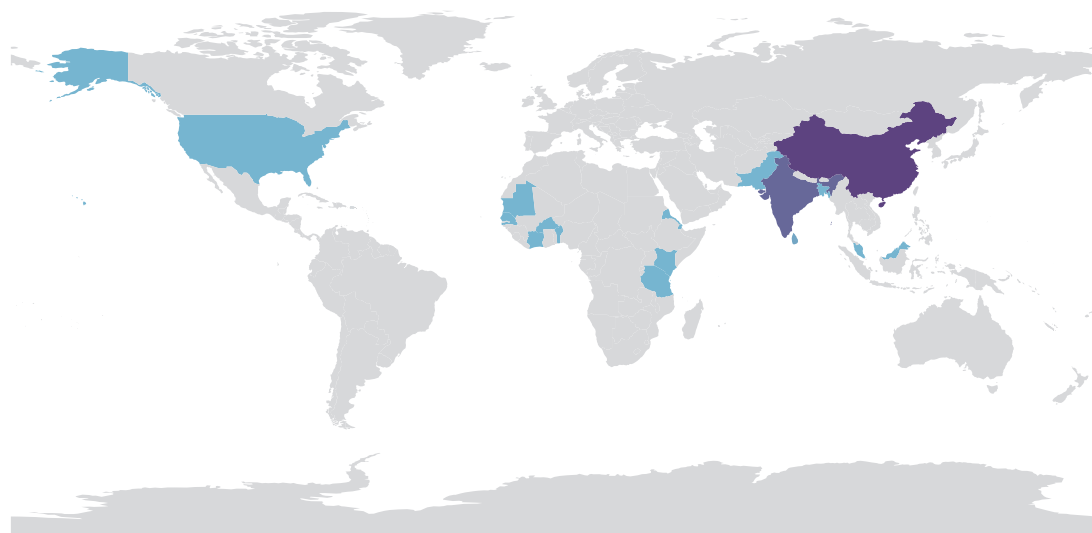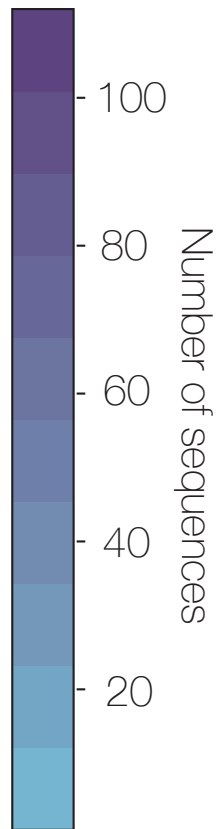

B

3III\_B

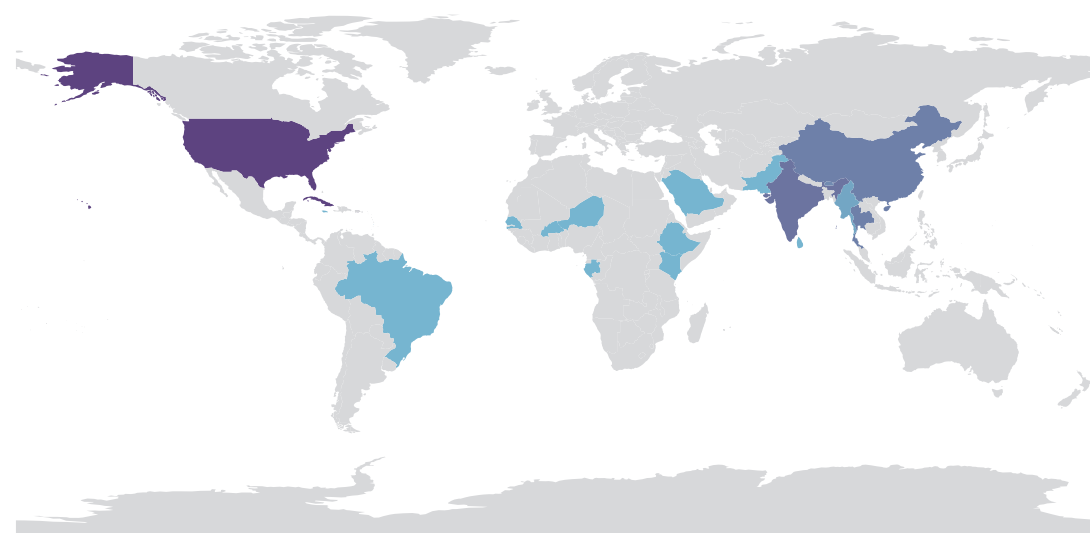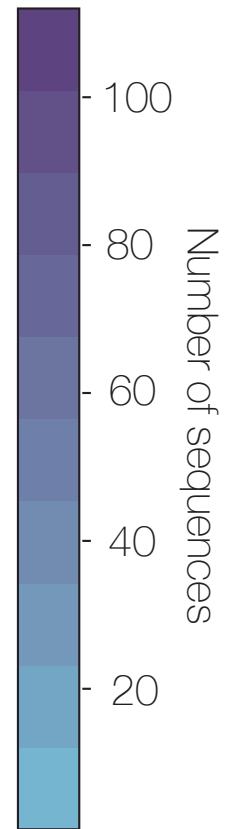

Supplement: S14 Fig — (A) Major lineage 1III_A which all DENV-1 sequences in these data sets are assigned to. (B) Major lineage 3III_B which all DENV-3 sequences are assigned to. All maps include all sublineages of each lineage, and colors show the number of whole genome sequences in the training dataset which are in each country or territory. Base map layer downloaded from the Global Administrative Database (https://gadm.org/download_world.html). (PDF) [file pbio.3002834.s018.pdf]
