# Supplementary material for: A new lineage nomenclature to aid genomic surveillance of dengue virus
Source: PLoS Biol. 2024 Sep 16;22(9):e3002834. doi: 10.1371/journal.pbio.3002834 (PMC11426435; doi:10.1371/journal.pbio.3002834)
Supplement: S3 Fig — All 4 putative lineages displayed in the top 2 trees are valid lineages as they meet all 3 criteria of branch length, clade size, and having a sister lineage at the same distance from the root in terms of node number. Invalid lineages are shown along the bottom, with the focal putative lineage shown in purple. (PDF) [file pbio.3002834.s007.pdf]

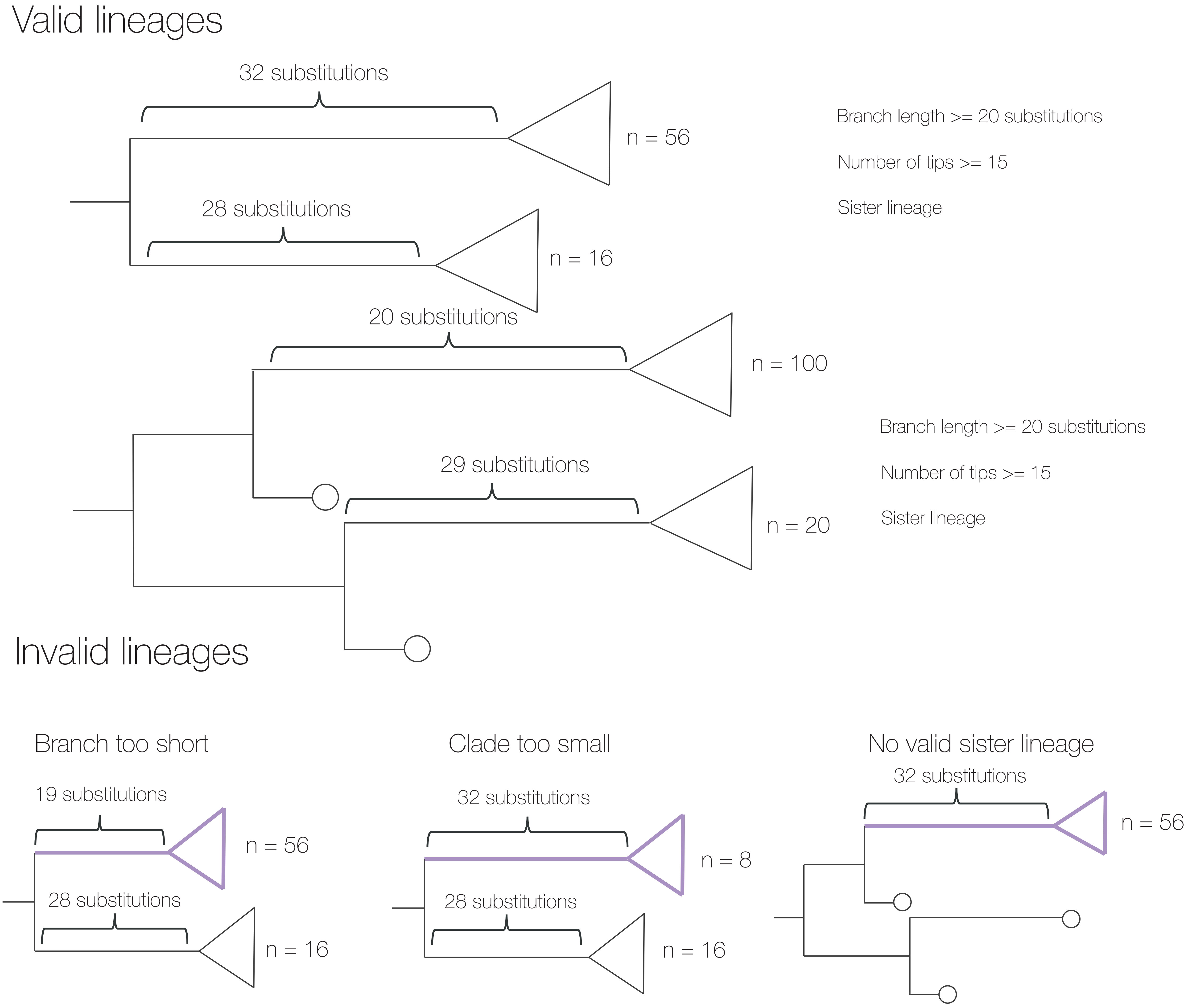
Valid lineages
32 substitutions
n = 56
28 substitutions
n = 16
Branch length >= 20 substitutions
Number of tips >= 15
Sister lineage
20 substitutions
n = 100
29 substitutions
n = 20
Branch length >= 20 substitutions
Number of tips >= 15
Sister lineage
Invalid lineages
Branch too short
19 substitutions
n = 56
28 substitutions
n = 16
Clade too small
32 substitutions
n = 8
28 substitutions
n = 16
No valid sister lineage
32 substitutions
n = 56
