# Supplementary material for: A new lineage nomenclature to aid genomic surveillance of dengue virus
Source: PLoS Biol. 2024 Sep 16;22(9):e3002834. doi: 10.1371/journal.pbio.3002834 (PMC11426435; doi:10.1371/journal.pbio.3002834)
Supplement: S8 Fig — The user can either upload a fasta file, or manually add sequences in the text field. (PDF) [file pbio.3002834.s012.pdf]

NO JOBS IN QUEUE

Dengue Virus Typing Tool

DENGUE VIRUS TYPING TOOL

Version 4.1

logged out message of the Day

Read more

DENGUE VIRUS TYPING TOOL

This tool is designed to use Blast and phylogenetic methods in order to identify the Dengue virus serotypes, genotypes and major lineages of a nucleotide sequence.

Note for batch analysis: The tool accepts up to 2000 sequences at a time.

INPUT

Submit one or more FASTA sequences to be typed individually. If you have raw NGS reads (short reads or long reads), please use the Genome Detective Platform to assemble first. Subtyping tools will be linked in the results.
Click here to load some sample data.

Sequence

CLICK OR DROP FILE

```
>MT832049.1|2012
ATGAACAACCAACGGAAAAAGACGGGTCGACCGTCTTTCAATATGCTGAAACGCGCGAGAAACCGCGTGTCAACTG
TTTCACAGTTGGCGAAGAGATTCTCAAAAGGATTGCTTTCAGGCCAAGGACCCATGAAATTAGTGATGGCTTTTAT
AGCATTCCTAAGATTTCTAGCCATACCTCCAACAGCAGGAATTTTGGCTAGATGGAGCTCATTCAAGAAGAATGGA
GCGATCAAAGTGTTACGGGGTTTCAAGAAAGAGATCTCAAACATGTTGAACATAATGAACAGGAGGAAAAGATCTG
TGACCATGCTCCTCATGCTGCTACCCACAGCCCTAGCGTTCCATCTGACCACCCGGGGAGGAGAGCCGCACATGAT
AGTTAGCAAGCTGGAAAGAGGAAAATCACTTTTGTTTAAGACCTCTGCAGGTGTCAACATGTGTACCCTTATTGCA
ATGGATTTGGGAGAGTTATGTGAGGACACAATGACCTACAAATGCCCCAGGATCACTGAGACGGAGCCAGATGACG
TTGACTGTTGGTGCAATGCCACGGAGACATGGGTGACCTATGGAACATGTTCTCAAACTGGTGAACACCGACGAGA
CAAACGTTCCGTCGCATTGGCACCACACGTAGGGCTTGGCCTAGAAACAAGAACCGAAACGTGGATGTCCTCTGAA
GGCGCTTGGAGACAAATACAAAAAGTGGAGACTTGGGCTCTGAGACACCCAGGATTCACGGTGATAGCCCTTTTTC
TAGCACATGCTATAGGAACATCCATCACCCAGAAAGGAATTATTTTCATTTTGCTGATGCTGGTAACTCCATCCAT
GGCCATGCGGTGTGTGGGAATAGGCAACAGAGACTTTGTGGAAGGACTGTCAGGAGCTACGTGGGTGGATGTGGTG
CTGGAGCATGGAAGTTGCGTCACTACCATGGCAAAAGACAAACCAACACTGGACATTGAACTCCTGAAGACGGAGG
TCACAAACCCTGCAGTCCTGCGCAAACTGTGCATTGAAGCTAAAATATCAAACACCACCACCGATTCGAGATGTCC
```

START FREE ANALYSIS

CLEAR

Log in or register to experience the advantages of a premium account.
