# Supplementary material for: A new lineage nomenclature to aid genomic surveillance of dengue virus
Source: PLoS Biol. 2024 Sep 16;22(9):e3002834. doi: 10.1371/journal.pbio.3002834 (PMC11426435; doi:10.1371/journal.pbio.3002834)
Supplement: S9 Fig — The results overview shows a short summary of the different assignments. (PDF) [file pbio.3002834.s013.pdf]

RESULTS

You may bookmark this page to revisit results of this job (**4fc274ef-e330-4d81-a811-ee48fe0500a9**) later.

NC_001477

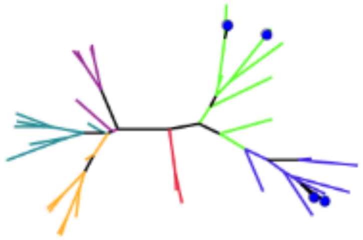

SUMMARY

Virus assignment	Genotype assignment	Major and minor lineage	Sequences count	Percentage	Legend
dengue virus type 1	I	E.2	1	14.3%	
		E	1	14.3%	
	IV	B.2	1	14.3%	
		A	1	14.3%	
dengue virus type 2	V	C	1	14.3%	
dengue virus type 3	II	A	1	14.3%	
dengue virus type 4	I	B.1	1	14.3%	
Total			7	100%	

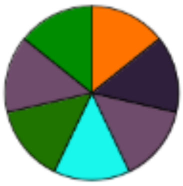

Name	Length	Blast/Serotype	Genotype, Major lineage, Minor lineage	Report	Genome
**MT832049.1|2012**	10179	dengue virus type 1	IV B 2	Report	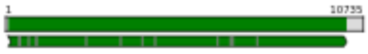
**3II.DQ675528.1998.Taiwan**	1305	dengue virus type 3	II A Related to but not part of 3	Report	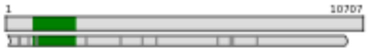
**MW946456.1|2001**	10925	dengue virus type 2	V C	Report	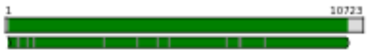
**ON123668.1|2018**	10164	dengue virus type 4	I B 1	Report	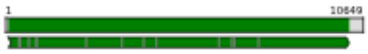
**MW315179.1|2016-06-06**	10179	dengue virus type 1	I E 2	Report	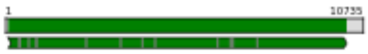
**MW265671.1|2012-04-29**	10179	dengue virus type 1	I E Could not assign	Report	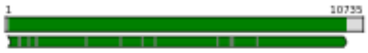
**MW265683.1|2009-01-06**	10179	dengue virus type 1	IV A	Report	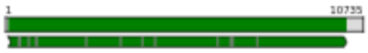

Download results: XML File Table (Excel format) Table (CSV format) Sequences (Fasta format)
