# Supplementary material for: A new lineage nomenclature to aid genomic surveillance of dengue virus
Source: PLoS Biol. 2024 Sep 16;22(9):e3002834. doi: 10.1371/journal.pbio.3002834 (PMC11426435; doi:10.1371/journal.pbio.3002834)
Supplement: S10 Fig — An example of the analysis details for a major lineage. (PDF) [file pbio.3002834.s014.pdf]

PHYLOGENETIC ANALYSIS DETAILS (MAJOR LINEAGE)

- Assignment: B
- Bootstrap support: 100.0, bootstrap inside 100.0, bootstrap outside 0.0
- Phylogenetic Tree (export as PDF, newick format)

TREE CONTROLS

Layout

Rectilinear

Transform

None

- [x] Show labels
- [x] Highlight clusters
- [x] Color branches

A

B

C

outgroup

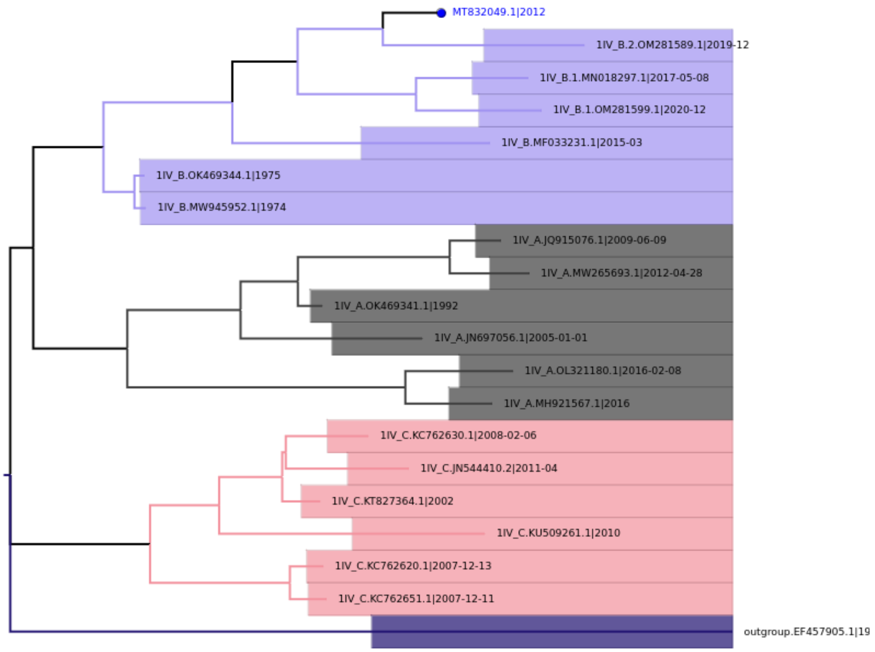

- View the Phylo Analysis Log file (Contains bootstrap values)
